# Synthesis and Biological Evaluation of a Series of New Hybrid Amide Derivatives of Triazole and Thiazolidine-2,4-dione

**DOI:** 10.3390/ph17060723

**Published:** 2024-06-03

**Authors:** Igor B. Levshin, Alexander Yu. Simonov, Alexey A. Panov, Natalia E. Grammatikova, Alexander I. Alexandrov, Eslam S. M. O. Ghazy, Vasiliy A. Ivlev, Michael O. Agaphonov, Alexey B. Mantsyzov, Vladimir I. Polshakov

**Affiliations:** 1 Gause Institute of New Antibiotics, 11 B. Pirogovskaya Street, 119021 Moscow, Russia; levshin@panavir.ru (I.B.L.); simonov-live@inbox.ru (A.Y.S.); ngrammatikova@yandex.ru (N.E.G.); 2 Bach Institute of Biochemistry, Federal Research Center of Biotechnology of the RAS, 119071 Moscow, Russia; alexvir@inbi.ras.ru (A.I.A.); 1072195050@rudn.ru (E.S.M.O.G.); agaphonov@inbi.ras.ru (M.O.A.); 3 Institute of Biochemical Technology and Nanotechnology, Peoples’ Friendship University of Russia (RUDN), 6 Miklukho-Maklaya Street, 17198 Moscow, Russia; chemistron@mail.ru; 4 Department of Microbiology, Faculty of Pharmacy, Tanta University, Tanta 31111, Egypt; 5 Faculty of Fundamental Medicine, Lomonosov Moscow State University, 27/1 Lomonosovsky Ave., 119991 Moscow, Russia; mantsab@gmail.com (A.B.M.); vpolsha@fbm.msu.ru (V.I.P.)

**Keywords:** antifungals, azoles, thiazolidine-2,4-dione

## Abstract

A series of hybrid compounds with triazole and thiazolidine nuclei connected by a linker has been synthesized and extensively studied. Various synthetic methods for the target compounds have been tested. A microbiological assessment of the obtained compounds was carried out on strains of pathogenic fungi *C. albicans*, *C. non-albicans*, multidrug-resistant *C. auris*, *Rhizopus arrhizus*, *Aspergillus* spp. and some dermatophytes and other yeasts. The lowest obtained MIC values for target compounds lie between 0.003 µg/mL and 0.5 µg/mL and therefore the compounds are not inferior or several times better than commercial azole drugs. The length of the acylpiperazine linker has a limited effect on antifungal activity. Some bioisosteric analogues were tested in microbiological analysis, but turned out to be weaker than the leader in activity. The highest activity was demonstrated by a compound with *para*-chlorobenzylidene substituent in the thiazolidine fragment. Molecular modelling was used to predict binding modes of synthesized molecules and rationalize experimentally observed SAR. The leader compound is twice more effective in inhibiting the formation of germ tubes by *Candida albicans* yeast cells compared to voriconazole. An increased level of Pdr5, an azoles drug efflux pump was observed, but the increase is lower than that caused by azoles. The results can be useful for further development of more powerful and safe antifungal agents.

## 1. Introduction

The problems caused by fungal lesions of internal organs have become increasingly widespread and important in recent years. At the end of October 2022, WHO appealed to the medical community because of the critical situation associated with pathogenic fungi due to the insufficiency of existing means of combating them, and the ever-growing resistance to the drugs used, as well as the emergence of new highly dangerous multi-resistant strains of fungi such as *Candida auris* [1]. The emergence of resistance to antifungal drugs is a global problem that needs to be addressed urgently. The most critical examples are the widespread growth and emergence of antifungal-resistant strains of *Candida glabrata* and *Candida auris* fungi and the identification of azole-resistant filamentous fungi *Aspergillus* spp. In addition, some problems remain with respect to some rarer mold fungi, such as *Mucor*, *Rhizopus*, *Fusarium*, for which there are still no effective and reliable antifungal drugs [2].

The creation of new antifungal drugs is a complex and lengthy process. The similarity of the fungal cell structure to the human one imposes serious limitations in terms of their toxic effect. One of the actively developing directions to overcome this circumstance is the synthesis of hybrid molecules based on triazole with multiple mechanisms of action directed at different targets of the microbial cell [3]. Modification of the triazole pharmacophore group is vital in developing antifungal agents because it offers broad-spectrum effectiveness, potency, minimal toxicity to mammalian cells, aids in managing resistance, favorable drug distribution, adaptable chemical structure, and proven clinical effectiveness against fungal infections [4].

**Figure 1:**
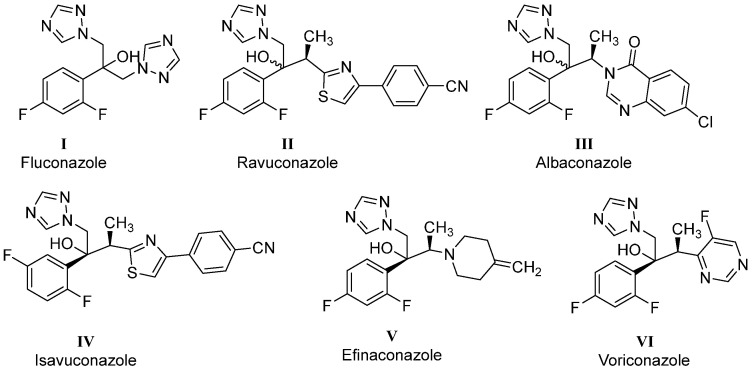
Structures of some modern commercial azole antifungal drugs.

Extension of the triazole pharmacophore group by an additional fragment, either directly or by a linker, is of significant interest. However, as experiments have shown, the obtained results do not always meet expectations and are often unpredictable [5]. In the years after the discovery of fluconazole (**I**, Figure 1) in 1986, many researchers have attempted to improve the microbiological and pharmacological properties of fluconazole by introducing substituents both into the triazole part of the molecule and by replacing one of the triazole heterocycles with other heterocyclic or aliphatic structures [6,7]. This has resulted in new drugs such as ravuconazole (**II**), albaconazole (**III**), izavuconazole (**IV**), efinaconazole (**V**), and voriconazole (**VI**) introduced into healthcare in many countries (Figure 1). 

Hybridization of the triazole ring can profoundly influence antifungal activity by enhancing binding affinity to the target, specificity, resistance mitigation, and potency enhancement, ultimately contributing to the development of more effective antifungal therapies. For instance, ravuconazole’s optimized triazole moiety boosts its effectiveness against diverse fungal pathogens, while albaconazole’s structure enhances its specificity to fungal ergosterol, reducing off-target effects. Isavuconazole’s adjustments improve its binding to ergosterol and pharmacokinetics for the treatment of invasive infections. Efinaconazole’s changes heighten its antifungal potency and nail bed penetration, while voriconazole’s modifications widen its activity spectrum against fungal pathogens [3,8,9].

Ergosterol is the main component of the fungal cell membrane that regulates the permeability, fluidity, and integrity of the membrane. Azoles block the synthesis of ergosterol by competitively inhibiting lanosterol 14α-demethylase (CYP51), which is the key enzyme that catalyzes the oxidative removal of the 14α-methyl group of lanosterol to form 14,15-unsaturated intermediates of ergosterol biosynthesis [10,11,12]. Selective inhibition of CYP51 leads to depletion of ergosterol reserves and accumulation of lanosterol. Ultimately, it leads to the inhibition of fungi proliferation. The biological assessment of both the drugs themselves and their analogues conducted in order to detect more effective molecules, is constantly underway and the search for new highly effective molecules is actively continuing all over the world.

In our previous report [13], we showed the topical antifungal action of 3,5-disubstituted thiazolidine-2,4-diones on pathogenic yeast fungi of the *Candida* spp. and dermatophytes *Microsporum canis* and *Trichophyton mentagrophytes*, presumably due to the destructive effect on the cell wall of a fungus. Some of the synthesized compounds exhibited high antifungal activity, both fungistatic and fungicidal, and led to morphological changes in the *Candida* yeast cell wall.

**Figure 2:**
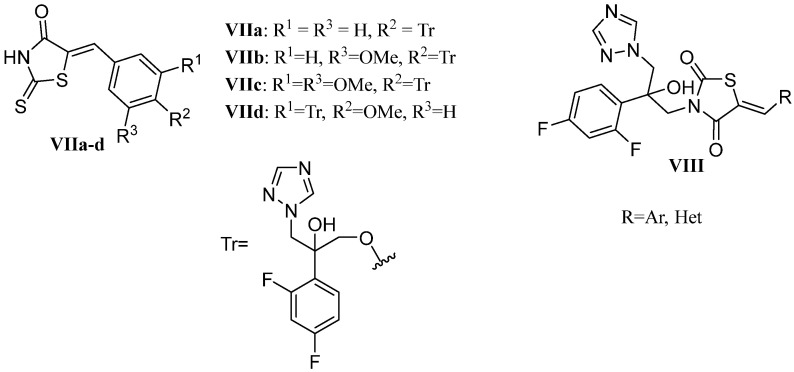
Some biologically active agents bearing hybrid scaffolds described in the literature [14,15].

Several options for attaching a thiazolidine molecule to a triazole pharmacophore group have been described in the literature. Borat and colleagues [14] described the addition of a triazole pharmacophore via the para-alkoxy group of the phenyl of (5-benzylidene)rhodanine (**VIIa**–**d**, Figure 2). However, the resulting compounds failed to show high fungicidal activity. Later, Wu et al. [15] presented hybrids of triazole with thiazolidine-2,4-dione with various arylidene and heteroarylidine substituents (**VIII**, Figure 2).

**Figure 3:**
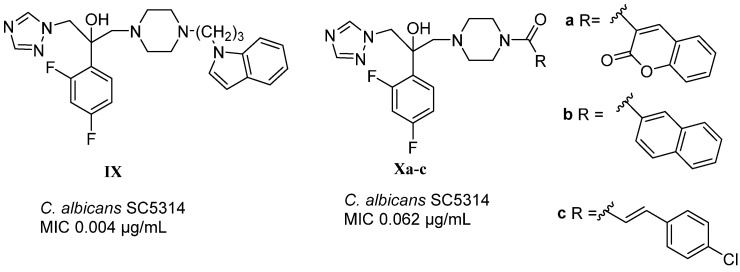
Structures of hybrid piperazine substituted triazoles described in the literature [15,16,17].

The inclusion of cyclic amine linkers into a hybrid structure with a triazole pharmacophore is widely used in the exploratory work of researchers in medical chemistry [15]. A number of compounds recently obtained by Zhu et al. (**IX**, **Xa**,**b**, Figure 3) demonstrated high antifungal activity not only in vitro, but also on animals with disseminated *Candida* infection [16]. The activity of the molecules largely depended on the substituent adjacent to the carbonyl group of the piperazine amide (Figure 3). The patent [17] describes a hybrid amide styrene derivative **Xc** (Figure 3), which, in addition to anti-*Candida* activity, exhibits activity against the “black” fungus of the *Zygomycetes* genus. This fact is especially important in connection with the recent sharp spike in infection in India in 2021, where the number of infected with the “black” fungus, according to the Ministry of Health of India, exceeded 28 thousand people [18].

**Figure 4:**
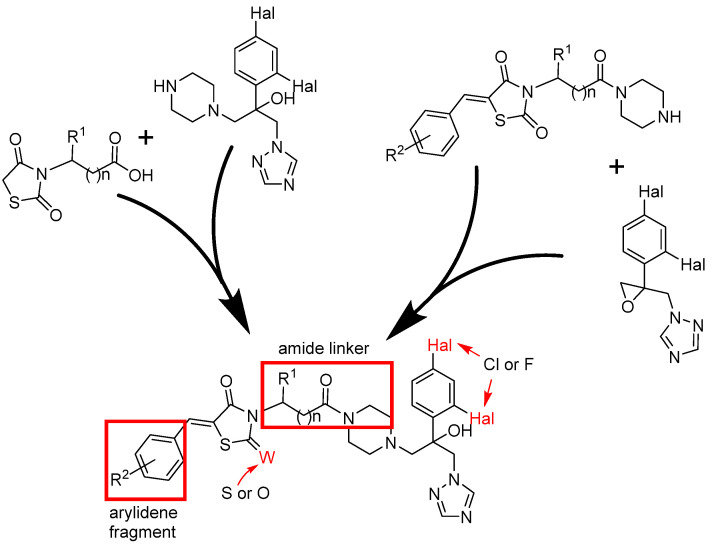
The newly designed antifungal scaffold and dissection of it into four domains for SAR study.

The purpose of our work was to study the approaches to the synthesis of new hybrid derivatives of thiazolidine-2,4-dione (TZD) and triazole containing a piperazine-based amide linker (Figure 4). The target 3-substituted derivatives of thiazolidine-4-one were obtained in several different ways, depending on the starting reagents: either from the corresponding carboxylic acid with piperazine-containing triazole fragment (Method A) or from piperazine amide by reaction with oxirane (Method B).

To study the structure–activity relationship, four domains of modifications were identified: (1) substituents in the phenyl core of the benzylidene-TZD part; (2) exocyclic substituent in the thiazolidine core; (3) piperazine amide linker with a different number of methylene units; (4) halogen in the triazole-containing part of the molecule (Figure 4).

**Figure 5:**
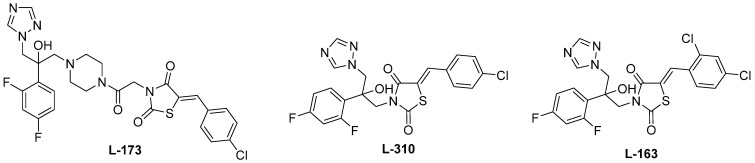
Structural formulas of previously synthesized hybrid compounds **L-173**^20^, **L-310**^16^, **L-163**^16^.

Synthesis, physicochemical and biological characteristics of some of the previously obtained hybrids were described by us in previous reports [19,20,21,22]. This paper describes various approaches to the synthesis of triazole/thiazolidine-2,4-diones hybrids and gives a deeper study of the biological properties of the compounds with various substituents in the thiazolidine ring. In addition, some thio-substituted thiazolidine-4-ones and the leader compound, formerly reported as **L-173** (Figure 5), were studied. The docking of amide hybrids was carried out and the key pharmacophoric features were described. The results obtained for **L-173** were compared with the previously described compound **L-310**, which contains no linker between the fragments [15] (Figure 5). Since a number of compounds had high antifungal activity and were effective against fluconazole-resistant clinical isolates, we reproduced *para*-chloro substituted **L-310** and 2,4-dichloro substituted **L-163** for analysis and comparison of microbiological activity. It has been previously found by molecular docking studies that these molecules interact with CACYP51 mainly through hydrogen bonding and hydrophobic interactions.

Microbiological evaluation of all the obtained derivatives of thiazolidine and triazole was carried out on strains of pathogenic yeast-like fungi *Candida albicans*, *C. non-albicans*, *C. auris*, filamentous fungi *Aspergillus* spp., dermatophytes *T. rubrum*, *M. canis*, and *Rhizopus* spp.

## 2. Results and Discussion

**Scheme 1:**
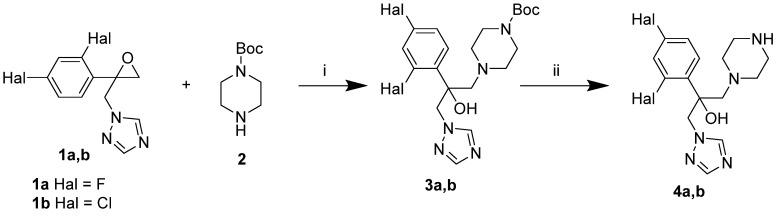
Synthesis of piperazine-derived intermediates **4a**,**b**. Reagents and conditions: (i) EtOH, Et_3_N, 80 °C; (ii) CF_3_COOH/CH_2_Cl_2_, r.t. or HCl/ethyl acetate, r.t.

To start the synthesis, triazole-containing piperazine derivatives **4a**,**b** were obtained by reaction of Boc-piperazine **2** with either 2,4-difluorophenyl-substituted oxirane **1a** (commercially available) or 2,4-dichlorophenyl-substituted oxirane **1b**, which have been synthesized before [23,24]. The reaction was carried out by reflux in ethanol with triethylamine. Then, the protecting group was removed by trifluoroacetic acid or hydrogen chloride in ethyl acetate (Scheme 1). For the next steps, the salts of **4a** and **4b** or their freebases are equally suitable.

**Scheme 2:**
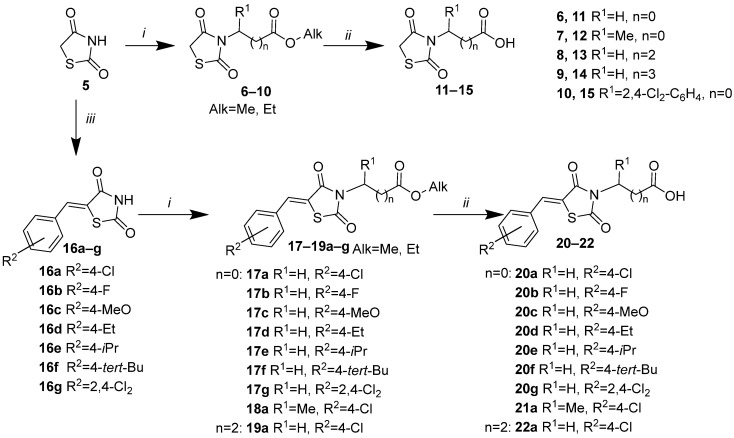
Synthesis of 3- and 5-substituted thiazolidine-2,4-diones. Reagents and conditions: (i) RCHBr(CH_2_)_n_COOAlk/K_2_CO_3_, acetonitrile, acetone or DMF, r.t. or 50 °C; (ii) CH_3_COOH, HCl, reflux; (iii) substituted benzaldehyde, CH_3_COOH, MeNH_2_, reflux.

Thiazolidine-substituted carboxylic acids **11**–**15** (Scheme 2) and their 5-arylidene derivatives **20**–**22** were obtained from thiazolidine-2,4-dione (**5**) by reproduction of the methods described in the literature [25,26,27] and presented in the Supporting Information.

The preparation of 5-arylidene derivatives **16a**–**g** by Knoevenagel reactions of **5** with aromatic aldehydes was carried out in an acetic acid medium with methylamine as a catalyst [28] (Scheme 2). The alkylation of TZDs was performed by appropriate alkylating agents in THF, acetonitrile, or DMF with potassium carbonate as a base. The temperature regime in the reaction was determined by the reactivity of the alkylating agent: room temperature was suitable for methyl 2-bromo-2-(2,4-dichlorophenyl)acetate, but ethyl bromoacetate required refluxing in DMF. Acid hydrolysis by refluxing in a mixture of acetic and hydrochloric acids led to the corresponding acids **20**–**22**.

**Scheme 3:**
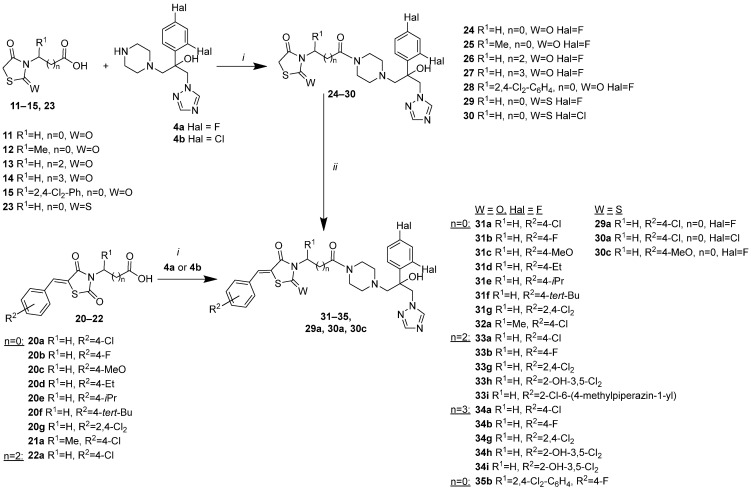
Synthesis of hybrid piperazine amides **24**–**30** and **31**–**35**. Reagents and conditions: (i) SOCl_2_, Et_3_N, r.t.; or TBTU, CH_2_Cl_2_; (ii) C_2_H_5_OH, Et_3_N, reflux.

The target hybrid amides **24**–**30** were synthesized from the carboxylic acids **11**–**16** and piperazines **4a**,**b**. 5-Arylidene-substituted hybrid derivatives were prepared in two ways, depending on the structure of the TZD derivative and the triazole intermediate used. By Method A, the carboxylic acids **11**–**15** were transformed into acyl chlorides by action of thionyl chloride and immediately brought into reaction with *N*-substituted piperazines **4a**,**b** in CH_2_Cl_2_ at room temperature (Scheme 3). Similarly, sulfur-containing analogues **29** and **30** were synthesized from 2-(4-oxo-2-thioxothiazolidin-3-yl)acetic acid (**23**) and piperazines **4a**,**b**. By the same method, 5-arylidene substituted **20a**–**g**, **21**–**22a** were converted into piperazinyl-amide derivatives **31a**–**g**, **32a**, **33a**–**g**, **34a**–**g** and **35b** in good yields (see Table 1). The use of TBTU as a condensation agent resulted in a much lower yield of 32%. Some 3-substituted 5-arylidenthiazolidine-2,4-diones **22a**, **22g**, **26a** were obtained and characterized in previously published work [13,19,25,26,27] (the methods are described in the Supporting Information).

To further confirm the structure of the compound obtained, an alternative synthetic route was carried out from unsubstituted thiazolidine–triazole hybrids **24**–**30** by Knoevenagel reaction with aromatic aldehydes (Method K) under milder conditions: ethanol with piperidine as the catalyst [29]. This method provided compounds **29a**, **30a**, and **30c**, expanding the range of target hybrid compounds.

**Scheme 4:**
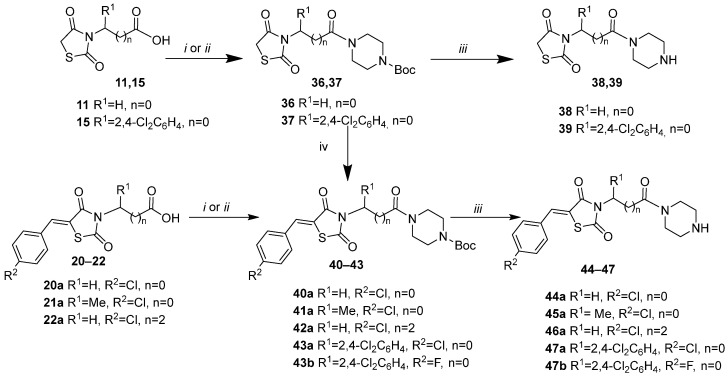
Synthesis of acylpiperazine derivatives. Reagents and conditions: (i) SOCl_2_, CH_2_Cl_2_, then 1-Boc-piperazine, Et_3_N, r.t.; (ii) CDI, CH_2_Cl_2_, then 1-Boc-piperazine, Et_3_N, r.t.; (iii) CF_3_COOH/CH_2_Cl_2_, r.t. or HCl/ethyl acetate, r.t.; (iv) aromatic aldehydes, EtOH, piperidine, reflux.

Condensation of (2,4-dioxothiazolidin-3-yl)-substituted acids **11**,**15** with Boc-piperazine provided compounds **36**, **37** (Scheme 4), and after acidic deprotection, the corresponding piperazines **38**, **39** were obtained. Similarly, amide derivatives **40**–**43a** were obtained from the corresponding 5-arylidene-substituted (thiazolidine-2,4-dione-3-yl)carboxylic acids **20**–**22** (Scheme 4). The Knoevenagel reaction seems to be suitable for any step of the synthesis: **40a** and **43a** were obtained from **36** and **37**, respectively (iv, Scheme 4) (methods are described in the Supporting Information) [22].

Compound **42a** was also prepared from the corresponding 3-carboxylic acid **22a** using CDI and Boc-piperazine in methylene chloride at room temperature. The yield in this case was slightly lower than by the acyl chloride method, but remained quite high (the technique is described in the Supporting Information).

**Scheme 5:**
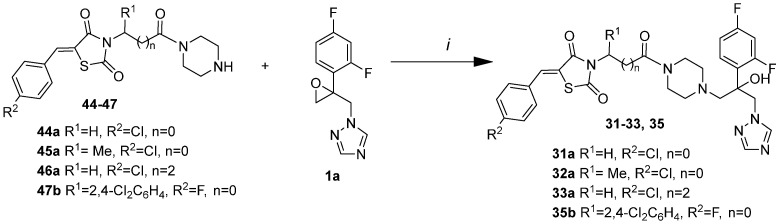
Synthesis of acylpiperazine hybrids by Method B. Reagents and conditions: (i) dry toluene/*N*-methylpyrrolidone, Et_3_N, reflux.

Some hybrid derivatives, i.e., **31**–**33**,**35**, were obtained from triazole-containing oxirane **1a** and 5-(benzylidene)-3-substituted thiazolidine-2,4-diones **44**–**47** by reflux in toluene with the addition of *N*-methylpyrrolidone and triethylamine (Method B) (Scheme 5).

**Scheme 6:**
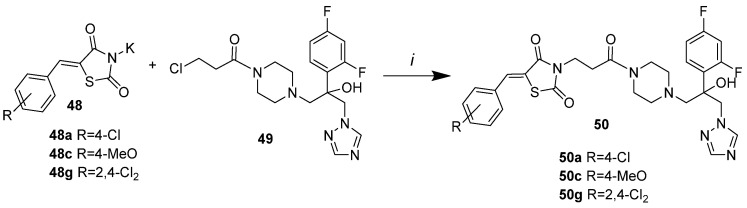
Synthesis of target hybrid derivatives by alkylation of K-salts of 5-arylidene thiazolidine-2,4-diones **48a**,**c**,**g** with 3-chloropropanoic acid amide **49**. Reagents and conditions: (i) dry DMF, KI, 100 °C.

To obtain hybrids **50a**,**c**,**g** with a linker of two methylene units, an entirely different method was used (Method C), consisting of the alkylation of K-salts of 5-arylideneTZDs **48a**,**c**,**g** by substituted 3-chloro-1-(piperazin-1-yl)propan-1-one **49** in DMF (Scheme 6). The yields of the target products **50a**,**c**,**g** were significantly lower than by other methods. A summary of the all acylpiperazine hybrids is presented in Table 1.

The structure of the obtained compounds was confirmed by NMR spectroscopy and mass spectrometry. In the ^1^H and ^13^C NMR spectra, for all hybrid derivatives containing no arylidene substituent in the TZD ring **24**–**30**, characteristic resonances of the triazole ring are observed (chemical shifts of ^1^H nuclei 8.31 and 7.75 ppm and ^13^C nuclei 145.4 and 150.9 ppm). For the phenyl group containing two fluorine atoms, ^1^H aromatic CH signals are observed at 6.99, 7.16, and 7.41 ppm and the corresponding ^13^C resonances at 110.9, 104.0, and 129.7 ppm. In the ^13^C spectrum of such substituents, characteristic doublets of doublets for the CF carbon atoms are also observed at ~159 and ~162 ppm. The coupling constants for these signals are ~246 Hz for 1J(CF) and ~12 Hz for 3J(CF). In hybrids without an arylidene substituent, signals of the thiazolidine CH_2_ group are also observed at 4.20–4.22 ppm for ^1^H and ~32–36 ppm for ^13^C resonances. All 5-substituted benzylidene derivatives contain a characteristic resonance of the olefin proton Ar-CH= at 7.93–8.01 ppm for ^1^H and 131.0–132.0 for ^13^C. These chemical shift values correspond to the thermodynamically stable Z-isomers [20,21].

The structures of key compounds **31a** (**L-173)**^20–23^ and **L-310**^16^ were unambiguously confirmed by analysis of a series of 2D NMR experiments, including the homonuclear ^1^H-^1^H DQF-COSY (Figures S7 and S12 in Supporting Information) and ROESY (Figures S8, S13 and S14 in Supporting Information) experiments, as well as the heteronuclear ^13^C-^1^H HSQC experiments (Figures S9 and S15 in Supporting Information) and ^13^C-^1^H HMBC (Supplementary Figure S15), performed at the natural abundance of the ^13^C isotope. Analysis of these 2D spectra allowed us to completely assign the ^1^H and ^13^C signals of the compounds **L-173** and **L-310** (Table 2) and to confirm their structure. Information on the assignment of the signals in the ^1^H and ^13^C spectra was further used in the interpretation of the 1D spectra of all other compounds studied.

## 3. Microbiology

### 3.1. Characterization of the Compounds’ Biological Activity and SAR

A comparative study of the spectrum of the antifungal action of the new compounds was carried out in vitro by the method of using double micro-dilutions in broth, using standard strains and clinical isolates. For references, standard preparations of Amphotericin B, ketoconazole (Ketoconazole, Sigma-Aldrich, St. Louis, MO, USA) and fluconazole (Fluconazole, Sigma-Aldrich, St. Louis, MO, USA) were used, as well as the control strain of *Candida parapsilosis* (ATCC 22019). The minimal inhibitory concentration (MIC) was taken as the lowest concentration of sample solutions at which at least 80% growth inhibition was observed. All experiments were repeated three times.

Initially, we tested compounds **3a**, **4a**, **L-310**, and **L-163**, which were described in the literature [15,23]. Hybrid derivatives **L-310** and **L-163** [15] showed moderate microbiological activity against the tested strains (Table 3). The manifestation of the activity of the Boc-protected **3a** on the *C. parapsilosis* strain was unexpected because of the weak activity of its analog **3b** without a Boc group.

3-Substituted derivatives of thiazolidine-4-one **24**–**30** without an arylidene substituent failed to exhibit any antifungal effect. In addition, the introduction of a 4-chlorobenzylidene fragment into the structure did not lead to a noticeable activity. However, in the presence of a 2,4-dichlorophenyl substituent in the amide linker chain, compounds **47a** and **47b** were noticeably active against *C. parapsilosis*, reaching a MIC value of 0.25–0.5 µg/mL (Table 4) and had slightly improved activity in dematophyte strains (MIC 8–16 µg/mL). These compounds were studied on fluconazole-resistant clinical isolates of the genus *Candida* spp. (Table 5).

The hybrid compounds **24**–**30** bearing no arylidene fragment in the thiazolidine ring were inactive against all the strains used (Table 6). When an arylidene substituent is introduced, activity on a wide range of pathogens appears. For reference, fluconazole, itraconazole, and amphotericin B were used.

As follows from Table 6, the activity of the *para*-chloro-benzylidene derivative **31a (L-173)** was the highest on *C. parapsilosis*, *M. canis* and *T. rubrum* (MIC 0.03 µg/mL) and *Aspergillus* spp. (MIC 1–2 µg/mL). The introduction of a methyl substutuent into the amide linker (**32a**) slightly reduced activity against *Candida* and dermatophytes. The same effect was observed upon increasing the length of the methylene chain from C2 (**33a**) to C3 (**34a**). Replacement of halogen atoms in the benzylidene residue for a methoxy group (compounds **30c**, **31c**) sharply reduced the activity of *Aspergillis* strains without changing the activity against *Candida* spp. and dermatophytes, which remain high with MIC of 0.03–0.06 µg/mL. Similar results were observed in the sulfur analog (**29a**) with a *para*-chlorine benzylidene substituent. On all strains of fungi, the activity of the compound **30a** with a dichlorophenyl substituent in the triazole part was slightly lower than the difluorine thio analog **29a** and much lower than the leader molecule **31a** with an oxygen atom at position 2 of the thiazolidine ring.

An in vitro comparative evaluation of the new compounds against fungal strains of dermatophytes with fluconazole and Amphotericin B as references showed that the main compounds under study are not inferior to widely used commercial preparations in terms of the activity. Only **31a** and **31g** with halogen substituents in phenyl ring showed the high activity (MIC of 0.015 µg/mL) against *M. canis* and *T. rubrum*.

Thus, the compounds have a wide spectrum of antifungal activity, which is promising for use in clinical practice for the treatment of diseases caused by fungal infections. In addition, the activity of most of the compounds against *Candida parapsilosis* is even superior than that of fluconazole and itraconazole. In addition, the new compounds, unlike Fluconazole, inhibit the growth of dermatophytes *M. canis B-200* and *T. rubrum 2002* while still being effective against *Candida* species. A comparative study of close analogues of the selected leader **31a (L-173)** was conducted on clinical isolates, including strains resistant to itraconazole and ketoconazole (Table 7). There are advantages of the new compounds in relation to various strains of *Candida* spp., including the resistant ones (*C. albicans* 604M and *C. albicans* 8) or insensitive to modern commercial azole drugs (*C. tropicalis* 3019, *C. glabrata* 61L, and *C. krusei* 432M). In terms of activity against *Candida* spp., compound **31a** showed MIC values close to ketoconazole and itraconazole, and is even better than ketoconazole and itraconazole against resistant strains of *C. albicans* 604M and *C. albicans* 8. Since the compounds are hybrids with an additional mechanism of action on a pathogenic cell, the possibility of developing resistance is significantly reduced. In contrast, a constantly growing resistance to fluconazole is recorded. In addition, ketoconazole has significant toxicity, so the new compounds are good competitors.

Compound **31a** (**L-173**) has been tested in the Hans Knöll Institute (HKI) on an expanded range of clinical isolates, including the following types: *Candida* spp., *Aspergillus* spp., *Fusarium petrolifilum*, *Lomentospora petrolifilum*, *Rhizopus arrhizus*, and *Scedosporium apiospermum* (Table 8).

In most cases, compound **31a (L-173)** was not inferior in terms of activity to the reference drug Voriconazole. In relation to some of the most problematic strains of fungi *Rhizopus arrhizus* (MIC 0.25 mg/L) and *C. auris* (MIC 2 mg/L), the results were even slightly better than Voriconazole (4 and 8 mg/L respectively). The results obtained confirm the prospects for continuing the study of leader molecule **31a (L-173)**.

### 3.2. Tests In Vivo

In order to assess the effect of lead compound on the formation of the germinal tube of *C. albicans*, which is the first stage of the development of tissue-invasive hyphal form found in systemic infection, an experiment on mice was conducted. The inhibitory activity against mycelial outgrowth correlates better with the therapeutic efficacy of drugs against *C. albicans*, so this test does reflect the degree of antifungal activity in vivo [30]. To compare the efficiency of suppressing the *C. albicans* cells, germination in the glands of mice by different doses of compound **31a (L-173)** and voriconazole, the percentage of yeast cell germination in each of these groups of animals was calculated in relation to control group D (injected with 20% DMSO). Then, the percentage of *C. albicans* cell germination suppression was calculated as 100% minus percentage of germination (Table 9).

As follows from Table 9, administered at a dose 2.4 times lower than voriconazole (5 mg/kg vs. 12 mg/kg), compound **L**-**173** suppressed the growth tube formation by *C. albicans* yeast cells more effectively (22.33 against 17.00%). Administered in equal doses (12 mg/kg), the compound was also superior to voriconazole (30.86% vs. 17.00%). With an increase in the dose of compound **31a** to 25 mg/kg, a stronger inhibitory effect was also noted (41.04%).

## 4. Molecular Modeling

**Figure 6:**
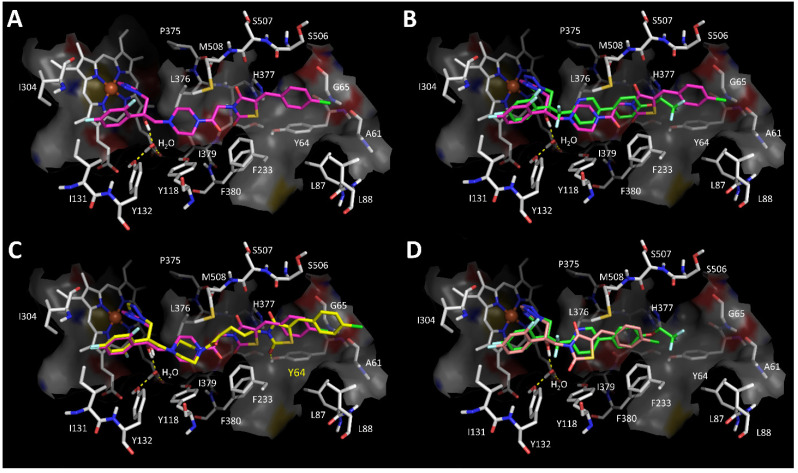
Binding modes of synthesized ligands in the active site of *C. albicans* CYP51 revealed by docking: (**A**) Ligand **31a**; (**B**) Superimposition of docked pose of **31a** (magenta) over the crystallographic conformation of VT1161 (green); (**C**) Superimposition of docked poses of **31a** (magenta) and **34a** (yellow); (**D**) Superimposition of docked pose of **L-310** (brown) over the crystallographic conformation of VT1161 (green). Hydrogen bonds are shown by dashed yellow lines; heme and selected residues of the receptor are depicted by sticks. A fragment of the binding site’s molecular surface is shown.

Docking studies were conducted to characterize the binding modes of the synthesized triazole-containing compounds in the active site of CYP51. The lead compound **31a (L-173)** demonstrated the expected orientation of the triazole core with a hydrogen bond formed between the hydroxyl group of the ligand and the water molecule coordinated by the heme and Y132 (Figure 6A). A thiazolidinedione arm mimicked the extended shape inherent in the 3-phenylpyridine chain of the crystallographic ligand VT1161 (Oteseconazole) and protruded through the substrate access channel toward the solvent-exposed protein surface (Figure 6B). None of the three carbonyl oxygens formed hydrogen bonds with the receptor, resulting in a poor stabilization of the polar 2-(piperazine-1-yl)-2-oxoethylthiazolidine-2,4-dione fragment in the lipophilic environment of the pocket. This effect was manifested as a complete loss of potency by compound **24** lacking benzylidene moiety (Table 6, here and below data for *C. parapsilosis* ATCC 22019 are interpreted). The benzylidene 4-chlorophenyl ring formed essential hydrophobic interactions with residues A61, Y64, G65, L87, and L88 at the gate to the substrate access channel.

Modifications of the structure accompanied by minor perturbations in the extended shape of the thiazolidinedione arm were tolerated: **32a** with the methylated linker and the 2-thio analog **50a** retained potency. In contrast, the bulky 2,4-dichlorophenyl group of **35b** did not fit within the substrate access channel, rendering the compound inactive. A linker extension by homologation (compounds **50a**, **33a**, **34a**) shifted the position of the benzylidene fragment toward the solvent-exposed region of the binding site, but essential hydrophobic interactions with the gate motif residues were mainly preserved (Figure 6C; Figure S1 in Supporting Information). Compounds with propylene and butylene linkers can pick the second hydrogen bond formed between the thiazolidinedione carbonyl and hydroxyl group of Y64 (Figure 6C; Figure S1). Enthalpic gain from this hydrogen bond can partially compensate for the increased entropic loss occurring upon binding the ligands with longer flexible alkyl chains, and explain comparable potencies of **31a** and **33a**. Substituents of the benzylidene phenyl ring modulated the interactions with the residues of the gate motif. Bulky substituents were not tolerated; this is presumably due to steric clashes (compounds **31f**, **33i**,**h**, **34i**,**h**). *para*-Chlorophenyl and 2,4-dichlorophenyl were the most versatile options featuring active compounds for all four tested alkyl linkers (compounds **31a**, **33**–**34a**, and **33**–**34g**).

The thiazolidinedione arm of **L-310** was shorter than that of **31a** due to the lack of *N*-acetylpiperazine fragment. Consequently, benzylidene moiety was shifted deeper inside the substrate access channel, disrupting hydrophobic interaction with the gate motif residues. The benzylidene(thiazolidine-2,4-dione) moiety of **L-310** bioisosterically mimicked 3-phenylpyridine chain of VT1161: the 4-chlorophenyl fragment fitted in the position of the oxyphenyl and thiazolidinedione ring superimposed over the pyridine with carbonyl oxygen matching position of the nitrogen (Figure 6D).

In order to confirm whether the interpretation of the SAR drawn based on the docking in the active site of *C. albicans* CYP51 SC5314 is valid for CYP51 from *C. parapsilosis* ATCC 22019, analysis of protein homology was conducted. An alignment of amino acid sequences revealed 75% identity between the enzymes (Figure S2 in Supporting Information). To further investigate the conservation of residues within the active site, a three-dimensional homology model of *C. parapsilosis* CYP51 was built and compared to the crystallographic structure of CYP51 from *C. albicans*. Structure-based alignment demonstrated 93% identity of residues within 6 Å around the crystallographic ligand. Three substitutions were revealed: 304 I > V was found next to the heme binding site; 87 L > M and 506 S > T occurred at the entrance to the substrate access channel (Figure S3). These results support that a docking-based interpretation of SAR proposed here for CYP51 from *C. albicans* SC5314 can be reliably transferred to *C. parapsilosis* ATCC 22019. The CYP51 gene has not yet been sequenced for strains of *A. niger*, *A. fumigatus*, *M. canis*, and *T. rubrum* studied in this work. However, as shown in Table 6, the SAR profiles for *M. canis* and *T. rubrum* are close to those of *C. parapsilosis*.

## 5. Biochemistry

**Figure 7:**
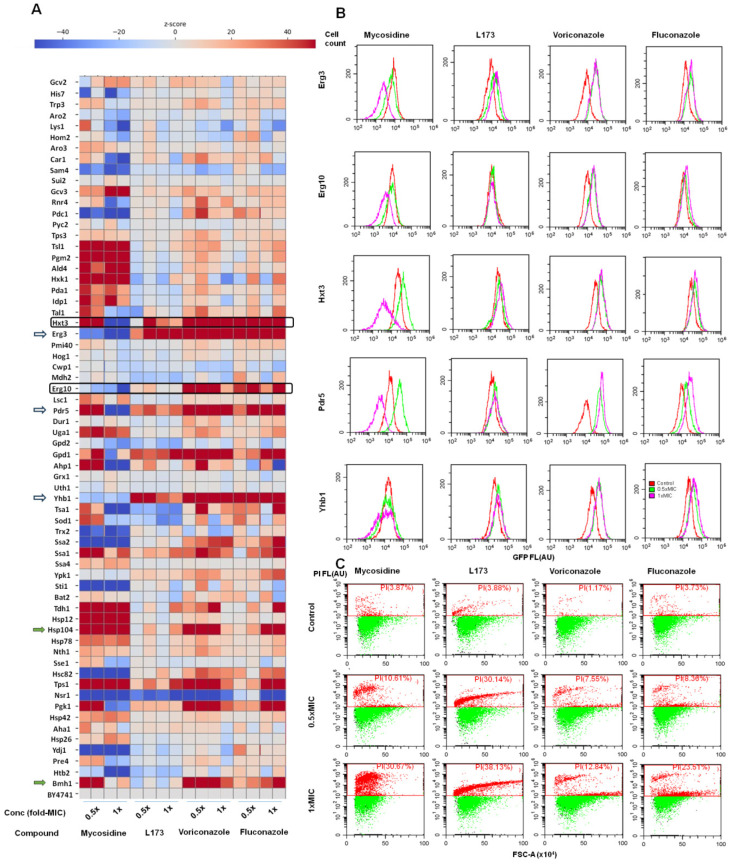
Effects of **L-173** and reference azole and thiazolidine compounds on the abundance of GFP-fusion proteins and staining with propidium iodide. (**A**) Flow cytometric analysis of GFP fluorescence. Red indicates increased abundance and blue indicates decreased. Dark arrows highlight changes that are similar between the azoles and **L-173**, black boxes indicate changes that are similar between **L-173** and Mycosidine, and green arrows highlight unique features of **L-173**. Each concentration was tested in duplicate; (**B**) distributions of GFP fluorescence of indicated proteins; (**C**) flow-cytometric-based scatter plot of cell size vs. propidium iodide (PI) fluorescence, a measure of cell membrane permeability. PI in red denotes the % of cell positive for PI staining. Mycosidine MIC = 15 µg/mL, 0.5 × MIC = 7.5 µg/mL, **L-173** MIC = 62 µg/mL, 0.5 × MIC = 31 µg/mL, Voriconazole MIC = 0.04 µg/mL, 0.5 × MIC = 0.02 µg/mL, Fluconazole MIC = 15 µg/mL, 0.5 × MIC = 7.5 µg/mL.

In order to assess whether classic azole compounds, thiazolidine antifungals and the hybrid compounds display distinct responses from fungal cells, we used the SCRAPPY method [31] to compare the previously published data on voriconazole and mycosidine [13] and obtained new data for **L-173**. The SCRAPPY method involves testing an array of 65 strains of yeast, which produce GFP-fusions of various proteins under the control of their native promoters, thus allowing for the monitoring of how a cell up- or downregulates specific proteins in response to various bioactive compounds. Our previous results showed that mycosidine and the azoles had considerably differing response patterns. Tests at the minimal inhibitory concentration (MIC) and half-MIC show that the response to **L-173** is highly similar to the azole drugs. This is noted by a general lack of changes in the levels of numerous proteins, including those that change abundance in response to Mycosidine. Notable similarity to azoles is also observed in the upregulation of Erg3 and Yhb1. Also, we observed an increased level of Pdr5, an azoles drug efflux pump (Figure 7A). Importantly, the increase caused by **L-173** is lower than by azoles (Figure 7B), suggesting the possibility that **L-173** has lower affinity to the multi-drug efflux system, although this requires additional study.

Importantly, we did not observe the induction of Erg10, which is a hallmark of azole activity. Mycosidine itself decreases the amount of Erg10, thus the hybrid molecule exhibits an intermediate response. Somewhat similarly, **L-173** exhibits a less dramatic induction of Hxt3 than the azole compounds, while Mycosidine was previously observed to exhibit a concentration-dependent Hxt3 response. Mycosidine toxicity depended on Hxt3 [13].

While azoles do not affect the abundance of the histone Htb2 (suggesting no effect of the cell cycle), both mycosidine and **L-173** decrease the abundance, which involves appearance of a low-fluorescence fraction (Figure 7B). While our quantifications remove dead cells (stained with propidium iodide) from the analysis, some reduction of protein levels in cells that have not been stained yet is also possible. Since both **L-173** and mycosidine exhibit noticeable fungicidal effects (Figure 7C), this is the most likely explanation for a fraction of cells with lowered Htb2 fluorescence. Lastly, **L-173** exhibits a lack of Hsp104 and Bmh1 induction, which were not observed either for the azoles or mycosidine.

## 6. Materials and Methods

### 6.1. Chemistry

All the reagents were obtained commercially and used without further purification. Oxirane **1** and (4-oxo-2-thioxo-3-thiazolidinyl)acetic acid (**23**) were purchased from Sigma-Aldrich. The purity of the compounds was checked by thin-layer chromatography using silica-gel 60 F254-coated Al plates (Merck, Rahway, NJ, USA) and spots were observed under UV light. ^1^H NMR and ^13^C NMR spectra were recorded on a Bruker Avance spectrometer (600 and 150 MHz, respectively, Bruker, Billerica, MA, USA) and Varian VXR-400 spectrometer (Varian, Berkeley, CA, USA) at 400 MHz and 101 MHz, respectively, at 298 K in CDCl_3_ or DMSO-*d*_6_ at a concentration of samples of 5–15 mmol, with TMS as an internal reference for ^1^H and ^13^C NMR spectra. The signal assignments of compounds **L-310** and **31a (L-173)** were performed using 2D spectra (DQF-COSY, ^13^C–^1^H HSQC, and ^13^C–^1^H HMBC); the chemical shifts are expressed in ppm (δ scale) using DMSO-*d_6_* or CDCl_3_ as an internal standard; the coupling constants expressed in Hz. The mass-spectral measurements were carried out by the ESI method on micrOTOF-QII (Brucker Daltonics GmbH, Bremen, Germany). Analytical HPLC was performed on a Shimadzu LC-20AD system using Kromasil-100-5-C18 (Akzo-Nobel, Amsterdam, The Netherlands) column, 4.6 × 250 mm, temperature 20 °C, UV detection, mobile phase A—0.2% HCOONH_4_), mobile phase B-MeCN, (pH 7.4), fl-1ml/min. LC/MS was performed on Agilent 1100 LC/MSD (Agilent Technologies, Santa Clara, CA, USA), MSD Ionization Source: APCI or ESI, detectors: ELSD, SEDEX 85, DAD (UV) 200–400 nm TWC (Total Wave Chromatogram).

**3-(2-(4-(2-(2,4-Difluorophenyl)-2-hydroxy-3-(1*H*-1,2,4-triazol-1-yl)propyl)piperazin-1-yl)-2-oxoethyl)thiazolidine-2,4-dione (24)** (Method A1). 2-(2,4-Dioxothiazolidin-3-yl)acetic acid (**11**) (175 mg, 1.0 mmol) was refluxed in 5 mL of SOCl_2_ for 2 h, then all volatiles evaporated in vacuo and the residue used without purification. 2-(2,4-Difluorophenyl)-4-(piperazin-1-yl)-3-(1*H*-1,2,4-triazol-1-yl)propan-2-ol hydrochloride (**4a**) (300 mg, 0.83 mmol) was suspended in 10 mL of CH_2_Cl_2_, then triethylamine (253 mg, 2.50 mmol, 3 equiv) was added. The reaction mixture was cooled in an ice bath and a solution of freshly prepared (see above) 2-(2,4-dioxothiazolidin-3-yl)acetyl chloride (194 mg, 1.0 mmol, 1.2 equiv) in CH_2_Cl_2_ was added dropwise. Upon completion of the acyl chloride addition, the pH of the mixture was adjusted to 8 with Et_3_N if needed. The reaction mixture was allowed to heat to room temperature and stirred overnight. Then it was washed with a saturated solution of citric acid until acidic pH, the organic layer was separated, dried over sodium sulfate and methylene chloride was distilled under vacuum. The resulting residue was redissolved in methylene chloride and the substance was purified by chromatography on silica gel (eluent—methanol:CH_2_Cl_2_ 1:33). Yield 52%, light brown foam.

(Method A2) In methylene chloride (3 mL), 2-(2,4-dioxothiazolidin-3-yl)acetic acid (**11**) (0.42 mmol) and 2-(2,4-difluorophenyl)-1-(piperazin-1-yl)-3-(1*H*-1,2,4-triazol-1-yl)propan-2-ol (**4a**) hydrochloride (181 mg, 0.50 mmol, 1.2 equiv) were suspended, then triethylamine (169 mg, 1.67 mmol, 4 equiv) was added. The resulting solution was stirred for 30 min, and TBTU (161 mg, 0.50 mmol, 1.2 equiv) was added. pH was then adjusted to 8 with Et_3_N and the reaction mixture stirred at RT for 16 h. The organic layer was washed with water, dried over Na_2_SO_4_ and evaporated on a rotary evaporator. The residue was purified by flash chromatography on silica gel (eluent—ethyl acetate:hexane 3:1) to provide **24**. Yield 32%, white foam.

^1^H NMR (400 MHz, DMSO-*d*_6_) δ 8.28 (s, 1H), 7.73 (s, 1H), 7.40 (td, *J* = 9.4, 9.0, 6.9, 1H), 7.13 (ddt, *J* = 11.5, 9.1, 2.4, 1H), 6.95 (td, *J* = 8.6, 2.5, 1H), 5.72 (s, 1H), 4.57 (d, *J* = 4.0, 2H), 4.33 (s, 1H), 4.26 (s, 1H), 3.98 (s, 1H), 3.46–3.42 (m, 4H + H_2_O), 2.86 (dd, *J* = 14.1, 7.2, 1H), 2.72–2.69 (dd, *J* = 13.9, 10.5, 1H), 2.38 (tp, *J* = 16.9, 5.3, 3H). ^13^C NMR (101 MHz, DMSO-*d*_6_) δ 172.23, 171.93, 163.37, 150.79, 145.25, 130.17, 111.09 (d, *J* = 20.6), 104.09 (t, *J* = 27.0), 75.04, 63.73, 55.94, 54.38, 54.09, 46.02, 44.46, 42.57, 42.11, 34.21, 21.44. LC/MS (ESI): *m*/*z* 481.1. Calculated for C_20_H_22_F_2_N_6_O_4_S [M + H]^+^ 481.1.

**3-(1-(4-(2-(2,4-Difluorophenyl)-2-hydroxy-3-(1*H*-1,2,4-triazol-1-yl)propyl)piperazin-1-yl)-1-oxopropan-2-yl)thiazolidin-2,4-dione (25)** was obtained as described above from **12** and **4a**. Yield 25%, crystalline yellow foam. ^1^H NMR (400 MHz, DMSO-*d*_6_) δ 8.26 (s, 1H), 7.72 (s, 1H), 7.38 (td, *J* = 8.9, 6.7, 1H), 7.12 (ddd, *J* = 11.9, 9.1, 2.6, 1H), 6.93 (td, *J* = 8.5, 2.6, 1H), 5.69 (s, 1H), 4.94 (qd, *J* = 7.1, 1.8, 1H), 4.55 (s, 2H), 4.21 (d, *J* = 2.7, 2H), 3.10 (s, 2H), 2.84 (d, *J* = 13.9, 1H), 2.67 (dd, *J* = 13.9, 2.1, 1H), 2.34 (s, 4H), 1.44–1.30 (m, 3H). ^13^C NMR (101 MHz, DMSO-*d*_6_) δ 171.95, 171.78, 166.34, 150.80, 145.24, 130.15, 111.09 (d, *J* = 20.2), 104.10 (t, *J* = 27.4), 75.10, 63.71, 55.94, 54.29, 49.97, 45.18, 42.41, 33.79, 14.79. LC/MS (ESI): *m*/*z* 495.2. Calculated for C_21_H_24_F_2_N_6_O_4_S [M + H]^+^ 495.2.

**3-(4-(4-(2-(2,4-Difluorophenyl)-2-hydroxy-3-(1*H*-1,2,4-triazol-1-yl)propyl)piperazin-1-yl)-4-oxobutyl)thiazolidine-2,4-dione (26)** was obtained as described above from **13** and **4a**. Yield 69%, yellow foam. ^1^H NMR (700 MHz, DMSO-*d*_6_) δ 8.30 (s, 1H), 7.75 (s, 1H), 7.43–7.39 (m, 1H), 7.18–7.13 (m, 1H), 6.97 (td, *J* = 8.5, 2.5, 1H), 5.71 (s, 1H), 4.58 (s, 2H), 4.36 (t, *J* = 5.1, 1H), 4.15–4.12 (m, 2H), 3.49 (t, *J* = 6.9, 2H), 3.47–3.42 (m, 3H), 3.34–3.29 (m, 2H), 3.24 (t, *J* = 4.7, 2H), 2.87 (dd, *J* = 13.9, 0.9, 1H), 2.69 (d, *J* = 13.9, 1H), 2.51 (dt, *J* = 3.6, 1.8, 1H), 2.43–2.38 (m, 1H), 2.39–2.33 (m, 2H), 2.25 (t, *J* = 7.2, 2H), 1.70 (*p*, *J* = 7.1, 2H). ^13^C NMR (176 MHz, DMSO-*d*_6_) δ 172.91, 172.55, 169.96, 162.13 (dd, *J* = 245.7, 12.6), 159.41 (dd, *J* = 246.9, 12.1), 150.92, 145.37, 130.36–130.13 (m), 126.68–126.39 (m), 111.20 (d, *J* = 20.3), 104.21 (t, *J* = 26.9), 75.13 (d, *J* = 5.3), 63.90, 56.50, 56.06, 56.05, 54.54 (d, *J* = 38.3), 45.22, 41.45 (d, *J* = 45.7), 34.36, 29.72, 22.99, 19.01. LC/MS (ESI): *m*/*z* 509.2. Calculated for C_22_H_26_F_2_N_6_O_4_S [M + H]^+^ 509.2.

**3-(5-(4-(2-(2,4-Difluorophenyl)-2-hydroxy-3-(1*H*-1,2,4-triazol-1-yl)propyl)piperazin-1-yl)-4-oxopentyl)thiazolidine-2,4-dione (27)** was obtained as described above from **14** and **4a**. Yield 65%, white foam. ^1^H NMR (700 MHz, DMSO-*d*_6_) δ = 8.31 (s, 1H), 7.75 (s, 1H), 7.44–7.37 (m, 1H), 7.16 (t, *J* = 9.5, 1H), 7.00–6.94 (m, 1H), 4.58 (s, 2H), 4.19 (d, *J* = 7.1, 4H), 3.48 (dt, *J* = 12.6, 6.9, 5H), 2.85 (d, *J* = 14.0, 1H), 2.68 (d, *J* = 13.9, 1H), 2.30 (s, 2H), 1.90–1.72 (m, 3H). ^13^C NMR (176 MHz, DMSO-*d*_6_) δ 172.89, 172.87, 172.53, 172.51, 170.56, 162.15 (dd, *J* = 245.5, 12.7), 159.40 (dd, *J* = 247.0, 12.0), 150.93, 145.40, 130.26, 126.47, 111.23 (d, *J* = 20.1), 75.09, 63.84, 56.01, 54.80, 54.45, 41.40, 41.27, 40.30, 40.18, 40.07, 34.33, 34.32, 33.49, 31.98, 27.14, 26.95, 22.33, 22.06. LC/MS (ESI): *m*/*z* 523.2. Calculated for C_23_H_28_F_2_N_6_O_4_S [M + H]^+^ 523.2.

**3-(1-(2,4-Dichlorophenyl)-2-(4-(2-(2,4-difluorophenyl)-2-hydroxy-3-(1*H*-1,2,4-triazol-1-yl)propyl)piperazin-1-yl)-2-oxoethyl)thiazolidine-2,4-dione (28)** was obtained from **15** and **4a**. Yield 34%, yellow foam. ^1^H NMR (CDCl_3_) δ = 8.07 (s, 1H), 7.80 (s, 1H), 7.58–7.39 (m, 2H), 7.31 (m, 1H), 6.84 (m, 2H), 6.38–6.25 (m, 1H), 6.00–5.83 (m, 1H), 4.88 (br, 1H), 4.57 (m, 2H), 4.05–3.88 (m, 2H), 3.80–2.24 (m, 8H). LC/MS (ESI): *m*/*z* 625.1. Calculated for C_26_H_24_Cl_2_F_2_N_6_O_4_S [M + H]^+^ 625.1.

**3-(2-(4-(2-(2,4-Difluorophenyl)-2-hydroxy-3-(1*H*-1,2,4-triazol-1-yl)propyl)piperazin-1-yl)-2-oxoethyl)-2-thioxothiazolidin-4-one (29)** was obtained as described above from **23** and **4a**. Yield 20%, brown foam. ^1^H NMR (700 MHz, DMSO-*d*_6_) δ = 8.30 (s, 1H), 7.75 (s, 1H), 7.41 (s, 1H), 7.16 (s, 1H), 6.97 (s, 1H), 5.76 (s, 1H), 4.78–4.28 (m, 5H), 3.31 (s, 2H), 2.89 (s, 1H), 2.69 (s, 2H), 2.56–2.31 (m, 4H). ^13^C NMR (176 MHz, DMSO-*d*_6_) δ 203.42, 174.39, 162.87, 162.17 (d, *J* = 246.6), 159.41 (dd, *J* = 246.9, 11.3), 150.92, 145.38, 130.26, 126.49 (d, *J* = 12.0), 111.22 (d, *J* = 20.2), 104.39, 104.23 (t, *J* = 26.9), 104.08, 75.19, 63.82, 56.02, 54.59, 54.28, 45.39, 44.75, 42.28, 38.71, 36.31. LC/MS (ESI): *m*/*z* 497.1. Calculated for C_20_H_22_F_2_N_6_O_3_S_2_ [M + H]^+^ 497.1.

**3-(2-(4-(2-(2,4-Dichlorophenyl)-2-hydroxy-3-(1*H*-1,2,4-triazol-1-yl)propyl)piperazin-1-yl)-2-oxoethyl)-2-thioxothiazolidin-4-one (30)** was obtained as described above from **23** and **4b**. Yield 28%, light brown foam. ^1^H NMR (700 MHz, DMSO-*d*_6_) δ = 8.32 (s, 2H), 7.86 (s, 1H), 7.75 (s, 2H), 7.68 (dd, *J* = 15.4, 7.4, 1H), 7.66–7.61 (m, 1H), 7.60–7.49 (m, 5H), 7.33 (s, 3H), 5.85 (s, 2H), 4.89 (d, *J* = 14.3, 3H), 4.29 (d, *J* = 4.9, 2H), 3.82–3.68 (m, 5H), 2.96–2.70 (m, 3H), 2.51 (s, 4H), 1.59 (s, 3H), 1.47 (d, *J* = 3.4, 6H). ^13^C NMR (176 MHz, DMSO-*d*_6_) δ 193.60, 181.20, 167.59, 166.95, 150.99, 145.53, 136.26, 133.03, 132.80, 132.62, 132.22, 131.99, 131.21, 130.24, 130.09, 127.30, 123.29, 54.88, 54.33, 54.12, 48.92, 46.93, 45.65, 44.82, 25.69, 24.41. LC/MS (ESI): *m*/*z* 529.1. Calculated for C_20_H_22_Cl_2_N_6_O_3_S_2_ [M + H]^+^ 529.1.

***(Z)*-5-(4-Chlorobenzylidene)-3-(2-(4-(2-(2,4-difluorophenyl)-2-hydroxy-3-(1*H*-1,2,4-triazol-1-yl)propyl) piperazin-1-yl)-2-oxoethyl)thiazolidine-2,4-dione (31a, L-173)^5^** was obtained as described above from **22a** and **4a**. Yield 67%, white crystals.

(Method B) To a solution of *(Z)*-5-(4-chlorobenzylidene)-3-(2-oxo-2-(piperazin-1-yl)ethyl)thiazolidin-2,4-dione (3.65 g, 10 mmol) in a mixture of PhMe:NMP—20:1 (50 mL), 1-((2-(2,4-difluorophenyl)oxiran-2-yl)methyl)-1H-1,2,4-triazole (2.37 g, 10 mmol) was added, followed by Et_3_N (5 mL, 38 mmol). The reaction mixture was stirred at 100 °C for 12 h, then cooled to room temperature and evaporated in vacuo. The residue was purified by flash chromatography using an EtOAc:MeOH:Et_3_N (25:1:0.1) mixture as an eluent. Yield 3.0 g (51%), crystalline powder. M.p. 150.3 °C. ^1^H NMR (700 MHz, DMSO-*d*_6_) δ 8.29 (s, 1H), 7.94 (s, 1H), 7.74 (s, 1H), 7.65 (d, *J* = 8.0, 2H), 7.57 (d, *J* = 8.0, 2H), 7.40 (td, *J* = 8.9, 6.8, 1H), 7.16 (ddd, *J* = 11.8, 9.1, 2.6, 1H), 6.96 (td, *J* = 8.4, 2.6, 1H), 5.73 (s, 1H), 4.58 (s, 2H), 4.54 (s, 2H), 3.4–3.25 (m, 4H), 2.88 (dd, *J* = 13.8, 1.5, 1H), 2.71 (d, *J* = 13.8, 1H), 2.49–2.40 (m, 3H), 2.40–2.31 (m, 4H). ^13^C NMR: (151 MHz, DMSO-*d*_6_) δ 166.78, 165.15, 162.89, 161.68 (dd, *J* = 245.9, 12.4), 158.94 (dd, *J* = 247.0, 12.0), 150.46, 144.90, 135.41, 132.17, 131.79, 131.71, 130.02–129.66 (m), 129.45, 126.03 (dd, *J* = 13.1, 3.6), 121.70, 110.98–110.49 (m), 103.76 (dd, *J* = 28.1, 25.7), 74.73 (d, *J* = 5.5), 63.37 (d, *J* = 3.6), 55.57 (d, *J* = 4.9), 54.04, 53.74, 44.18, 42.66, 41.83. HRMS (ESI): Calcd for C_27_H_25_ClF_2_N_6_O_4_S [M + H]^+^ 603.1387. Found: *m*/*z* 603.1485.

**(*Z*)-3-(2-(4-(2-(2,4-Difluorophenyl)-2-hydroxy-3-(1*H*-1,2,4-triazol-1-yl)propyl)piperazin-1-yl)-2-oxoethyl)-5-(4-fluorobenzylidene)thiazolidine-2,4-dione (31b)** was obtained from **22b** and **4a**. Yield 72%, white crystals. ^1^H NMR (400 MHz, DMSO-*d*_6_) δ 8.29 (s, 1H), 7.94 (s, 1H), 7.75 (s, 1H), 7.69 (dd, *J* = 8.7, 5.4, 2H), 7.45–7.30 (m, 3H), 7.14 (ddd, *J* = 11.8, 9.1, 2.6, 1H), 6.95 (td, *J* = 8.5, 2.6, 1H), 5.76 (s, 1H), 4.58 (s, 2H), 4.54 (s, 2H), 3.45–3.35 (m, 3H), 3.34–3.23 (m, 3H), 2.88 (d, *J* = 13.9, 1H), 2.70 (d, *J* = 13.8, 1H), 2.56–2.33 (m, 5H). ^13^C NMR (101 MHz, DMSO-*d*_6_) δ 167.37, 165.67, 163.49 (d, *J* = 251.3), 163.34, 162.11 (dd, *J* = 246.0, 12.7), 159.37 (dd, *J* = 246.7, 11.9), 150.92, 145.37, 133.17, 133.09, 132.85, 130.23 (t), 129.92 (d, *J* = 3.2), 126.48 (d, *J* = 13.0), 121.07 (d, *J* = 2.4), 117.00 (d, *J* = 22.0), 111.20 (d, *J* = 20.7), 104.21 (t, *J* = 26.8), 75.16 (d, *J* = 5.5), 62.48, 56.00 (d), 44.60, 43.07, 42.25. LC/MS (ESI): *m*/*z* 587.2. Calcd for C_27_H_25_F_3_N_6_O_4_S [M + H]^+^ 587.2.

**(*Z*)-3-(2-(4-(2-(2,4-Difluorophenyl)-2-hydroxy-3-(1*H*-1,2,4-triazol-1-yl)propyl)piperazin-1-yl)-2-oxoethyl)-5-(4-methoxybenzylidene)thiazolidine-2,4-dione (31c)** was obtained from **22c** and **4a**; Yield 61%, bright yellow foam. ^1^H NMR (400 MHz, DMSO-*d*_6_) δ 8.30 (s, 1H), 7.88 (s, 1H), 7.75 (s, 1H), 7.64–7.55 (m, 3H), 7.41 (td, *J* = 9.0, 6.8, 1H), 7.23–7.05 (m, 4H), 6.96 (td, *J* = 8.5, 2.7, 1H), 5.77 (s, 1H), 4.58 (s, 2H), 4.51 (d, *J* = 9.5, 3H), 3.82 (d, *J* = 2.4, 5H), 3.70 (s, 1H), 3.47–3.22 (m, 6H), 2.89 (d, *J* = 13.9, 1H), 2.71 (d, *J* = 13.8, 1H), 2.57–2.30 (m, 7H). ^13^C NMR (101 MHz, DMSO-*d*_6_) δ 167.25, 167.12, 165.38, 162.98, 161.26, 150.46, 144.91, 134.15, 133.44, 132.47, 132.33, 129.98–129.57 (m), 126.35–125.65 (m), 125.29, 117.64, 114.99, 110.75 (d, *J* = 21.1), 103.76 (t, *J* = 27.0), 74.71 (d, *J* = 5.3), 63.34, 55.51, 54.03, 53.73, 52.65, 44.13, 42.52, 42.01, 41.77. LC/MS (ESI): m/z 599.2. Calcd for C_28_H_28_F_2_N_6_O_5_S [M + H]^+^ 599.2.

**(*Z*)-5-(4-Ethylbenzylidene)-3-(2-(4-(2-(2,4-difluorophenyl)-2-hydroxy-3-(1*H*-1,2,4-triazol-1-yl)propyl)piperazin-1-yl)-2-oxoethyl)thiazolidine-2,4-dione (31d)** was obtained from **22d** and **4a**. Yield 72%, white crystals. ^1^H NMR (400 MHz, DMSO-*d*_6_) δ 8.30 (s, 1H), 7.90 (s, 1H), 7.75 (s, 1H), 7.54 (d, *J* = 8.1, 2H), 7.40 (s, 1H), 7.38–7.33 (m, 2H), 7.15 (ddd, *J* = 11.8, 9.1, 2.6, 1H), 6.96 (td, *J* = 8.5, 2.6, 1H), 5.76 (s, 1H), 4.58 (s, 2H), 4.53 (s, 2H), 3.36 (d, *J* = 4.9, 3H), 3.34–3.22 (m, 1H), 2.95–2.83 (m, 1H), 2.71 (d, *J* = 13.8, 1H), 2.64 (q, *J* = 7.6, 2H), 2.42 (qd, *J* = 12.8, 12.3, 6.3, 2H), 1.17 (t, *J* = 7.6, 3H). ^13^C NMR (101 MHz, DMSO-*d*_6_) δ 167.50, 165.74, 163.35, 160.86 (d, *J* = 12.3), 160.57 (d, *J* = 12.2), 158.12 (d, *J* = 12.3), 150.88, 147.65, 145.32, 133.94, 130.76 (d, *J* = 4.2), 130.17 (d, *J* = 8.6), 129.26, 126.45 (d, *J* = 11.7), 120.17, 111.16 (d, *J* = 20.4), 104.19 (t, *J* = 26.8), 75.14 (d, *J* = 5.4), 63.77, 55.96, 54.46, 54.16, 44.57, 42.99, 42.22, 28.55, 15.59. HRMS (ESI): Calcd for C_29_H_30_F_2_N_6_O_4_S [M + H]^+^ 597.2090. Found: *m*/*z* 597.2103.

**(*Z*)-5-(4-Isopropylbenzylidene)-3-(2-(4-(2-(2,4-difluorophenyl)-2-hydroxy-3-(1*H*-1,2,4-triazol-1-yl)propyl)piperazin-1-yl)-2-oxoethyl)thiazolidine-2,4-dione (31e)** was obtained from **22e** and **4a**. Yield 65%, white crystals. ^1^H NMR (400 MHz, DMSO-*d*_6_) δ 8.30 (s, 1H), 7.90 (s, 1H), 7.75 (s, 1H), 7.62–7.52 (m, 2H), 7.47–7.38 (m, 3H), 7.16 (ddd, *J* = 11.8, 9.1, 2.6, 1H), 6.96 (td, *J* = 8.5, 2.6, 1H), 4.58 (s, 2H), 4.54 (s, 2H), 3.37 (t, *J* = 5.0, 2H), 3.35–3.21 (m, 2H), 3.00–2.84 (m, 2H), 2.71 (d, *J* = 13.7, 1H), 2.41 (tt, *J* = 12.9, 6.7, 4H), 1.20 (d, *J* = 6.9, 6H). ^13^C NMR (101 MHz, DMSO-*d*_6_) δ 167.51, 165.74, 163.35, 160.85 (d, *J* = 12.5), 160.63, 158.05, 152.13, 150.88, 145.32, 133.90, 130.86 (d, *J* = 6.7), 130.16 (d, *J* = 9.4), 127.84, 126.44 (d, *J* = 13.7), 120.21, 111.17 (d, *J* = 20.1), 104.60–103.83 (m), 85.45, 75.12 (d, *J* = 5.5), 63.76, 55.95, 54.45, 54.15, 44.54, 42.99, 42.19, 33.86, 23.90. HRMS (ESI): Calcd for C_30_H_32_F_2_N_6_O_4_S [M + H]^+^ 611.2247. Found: *m*/*z* 612.2445.

**(*Z*)-5-(4-*tert*-Butylbenzylidene)-3-(2-(4-(2-(2,4-difluorophenyl)-2-hydroxy-3-(1*H*-1,2,4-triazol-1-yl)propyl)piperazin-1-yl)-2-oxoethyl)thiazolidine-2,4-dione (31f)** was obtained from **22f** and **4a**. Yield 63%, white crystals. ^1^H NMR (400 MHz, DMSO-*d*_6_) δ 8.30 (s, 1H), 7.91 (s, 1H), 7.75 (s, 1H), 7.57 (d, *J* = 3.0, 4H), 7.40 (td, *J* = 9.0, 6.8, 1H), 7.16 (ddd, *J* = 11.8, 9.1, 2.6, 1H), 6.96 (td, *J* = 8.5, 2.6, 1H), 5.78 (s, 1H), 4.58 (s, 2H), 4.54 (s, 2H), 3.37 (d, *J* = 5.2, 2H), 3.34–3.21 (m, 3H), 2.89 (d, *J* = 13.9, 1H), 2.71 (d, *J* = 13.8, 1H), 2.42 (tt, *J* = 12.9, 6.7, 4H), 1.28 (s, 9H). ^13^C NMR (101 MHz, DMSO-*d*_6_) δ 167.51, 165.74, 163.35, 160.85 (d, *J* = 12.5), 160.51, 158.12 (d, *J* = 12.0), 154.30, 150.88, 145.33, 133.78, 130.57, 130.22, 126.71, 126.59–126.28 (m), 120.32, 111.17 (d, *J* = 20.6), 104.19 (t, *J* = 26.9), 75.14 (d, *J* = 5.4), 63.78, 55.95, 54.46, 54.15, 44.57, 43.00, 42.21, 35.20, 31.19. HRMS (ESI):Calcd for C_31_H_34_F_2_N_6_O_4_S [M + H]^+^ 625.2403. Found: *m*/*z* 625.2295.

**(*Z*)-5-(2,4-Dichlorobenzylidene)-3-(2-(4-(2-(2,4-difluorophenyl)-2-hydroxy-3-(1*H*-1,2,4-triazol-1-yl)propyl)piperazin-1-yl)-2-oxoethyl)thiazolidine-2,4-dione (31g)** was obtained from **22g** and **4a**. Yield 58%, white crystals. ^1^H NMR (400 MHz, DMSO-*d*_6_) δ 8.28 (s, *J* = 4.1, 1H), 7.94 (d, *J* = 8.3, 2H), 7.83 (s, 1H), 7.74 (s, 1H), 7.60 (s, 2H), 7.40 (dd, *J* = 15.9, 8.5, 1H), 7.14 (t, *J* = 9.7, 1H), 6.95 (t, *J* = 7.4, 1H), 5.73 (s, 1H), 4.57 (s, *J* = 10.3, 2H), 4.55 (s, 2H), 3.37 (s, 2H), 2.87 (s, 5H), 2.71 (s, 5H), 2.46–2.32 (m, 3H). ^13^C NMR (101 MHz, DMSO-*d*_6_) δ 167.00, 165.22, 163.23, 162.72, 150.90, 145.34, 136.28, 135.86, 130.60, 130.42, 130.34, 130.22, 128.84, 127.91, 125.76, 111.28, 111.07, 104.47, 104.19, 103.95, 75.19, 75.13, 63.77, 59.91, 56.02, 55.96, 54.48, 54.17, 49.97, 44.62, 43.20, 42.43, 42.28, 36.21, 31.20. LC/MS (ESI): *m*/*z* 637.1. Calculated for C_27_H_24_Cl_2_F_2_N_6_O_4_S [M + H]^+^ 637.1.

**(*Z*)-5-(4-Chlorobenzylidene)-3-(1-(4-(2-(2,4-difluorophenyl)-2-hydroxy-3-(1*H*-1,2,4-triazol-1-yl)propyl)piperazine-1-yl)-1-oxopropan-2-yl)thiazolidine-2,4-dione (32a)** was obtained from **23a** and **4a**. Yield 72%, white crystals. ^1^H NMR (700 MHz, DMSO-*d*_6_) δ 8.26 (s, 1H), 7.92–7.90 (m, 1H), 7.72 (s, 1H), 7.64 (d, *J* = 8.4, 2H), 7.60 (d, *J* = 8.3, 2H), 7.37 (q, *J* = 8.3, 1H), 7.16–7.09 (m, 1H), 6.93 (td, *J* = 8.4, 2.6, 1H), 5.69 (d, *J* = 2.5, 1H), 5.19 (qd, *J* = 7.2, 2.9, 1H), 4.55 (s, 2H), 3.35–3.30 (m, 3H), 3.20 (s, 2H), 2.83 (dd, *J* = 14.0, 4.9, 1H), 2.67 (dd, *J* = 14.0, 4.4, 1H), 2.45–2.40 (m, 3H), 2.33 (s, 2H), 2.29 (s, 1H), 1.49 (dd, *J* = 7.2, 4.0, 3H). ^13^C NMR (176 MHz, DMSO-*d*_6_) δ 166.82, 166.42, 165.46, 162.02 (dd, *J* = 245.7, 12.5), 159.29 (dd, *J* = 247.0, 12.0), 150.84, 145.27, 135.75, 132.51, 132.23, 132.18, 130.16, 129.87, 126.36 (d, *J* = 13.1), 121.62, 111.11 (d, *J* = 20.5), 104.13 (t, *J* = 26.9), 75.15, 63.71, 55.92, 54.45, 54.40, 54.27, 50.85 (d, *J* = 7.7), 45.34, 42.52, 14.90. LC/MS (ESI): *m*/*z* 617.2. Calculated for C_28_H_27_ClF_2_N_6_O_4_S [M + H]^+^ 617.2

The compound was also synthesized by Method B (see **31a**) with a 46% yield.

**(*Z*)-5-(4-Chlorobenzylidene)-3-(4-(4-(2-(2,4-difluorophenyl)-2-hydroxy-3-(1*H*-1,2,4-triazol-1-yl)propyl)piperazin-1-yl)-4-oxobutyl)thiazolidine-2,4-dione (33a)** was obtained from **24a** and **4a**. Yield 41%, white crystals. ^1^H NMR (700 MHz, DMSO-*d*_6_) δ = 8.30 (s, 1H), 7.89 (s, 1H), 7.75 (s, 1H), 7.63 (d, *J* = 8.6, 2H), 7.60 (d, *J* = 8.6, 1H), 7.40 (d, *J* = 7.0, 1H), 7.16–7.13 (m, 1H), 6.96 (td, *J* = 8.5, 2.4, 1H), 5.84–5.60 (m, 1H), 4.58 (s, 2H), 3.66 (br. s, 2H), 2.85 (d, *J* = 14.0, 1H), 2.68 (d, *J* = 13.9, 1H), 2.30 (s, 2H), 1.90–1.72 (m, 3H). ^13^C NMR (176 MHz, DMSO-*d*_6_) δ 169.93, 167.63, 166.12, 162.12 (dd, *J* = 245.7, 12.4), 159.39 (dd, *J* = 246.9, 12.2), 150.91, 145.37, 135.61, 132.39, 132.15, 131.62, 130.25, 129.91, 126.53 (d, *J* = 13.3), 122.83, 111.19 (d, *J* = 20.5), 104.20, 75.13 (d, *J* = 5.3), 63.90, 56.02, 54.62, 54.39, 45.22, 41.92, 41.60, 29.74, 22.96. LC/MS (ESI): *m*/*z* 631.2. Calculated for C_29_H_29_ClF_2_N_6_O_4_S [M + H]^+^ 631.2.

The compound was also synthesized by Method B (see **31a**) with a 32% yield.

**(*Z*)-3-(4-(4-(2-(2,4-Difluorophenyl)-2-hydroxy-3-(1*H*-1,2,4-triazol-1-yl)propyl)piperazin-1-yl)-4-oxobutyl)-5-(4-fluorobenzylidene)thiazolidine-2,4-dione (33b)** was obtained from **24b** and **4a**. Yield 37%, yellow crystals. ^1^H NMR (700 MHz, DMSO-*d*_6_) δ 8.31 (s, 1H), 7.90 (s, 1H), 7.75 (s, 1H), 7.72–7.65 (m, 3H), 7.44–7.33 (m, 4H), 7.15 (t, *J* = 9.5, 1H), 6.96 (t, *J* = 7.2, 1H), 5.91–5.55 (m, 1H), 4.58 (s, 2H), 3.66 (t, *J* = 6.8, 2H), 3.26 (br. s, 6H), 2.47–2.21 (m, *J* = 7.0, 8H), 2.30 (t, *J* = 7.0, 2H), 1.82–1.79 (m, 2H). ^13^C NMR (176 MHz, DMSO-*d*_6_) δ 169.96, 167.77, 166.19, 163.40 (d, *J* = 251.0), 162.16 (dd, *J* = 245.7, 12.2), 159.39 (dd, *J* = 246.9, 12.1), 150.94, 145.41, 133.04, 132.99, 131.91, 130.25, 130.16, 126.43, 121.75, 117.02 (d, *J* = 22.0), 111.24 (d, *J* = 20.4), 104.24 (t, *J* = 26.8), 75.04, 56.01, 54.35, 41.87, 29.73, 22.97. LC/MS (ESI): *m*/*z* 615.2. Calculated for C_29_H_29_F_3_N_6_O_4_S [M + H]^+^ 615.2.

**(*Z*)-5-(2,4-Dichlorobenzylidene)-3-(4-(4-(2-(2,4-difluorophenyl)-2-hydroxy-3-(1*H*-1,2,4-triazol-1-yl)propyl)piperazin-1-yl)-4-oxobutyl)thiazolidin-2,4-dione (33g)** was obtained from **13g** and **4a**. Yield 45%, yellow crystals. ^1^H NMR (700 MHz, DMSO-*d*_6_) δ 8.30 (s, 1H), 7.93 (s, 1H), 7.84 (d, *J* = 2.0, 1H), 7.75 (s, 1H), 7.61 (d, *J* = 1.9, 1H), 7.57 (d, *J* = 8.5, 1H), 7.41 (d, *J* = 7.0, 1H), 7.18–7.11 (m, *J* = 9.3, 1H), 6.97–6.95 (m, 1H), 5.72 (s, 1H), 4.58 (s, 2H), 3.67 (t, *J* = 6.8, 2H), 3.25 (s, 3H), 2.85 (d, *J* = 13.9, 1H), 2.68 (d, *J* = 13.9, 1H), 2.45–2.24 (m, 7H), 1.86–1.76 (m, 2H). ^13^C NMR (176 MHz, DMSO-*d*_6_) δ 169.91, 167.41, 165.79, 162.12 (dd, *J* = 245.8, 12.4), 159.39 (dd, *J* = 246.9, 12.1), 150.92, 145.37, 136.07, 135.85, 130.50, 130.48, 130.45, 130.26 (t), 128.86, 126.51 (d, *J* = 2.0), 111.19 (d, *J* = 20.1), 104.20, 75.13 (d, *J* = 5.1), 63.90, 56.04, 56.02, 54.62, 54.41, 45.22, 42.09, 41.61, 29.77, 25.80, 22.88. LC/MS (ESI): *m*/*z* 665.1. Calculated for C_29_H_28_Cl_2_F_2_N_6_O_4_S [M + H]^+^ 665.1.

**(*Z*)-3-(2-(4-(2-(2,4-Difluorophenyl)-2-hydroxy-3-(1*H*-1,2,4-triazol-1-yl)propyl)piperazin-1-yl)-2-oxoethyl)-5-(4-chlorobenzylidene)-2-thio-1,3-thiazolidin-4-one (29a)** (Method K): To a solution of **29** (0.33 mmol) in 3 mL of ethanol, 4-chlorobenzaldehyde (0.36 mmol, 1.1 equiv) was added, followed by piperidine (6 mg, 0.07 mmol, 0.2 equiv.). The mixture refluxed for 16 h, then was cooled and evaporated on a rotary evaporator. The product was isolated by flash chromatography on silica gel (elution system — 15% methanol in CH_2_Cl_2_). Yield 60%, yellow crystals. ^1^H NMR (700 MHz, DMSO-*d*_6_) δ = 8.36 (s, 1H), 7.89 (s, 1H), 7.81 (s, 1H), 7.72 (d, *J* = 8.2, 2H), 7.67 (d, *J* = 8.3, 2H), 7.47 (q, *J* = 8.4, 1H), 7.22 (ddd, *J* = 11.7, 8.9, 2.6, 1H), 7.03 (td, *J* = 8.5, 2.5, 1H), 5.82 (s, 1H), 4.95 (s, 2H), 4.65 (s, 1H), 3.53–3.47 (m, 3H), 3.38 (dt, *J* = 10.5, 5.2, 2H), 2.96 (d, *J* = 13.8, 1H), 2.54–2.40 (m, 2H). ^13^C NMR (176 MHz, DMSO-*d*_6_) δ 193.56, 166.97, 162.76, 162.16 (dd, *J* = 246.0, 12.7), 159.42 (dd, *J* = 247.0, 12.1), 150.95, 145.39, 136.27, 132.60, 132.20, 130.28 (t, *J* = 8.2), 130.07, 126.4 (d, *J* = 12.9), 123.26, 111.26 (d, *J* = 20.4), 104.25 (t, *J* = 27.0), 75.19 (d, *J* = 5.3), 63.82, 56.03 (d, *J* = 5.0), 54.61, 54.29, 45.67, 44.82, 42.35. HRMS (EI): Calcd for C_27_H_25_ClF_2_N_6_O_3_S2 [M + H]^+^ 619.1159. Found: 619.1137.

**(*Z*)-5-(4-Chlorobenzylidene)-3-(2-(4-(2-(2,4-dichlorophenyl)-2-hydroxy-3-(1*H*-1,2,4-triazol-1-yl)propyl)piperazin-1-yl)-2-oxoethyl)-2-thioxothiazolidin-4-one (30a)** was obtained as described above from **30** and 4-chlorobenzaldehyde. Yield 58%, bright yellow crystals. ^1^H NMR (700 MHz, DMSO-*d*_6_) δ 8.32 (s, 1H), 7.84 (s, 1H), 7.76 (s, 1H), 7.68 (d, *J* = 8.4, 2H), 7.62 (d, *J* = 8.3, 2H), 7.59–7.51 (m, 2H), 7.33 (d, *J* = 7.8, 1H), 5.86 (s, 1H), 4.91 (s, 1H), 4.89 (s, 2H), 4.67 (d, *J* = 14.0, 1H), 3.33–3.20 (m, 3H), 2.82 (d, *J* = 10.3, 1H), 2.47–2.24 (m, 3H). ^13^C NMR (176 MHz, DMSO-*d*_6_) δ 193.56, 166.95, 162.76, 150.98, 145.51, 136.26, 133.03, 132.79, 132.61, 132.20, 132.02, 131.22, 130.22, 130.08, 127.28, 123.26, 76.18, 62.31, 54.89, 54.32, 54.04, 45.65, 44.73, 42.27. LC/MS (ESI): *m*/*z* 651.1. Calculated for C_27_H_25_Cl_3_N_6_O_3_S_2_ [M + H]^+^ 651.1.

**(*Z*)-5-(4-Methoxybenzylidene)-3-(2-(4-(2-(2,4-dichlorophenyl)-2-hydroxy-3-(1*H*-1,2,4-triazol-1-yl)propyl)piperazin-1-yl)-2-oxoethyl)-2-thio-1,3-thiazolidin-4-one (30c)** was obtained as described above from **30** and 4-methoxybenzaldehyde. Yield 39%, bright yellow crystals. ^1^H NMR (700 MHz, DMSO-*d*_6_) δ 8.32 (s, 1H), 7.80 (s, 1H), 7.76 (s, 1H), 7.62 (d, *J* = 8.7, 2H), 7.57 (d, *J* = 8.6, 1H), 7.54 (s, 1H), 7.33 (d, *J* = 8.1, 1H), 7.12 (d, *J* = 8.7, 2H), 5.87 (s, 1H), 4.90 (d, *J* = 14.3, 1H), 4.88 (s, 2H), 4.67 (d, *J* = 14.3, 1H), 3.84 (s, 3H), 3.34–3.18 (m, 1H), 2.82 (d, *J* = 11.1, 1H), 2.48–2.28 (m, 1H). ^13^C NMR (176 MHz, DMSO-*d*_6_) δ 193.68, 167.09, 162.87, 162.14, 150.97, 145.50, 134.20, 133.51, 133.03, 132.02, 131.22, 130.22, 127.28, 125.88, 119.17, 115.66, 76.17, 62.31, 56.07, 54.89, 54.32, 54.04, 45.57, 44.73, 42.25. LC/MS (ESI): *m*/*z* 647.1. Calculated for C_28_H_28_Cl_2_N_6_O_4_S_2_ [M + H]^+^ 647.1.

**(*Z*)-5-(3,5-Dichloro-2-hydroxybenzylidene)-3-(5-(4-(2-(2,4-difluorophenyl)-2-hydroxy-3-(1*H*-1,2,4-triazole-1-yl)propyl)piperazin-1-yl)-5-oxobutyl)thiazolidine-2,4-dione (33h)** was obtained as described above from **26** and 3,5-dichloro-2-hydroxybenzaldehyde. Yield 51%, white crystals. ^1^H NMR (700 MHz, DMSO-*d*_6_) δ 8.30 (s, 1H), 7.98 (s, 1H), 7.75 (d, *J* = 3.4, 1H), 7.65 (d, *J* = 2.2, 1H), 7.40 (q, *J* = 8.8, 1H), 7.27 (d, *J* = 2.2, 1H), 7.19–7.10 (m, 1H), 6.96 (dd, *J* = 8.1, 6.5, 1H), 4.58 (s, 2H), 3.66 (t, *J* = 6.8, 2H), 2.86 (s, 1H), 2.69 (d, *J* = 13.8, 1H), 2.46–2.32 (m, 4H), 1.85–1.73 (m, 2H). ^13^C NMR (176 MHz, DMSO-*d*_6_) δ 192.49, 172.92, 172.55, 169.98, 169.93, 167.60, 166.00, 162.13 (dd, *J* = 245.7, 12.3), 159.39 (dd, *J* = 247.1, 12.1), 152.23, 150.92, 145.37, 135.49, 131.35, 130.24 (t), 129.07, 126.95, 126.51 (d, *J* = 12.6), 124.90, 124.21, 123.92, 123.81, 111.20 (d, *J* = 20.4), 104.21 (t, *J* = 26.7), 75.08 (d, *J* = 4.9), 63.88, 56.04, 54.62, 54.40, 45.19, 41.90, 41.57, 41.32, 34.36, 29.75, 23.00. LC/MS (ESI): *m*/*z* 681.1. Calculated for C_29_H_28_Cl_2_F_2_N_6_O_5_S [M + H]^+^ 681.1.

**(*Z*)-5-(2-Chloro-6-(4-methylpiperazin-1-yl)benzylidene)-3-(4-(4-(2-(2,4-difluorophenyl)-2-hydroxy-3-(1*H*-1,2,4-triazol-1-yl)propyl)piperazin-1-yl)-4-oxobutyl)thiazolidine-2,4-dione (33i)** was obtained as described above from **26** and 2-chloro-6-(4-methylpiperazino)benzaldehyde. Yield 46%, white crystals. ^1^H NMR (700 MHz, DMSO-*d*_6_) δ 8.37 (s, 1H), 8.00 (s, 1H), 7.80 (s, 1H), 7.50–7.42 (m, *J* = 8.1, 2H), 7.27 (d, *J* = 7.9, 1H), 7.21 (d, *J* = 8.1, 1H), 7.20–7.17 (m, 1H), 7.01 (td, *J* = 8.5, 2.5, 1H), 5.82 (s, 1H), 4.63 (s, 2H), 3.70 (t, *J* = 6.8, 2H), 3.05 (br.s, 5H), 2.92 (d, *J* = 14.1, 1H), 2.73 (d, *J* = 13.8, 1H), 2.66 (s, 4H), 2.50–2.30 (m, 9H), 1.90–1.82 (m, 2H). ^13^C NMR (176 MHz, DMSO-*d*_6_) δ 169.88, 167.87, 166.04, 163.61, 162.11 (dd, *J* = 245.7, 12.2), 159.40 (dd, *J* = 246.9, 12.0), 152.53, 150.90, 145.39, 134.34, 132.08, 130.26, 129.93, 126.56 (d, *J* = 12.8), 126.04, 125.18, 124.00, 118.47, 111.19 (d, *J* = 20.2), 104.18 (t, *J* = 26.9), 75.15 (d, *J* = 5.4), 63.90, 56.02, 54.60, 54.39, 54.17, 51.24, 45.25, 44.05, 41.75, 41.62, 29.83, 23.03, 22.59. LC/MS (ESI): *m*/*z* 729.3. Calculated for C_34_H_39_ClF_2_N_8_O_4_S [M + H]^+^ 729.3.

**(*Z*)-5-(4-Chlorobenzylidene)-3-(5-(4-(2-(2,4-difluorophenyl)-2-hydroxy-3-(1*H*-1,2,4-triazol-1-yl)propyl)piperazin-1-yl)-5-oxopentyl)thiazolidin-2,4-dione (34a)** was obtained as described above from **27** and 4-chlorobenzaldehyde. Yield 44%, yellow crystals. ^1^H NMR (700 MHz, DMSO-*d*_6_) δ 8.30 (s, 1H), 7.90 (s, 1H), 7.75 (s, 1H), 7.63 (d, *J* = 8.4, 2H), 7.59 (d, *J* = 8.5, 2H), 7.45–7.35 (m, 1H), 7.13 (t, *J* = 9.3, 1H), 6.95 (t, *J* = 7.2, 1H), 4.57 (s, *J* = 7.5, 2H), 3.64 (t, *J* = 6.9, 2H), 2.85 (d, *J* = 14.0, 1H), 2.68 (d, *J* = 13.8, 1H), 2.44–2.30 (m, 4H), 2.30–2.19 (m, 2H), 1.70–1.52 (m, 2H), 1.52–1.36 (m, 2H). ^13^C NMR (176 MHz, DMSO-*d*_6_) δ 174.64, 170.52, 167.54, 166.03, 162.13 (dd, *J* = 245.8, 12.3), 159.38 (dd, *J* = 247.0, 12.1), 150.91, 145.37, 135.69, 132.32, 132.18, 131.98, 130.24 (t), 129.89, 126.50 (d, *J* = 12.9), 122.54, 111.19 (d, *J* = 20.3), 104.20 (t, *J* = 26.9), 75.11 (d, *J* = 5.1), 63.87, 56.02, 54.84, 54.48, 45.32, 41.93, 41.49, 32.00, 27.22, 22.4. LC/MS (ESI): *m*/*z* 645.2. Calculated for C_30_H_31_ClF_2_N_6_O_4_S [M + H]^+^ 645.2.

**(*Z*)-3-(5-(4-(2-(2,4-Difluorophenyl)-2-hydroxy-3-(1*H*-1,2,4-triazol-1-yl)propyl)piperazin-1-yl)-5-oxopentyl)-5-(4-fluorobenzylidene)thiazolidine-2,4-dione (34b)** was obtained from **27** and 4-fluorobenzaldehyde. Yield 48%, yellow crystals. ^1^H NMR (700 MHz, DMSO-*d*_6_) δ 8.31 (s, 1H), 7.94 (s, 1H), 7.75 (s, 1H), 7.73–7.67 (m, 2H), 7.48–7.33 (m, 3H), 7.18–7.13 (m, 1H), 7.00–6.91 (m, 1H), 4.57 (s, 2H), 3.64 (t, *J* = 6.9, 2H), 3.29 (s, 4H), 2.87 (d, *J* = 12.2, 1H), 2.76–2.61 (m, 1H), 2.37 (s, 4H), 2.30–2.16 (m, 3H), 1.69–1.34 (m, 6H). ^13^C NMR (176 MHz, DMSO-*d*_6_) δ 174.63, 170.52, 167.69, 166.10, 163.44 (d, *J* = 250.8), 162.14 (dd, *J* = 245.8, 12.3), 159.39 (dd, *J* = 247.0, 12.1), 150.93, 145.39, 133.07 (d, *J* = 8.8), 132.26, 130.25 (t), 130.12, 126.46 (d, *J* = 11.9), 121.50, 117.02 (d, *J* = 22.0), 116.08 (d, *J* = 22.3), 111.22 (d, *J* = 20.2), 104.23 (t, *J* = 26.9), 75.06, 63.83, 56.01, 54.81, 54.46, 45.25, 41.89, 41.40, 33.46, 31.99, 27.23, 27.15, 27.06, 22.41, 22.33, 22.11. LC/MS (ESI): *m*/*z* 629.2. Calculated for C_30_H_31_F_3_N_6_O_4_S [M + H]^+^ 629.2.

**(*Z*)-5-(2,4-Dichlorobenzylidene)-3-(5-(4-(2-(2,4-difluorophenyl)-2-hydroxy-3-(1*H*-1,2,4-triazol-1-yl)propyl)piperazin-1-yl)-5-oxopentyl)thiazolidin-2,4-dione (34g)** was obtained from **27** and 2,4-dichlorobenzaldehyde. Yield 44%, bright yellow crystals. ^1^H NMR (700 MHz, DMSO-*d*_6_) δ 8.30 (s, 1H), 7.94 (s, 1H), 7.82 (d, *J* = 1.6, 1H), 7.74 (s, 1H), 7.67–7.54 (m, 2H), 7.46–7.36 (m, 1H), 7.19–7.09 (m, 1H), 6.95 (t, *J* = 6.8, 1H), 4.58 (s, 2H), 3.64 (t, *J* = 6.9, 2H), 3.44–3.18 (m, 6H), 2.85 (d, *J* = 14.2, 1H), 2.68 (d, *J* = 13.7, 1H), 2.46–2.30 (m, 4H), 2.30–2.21 (m, 2H), 1.60–1.55 (m, 2H), 1.52–1.31 (m, 4H). ^13^C NMR (176 MHz, DMSO-*d*_6_) δ 174.62, 170.49, 167.34, 165.70, 162.12 (dd, *J* = 245.7, 12.4), 159.39 (dd, *J* = 246.9, 12.1), 150.91, 145.37, 136.14, 135.89, 130.53, 130.43, 130.25, 130.20, 128.83, 127.07, 126.52 (d, *J* = 12.9), 126.14, 111.19 (d, *J* = 20.3), 104.19 (t, *J* = 26.9), 75.12 (d, *J* = 4.7), 63.89, 56.02, 54.85, 54.49, 45.34, 42.09, 41.50, 32.00, 27.19, 22.41, 22.12. LC/MS (ESI): *m*/*z* 679.1. Calculated for C_30_H_30_Cl_2_F_2_N_6_O_4_S [M + H]^+^ 679.1.

**(*Z*)-5-(3,5-Dichloro-2-hydroxybenzylidene)-3-(5-(4-(2-(2,4-difluorophenyl)-2-hydroxy-3-(1*H*-1,2,4-triazol-1-yl)propyl)piperazin-1-yl)-5-oxopentyl)thiazolidine-2,4-dione (34h)** was obtained as described above from **27** and 3,5-dichloro-2-hydroxybenzaldehyde. Yield 41%, white crystals. ^1^H NMR (700 MHz, DMSO-*d*_6_) δ 8.30 (d, *J* = 2.8, 1H), 7.99 (s, 1H), 7.75 (d, *J* = 4.3, 1H), 7.65 (d, *J* = 2.4, 1H), 7.45–7.35 (m, 1H), 7.31–7.25 (m, 1H), 7.18–7.10 (m, 1H), 6.96 (dd, *J* = 5.9, 2.4, 1H), 4.58 (d, *J* = 5.0, 2H), 4.18 (d, *J* = 7.3, 1H), 2.87 (t, *J* = 13.8, 1H), 2.73–2.64 (m, 1H), 2.47–2.30 (m, 4H), 2.29–2.16 (m, 2H), 1.65–1.31 (m, 6H). ^13^C NMR (176 MHz, DMSO-*d*_6_) δ 174.65, 172.85, 172.50, 170.54, 167.54, 165.94, 162.13 (dd, *J* = 245.7, 12.4), 159.39 (dd, *J* = 247.5, 12.0), 152.21, 150.92, 145.37, 131.40, 130.23, 127.30, 127.04, 126.53, 126.49 (d, *J* = 13.0), 124.85, 124.02, 123.94, 123.81, 111.21 (d, *J* = 20.2), 104.29 (d, *J* = 26.3), 75.09 (d, *J* = 4.8), 63.86, 56.03, 54.83, 54.48, 45.30, 41.93, 41.47, 41.40, 41.27, 34.31, 33.49, 33.46, 31.99, 27.22, 27.15, 27.05, 26.95, 22.41, 22.34, 22.11, 22.07. LC/MS (ESI): *m*/*z* 695.1. Calculated for C_30_H_30_Cl_2_F_2_N_6_O_5_S [M + H]^+^ 695.1.

**(*Z*)-5-(2-Chloro-6-(4-methylpiperazin-1-yl)benzylidene)-3-(5-(4-(2-(2,4-difluorophenyl)-2-hydroxy-3-(1*H*-1,2,4-triazol-1-yl)propyl)piperazin-1-yl)-5-oxopentyl)thiazolidin-2,4-dione (34i)** was obtained as described above from **27** and 2-chloro-6-(4-methylpiperazin-1-yl)benzaldehyde. Yield 38%, white crystals. ^1^H NMR (700 MHz, DMSO-*d*_6_) δ = 8.30 (s, 1H), 7.98 (s, 1H), 7.75 (s, *J* = 3.6, 1H), 7.40 (t, *J* = 8.0, 2H), 7.20 (d, *J* = 7.9, 1H), 7.14 (d, *J* = 8.3, 2H), 7.00–6.91 (m, 1H), 5.73 (s, 1H), 4.57 (s, 2H), 3.64 (t, *J* = 6.8, 2H), 3.59–3.10 (m, 10H), 2.94 (s, 3H), 2.85 (d, *J* = 13.7, 2H), 2.67 (d, *J* = 13.9, 1H), 2.44 (s, 3H), 2.42–2.29 (m, 5H), 2.27 (t, *J* = 7.2, 2H), 2.20 (s, 4H), 1.63–1.53 (m, 2H), 1.46–1.38 (m, 2H). ^13^C NMR (176 MHz, DMSO-*d*_6_) δ 170.48, 167.77, 165.98, 163.51, 162.12 (dd, *J* = 245.6, 12.3), 159.39 (dd, *J* = 246.8, 12.3), 152.79, 150.91, 145.37, 134.35, 132.11, 130.41, 130.24, 126.54 (d, *J* = 12.6), 125.62, 125.05, 123.79, 118.42, 111.19 (d, *J* = 20.3), 104.20 (t, *J* = 26.8), 75.14 (d, *J* = 5.2), 63.90, 56.04, 55.31, 54.86, 54.55, 54.49, 51.82, 45.96, 45.34, 41.69, 41.51, 31.98, 27.31, 22.38. LC/MS (ESI): *m*/*z* 743.3. Calculated for C_35_H_41_ClF_2_N_8_O_4_S [M + H]^+^ 743.3.

**(*Z*)-3-(1-(2,4-Dichlorophenyl)-2-(4-(2-(2,4-difluorophenyl)-2-hydroxy-3-(1*H*-1,2,4-triazol-1-yl)propyl)piperazin-1-yl)-2-oxoethyl)-5-(4-fluorobenzylidene)thiazolidine-2,4-dione (35b)** was obtained from **30** and 4-fluorobenzaldehyde. Yield 33%, white crystals. ^1^H NMR (700 MHz, DMSO-*d*_6_) δ 8.27 (d, *J* = 1.1, 1H), 7.95 (s, 1H), 7.74 (d, *J* = 1.2, 1H), 7.72–7.68 (m, 3H), 7.52–7.47 (m, 1H), 7.43–7.35 (m, 4H), 7.13 (t, *J* = 9.8, 1H), 6.94 (td, *J* = 8.5, 2.5, 1H), 6.32 (d, *J* = 2.3, 1H), 5.70 (s, 1H), 4.55 (d, *J* = 6.7, 2H), 3.47 (d, *J* = 23.1, 2H), 3.09 (d, *J* = 3.2, 1H), 2.91–2.76 (m, 2H), 2.68 (t, *J* = 13.8, 1H), 2.59–2.37 (m, 3H), 2.36–2.15 (m, 2H). ^13^C NMR (176 MHz, DMSO-*d*_6_) δ 166.62, 165.09, 163.62 (d, *J* = 251.5), 163.10 (d, *J* = 4.0), 163.11, 163.09, 162.11 (dd, *J* = 245.8, 12.5), 159.35 (dd, *J* = 248.7, 10.2), 150.94, 145.36, 135.06, 134.37, 134.18, 134.14, 133.78, 133.34, 133.29, 130.24, 129.88, 129.65, 129.10, 129.07, 127.63, 126.36 (d, *J* = 11.8), 119.80, 117.14, 117.02, 111.21 (d, *J* = 20.4), 104.23 (t, *J* = 26.8), 75.18 (dd, *J* = 12.6, 5.2), 63.67, 63.61, 56.54, 56.03, 55.95, 54.05, 54.00, 53.91, 45.80, 42.76. HRMS (EI): Calcd for C_33_H_27_Cl_2_F_3_N_6_O_4_S [M + H]^+^ 731.1216. Found: *m*/*z* 731.1033.

**3-Chloro-1-(4-(2-(2,4-difluorophenyl)-2-hydroxy-3-(1*H*-1,2,4-triazol-1-yl)propyl)-piperazin-1-yl)propan-1-one (49)**. 2-(2,4-Difluorophenyl)-1-(piperazin-1-yl)-3-(1*H*-1,2,4-triazol-1-yl)propan-2-ol hydrochloride (**4b**) (700 mg, 1.95 mmol) was suspended in methylene chloride (20 mL), then triethylamine (591 mg, 5.84 mmol, 3 equiv) was added. RM was cooled in an ice bath, and 3-chloropropionyl chloride (296 mg, 2.33 mmol, 1.2 equiv) was added dropwise. Upon full addition of the acyl chloride, the pH of the mixture was adjusted to 8 with Et_3_N if needed. The reaction mixture was allowed to heat to room temperature and stirred overnight. It was then washed with a saturated citric acid solution to achieve an acidic pH, the organic layer was separated, dried over Na_2_SO_4_, and the solvent evaporated under vacuum. The resulting residue was redissolved in CH_2_Cl_2_ and purified by flash chromatography on silica gel (eluent—methanol:CH_2_Cl_2_ 1:25). Yield 80% (647 mg), dark yellow oil. The substance was used in the next step without purification. LC/MS(+ESI): Found *m*/*z* 414.2. Calcd for C_18_H_22_ClF_2_N_5_O_2_ [M + H]^+^ 414.2.

**(*Z*)-5-(4-Chlorobenzylidene)-3-(3-(4-(2-(2,4-difluorophenyl)-2-hydroxy-3-(1*H*-1,2,4-triazol-1-yl)propyl)piperazin-1-yl)-3-oxopropyl)thiazolidin-2,4-dione (50a)** (Method C). Potassium salt of 5-(4-chlorobenzylidene)thiazolidine-2,4-dione (222 mg, 0.8 mmol) is suspended in 4 mL of DMF, then 4-(3-chloroethylcarbonylpiperazin-1-yl)-3-(1*H*-1,2,4-triazolyl-1-yl)propan-2-ol (**49**) (322 mg, 0.8 mmol, 1 equiv.) and potassium iodide (134 mg, 0.8 mmol, 1 equiv) were added. The resulting suspension was stirred for 12 h at 80 °C. Then, the reaction mixture was diluted with water (20 mL) and extracted with ethyl acetate (3 × 3 mL). The organic layer was separated, dried over Na_2_SO_4_, and evaporated on a rotary evaporator. The residue was redissolved in CH_2_Cl_2_, and subjected to chromatography on silica gel (eluent— 5% methanol in methylene chloride). Yield 18%, pale yellow foam. ^1^H NMR (700 MHz, DMSO-*d*_6_) δ 8.30 (s, 1H), 7.93 (s, 1H), 7.75 (s, 1H), 7.65 (d, *J* = 8.7, 2H), 7.62 (d, *J* = 8.6, 2H), 7.40 (d, *J* = 6.9, 1H), 7.19–7.13 (m, 1H), 6.96 (td, *J* = 8.5, 2.5, 1H), 5.73 (s, 1H), 4.58 (s, 2H), 3.84–3.78 (m, 2H), 3.31–3.23 (m, 3H), 2.87 (d, *J* = 13.8, 1H), 2.69 (d, *J* = 13.9, 1H), 2.67–2.61 (m, 2H), 2.47–2.32 (m, 4H). ^13^C NMR (176 MHz, DMSO-*d*_6_) δ 168.18, 167.31, 165.80, 162.12 (dd, *J* = 245.7, 12.4), 159.40 (dd, *J* = 246.9, 12.2), 150.94, 145.38, 135.71, 132.33, 132.20, 131.96, 130.26, 129.95, 126.52 (d, *J* = 11.6), 122.59, 111.21 (d, *J* = 20.3), 104.22, 75.18 (d, *J* = 4.7), 63.87, 56.02, 54.61, 54.27, 45.27, 41.45, 40.48, 38.45, 30.53. LC/MS (ESI): *m*/*z* 617.2. Calculated for C_28_H_27_ClF_2_N_6_O_4_S [M + H]^+^ 617.2.

**(*Z*)-3-(3-(4-(2-(2,4-Difluorophenyl)-2-hydroxy-3-(1*H*-1,2,4-triazol-1-yl)propyl)piperazin-1-yl)-3-oxopropyl)-5-(4-methoxybenzylidene)thiazolidine-2,4-dione (50c)** was obtained as described above from potassium salt of 5-(4-methoxybenzylidene)thiazolidine-2,4-dione **(5c)** and **49**. Yield 10%, white crystals. ^1^H NMR (700 MHz, DMSO-*d*_6_) δ 8.29 (s, 1H), 7.88 (s, 1H), 7.75 (s, 2H), 7.60 (d, *J* = 8.8, 2H), 7.57 (d, *J* = 8.8, 2H), 7.43–7.37 (m, 1H), 7.19–7.14 (m, 1H), 7.11 (t, *J* = 8.2, 4H), 6.96 (td, *J* = 8.5, 2.5, 1H), 4.58 (s, 2H), 3.83 (d, *J* = 4.3, 7H), 3.82–3.79 (m, 1H), 3.30–3.24 (m, 1H), 2.86 (d, *J* = 14.1, 1H), 2.69 (d, *J* = 13.9, 1H), 2.66–2.59 (m, 1H), 2.46–2.32 (m, 4H). ^13^C NMR (176 MHz, DMSO-*d*_6_) δ 168.58, 168.21, 167.63, 166.03, 162.12 (dd, *J* = 245.3, 12.1), 161.65, 161.42, 159.40 (dd, *J* = 246.9, 12.3), 150.94, 145.37, 133.36, 132.72, 132.53, 132.12, 130.25, 126.53 (d, *J* = 13.0), 126.02, 125.88, 121.01, 118.48, 115.47, 115.38, 111.21 (d, *J* = 19.9), 104.22 (t, *J* = 26.9), 75.18 (d, *J* = 5.1), 63.88, 55.99, 55.95, 54.62, 54.28, 45.27, 41.45, 40.48, 38.31, 30.62. LC/MS (ESI): *m*/*z* 613.2. Calculated for C_29_H_30_F_2_N_6_O_5_S [M + H]^+^ 613.2.

**(*Z*)-5-(2,4-Dichlorobenzylidene)-3-(3-(4-(2-(2,4-difluorophenyl)-2-hydroxy-3-(1*H*-1,2,4-triazol-1-yl)propyl)piperazin-1-yl)-3-oxopropyl)thiazolidine-2,4-dione (50g)** was obtained as described above from potassium salt of 5-(2,4-dichlorobenzylidene)thiazolidine-2,4-dione (**5g**) and **49**. Yield 20%, white crystals. ^1^H NMR (700 MHz, DMSO-*d*_6_) δ = 8.32 (s, 1H), 7.95 (s, 1H), 7.86 (d, *J* = 2.1, 1H), 7.76 (s, 1H), 7.62 (dd, *J* = 8.4, 2.0, 1H), 7.58 (d, *J* = 8.4, 1H), 7.41 (d, *J* = 7.1, 1H), 7.20–7.14 (m, 1H), 6.98 (s, 1H), 4.59 (s, 2H), 3.85–3.77 (m, 2H), 2.89 (s, 1H), 2.74 (s, 1H), 2.66 (t, *J* = 7.6, 2H). ^13^C NMR (176 MHz, DMSO-*d*_6_) δ 168.20, 167.09, 165.45, 164.48, 162.77, 162.20 (dd, *J* = 246.0, 12.3), 159.39 (dd, *J* = 247.0, 12.2), 150.98, 145.43, 136.17, 135.86, 130.54, 130.47, 130.44, 130.39, 130.26, 128.89, 128.80, 128.50, 127.81, 127.16, 126.19, 125.91, 111.30 (d, *J* = 19.8), 104.30 (t, *J* = 26.9), 74.97, 63.64, 55.99, 54.48, 54.18, 40.47, 38.54, 36.25, 31.23, 30.41. LC/MS (ESI): *m*/*z* 651.1. Calculated for C_28_H_26_Cl_2_F_2_N_6_O_4_S [M + H]^+^ 651.1.

### 6.2. Biochemistry and Microbiology

MIC determination. The minimal inhibitory concentrations (MICs) of the compounds obtained were determined using the microdilution method in RPMI 1640 medium buffered to pH 7.0 with MOPS [32]. The test compounds were evaluated against human pathogens: Candida spp. and dermatophytes. Fluconazole was used as the reference antifungal drug. Compounds were dissolved in DMSO and serially diluted in nutrient medium (concentration range 0.125–128 mg/L). The inoculum suspension was added to each well and incubated at 35 °C. Microdilution plates were visually inspected after 24–48 h and 96 h of incubation for growth of yeast and filamentous fungi, respectively. MIC was defined as the minimum inhibitory concentration of test compound which resulted in total inhibition of the fungal growth. All susceptibility testing was performed in triplicate. Clinical and reference strains of *Candida* spp.: *C. parapsilosis* ATCC 22019, *C. albicans* ATCC 24433, *C. tropicalis* 3019, *C. kefyr* 77, *C. famata* 312, *C. guilliermondii* 355, *C. parapsilosis* 58N, *C. krusei* 432M, and *C. glabrata* 61L. Clinical and reference strains of filamentous fungi: *A. niger* 37a, *Aspergillus fumigatus* ATCC 46645, *Microsporum canis* B-200, and *Trichophyton rubrum* 2002.

Microbiology. The analysis was carried out by the serial microdilutions method in accordance with ISO 16256:2012 [33] “Clinical laboratory testing and in vitro diagnostic test systems—Reference method for testing the in vitro activity of antimicrobial agents against yeast fungi involved in infectious diseases” and the recommendations of the World Non-Profit Organization for the Development of Standards and Recommendations in Medicine (Reference method for broth dilution antifungal susceptibility testing of filamentous fungi; approved standard, 2nd ed. CLSI document M38-A, Antifungal Susceptibility Testing of Yeasts: M27-A2. 2008. Clinical and Laboratory Standards Institute, Wayne, PA, USA). The samples of all the compounds were dissolved in dimethyl sulfoxide (DMSO) to provide the main solutions with concentration of 10.0 mg/mL. The main solutions were diluted in a nutrient medium to a concentration of 64 μg/mL to make the working solutions, then further diluted to the concentrations from 32.0 to 0.015 μg/mL. A series of double dilutions of the test samples was prepared in 96-well immunological plates in a volume of 100 μL of RPMI-1640 medium with L-glutamine (Sigma-Aldrich) containing 0.2% glucose. After sowing, the yeast cultures were incubated 24–48 h at 35 °C, filamentous fungi—for 48–96 h.

In vivo tests. The *Candida albicans* RCPH Y 1274 strain was obtained from the Russian Collection of Pathogenic Fungi, Research Institute of Medical Mycology named after P.N. Kashkin. The strain is resistant to fluconazole and sensitive to voriconazole. Saburo medium composition: distilled water—1 L, enzymatic peptone—10 g, glucose—40 g, agar—18 g.

Laboratory animals. For this study, white male outbred mice from Rappolovo nursery were used. Body weight at the beginning of the experiment was 20 g. Animals were subjected to adaptation for 7 days of admission to the laboratory. During this period, a daily inspection of the condition of the animals was carried out. Before inclusion in the study, the animals were examined by a veterinarian. Animals with deviations detected during the examination were not included in the study. For the experiment, five groups of animals were formed (six mice in each group) by random selection, including three experimental (No. 1, 2, 3), one group for the introduction of voriconozole (B), and the control group (D—intraperitoneal injection of 0.5 mL 20% DMSO). To compare the efficiency of suppressing *C. albicans* yeast cell germination in the mice glands with different doses of compound **31a** and voriconazole, the percentage of germination of yeast cells in each of these groups of animals was calculated in relation to control group D (20% DMSO). The percentage of inhibition of germination of *C. albicans* cells was calculated as 100% minus percentage of germination.

### 6.3. Molecular Modeling

Docking of ligands in the binding site of CYP51 was done using DOCK 6.9 software [34]. Crystallographic structure of CYP51 from *C. albicans* SC5314 in complex with VT1161 (PDB ID 5TZ1, resolution of 2.0 Å) was chosen as a receptor model and prepared for docking using Chimera [35]. The water molecule coordinated by hydrogen bonds with the hydroxyl group of the VT1161, heme, and Y132 sidechain was considered as essential [36]. It was retained in the receptor’s model because studied ligands shared similar with fluconazole and VT1161 core bearing hydroxyl group at quaternary carbon. Standard procedure of DOCK 6.9 was used to map the binding site and to generate grids [34].

Chimera 1.15 [35] was used to calculate three-dimensional conformations of ligands and protonation states of polar heavy atoms. AM1-BCC charges were added [37] and docking was performed using Grid Score scoring function. The redocking of crystallographic ligand VT1161 reproduced experimental conformation with RMSD over heavy atoms of 0.5 Å (Figure S4A). However, the docking of fluconazole provided inverted binding mode. The latter was attributed to the lack of parametrization for chelating interactions in the scoring function. To address this issue, positional constraints were applied to triazole cycle of the docked ligands to ensure its correct orientation in the chelate complex with heme. Restrained docking produced the expected conformation of fluconazole (Figure S4B) and was used to predict binding modes of studied ligands. Pymol 1.8 (Schrodinger, LLC, New York, NY, USA) and Chimera 1.15 [35] were used for analysis and visualization of binding modes.

### 6.4. Analysis of Homology

The alignment of CYP51 amino acid sequences from *C. albicans* (Uniprot ID P10613) and *C. parapsilosis* ATCC 22019 (Uniprot ID C7EXA5) was conducted using NCBI Blast [37]. The three-dimensional homology model of *C. parapsilosis* CYP51 was built using Swiss-Model web server [38]. The model was superimposed over the crystallographic structure of CYP51 from *C. albicans* (PDB ID 5TZ1) and structure-based sequence alignment was performed in Chimera 1.15 [39], followed by an analysis of the binding site homology using in-house scripts.

## 7. Conclusions

In this work, a series of hybrid molecules based on thiazolidine-4-one and triazole have been synthesized. Various synthetic methods for them have been tested. A microbiological assessment of new hybrid derivatives in comparison with the previously obtained compounds was carried out. The lowest obtained MIC values on strains of yeast and filamentous fungi of the genus *Candida* spp., *Aspergillus* spp., *Rhizopus* spp. for compounds with 4-chloro-, 2,4-dichloro-, and 4-methoxy- substituents range between 0.003 µg/mL and 0.5 µg/mL and are not inferior or several times better than commercial drugs fluconazole, amphotericin B, itraconazole, and ketoconazole. Structural changes made to various fragments of the leader molecule **L-173** did not result in an improvement in MIC values. The homological increase in the linker chain slightly reduced the activity compared to **L-173**, and to a greater extent, this was reflected in the MIC values for the longest linker (compound **34a**, n = 3). The activity remained sufficiently high for all strains of fungi (MIC 0.25–4 µg/mL). At the same time, the addition of a large 2,4-dichlorophenyl substituent (**31b**) next to the thiazolidine core in the hybrid structure completely deprived the molecules of microbiological activity, which is in good agreement with the binding model predicted by docking. The replacement of oxygen for sulfur in the thiazolidine nucleus (**29a**) or fluorine atoms for chlorine atoms in the phenyl nucleus of the triazole fragment (**30a**) slightly reduced the activity of *Candida parapsilosis*, and completely deprived the molecules of activity against *Aspergillus* spp.

Molecular modeling provided a congruent interpretation of the SAR experimentally observed for triazole series. The optimal extended shape of thiazolidinedione arm is crucial for affinity as well as hydrophobic interactions between benzylidene moiety and residues at the gate to the substrate access channel. In vivo the leader **L-173** is twice more effective in inhibiting the formation of germ tubes by *Candida albicans* yeast cells, in the same doses as Voriconazole (31 via 17%). Thus, **L-173** is of interest for medicine and can be used for prevention and treatment of infectious diseases, in particular caused by various fungal infections, e.g.,: dermatophytosis, superficial mycosis, candidiasis of the skin and nails, vaginal candidiasis, candidal stomatitis, endocarditis and other diseases of humans and animals.

## Figures and Tables

**Table 1:** Overview of the synthesized hybrid compounds.

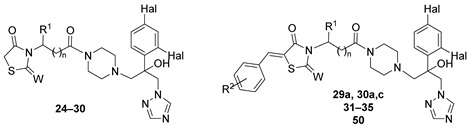							
№	W	n	R^1^	R^2^	Hal	Method	Yield, %
**24**	O	0	H	-	F	A	52
**25**	O	0	Me	-	F	A	25
**26**	O	2	H	-	F	A	69
**27**	O	3	H	-	F	A	65
**28**	O	0	2,4-Cl_2_Ph	-	F	A	34
**29**	S	0	H	-	F	A	20
**3** **0**	S	0	H	-	Cl	A	28
**3** **1a**	O	0	H	4-Cl	F	A, B, K	69, 51, 57
**3** **1b**	O	0	H	4-F	F	A	72
**3** **1c**	O	0	H	4-OMe	F	A	61
**3** **1d**	O	0	H	4-Et	F	A	72
**3** **1e**	O	0	H	4-*i*-Pr	F	A	65
**3** **1f**	O	0	H	4-*t*-Bu	F	A	63
**3** **1g**	O	0	H	2,4-Cl_2_	F	A, K	58, 48
**3** **2a**	O	0	Me	4-Cl	F	A, B	72, 46
**33a**	O	2	H	4-Cl	F	A, B, K	41, 32, 42
**33** **b**	O	2	H	4-F	F	A	37
**33** **g**	O	2	H	2,4-Cl_2_	F	A	45
**33** **h**	O	2	H	2-OH-3,5-Cl_2_	F	K	51
**33** **i**	O	2	H	2-Cl-6-(4-MePip)	F	K	46
**34** **a**	O	3	H	4-Cl	F	K	44
**34b**	O	3	H	4-F	F	K	48
**34g**	O	3	H	2,4-Cl_2_	F	K	44
**34** **h**	O	3	H	2-OH-3,5-Cl_2_	F	K	41
**34** **i**	O	3	H	2-Cl-6-(4-MePip)	F	K	38
**3** **5** **b**	O	0	2,4-Cl_2_Ph	4-F	F	K	33
**50** **a**	O	1	H	4-Cl	F	C	18
**50** **c**	O	1	H	4-OMe	F	C	10
**50** **g**	O	1	H	2,4-Cl_2_	F	C	20
**29** **a**	S	0	H	4-Cl	F	K	60
**30a**	S	0	H	4-Cl	Cl	K	58
**30** **c**	S	0	H	4-OMe	Cl	K	39

**Table 2:** ^1^H, ^13^C, and ^15^N chemical shifts of **L-173** and **L-310** measured in DMSO-*d*_6_ at 298 K and 600 MHz proton resonance frequency.

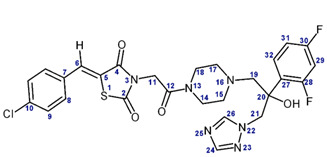 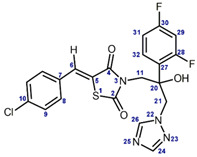					
Position	L-173		L-310		
	δ(^1^H), ppm	δ(^13^C), ppm		δ(^1^H), ppm	δ(^13^C), ppm
2	-	166.8	-	167.4	-
3	-	-	-	-	162.6
4	-	165.2	-	166.0	-
5	-	121.7	-	122.0	-
6	7.92	132.1	7.86	131.5	-
7	-	135.4	-	135.3	-
8	7.63	131.8	7.62	131.9	-
9	7.58	129.5	7.58	129.6	-
10	-	131.7	-	132.2	-
11	4.54	42.6	4.11; 4.13	48.1	-
12	-	162.9	-	-	-
13	-	-	-	-	-
14	3.32	41.8	-	-	-
15	2.38; 2.41	53.7	-	-	-
16	-	-	-	-	-
17	2.43; 2.48	54.0	-	-	-
18	3.37	44.1	-	-	-
19	2.71; 2.88	63.4	-	-	-
20	5.74 (OH)	74.7	6.11 (OH)	74.3	-
21	4.58	55.5	4.59; 4.81	55.0	-
22	-	-	-	-	211.6
23	-	-	-	-	298.4
24	7.75	150.5	7.69	150.6	-
25	-	-	-	-	252.3
26	8.29	144.9	8.27	145.0	-
27	-	126.0	-	123.9	-
28	-	158.9	-	159.5	-
29	7.14	103.8	7.17	104.0	-
30	-	161.7	-	162.1	-
31	6.95	110.8	6.87	110.9	-
32	7.41	129.8	7.25	129.7	-

**Table 3:** In vitro antifungal action (MIC, µg/mL) of **3a**, **4a**, **L-310**, **L-163** against *C. parapsilosis*,* Aspergillus* spp., and dermatophytes.

Strains	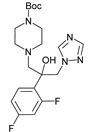		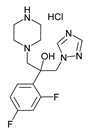		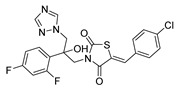		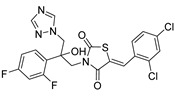	
	3a		4a		L-310^16^		L-163^16^	
	24 h	48 h	24 h	48 h	24 h	48 h	24 h	48 h
*C. parapsilosis* ATCC 22019	1	2–4	32	>32	0.5	1.0	0.5–1.0	2
*A. niger* 37a	8		16–32		4		16	
*M. canis* B-200	16		32		0.5		0.5–1.0	
*T. rubrum* 2002	16		>32		4		4	

**Table 4:** Antifungal activity (MIC, µg/mL) of some hybrid compounds against *C. parapsilosis*, *Aspergillus* spp., and dermatophytes evaluated by the double micro-dilutions method in broth.

№	*C. parapsilosis* ATCC 22019		*A. niger* 37a	*A. fumigatus* 46645	*M. canis* B-200	*T. rubrum* 2002
	24 h	48 h				
**38** **a**	>32	>32	>32	>32	>32	>32
**39a**	4	8	>32	>32	>32	>32
**39b**	4	8	>32	>32	>32	>32
**44a**	16	32	>32	>32	32	32
**45a**	32	32	>32	>32	32	32
**46a**	32	32	>32	>32	32	32
**47a**	0.25	0.5	32	32	8	8–16
**47b**	0.25	0.5	32	32	8	8

**Table 5:** In vitro antifungal activities (MIC, µg/mL) of compounds **47a**,**b** and fluconazole against clinical species of *Candida* spp. and *Cryptococcus neoformans*. * Flc—fluconazole.

Strains	*C. parapsilosis* ATCC 22019		*C. albicans* ATCC 24433		*C. parapsilosis* 58L		*C. tropicalis* 3019		*C. glabrata* 61L		*Cryptococcus neoformans*		*C. albicans* 8 (R)		*C. krusei* 432M	
	24 h	48 h	24 h	48 h	24 h	48 h	24 h	48 h	24 h	48 h	24 h	48 h	24 h	48 h	24 h	48 h
**47a**	0.5	2	16	32	0.5	2	0.5	2	4	32	16	16	2	32	8	32
**47b**	0.5	1	16	32	0.5	1	0.5	1	4	16	16	16	2	32	8	16
Flc *	4	4	>32	>32	8	16	16	16	8	16	32	32	>32	>32	32	>32

**Table 6:** Results of an in vitro study of the antifungal action of the hybrid compounds against *C. parapsilosis*, *Aspergillus* spp., and dermatophytes. Presented values are MIC in µg/mL.

№	*C. parapsilosis* ATCC 22019		*A. niger* 37a	*A. fumigatus* 46645	*M. canis* B-200	*T. rubrum* 2002
	24 h	48 h				
**24**	>32	>32	>32	>32	>32	>32
**25**	>32	>32	>32	>32	>32	>32
**26**	>32	>32	>32	>32	>32	>32
**27**	>32	>32	>32	>32	>32	>32
**28**	>32	>32	>32	>32	>32	>32
**29**	>32	>32	>32	>32	>32	>32
**29a**	0.03	0.25	>32	>32	0.5–1	0.5
**30**	>32	>32	>32	>32	>32	>32
**30a**	0.5	0.5–1	>32	>32	0.5–1	0.5
**30c**	<0.015	0.06	8	16	0.125–0.015	1.0–0.015
**31a (L-173)**	0.03	0.03	1	2	0.015	0.015
**31b**	0.25	2	4	4	0.25	0.25
**31c**	0.03	0.06	>32	>32	0.125–0.03	1.0–0.03
**31d**	0.03	0.5	0.25–0.5	4	1	0.5
**31e**	0.125	0.25	0.5	4	2	0.5–1
**31f**	1	2	1	2–4	2	0.5
**31g**	0.03	0.06	2	4	0.06	0.03
**32a**	0.125	0.5	2	2	0.5	1
**33a**	0.015	0.5–1	4	4	0.5–1	0.25
**33b**	4	8–16	8	8	0.5	0.25
**33h**	8	8	>32	>32	32	32
**33i**	2	2–4	>32	>32	16	16
**34a**	2	2–4	4	4	0.5	0.25
**34b**	0.5	16	4	4	0.5	0.25
**34h**	4	4	>32	>32	16	8
**34i**	8	8	>32	>32	16	8
**35b**	>32	>32	>32	>32	>32	>32
**37g**	1	1–2	4	4–8	0.5–1	32
**38g**	2	>32	8	8	1	0.25
**50a**	0.125	0.25	4	4	0.25	0.125
**50c**	1	1	8	8–16	4	2
**50g**	0.5	0.5–1	8	8	0.25	0.125
**Flu**	0.5	4	>32	16	32	>32
**Itra**	0.03	0.06	0.5	2	0.5	0.25
**AmB**	1	2	0.5	0.25–0.5	0.125	0.25

**Table 7:** In vitro antifungal activities (MIC, µg/mL) of compounds **29a**, **30a**, **31a**,**d**–**g**, and **32a** against clinical species of *Candida* spp. and *Cryptococcus neoformans*.

Strain \ Compound		*C. albicans* 8P	*C. albicans* 8 R	*C. albicans* 604M	*C. parapsilosis* 58L	*C. utilis* 84	*C. tropicalis* 3019	*C. glabrata* 61L	*C. krusei* 432M	*Cryptococcus neoformans*
Itraconazole	24 h	16	32	32	0.25	0.125	2	0.25	0.5	0.125
	48 h	16–32	32	32	0.5	0.5	4	1	0.5	0.125
Ketoconazole	24 h	8	8	8	0.06	0.03	0.25	0.25	0.25	0.03
	48 h	8	8	8	0.5	0.03	0.5	0.25	1	0.03
**29a**	24 h	8	8	8	0.5	1	1	1	1	0.25
	48 h	8	16	32	2	2	1	16	2	0.25
**30a**	24 h	16	32	8	8	8	32	1	16	0.5
	48 h	16	32	16	16	16	32	2	32	0.5
**31a**	24 h	0.015	4	32	0.125	0.125	0.5	0.5	2	0.03
	48 h	4	32	32	0.125	0.25	0.5	2	4	0.03
**31d**	24 h	32	32	16	0.25	2	0.25	0.25	1	0.25
	48 h	>32	32	>32	1	8	1	2	2	4
**31e**	24 h	32	32	16	0.125	16	1	0.25	4	1
	48 h	>32	32	>32	0.25	16	2	>32	8	8
**31f**	24 h	16	>32	8	0.25	16	1	1	1	0.015
	48 h	16	>32	16	0.5	16	2	>32	2	0.06
**31g**	24 h	8	8	8	0.25	0.25	2	2	1	0.06
	48 h	8	8	8	0.5	0.25	4	4	1	0.06
**32a**	24 h	16	32	32	0.5	2	2	1	4	<0.5
	48 h	32	32	32	2	4	8	4	8	<0.5

**Table 8:** Comparative results of **L-173** and voriconazole on clinical strains from HKI. Presented values are MIC in µg/mL.

Strain	Strain-N	HKI 12034018, L-173	Voriconazole	Remark
*Candida albicans*	2016-303	<0.016	<0.016/0.03	Ecninocandin-R (Mut)
*Candida auris*	2019-731	2	8	Azol-R
*Candida glabrata*	2018-606	>8	>8	Ecninocandin-R (Mut)
*Candida guilliermondii*	2020-194	0.25	0.125	
*Candida krusei*	2017-144	2	0.25	
*Candida parapsilosis*	ATCC 22019	0.03	0.03	
*Aspergillus fumigatus*	2020-364	>8	>8	Azol-R (Mut)
*Aspergillus fumigatus*	ATCC 204305	1	0.5	Azol-S
*Fusarium petrolifilum*	2020-183	>8	>8	
*Lomentospora prolificans*	2020-066	>8	>8	
*Rhizopus arrhizus*	2020-227	0.25	4	
*Scedosporium apiospermum*	2020-135	>8	0.5	

**Table 9:** Suppression of *C. albicans* (strain RCPG Y 1274) growth tube formation in the glands of mice by various doses of compound **L-173** (**31a**) and voriconazole.

Animal Group	Administered Compound	Dose, mg/kg	% Suppression **	*p* ***
1	**L-173** *	5	22.33	0.00001
2	**L-173** *	12	30.86	0.00001
3	**L-173** *	25	41.04	0.00001
4	Voriconazole *	12	17.00	0.00001
D (control)	-		0	

* Due to the poor solubility of compound **L-173** and voriconazole, 20% DMSO in water was used as a solvent. ** % inhibition—the proportion of cells that did not form growth tubes; *** *p*—comparison with voriconazole.

## Data Availability

Data are contained within the article and Supplementary Materials.

## Supplementary Materials

The following supporting information can be downloaded at: https://www.mdpi.com/article/10.3390/ph17060723/s1. References [40,41] are cited in the supplementary materials.
