# Supplementary material for: Synthesis and Biological Evaluation of a Series of New Hybrid Amide Derivatives of Triazole and Thiazolidine-2,4-dione
Source: Pharmaceuticals (Basel). 2024 Jun 3;17(6):723. doi: 10.3390/ph17060723 (PMC11206592; doi:10.3390/ph17060723)

Supporting information

Synthesis and Biological Evaluation of a Series of New Hybrid Amide Derivatives of Triazole and Thiazolidine-2,4-dione

Igor B. Levshin, Alexander Yu. Simonov, Alexey A. Panov, Natalia E. Grammatikova, Alexander I. Alexandrov, Eslam S.M.O. Ghazy, Vasiliy A. Ivlev, Michael O. Agaphonov, Alexey B. Mantsyzov, Vladimir I. Polshakov

Table of contents

Table of contents 1
1. CHEMISTRY 2
   Triazole derivatives 2
   Alkylation of thiazolidine-2,4-dione derivatives 3
   Acid-catalyzed ester hydrolysis 4
   Knoevenagel reaction (5-arylidenethiazolidinediones synthesis) 5
   Synthesis of amides 8
   References 10
2. MOLECULAR MODELING 12
3. NMR SPECTRA 16

1. CHEMISTRY

The chemical shifts are expressed in ppm (δ scale) using DMSO or $CDCl_3$ as an internal standard, the coupling constants expressed in Hz.

Triazole derivatives

***tert*-Butyl 4-(2-(2,4-difluorophenyl)-2-hydroxy-3-(1*H*-1,2,4-triazol-1-yl)propyl)-piperazine-1-carboxylate (3a)** A mixture of 1-((2-(2,4-difluorophenyl)oxiran-2-yl)methyl)-1*H*-1,2,4-triazole methanesulfonate (17.2 g, 51.7 mmol), Boc-piperazine (9.6 g, 51.7 mmol, 1 equiv.) and triethylamine (10.4 ml, 1.5 equiv.) were refluxed in ethanol for 3 h. After that, the solvent is evaporated, the residue redissolved in methylene chloride and washed with aqueous solution of citric acid until acidic, then with water. The organic phase is dried over Na_2SO_4 and evaporated. The product in the form of oil crystallized upon standing. Yield 13.02 g (59%). ^{1}H NMR (700 MHz, DMSO-d_6) δ 8.30 (s, 1H), 7.75 (s, 1H), 7.43 – 7.38 (m, 1H), 7.19 – 7.10 (m, 1H), 6.96 (td, *J* = 8.5, 2.4, 1H), 5.70 (s, 1H), 4.58 (s, 2H), 3.17 (s, 4H), 2.86 (d, *J* = 14.0, 1H), 2.68 (d, *J* = 13.9, 1H), 2.35 (d, *J* = 21.8, 4H), 1.36 (s, 9H). ^{13}C NMR (176 MHz, DMSO-d_6) δ 162.12 (dd, *J* = 245.7, 12.3), 159.39 (dd, *J* = 246.6, 12.1), 154.24, 150.92, 145.36, 130.48 – 129.98 (m), 126.55 (d, *J* = 13.1), 111.18 (d, *J* = 20.0), 104.19 (t, *J* = 26.9), 79.13, 75.12 (d, *J* = 5.2), 63.98, 56.04 (d, *J* = 3.9), 54.36, 28.47. LC/MS(+ESI): Found *m/z* 424.2. Calcd for $C_{20}H_{27}F_2N_5O_3$ $[M+H]^+$ 424.2.

2-(2,4-Difluorophenyl)-1-(piperazin-1-yl)-3-(1*H*-1,2,4-triazol-1-yl)propan-2-ol hydrochloride (3b). A solution of **3a** (10.0 g, 23.6 mmol) was dissolved in a saturated solution of hydrogen chloride in ethyl acetate (60 ml) and stirred overnight at room temperature. The precipitate formed was filtered off and washed with ethyl acetate. Yield 8.44 g (98%). ^{1}H NMR (500 MHz, DMSO-d_6) δ = 9.63 (br, 2H), 8.56 (s, 1H), 7.88 (s, 1H), 7.42 (s, 1H), 7.24 (m, 1H), 7.02 (m, 1H), 4.79 (d, *J* = 14.0, 1H), 4.70 (d, *J* = 14.0, 1H), 4.56 (br, 1H), 4.02 (q, *J* = 7.1, 2H), 3.71-3.19 (m, 8H). LC/MS(+ESI): Found *m/z* 324.2. Calcd for $C_{15}H_{19}F_2N_5O$ $[M+H]^+$ 324.2.

1-(2,4-dichlorophenyl)-2-(1*H*-1,2,4-triazol-1-yl)ethan-1-one 2,2',4'-Trichloroacetophenone (4 g, 18 mmol), triazole (1.48 g, 21 mmol, 1.2 equiv) and sodium bicarbonate (2.26 g, 27 mmol, 1.5 equiv) were suspended in absolute toluene (120 ml) and refluxed overnight with a calcium chloride tube. Then the reaction mixture was cooled, toluene was distilled off on a rotary evaporator and the residue was partitioned between water and ethyl acetate. The organic layer was separated, washed with saturated sodium chloride solution, dried over sodium sulfate, and ethyl acetate was distilled off on a rotary evaporator. The resulting residue was dissolved in methylene chloride, and the substance was purified by flash chromatography on silica gel (eluent – methanol:CH_2Cl_2 1:33). Yield 2.56 g (56%), light brown powder. ^{1}H NMR ($CDCl_3$): 8.28 (s, 1H), 7.97 (s, 1H), 7.64 (d, *J* = 8.4, 1H), 7.49 (d, *J* = 2.0, 1H), 7.36 (dd, *J* = 8.4, 2.0, 1H), 5.62 (s, 2H). LC/MS(+ESI): Found *m/z* 256.0. Calcd for $C_{10}H_7Cl_2N_3O$ $[M+H]^+$ 256.0.

1-((2-(2,4-dichlorophenyl)oxiran-2-yl)methyl)-1*H*-1,2,4-triazole To a mixture of 2,4-dichloro-(1*H*-1,2,4-triazolyl)acetophenone (2.0 g, 7.81 mmol, 1 equiv.), trimethylsulfoxonium iodide (1.72 g, 7.81 mmol, 1 equiv) and hexadecyltrimethylammonium bromide (71 mg, 0.20 mmol, 0.025 equiv.) in 20 ml of toluene is added 1.28 ml of a 20% sodium hydroxide solution (312 mg, 7.81 mmol, 1 equiv). The mixture is stirred at 60 °C for 2 hours. The emulsion is filtered, the toluene phase is separated and evaporated. The resulting substance is pure enough for the

next step. Quantitative yield (2.1 g), brown oil. ^{1}H NMR ($CDCl_3$): 8.10 (s, 1H), 7.88 (s, 1H), 7.38 (d, *J* = 1.8, 1H), 7.16 (dd, J_1 = 8.2, 1.8, 1H), 7.10 (d, *J* = 8.2, 1H), 4.86 (d, *J* = 14.9, 1H) 4.48 (d, *J* = 14.9, 1H), 2.96 (d, *J* = 4.3, 1H), 2.88 (d, *J* = 4.3, 1H). LC/MS(+ESI): Found *m/z* 270.0. Calcd for $C_{11}H_9Cl_2N_3O$ $[M+H]^+$ 270.0.

***tert*-Butyl 4-(2-(2,4-dichlorophenyl)-2-hydroxy-3-(1*H*-1,2,4-triazol-1-yl)-propyl)piperazine-1-carboxylate (3b)** A mixture of 1-((2-(2,4-dichlorophenyl)oxiran-2-yl)methyl)-1*H*-1,2,4-triazole (2.1 g, 7.77 mmol), Boc-piperazine (1.45 g, 7.77 mol, 1 equiv) and triethylamine (944 mg, 9.33 mmol, 1.2 equiv.) were refluxed in ethanol overnight. Ethanol is evaporated, the residue redissolved in methylene chloride and washed with aqueous citric acid to achieve acidic pH, and then with water. The organic phase was dried over Na_2SO_4 and evaporated. The residue is dissolved in methylene chloride and the substance is purified by chromatography on silica gel (eluent – methanol:CH_2Cl_2 1:33). Yield 830 mg (26%), brown oil. The substance was used in the next step without purification. LC/MS(+ESI): Found *m/z* 456.2. Calcd for $C_{20}H_{27}Cl_2N_5O_3$ $[M+H]^+$ 456.2.

2-(2,4-dichlorophenyl)-1-(piperazin-1-yl)-3-(1*H*-1,2,4-triazol-1-yl)propan-2-ol hydrochloride (4b) A solution of *tert*-butyl 4-(2-(2,4-dichlorophenyl)-2-hydroxy-3-(1*H*-1,2,4-triazol-1-yl)propyl)piperazine-1-carboxylate (456 mg) is suspended in 5 ml of saturated hydrogen chloride solution in ethyl acetate and stirred overnight at room temperature, while a precipitate gradually forms. The next day, the precipitate is filtered off and washed with ethyl acetate. Yield 381 mg (97%). LC/MS(+ESI): Found *m/z* 356.1. Calcd for $C_{15}H_{19}Cl_2N_5O$ $[M+H]^+$ 356.1.

Alkylation of thiazolidine-2,4-dione derivatives

Ethyl 2-(2,4-dioxothiazolidin-3-yl)acetate (**6**). To a solution of thiazolidine-2,4-dione (1 g, 8.5 mmol) and ethyl bromoacetate (3.2 g, 17 mmol) in THF (25 mL), potassium carbonate (2.35 g, 17 mmol) was added. The mixture was refluxed with stirring for 5 h, then the solvent was evaporated under reduced pressure. The residue was washed with methanol to provide 2-(2,4-dioxothiazolidin-3-yl)acetic acid ethyl ester (1.62 g, 93%) as white oil. ^{1}H NMR (400 MHz, DMSO-d_6) δ 4.32 (2H, s), 4.20 (2H, s), 4.29 (2H, s), 4.12 (2H, q, *J* = 7.1), 1.18 (3H, t, *J* = 7.1). ^{13}C NMR (101 MHz, DMSO-d_6) δ 172.20, 171.77, 167.14, 61.93, 42.16, 42.34, 34.43, 14.36. HRMS (EI): Calcd for $C_7H_9NO_4S$ $[M+H]^+$ 204.0325. Found: *m/z* 204.0355. Spectroscopic data matched that reported in literature[1].

Methyl 2-(2,4-dioxothiazolidin-3-yl)propanoate (**7**). Methyl 2-bromopropionate (6.0 g, 1.05 equiv) and potassium salt of thiazolidine-2,4-dione (5,0 g, 1 equiv) in 50 ml of DMF were stirred overnight at room temperature with control by TLC (eluent: CH_2Cl_2). The reaction mixture was diluted with water (75 ml) and extracted with diethyl ether (2*20 ml). The organic layer was separated, dried over sodium sulfate and the solvent was removed on a rotary evaporator. The resulting residue was dissolved in a minimum amount of methylene chloride, diluted with hexane until cloudy and purified by flash chromatography on silica gel (hexane:CH_2Cl_2 4:1). Yield 4.95 g (70%). ^{1}H NMR (400 MHz, $CDCl_3$) δ 1.55 (d, *J* = 7.8, 3H), 3.71 (s, 3H), 3.97 (s, 2H), 4.88 (q, *J* = 7.8, 1H); ^{13}C NMR (101 MHz, $CDCl_3$) δ 14.1, 33.6, 50.5, 52.9, 169.0, 170.5, 170.9. LC/MS(+ESI): Found *m/z* 204.0. Calcd for $C_7H_9NO_4S$ $[M+H]^+$ 204.0. Spectroscopic data matched that reported in literature.[2]

Methyl 4-(2,4-dioxothiazolidin-3-yl)butanoate (8)[3-5] Potassium salt of thiazolidine-2,4-dione (1.0 g, 6.44 mmol, 1 equiv) is suspended in dry DMF (15 ml) and 4-bromobutyric acid methyl ester (1.17 g, 6.44 mmol, 1 equiv) is added. The reaction

mixture is stirred for 16 hours at 50 °C, monitored by TLC (CH_2Cl_2:hexane 3:1). RM was diluted with water (15 ml) and extracted with diethyl ether (2*20 ml). The combined extracts were dried over sodium sulfate and the solvent removed on a rotary evaporator. The resulting residue was dissolved in a minimum amount of methylene chloride, diluted with hexane until cloudy and purified by flash chromatography on silica gel (hexane:CH_2Cl_2 4:1). Yield 816 mg (58%), colorless oil. ^1H NMR (400 MHz, DMSO-*d6*) δ 4.13 (s, 2H), 3.57 (s, 3H), 3.51 (t, *J* = 6.8, 2H), 2.31 (t, *J* = 7.3, 2H), 1.75 (p, *J* = 6.8, 2H). ^{13}C NMR (101 MHz, DMSO-*d*6) δ 172.96, 172.65, 172.34, 51.61, 40.97, 34.23, 31.04, 22.83.

Ethyl 5-(2,4-dioxothiazolidin-3-yl)-valerate (9)[4,5] TZD potassium salt (1 g, 6.44 mmol) is suspended in 15 ml of absolute DMF and ethyl 5-bromovalerate (1.35 g, 6.5 mmol, 1 equiv) is added and the reaction mixture is stirred for 16 hours at 50 °C, monitored by TLC (CH_2Cl_2:hexane 3:1). RM was diluted with water (15 ml) and extracted with diethyl ether (2*20 ml). The combined extracts were dried over sodium sulfate and the solvent removed on a rotary evaporator. The resulting residue was dissolved in a minimum amount of methylene chloride, diluted with hexane until cloudy and purified by flash chromatography on silica gel (hexane:CH_2Cl_2 4:1). Yield 886 mg (56%), colorless oil. ^1H NMR ($CDCl_3$): δ = 4.12 (s, 2H), 3.94 (s, 2H), 3.64 (m, 2H), 2.32 (m, 2H), 1.64 (m, 4H), 1.25 (t, 3H, J_1 = 6.84). LC/MS(+ESI): Found *m/z* 246.1. Calcd for $C_{10}H_{15}NO_4S$ $[M+H]^+$ 246.1.

Methyl 2-(2,4-dichlorophenyl)-2-(2,4-dioxothiazolidin-3-yl)acetate (10)[4] Methyl 2-bromo-2-(2,4-dichlorophenyl)acetate (14.24 g, 47.8 mmol, 1 equiv) and thiazolidine-2,4-dione potassium salt (7.42 g, 47.9 mmol 1 equiv) in 50 ml of acetonitrile are stirred overnight at room temperature. After completion, as seen by TLC control (eluent: CH_2Cl_2), the precipitate is filtered off, washed with acetonitrile, and the solution is evaporated. The residue is dissolved in methylene chloride and purified by flash chromatography on silica gel (eluent: methylene chloride). The fractions were evaporated to give a yellowish crystalline powder. Yield 6.8 g (43%). ^1H NMR (500 MHz, $CDCl_3$): δ = 7.51 (d, *J* = 8.3, 1H), 7.43 (d, *J* = 2.0, 1H), 7.27 (m, 1H), 6.39 (s, 1H), 4.02 (s, 2H), 3.83 (s, 3H). LC/MS(+ESI): Found *m/z* 334.0. Calcd for $C_{12}H_9Cl_2NO_4S$ $[M+H]^+$ 334.0.

Acid-catalyzed ester hydrolysis

2-(2,4-Dioxothiazolidin-3-yl)acetic acid (**11**) A solution of **6** (1.0 g, 5 mmol) in glacial acetic acid (25 mL) with addition of 2 mL 2N HCl was refluxed for 2 h. After evaporation to dryness *in vacuo*, the crude oil was washed with water and ethanol to provide pure (2,4-dioxothiazolidin-3-yl)acetic acid (0.82 g, 94%) as a white amorphous solid. ^1H NMR (400 MHz, DMSO-*d6*): δ 13.30 (1H, s), 4.32 (2H, s), 4.20 (2H, s). ^{13}C NMR (101 MHz, DMSO-*d*6) δ 171.90, 171.48, 168.11, 42.06, 34.04. LC/MS(+ESI): Found *m/z* 176.0. Calcd for $C_5H_5NO_4S$ $[M+H]^+$ 176.0. Spectroscopic data matched that reported in literature.[1]

2-(2,4-Dioxothiazolidin-3-yl)propanoic acid (**12**)[4] The same procedure as above was carried out with **7**. Yield 2.51 g (90%), white crystals. ^1H NMR (700 MHz, DMSO-*d*6) δ 12.64 (s, 1H), 3.50 (q, *J* = 7.2, 1H), 3.41 (d, *J* = 15.5, 1H), 3.36 (d, *J* = 15.4, 1H), 1.31 (d, *J* = 7.2, 3H). ^{13}C NMR (176 MHz, DMSO-*d*6) δ 174.02, 171.47, 41.14, 33.40, 17.45. LC/MS(+ESI): Found *m/z* 190.0. Calcd for $C_6H_7NO_4S$ $[M+H]^+$ 190.0.

4-(2,4-Dioxothiazolidin-3-yl)butanoic acid (13)[4,5] The same procedure as above was carried out with **8**. Yield 98%, colorless oil. ^1H NMR (400 MHz, DMSO-*d*6) δ 12.07 (s, 1H), 4.13 (s, 2H), 3.50 (t, *J* = 6.8, 2H), 2.20 (t, *J* = 7.3, 2H), 1.76 – 1.64 (m,

2H). ^{13}C NMR (101 MHz, DMSO-d_6) δ 174.16, 172.90, 172.53, 40.97, 34.34, 31.19, 22.87. LC/MS(+ESI): Found *m/z* 204.0. Calcd for $C_7H_9NO_4S$ $[M+H]^+$ 204.0.

5-(2,4-Dioxothiazolidin-3-yl)pentanoic acid (14)[3-5] The same procedure as above was carried out with **9**. Yield 94%, colorless oil. ^1H NMR ($CDCl_3$): δ = 8.72 (br, 1H), 3.96 (s, 2H), 3.66 (m, 2H), 2.40 (m, 2H), 1.67 (m, 4H). LC/MS(+ESI): Found *m/z* 218.0. Calcd for $C_8H_{11}NO_4S$ $[M+H]^+$ 218.0.

2-(2,4-Dichlorophenyl)-2-(2,4-dioxothiazolidin-3-yl)acetic acid (15)[4] The same procedure as above was carried out for Yield 6.51 g (98%). ^1H NMR (500 MHz, DMSO-d_6) δ = 12.25 (br.s, 1H), 7.68 (d, *J* = 2.0, 1H), 7.49 (s, 1H), 7.48 (d, *J* = 2.0, 1H), 6.07 (s, 1H), 4.46 – 4.34 (m, 2H). LC/MS(+ESI): Found *m/z* 320.0. Calcd for $C_{11}H_7Cl_2NO_4S$ $[M+H]^+$ 320.0.

Knoevenagel reaction (5-arylidenethiazolidinediones synthesis)

***(Z)*-5-(4-Chlorobenzylidene)thiazolidine-2,4-dione** (**16a**). Thiazolidine-2,4-dione (27.49 mmol) was suspended in 50 ml of acetic acid, and 4-chlorobenzaldehyde (27.49 mmol, 1 equiv) was added, followed by 40% aqueous methylamine solution (16.49 mmol, 0.6 equiv). The reaction mass was stirred for 8 h at 100 °C, then cooled to room temperature. The precipitate was filtered off, washed with water (2 × 30 ml), then with diethyl ether (40 ml) and dried in air. Yield 91%, white crystals. ^1H NMR (400 MHz, DMSO-d_6) δ 12.63 (1H, s, NH), 7.71 (1H, s), 7.61 – 7.51 (m, 4H). ^{13}C NMR (101 MHz, DMSO-d_6) δ 167.99, 167.59, 135.42, 132.30, 132.00 (2C), 130.82, 129.74 (2C), 124.66. LC/MS(+ESI): Found *m/z* 240.0. Calcd for $C_{10}H_6ClNO_2S$ $[M+H]^+$ 240.0.

***(Z)*-5-(4-Fluorobenzylidine)thiazolidine-2,4-dione (16b)**. The same procedure as above was carried out with 4-fluorobenzaldehyde. Yield 81%, white crystals. ^1H NMR (400 MHz, DMSO-d_6) δ 12.64 (s, 1H), 7.80 (s, 1H), 7.67 (dd, *J* = 8.5, 5.2, 2H), 7.38 (t, *J* = 8.6, 2H). ^{13}C NMR (101 MHz, DMSO-d_6) δ 167.96, 167.70, 163.81, 163.80 (d, *J* = 251.3), 132.46 (d, *J* = 4.7), 132.39, 129.81 (d, *J* = 3.0), 123.63 (d, *J* = 2.3), 116.59 (d, *J* = 22.1), 116.39. LC/MS(+ESI): Found *m/z* 224.0. Calcd for $C_{10}H_6FNO_2S$ $[M+H]^+$ 224.0.

***(Z)*-5-(4-Methoxybenzylidine)thiazolidine-2,4-dione (16c)**. The same procedure as above was carried out with 4-methoxybenzaldehyde.Yield 90%, white crystals.^1H NMR (400 MHz, DMSO-d_6): δ 7.72 (s, 1H, CH), 7.55 (d, *J* = 8.0, 2H, ArH), 7.09 (d, *J* = 8.0, 2H, ArH), 3.82 (s, 3H, CH_3). LC/MS(+ESI): Found *m/z* 236.0. Calcd for $C_{11}H_9NO_3S$ $[M+H]^+$ 236.0.

***(Z)*-5-(4-Ethylbenzylidine)thiazolidine-2,4-dione (16d)**. The same procedure as above was carried out with 4-ethylbenzaldehyde**.** Yield 83%, light yellow crystals.^1H NMR (500 MHz, $CDCl_3$): δ 12.56 (br, 1H), 7.75 (s, 1H), 7.49 (d, 2H, *J* = 8.1), 7.36 (d, 2H, *J* = 8.1), 2.64 (q, 2H, *J* = 7.6), 1.18 (t, 3H, *J* = 7.6). LC/MS(+ESI): Found *m/z* 234.1. Calcd for $C_{12}H_{11}NO_2S$ $[M+H]^+$ 234.1.

***(Z)*-5-(4-Isopropylbenzylidine)thiazolidine-2,4-dione (16e)**. The same procedure as above was carried out with 4-isopropylbenzaldehyde. Yield 75%, white crystals. ^1H NMR (500 MHz, $CDCl_3$): δ 12.57 (br, 1H), 7.75 (s, 1H), 7.51 (d, 2H, *J* = 8.3), 7.39 (d, 2H, *J* = 8.3), 2.92 (m, 1H), 1.20 (m, 6H). LC/MS(+ESI): Found *m/z* 248.1. Calcd for $C_{13}H_{13}NO_2S$ $[M+H]^+$ 248.1.

***(Z)*-5-(4-*tert*-Butylbenzylidine)thiazolidine-2,4-dione (16f)**. The same procedure as above was carried out with 4-(*tert*-butyl)benzaldehyde**.** Yield 77%, white crystals. ^1H NMR (500 MHz, $CDCl_3$): δ = 12.57 (br, 1H), 7.75 (s, 1H), 7.55 (d, 2H, *J* = 8.8), 7.52 (d, 2H, *J* = 8.8), 1.29 (s, 9H). LC/MS(+ESI): Found *m/z* 262.1. Calcd for $C_{14}H_{15}NO_2S$ $[M+H]^+$ 262.1.

***(Z)*-5-(2,4-Dichlorobenzylidene)thiazolidine-2,4-dione (16g).** The same procedure as above was carried out with 2,4-dichlorobenzaldehyde. Yield 76%, light yellow powder. ^{1}H NMR (400 MHz, DMSO-d_6) δ 12.73 (1H, s, NH), 7.81 (1H, s, C=CH), 7.76 (1H, s, Ar), 7.55-7.52 (2H, m, Ar). ^{13}C NMR (101 MHz, DMSO-d_6) δ: 167.72 (C2=O), 167.97 (C4=O), 135.88, 135.79, 130.52, 130.40, 128.72, 128.34, 125.97. LC/MS(+ESI): Found *m/z* 273.9. Calcd for $C_{10}H_5Cl_2NO_2S$ [M+H]$^+$ 273.9.

Ethyl *(Z)*-2-(5-(4-chlorobenzylidene)-2,4-dioxothiazolidin-3-yl)acetate (17a)[7] was prepared by the method described for **9.** Yield 78%, colorless crystals. ^{1}H NMR (600 MHz, $CDCl_3$) δ 7.90 (1H, s), 7.46 (2H, d, *J* = 8.4), 7.42 (2H, d, *J* = 8.4), 4.68 (2H, s), 4.22 (2H, q, *J* = 7.1), 1.27 (3H, t, *J* = 7.1). ^{13}C NMR (151 MHz, $CDCl_3$) δ 164.67, 164.34, 163.23, 137.60, 134.95, 131.55, 129.77, 119.39, 113.90, 44.82, 42.07, 14.08.

Ethyl *(Z)*-2-(5-(4-fluorobenzylidene)-2,4-dioxothiazolidin-3-yl)acetate (17b)[7] was prepared by the method described for **9**. Yield 58%, white crystals. ^{1}H NMR (400 MHz, DMSO-d_6) δ 8.02 (s, 1H), 7.74 (dd, *J* = 8.7, 5.6, 2H), 7.41 (t, *J* = 8.8, 2H), 4.51 (s, 2H), 4.19 (q, *J* = 7.1, 2H), 1.22 (t, *J* = 7.1, 3H). Calcd for $C_{14}H_{12}FNO_4S$ 309.31. HRMS (EI): Calcd for $C_{14}H_{12}FNO_4S$ [M+H]$^+$ 310.0544. Found: *m/z* 310.0550.

Methyl *(Z)*-2-(5-(4-methoxybenzylidene)-2,4-dioxothiazolidin-3-yl)acetate (17c)[8] was prepared by the method described for **9**. Yield 69%, white crystals. ^{1}H (400 MHz, $CDCl_3$): δ 7.92 (s, 1H, CH), 7.51 (d, *J* = 8.0, 2H, ArH), 7.03 (d, *J* = 8.0, 2H, ArH), 4.51 (s, 2H, CH_2), 3.90 (s, 3H, CH_3), 3.81 (s, 3H, CH_3). LC/MS(+ESI): Found *m/z* 308.1. Calcd for $C_{14}H_{13}NO_5S$ [M+H]$^+$ 308.1.

Ethyl *(Z)*-2-(5-(4-ethylbenzylidene)-2,4-dioxothiazolidin-3-yl)acetate (17d) was prepared by the method described for **9**. Yield 89%, colorless crystals. ^{1}H NMR (500 MHz, $CDCl_3$) δ = 7.93 (s, 1H), 7.46 (d, *J* = 8.3, 2H), 7.33 (d, *J* = 8.3, 2H), 4.48 (s, 2H), 4.25 (q, *J* = 6.8, 2H), 2.71 (q, *J* = 7.3, 2H), 1.29 (m, 6H). LC/MS(+ESI): Found *m/z* 320.1. Calcd for $C_{16}H_{17}NO_4S$ [M+H]$^+$ 320.1.

Ethyl *(Z)*-2-(5-(4-isopropylbenzylidene)-2,4-dioxothiazolidin-3-yl)acetate (17e) was prepared by the method described for **9**. Yield 70%, colorless crystals. ^{1}H NMR (500 MHz, $CDCl_3$): δ = 7.93 (s, 1H), 7.47 (d, 2H, *J* = 8.3), 7.36 (d, 2H, *J* = 8.3), 4.48 (s, 2H), 4.25 (m, 2H), 2.97 (m, 1H), 1.29 (m, 9H). LC/MS(+ESI): Found *m/z* 334.1. Calcd for $C_{17}H_{19}NO_2S$ [M+H]$^+$ 334.1.

Ethyl *(Z)*-2-(5-(4-*tert*-butylbenzylidene)-2,4-dioxothiazolidin-3-yl)acetate (17f) was prepared by the method described for **9**. Yield 86%, colorless crystals. ^{1}H NMR (500 MHz, $CDCl_3$): δ = 7.94 (s, 1H), 7.52 (d, 2H, *J* = 8.3), 7.48 (d, 2H, *J* = 8.3), 4.49 (s, 2H), 4.25 (m, 2H), 1.36 (s, 9H), 1.31 (m, 3H). LC/MS(+ESI): Found *m/z* 348.1. Calcd for $C_{18}H_{21}NO_4S$ [M+H]$^+$ 348.1.

Ethyl *(Z)*-2-(5-(2,4-Dichlorobenzylidene)-2,4-dioxothiazolidin-3-yl)acetate (17g) was prepared by the method described for **9**. Yield 69%, colorless crystals. ^{1}H NMR (400 MHz, DMSO-d_6) δ 7.99 (s, 1H), 7.83 (s, 1H), 7.59 (s, 2H), 4.50 (s, 2H), 4.16 (d, *J* = 6.8, 2H), 1.20 (t, *J* = 6.6, 3H). ^{13}C NMR (101 MHz, DMSO-d_6) δ 166.98, 166.80, 164.88, 136.44, 135.95, 130.63, 130.44, 130.19, 128.84, 128.46, 125.29, 62.17, 42.79, 14.39. LC/MS(+ESI): Found *m/z* 360.0. Calcd for $C_{14}H_{11}Cl_2NO_4S$ [M+H]$^+$ 360.0.

Ethyl *(Z)*-2-(5-(4-chlorobenzylidene)-2,4-dioxothiazolidin-3-yl)propionate (18a) was prepared by the method described for **9**. Yield 59%, light yellow crystals. ^{1}H NMR (500 MHz, $CDCl_3$) δ = 7.87 (s, 1H), 7.47 (m, 4H), 5.08 (q, *J* = 7.1, 1H), 4.25 (q, *J* = 7.3, 2H), 1.68 (d, *J* = 7.1, 3H), 1.28 (t, *J* = 7.3, 3H). LC/MS(+ESI): Found *m/z* 340.0. Calcd for $C_{15}H_{14}ClNO_4S$ [M+H]$^+$ 340.0.

Ethyl *(Z)*-4-(5-(4-chlorobenzylidene)-2,4-dioxothiazolidin-3-yl)butanoate (**19a**). Yield 49%, white crystals. ^{1}H NMR (500 MHz, $CDCl_3$) δ = 7.83 (s, 1H), 7.49 – 7.39 (m, 4H), 4.13 (q, *J* = 7.1, 2H), 3.82 (t, *J* = 6.8, 2H), 2.37 (t, *J* = 7.3, 2H), 2.01 (m,

2H), 1.25 (t, *J* = 7.1, 3H). LC/MS(+ESI): Found *m/z* 354.1. Calcd for $C_{16}H_{16}ClNO_4S$ $[M+H]^+$ 354.1.

***(Z)*-2-(5-(4-Chlorobenzylidene)-2,4-dioxothiazolidin-3-yl)acetic acid (20a)**[7,8] was prepared by the method described for **11**. Yield 97%, colorless crystals. ^{1}H NMR (600 MHz, $CDCl_3$) δ 7.90 (1H, s), 7.46 (2H, d, *J* = 8.4), 7.42 (2H, d, *J* = 8.4), 4.68 (2H, s). ^{13}C NMR (151 MHz, $CDCl_3$) δ 164.67, 164.34, 163.23, 137.60, 134.95, 131.55, 129.77, 119.39, 113.90, 44.82. HRMS (EI): Calcd for $C_{12}H_8ClNO_4S$ $[M+H]^+$ 297.9935. Found: 297.9933.

***(Z)*-2-(5-(4-Fluorobenzylidene)-2,4-dioxothiazolidin-3-yl)acetic acid (20b)**[7] was prepared by the method described for **11**. Yield 86%, white crystals. ^{1}H NMR (400 MHz, DMSO-d6) δ 13.44 (s, 1H), 7.95 (s, 1H), 7.67 (dd, *J* = 8.5, 5.2, 2H), 7.35 (t, *J* = 8.6, 2H), 4.37 (s, 2H). ^{13}C NMR (101 MHz, DMSO-*d6*) δ 168.42, 167.20, 165.39, 163.55 (d, *J* = 251.3), 133.24 (d, *J* = 4.7), 133.12, 129.82 (d, *J* = 3.0), 120.73 (d, *J* = 2.3), 116.99 (d, *J* = 22.1), 42.71. HRMS (EI): Calcd for $C_{12}H_8FNO_4S$ $[M+Na]^+$ 304.0050. Found: 304.0052.

***(Z)*-2-(5-(4-Methoxybenzylidene)-2,4-dioxothiazolidin-3-yl)acetic acid (20c)**[7,8] was prepared by the method described for **11**. Yield 78%, colorless crystals. ^{1}H NMR (DMSO-*d6*): δ 13.35 (1H, s, COOH), 7.94, (1H, m, CH), 7.61, 7.11 (d, J=8.7, 4H, Ph), 4.39 (s, 2H), 3.84 (s, 3H). LC/MS(+ESI): Found *m/z* 294.0. Calcd for $C_{13}H_{11}NO_5S$ $[M+H]^+$ 294.0.

***(Z)*-2-(5-(4-Ethylbenzylidene)-2,4-dioxothiazolidin-3-yl)acetic acid (20d)** was prepared by the method described for **11**. Yield 89%, colorless crystals. ^{1}H NMR (500 MHz, DMSO-*d6*) δ = 7.96 (s, 1H), 7.57 (d, *J* = 8.3, 2H), 7.40 (d, *J* = 8.3, 2H), 4.38 (s, 2H), 2.66 (q, *J* = 7.3, 2H), 1.19 (t, *J* = 7.3, 3H). LC/MS(+ESI): Found *m/z* 292.1. Calcd for $C_{14}H_{13}NO_4S$ $[M+H]^+$ 292.1.

***(Z)*-2-(5-(4-Isopropylbenzylidene)-2,4-dioxothiazolidin-3-yl)acetic acid (20e)** was prepared by the method described for **11**. Yield 92%, colorless crystals. ^{1}H NMR (500 MHz, DMSO-*d6*): δ = 7.96 (s, 1H), 7.58 (d, 2H, J = 7.82), 7.44 (d, 2H, J = 7.82), 4.37 (s, 2H), 3.37 (bs, 1H), 2.95 (m, 1H), 1.22 (m, 6H). LC/MS(+ESI): Found *m/z* 306.1. Calcd for $C_{15}H_{15}NO_4S$ $[M+H]^+$ 306.1.

***(Z)*-2-(5-(4-*tert*-Butylbenzylidene)-2,4-dioxothiazolidin-3-yl)acetic acid (20f)** was prepared by the method described for **11**. Yield 92%, colorless crystals. ^{1}H NMR (500 MHz, DMSO-*d6*): δ = 7.95 (s, 1H), 7.58 (m, 4H), 4.37 (s, 2H), 3.39 (bs, 1H), 1.30 (m, 9H). LC/MS(+ESI): Found *m/z* 320.1. Calcd for $C_{16}H_{17}NO_4S$ $[M+H]^+$ 320.1.

***(Z)*-2-(5-(2,4-dichlorobenzylidene)-2,4-dioxothiazolidin-3-yl)acetic acid (20g)** was prepared by the method described for **11**. Yield 87%, white crystal. ^{1}H NMR (400 MHz, DMSO-*d6*): 13.46 (1H, br), 8.03 (1H, s), 7.88 (1H, s), 7.65 (2H, s), 4.41 (2H, s). ^{13}C NMR (400 MHz, DMSO-*d6*) δ: 168.26, 166.85, 164.97, 136.38, 135.91, 130.67, 130.43, 128.84, 128.29, 125.49, 42.88. HRMS (EI): Calcd for $C_{12}H_7Cl_2NO_4S$ $[M+H]^+$ 333.1585. Found: *m/z* 333.1608.

***(Z)*-2-(5-(4-Chlorobenzylidene)-2,4-dioxothiazolidin-3-yl)propionic acid (21a)** was prepared by the method described for **11**. Yield 59%, light yellow crystals. ^{1}H NMR (400 MHz, DMSO-*d6*) δ 13.27 (s, 1H), 7.93 (s, 1H), 7.62 (d, *J* = 8.6, 2H), 7.57 (d, *J* = 8.7, 2H), 5.00 (q, *J* = 7.1, 1H), 1.50 (d, *J* = 7.2, 3H). ^{13}C NMR (101 MHz, DMSO-*d6*) δ 170.44, 166.86, 165.20, 135.87, 132.87, 132.24(2C), 132.14, 129.88(2C), 121.79, 50.85, 14.38. LC/MS(+ESI): Found *m/z* 334.0. Calcd for $C_{13}H_{10}ClNO_4S$ $[M+Na]^+$ 334.0.

***(Z)*-4-(5-(4-chlorobenzylidene)-2,4-dioxothiazolidin-3-yl)butanoic acid (22a)** was prepared by the method described for **11**. Yield 71%, white crystals. ^{1}H NMR (500 MHz, DMSO-*d6*) δ = 7.87 (s, 1H), 7.61 (d, *J* = 8.6, 2H), 7.58 (d, *J* = 8.6, 2H), 3.67 (t, *J* = 6.8, 2H), 2.25 (t, *J* = 7.0, 2H), 1.79 (m, 2H). LC/MS(+ESI): Found *m/z* 348.0. Calcd for $C_{14}H_{12}ClNO_4S$ $[M+H]^+$ 348.0.

Synthesis of amides

A solution of the carboxylic acid (6.6 mmol) in CH_2Cl_2 (20 mL) was suspended in $SOCl_2$ (10 eq.) followed by DMF (2 drops) and the reaction stirred for 2 h. The solution was then concentrated *in vacuo* and the crude acyl chloride used immediately in the following step. A solution of Boc-piperazine (4.9 mmol, 0.75 eq.) and Et_3N (10 mmol, 1.5 eq.) in THF (30 mL) was cooled to 0 °C and the acyl chloride solution in THF (5 mL) was added dropwise keeping the temperature at 3-5 °C. The reaction mixture was allowed to warm to room temperature and stirred for 10 h. The RM was filtered, the filtrate concentrated *in vacuo*, and the residue was purified by flash column chromatography on silica gel.

***tert*-Butyl 4-(2-(2,4-dioxothiazolidin-3-yl)acetyl)piperazine-1-carboxylate (36)** was obtained as described above from **11** and 1-Boc-piperazine. Yield 54%, white crystals. ^1H NMR (400 MHz, DMSO-*d*6) δ 4.42 (s, 2H), 4.28 (s, 2H), 3.62 – 3.19 (m, 8H), 1.40 (s, 9H). ^{13}C NMR (101 MHz, DMSO-*d*6) δ 172.26, 171.96, 163.92, 154.22, 79.67, 44.26, 42.73, 41.84, 34.29, 28.49. LC/MS(+ESI): Found *m/z* 344.1. Calcd for $C_{14}H_{21}N_3O_5S$ $[M+H]^+$ 344.1.

***tert*-Butyl 4-(2-(2,4-Dichlorophenyl)-2-(2,4-dioxothiazolidin-3-yl)acetyl)-piperazine-1-carboxylate (37)** was obtained as described above from **15** and 1-Boc-piperazine. Yield 57%, white crystals. ^1H NMR (500 MHz, DMSO-*d*6) δ 7.77 (s, 1H), 7.55-7.44 (m, 2H), 6.24 (s, 1H), 4.45 – 4.35 (m, 2H), 3.68 (m, 1H), 3.51 (m, 1H), 3.47-3.18 (m, 4H), 3.12-2.94 (m, 2H), 1.44 (s, 9H). LC/MS(+ESI): Found *m/z* 488.1. Calcd for $C_{20}H_{23}Cl_2N_3O_5S$ $[M+H]^+$ 488.1.

3-(2-Oxo-2-(piperazin-1-yl)ethyl)thiazolidine-2,4-dione trifluoroacetate (**38**) was obtained from **36** by hydrolysis in 1:1 CH_2Cl_2:TFA mixture at room temperature for 8 h. The solvent was removed under reduced pressure and the residue was dried under vacuum overnight to obtain 38 as colorless crystals (94%). ^1H NMR (400 MHz, DMSO-*d*6) δ 9.62 (s, 2H), 4.49 (s, 2H), 4.30 (s, 2H), 3.76 (s, 3H), 3.65 (s, 2H), 3.12 (s, 2H), 3.03 (s, 2H). ^{13}C NMR (101 MHz, DMSO-*d*6) δ 172.37, 172.04, 164.19, 42.69, 42.56, 41.46, 38.68. LC/MS(+ESI): Found *m/z* 244.1. Calcd for $C_9H_{13}N_3O_3S$ $[M+H]^+$ 244.1.

3-(1-(2,4-Dichlorophenyl)-2-oxo-2-(piperazin-1-yl)ethyl)thiazolidine-2,4-dione hydrochloride (**39**) was obtained from **37** by hydrolysis the same method as described for **38**. Yield 94%, white crystals. ^1H NMR (500 MHz, DMSO-*d*6) δ 9.44 (bs, 2H), 7.73 (s, 1H), 7.48 (m, 2H), 6.23 (s, 1H), 4.45 – 4.35 (m, 2H), 3.80 (m. 2H), 3.45 (m, 1H), 3.27-3.06 (m, 3H), 2.98 (m. 1H). 2.78 (m, 1H). LC/MS(+ESI): Found *m/z* 388.0. Calcd for $C_{15}H_{15}Cl_2N_3O_3S$ $[M+H]^+$ 388.0.

tert*-Butyl *(Z)*-4-(2-(5-(4-Chlorobenzylidene)-2,4-dioxothiazolidin-3-yl)acetyl)-piperazine-1-carboxylate (40a)** ***<u>(Method 1):</u> prepared as described above from **22a** and 1-Boc-piperazine. Yield 68%.

<u>(Method 2):</u> A mixture of thiazolidinedione **36** (0.571 mmol), an appropriate benzaldehyde (0.571 mmol) and piperidine (0.045 mL, 0.457 mmol) was refluxed in ethanol (6 mL) for 24 h. The reaction was poured into water and acidified with acetic acid to form a precipitate which was collected by vacuum filtration. Yield 54%.

White crystals. ^1H NMR (400 MHz, DMSO-*d*6) δ 7.94 (s, 1H), 7.72 – 7.54 (m, 4H), 4.62 (s, 2H), 3.58 – 3.21 (m, 8H), 1.40 (s, 9H). ^{13}C NMR (101 MHz, DMSO-*d*6) δ 167.25, 165.60, 163.78, 154.17, 135.84, 132.66, 132.27, 132.17, 132.09, 129.91, 129.84, 122.13, 79.69, 44.27, 43.19, 41.87, 28.46, 25.92. LC/MS(+ESI): Found *m/z* 466.1. Calcd for $C_{21}H_{24}ClN_3O_5S$ $[M+H]^+$ 466.1.

***tert*-Butyl *(Z)*-4-(2-(5-(4-chlorobenzylidene)-2,4-dioxothiazolidin-3-l)propanoyl)-piperazine-1-carboxylate (41a)** was obtained as described above from

21a and Boc-piperazine. Yield 58%, yellow crystals. ^{1}H NMR (700 MHz, DMSO-*d*$_6$) δ 7.85 (s, 1H), 7.57 (d, *J* = 8.6, 2H), 7.53 (d, *J* = 8.6, 2H), 5.19 (q, *J* = 7.1, 1H), 3.34 – 3.25 (m, 4H), 3.24 – 3.16 (m, 4H), 1.47 (d, *J* = 7.1, 3H), 1.31 (s, 9H). ^{13}C NMR (176 MHz, DMSO-*d*$_6$) δ 166.97, 166.93, 165.54, 154.17, 135.83, 132.63, 132.31(2C), 132.27, 129.94(2C), 121.73, 79.69, 51.01, 45.12, 42.38, 28.46(3C), 15.03. LC/MS(+ESI): Found *m/z* 480.1. Calcd for $C_{22}H_{26}ClN_3O_5S$ $[M+H]^+$ 480.1.

***tert*-Butyl *(Z)*-4-(4-(5-(4-Chlorobenzylidene)-2,4-dioxothiazolidin-3-yl)butanoyl)-piperazine-1-carboxylate (42a)** was obtained as described above from **22a** and Boc-piperazine. Yield 41%.

(Alternative method* with *CDI) To a solution of **22a** (12.3 mmol) and CDI (13.0 mmol, 1.1 eq.) in 1:3 v/v DMF/CH_2Cl_2 (40 mL), Et_3N (24.7 mmol, 2 eq.) was added and the reaction was stirred for 20 min. After that, 1-Boc-piperazine (13.6 mmol, 1.2 eq.) was added and the reaction was stirred for 12 h. The reaction mixture was then diluted with EtOAc (150 mL), washed with 1M aq. HCl (100 mL), 1M aq. NaOH (100 mL) and brine (150 mL), dried ($MgSO_4$) and concentrated in vacuo to obtain the crude product. The resulting residue was purified by flash chromatography on silica gel, yielding the desired product. Yield 39%.

White crystals. ^{1}H NMR (400 MHz, DMSO-*d*$_6$) δ 7.88 (s, 1H), 7.62 (d, *J* = 8.5, 2H), 7.58 (d, *J* = 8.5, 2H), 3.66 (t, *J* = 6.5, 2H), 3.42 – 3.20 (m, 8H), 2.36 (t, *J* = 6.7, 2H), 1.87 – 1.75 (m, 2H), 1.38 (s, 9H). ^{13}C NMR (101 MHz, DMSO-*d*$_6$) δ 170.26, 167.67, 166.14, 154.24, 135.58, 132.36, 132.14, 131.63, 129.88, 122.78, 79.55, 44.93, 41.88, 41.30, 29.81, 28.46, 22.94. LC/MS(+ESI): Found *m/z* 494.2. Calcd for $C_{23}H_{28}ClN_3O_5S$ $[M+H]^+$ 494.2.

***tert*-Butyl *(Z)*-4-(2-(5-(4-chlorobenzylidene)-2,4-dioxothiazolidin-3-yl)-2-(2,4-dichlorophenyl)acetyl)piperazine-1-carboxylate (43a)** was obtained from **37** and 4-chlorobenzaldehyde. Yield 65%, white crystals. ^{1}H NMR (500 MHz, DMSO-*d*$_6$) δ = 7.96 (s, 1H), 7.75 (s, 1H), 7.67 (d, *J* = 8.5, 2H), 7.63 (d, *J* = 8.5, 2H), 7.50 (m, 2H), 6.39 (s, 1H), 3.65 (m, 1H), 3.53 (m, 1H), 3.46-3.17 (m, 4H), 3.11-2.89 (m, 2H), 1.40 (s, 9H). LC/MS(+ESI): Found *m/z* 610.1. Calcd for $C_{27}H_{26}Cl_3N_3O_5S$ $[M+H]^+$ 610.1.

***tert*-Butyl *(Z)*-4-(2-(2,4-Dichlorophenyl)-2-(5-(4-fluorobenzylidene)-2,4-dioxothiazolidin-3-yl)acetyl)piperazine-1-carboxylate (43b)** was obtained from **37** and 4-fluorobenzaldehyde. Yield 51%, white crystals. ^{1}H NMR (500 MHz, DMSO-*d*$_6$) δ = 7.97 (s, 1H), 7.74 (m, 3H), 7.57-7.33 (m, 4H), 6.39 (s, 1H), 3.65 (m, 1H), 3.54 (m, 1H), 3.47-3.13 (m, 4H), 3.10-2.88 (m, 2H), 1.40 (s, 9H). LC/MS(+ESI): Found *m/z* 594.1. Calcd for $C_{27}H_{26}Cl_2FN_3O_5S$ $[M+H]^+$ 594.1.

***(Z)*-5-(4-Chlorobenzylidene)-3-(2-oxo-2-(piperazin-1-yl)ethyl)thiazolidine-2,4-dione (44a)** was obtained from **17a.** Yield 93%, white crystals. ^{1}H NMR (400 MHz, DMSO-*d*$_6$) δ 7.94 (s, 1H), 7.65 (d, *J* = 8.5, 2H), 7.60 (d, *J* = 8.5, 2H), 4.59 (s, 2H), 3.54 – 3.32 (m, 5H), 2.79 (s, 2H), 2.71 (s, 2H). ^{13}C NMR (101 MHz, DMSO-*d*$_6$) δ 167.25, 165.62, 163.47, 135.83, 132.61, 132.25, 132.18, 122.18, 45.59, 45.19, 43.17, 42.56. LC/MS(+ESI): Found *m/z* 366.1. Calcd for $C_{16}H_{16}ClN_3O_3S$ $[M+H]^+$ 366.1.

***(Z)*-5-(4-Chlorobenzylidene)-3-(1-oxo-1-(piperazin-1-yl)propan-2-yl)thiazolidine-2,4-dione (45a)** was obtained from **41a**. Yield 91%, white crystals. ^{1}H NMR (700 MHz, DMSO-*d*$_6$) δ 7.94 (s, 1H), 7.66 (d, *J* = 8.6, 2H), 7.62 (d, *J* = 8.6, 2H), 5.23 (q, *J* = 7.1, 1H), 3.39 – 3.24 (m, 4H), 2.72 – 2.54 (m, 4H), 1.53 (d, *J* = 7.1, 3H). ^{13}C NMR (176 MHz, DMSO-*d*$_6$) δ 166.91, 166.49, 165.55, 135.84, 132.62, 132.32(2C), 132.25, 129.95(2C), 121.70, 50.93, 46.95, 46.20, 45.93, 43.88, 15.04. LC/MS(+ESI): Found *m/z* 380.1. Calcd for $C_{17}H_{18}ClN_3O_3S$ $[M+H]^+$ 380.1.

***(Z)*-5-(4-Chlorobenzylidene)-3-(4-oxo-4-(piperazin-1-yl)butyl)thiazolidine-2,4-dione (46a)** was obtained from **42a.** Yield 94%, white crystals. ^{1}H NMR (400 MHz, DMSO-*d*$_6$) δ 7.88 (s, 1H), 7.63 (d, *J* = 8.8, 2H), 7.59 (d, *J* = 8.7, 2H), 3.66 (t, *J* = 6.7,

2H), 2.95 (br.s, 2H), 2.89 (br. s, 2H), 2.37 (t, *J* = 6.9, 2H), 1.87 – 1.71 (m, 2H). ^{13}C NMR (101 MHz, DMSO-d_6) δ 170.30, 167.71, 166.15, 135.60, 132.35, 132.14, 131.64, 129.90, 122.79, 62.45, 44.00, 43.86, 43.43, 41.84, 29.53, 25.91, 22.87. LC/MS(+ESI): Found *m/z* 394.1. Calcd for $C_{18}H_{20}ClN_3O_3S$ [M+H]$^+$ 394.1.

***(Z)*-5-(4-Chlorobenzylidene)-3-(1-(2,4-dichlorophenyl)-2-oxo-2-(piperazin-1-yl)ethyl)thiazolidine-2,4-dione hydrochloride** (**47a**) was obtained as previously described from **43a**. Yield 98%, white crystals. ^{1}H NMR (500 MHz, DMSO-d_6) δ = 9.29 (br, 2H), 7.96 (s, 1H), 7.80-7.43 (m, 7H), 6.46 (s, 1H), 3.83 (m. 2H), 3.49 (m, 1H), 3.43-3.08 (m, 3H), 3.02 (m, 1H), 2.81 (m, 1H). LC/MS(+ESI): Found *m/z* 510.0. Calcd for $C_{22}H_{18}Cl_3N_3O_3S$ [M+H]$^+$ 510.0.

***(Z)*-3-(1-(2,4-Dichlorophenyl)-2-oxo-2-(piperazin-1-yl)ethyl)-5-(4-fluoro-benzylidene)thiazolidine-2,4-dione hydrochloride (47b)** was obtained from **43b.** ^{1}H NMR (500 MHz, DMSO-d_6) δ = 9.30 (br, 2H), 7.97 (s, 1H), 7.72 (m, 3H), 7.61-7.35 (m. 4H), 6.45 (s, 1H), 3.83 (m. 2H), 3.49 (m, 1H), 3.45-3.07 (m, 3H), 3.02 (m. 1H). 2.81 (m, 1H). LC/MS(+ESI): Found *m/z* 494.0. Calcd for $C_{22}H_{18}Cl_2FN_3O_3S$ [M+H]$^+$ 494.0.

References

1. Maccari, R.; Ottana, R.; Curinga, C.; Vigorita, M. G.; Rakowitz, D.; Steindl, T.; Langer T. Structure–Activity Relationships and Molecular Modelling of 5-Arylidene-2,4-Thiazolidinediones Active as Aldose Reductase Inhibitors. *Bioorg. Med. Chem*., **2005**, 13, 2809–2823. doi:10.1016/j.bmc.2005.02.026

2. Whiting, E.; Raje, M.R.; Chauhan, J.; Wilder, P.T.; Van Eker, D.; Hughes, S.J.; Bowen, N.G.; Vickers, G.E.A.; Fenimore, I.C.; Fletcher, S. Discovery of Mcl-1 inhibitors based on a thiazolidine-2,4-dione scaffold. *Bioorg. Med. Chem. Lett.* **2018**, 28, 3, 523-528. https://doi.org/10.1016/j.bmcl.2017.11.023

3. Yuli Xie, Yidong Liu, Gangli Gong, Alison Rinderspacher, Shi-Xian Deng, Deborah H. Smith, Udo Toebben, Effie Tzilianos, Lars Branden, Dusˇica Vidovic, Caty Chung, Stephan Schu¨rer, Lutz Tautz, Donald W. Landry. Discovery of a novel submicromolar inhibitor of the lymphoid specific tyrosine phosphatase. *Bioorg. Med. Chem. Lett.* **2008**, 18, 2840–2844.

4. Levshin, I.B.; Sandulenko, Y.B.; Polyakova, M.V.; Grammatikova, N. E.; Vasileva, N.V.; Bogomolova, T.S. RU Patent 2703997C1. WO2020122766A1. Hybrid amides based on triazole and thiazolidine having antimicrobial activity. 13 December 2018.

5. Christoffa, R.M.; Soares da Costab, T.P.; Bayata, S.; Holienc, J.K.; Matthew A.; Peruginib, M.A.; Abbotta, B.A. Synthesis and Structure-Activity Relationship Studies of 2,4-Thiazolidinediones and Analogous Heterocycles as Inhibitors of Dihydrodipicolinate Synthase. *Bioorg. Med. Chem.* **2021,** 52, 116518. doi: 10.1016/j.bmc.2021.116518

6. Levshin, I.B.; Tsurkan, A.A.; V'yunov, K.A.; Ginak, A.I. J. Appl. Chem. USSR (Engl. Transl.). **1983**, 56, 7, 1453 – 1455.

7. Fu, X.; Mao, Q.; Zhang, B.; *et al.* Thiazolidinedione-Based Structure Modification of Celastrol Provides Thiazolidinedione-Conjugated Derivatives as Potent Agents against Non-Small-Cell Lung Cancer Cells through a Mitochondria-Mediated Apoptotic Pathway. *J. Nat. Prod.* **2022,** 85, 4, 1147-1156. Doi: 10.1021/acs.jnatprod.2c00104

8. Abd Alhameed, R.; Almarhoon, Z.; Bukhari, S.I.; El-Faham, A.; de la Torre, B.G.; Albericio, F. Synthesis and Antimicrobial Activity of a New Series of Thiazolidine-

2,4-diones Carboxamide and Amino Acid Derivatives. *Molecules,* **2020,** 25, 105. doi:10.3390/molecules25010105
9. Wang,Y.; Xu, K.; Bai, G.; Huang,L.; Wu, Q.; Pan, W.; Yu, Sh. Synthesis and Antifungal Activity of Novel Triazole Compounds Containing Piperazine Moiety. *Molecules,* **2014**, 19, 11333-11340; doi:10.3390/molecules190811333

2. MOLECULAR MODELING

Figure S1. Binding modes of synthesized ligands in the active site of *C. albicans* CYP51 as revealed by docking. Superimposition of docked poses of **31a** (magenta), **50a** (cyan), **33a** (blue), and **34a** (yellow) is shown. Hydrogen bonds are shown by dashed yellow lines; heme and selected residues of receptor are depicted by sticks. Fragment of the binding site molecular surface is shown.

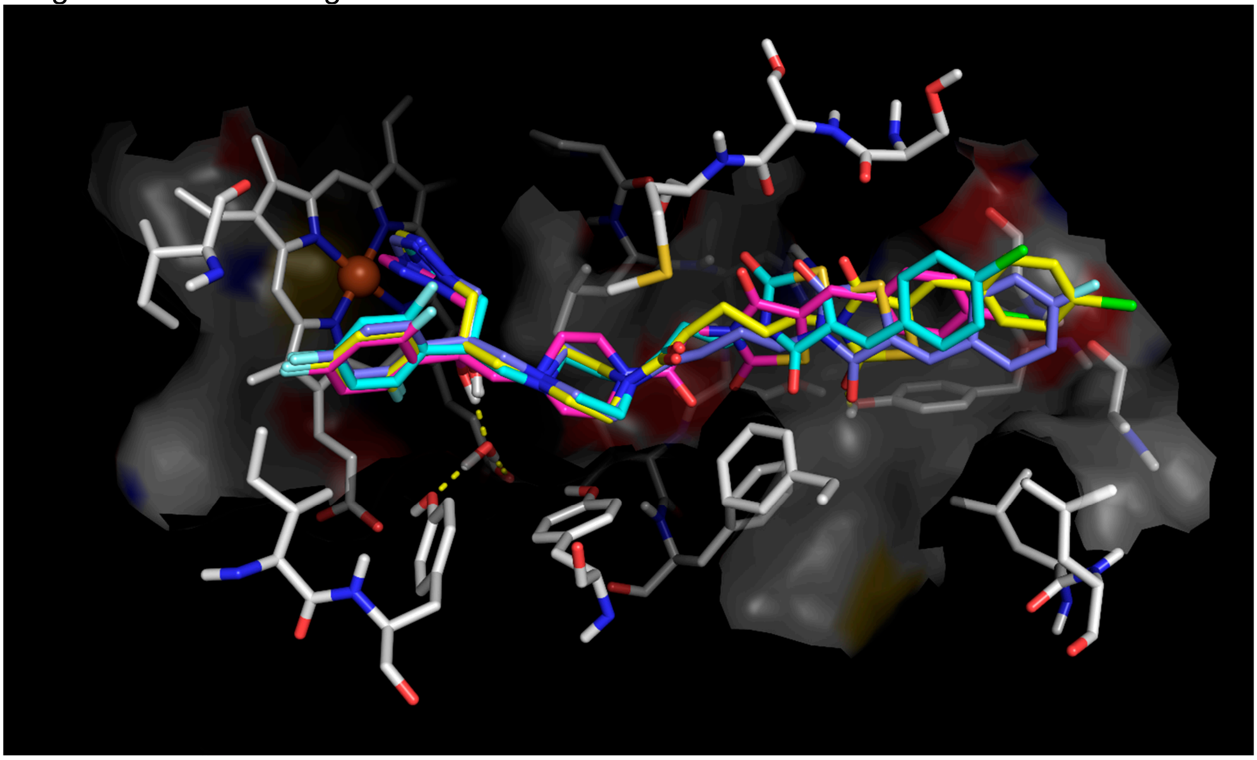

Figure S2. Alignment of CYP51 amino acid sequence for *Candida albicans* SC5314 (Query) vs *Candida parapsilosis* ATCC 22019 (Subject).

```
Query  1    MAKKTPPLVFYWIPWFGSAASYGQQPYEFFESCRQKYGDVFSFMLLGKIMTVYLGPKGHE  60
            + K   PLVFYWIPW GSA SYGQ PY FFE CR+KYGD+FSF++LG++MTVYLGPKGHE
Sbjct  43   LRKDRVPLVFYWIPWVGSAVSYGQDPYGFFEQCREKYGDLFSFVMLGRVMTVYLGPKGHE  102

Query  61   FVFNAKLSDVSAEEAYKHLTTPVFGTGVIYDCPNSRLMEQKKFAKFALTTDSFKRYVPKI  120
            FVFNAKLSDVSAE+AY+HLTTPVFG GVIYDCPN+RLMEQKKFAK ALTTDSF+RYVP I
Sbjct  103  FVFNAKLSDVSAEDAYQHLTTPVFGKGVIYDCPNARLMEQKKFAKTALTTDSFRRYVPLI  162

Query  121  REEILNYFVTDESFKLKEKTHGVANVMKTQPEITIFTASRSLFGDEMRRIFDRSFAQLYS  180
            R EIL+YF   + F +K++  GV +V+++QPEITIFTASRSL G+ MR+ FD SFAQLY+
Sbjct  163  RGEILDYFTKSKVFNMKKQKSGVVDVLQSQPEITIFTASRSLLGEAMRKRFDASFAQLYA  222

Query  181  DLDKGFTPINFVFPNLPLPHYWRRDAAQKKISATYMKEIKLRRERGDIDPNRDLIDSLLI  240
            DLDKGFTPINFVFP+LPLPHYW+RDAAQ+KIS TYM EI  RRE GDID NRDLIDSLL+
Sbjct  223  DLDKGFTPINFVFPHLPLPHYWKRDAAQQKISETYMTEIARRRETGDIDENRDLIDSLLV  282

Query  241  HSTYKDGVKMTDQEIANLLIGILMGGQHTSASTSAWFLLHLGEKPHLQDVIYQEVVELLK  300
            +STYKDGVKMTDQEIANLLIG+LMGGQHTSA+TSAWFLLHL EKP LQD +YQEV+  L
Sbjct  283  NSTYKDGVKMTDQEIANLLIGVLMGGQHTSATTSAWFLLHLAEKPQLQDELYQEVLNALS  342

Query  301  EKGGDLNDLTYEDLQKLPSVNNTIKETLRMHMPLHSIFRKVTNPLRIPETNYIVPKGHYV  360
             KGG+L+DL+YEDLQ++P VNNTIKETLR+HMPLHSIFRKV +PL +P T YIVP+GH+V
Sbjct  343  GKGGNLDDLSYEDLQQMPLVNNTIKETLRLHMPLHSIFRKVVSPLVVPNTKYIVPRGHHV  402

Query  361  LVSPGYAHTSERYFDNPEDFDPTRWDTAAAKANSVSFNSSDEVDYGFGKVSKGVSSPYLP  420
            LVSPGYAHT+ER++ +  DF+P RWD +A      S N + EVDYGFGKVSKGVSS YLP
Sbjct  403  LVSPGYAHTNERFYKDASDFNPHRWDESA------STNDAGEVDYGFGKVSKGVSSSYLP  456

Query  421  FGGGRHRCIGEQFAYVQLGTILTTFVYNLRWTIDGYKVPDPDYSSMVVLPTEPAEIIWEK  480
            FGGGRHRCIGEQFAYVQLGTILTTFVYNL+W +   KVPD DY+SMV LP  PAEI+WEK
Sbjct  457  FGGGRHRCIGEQFAYVQLGTILTTFVYNLKWKLANGKVPDVDYTSMVTLPQHPAEIVWEK  516

Query  481  RETCM  485
            R+TC+
Sbjct  517  RDTCV  521
```

Figure S3. Comparison of homology of the binding site of CYP51 from *C. albicans* with that from *C. parapsilosis* ATCC 22019. Residues within the distance of 6 Å around the ligand VT1161 are shown on crystallographic structure of CYP51 form *C. albicans* (PDB ID 5TZ1). Identical residues are in green. Residues corresponding to three substitutions are in red (I304 > V; L87 > M; S506 > T).

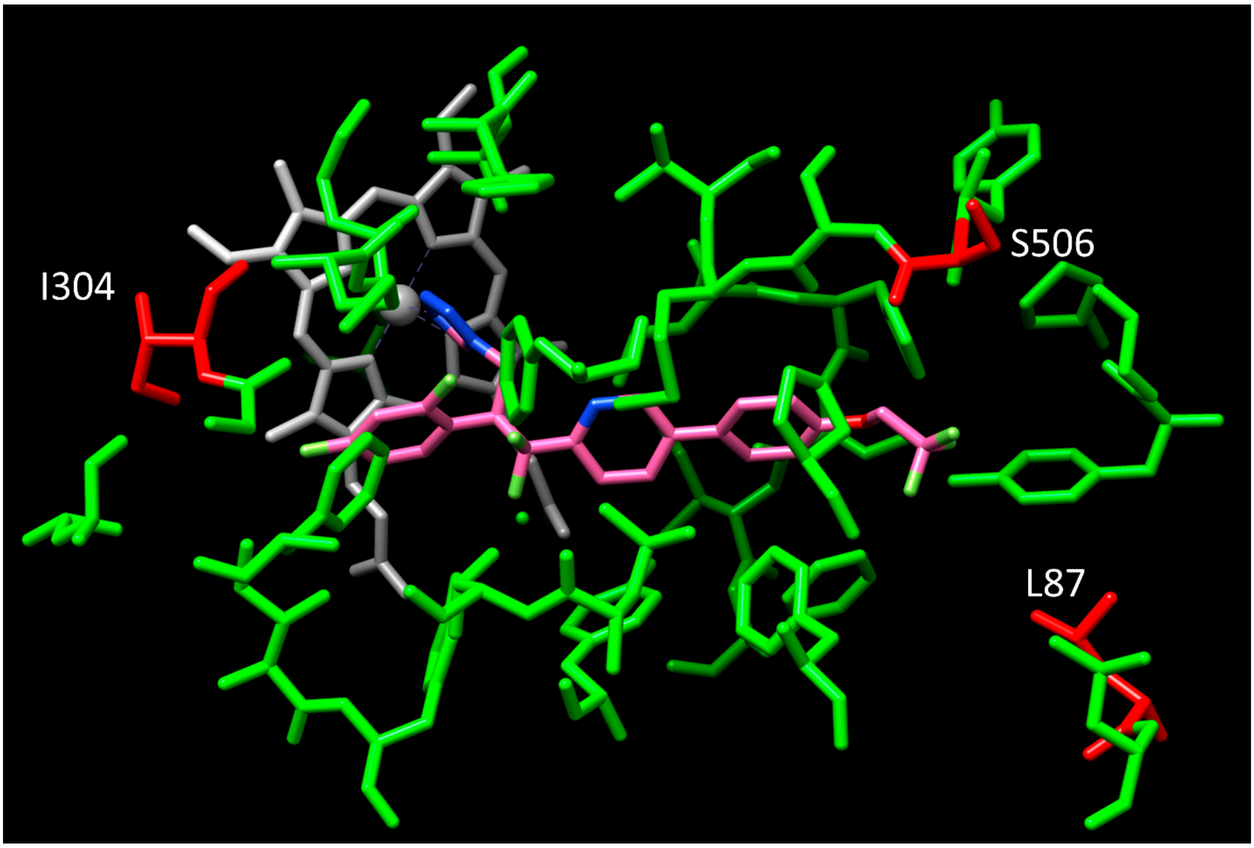

Figure S4. Validation of docking: **a.** Docked ligand VT1161 (yellow) superimposed over its crystallographic conformation (green), **b.** Docked fluconazole (yellow) superimposed over the crystallographic conformation of VT1161 (green).

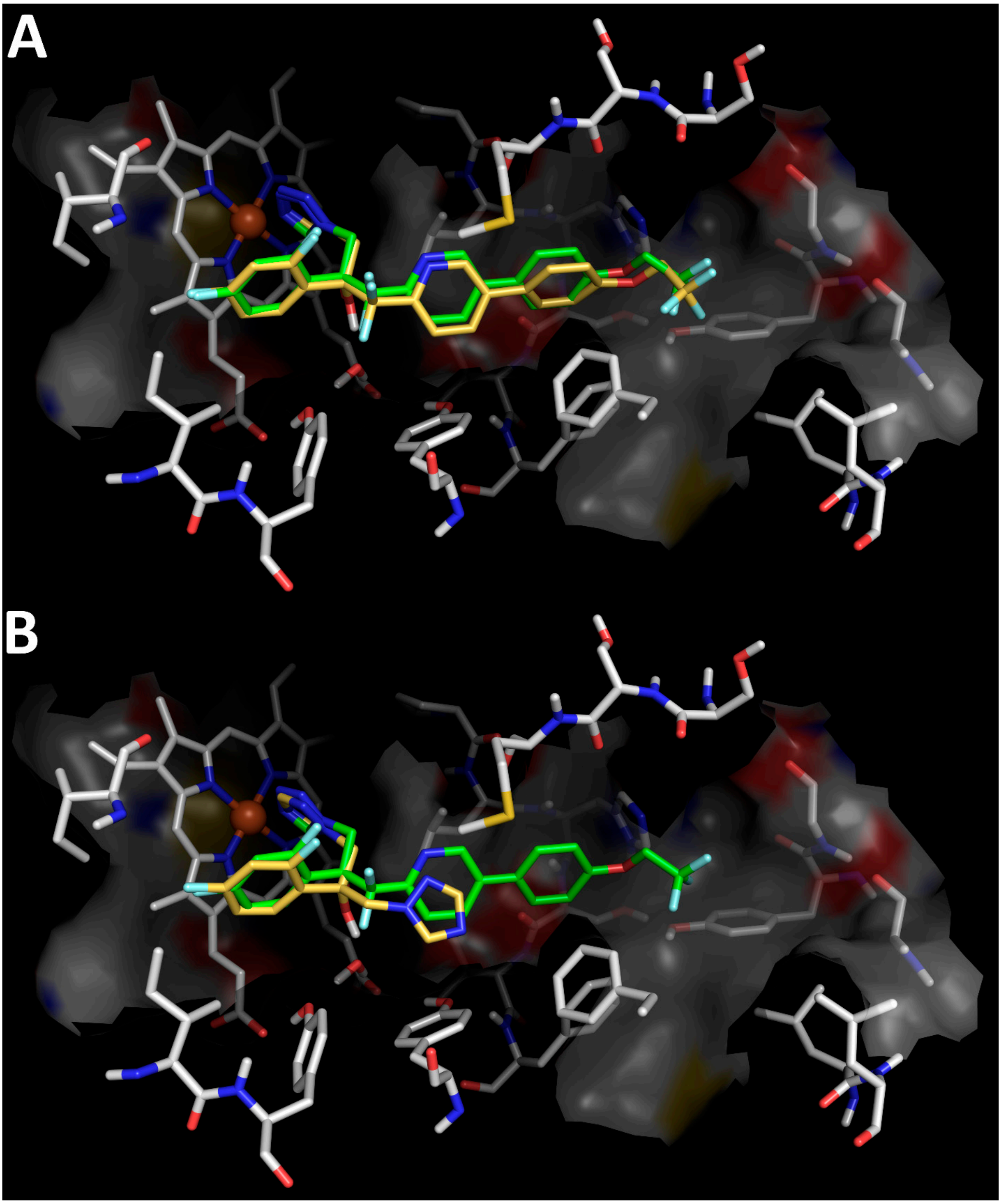

3. NMR SPECTRA

Table S1. ^{1}H, ^{13}C and ^{15}N chemical shifts of **31a** (**L-173**) and **L-310** measured in DMSO-d_6 at 298K and 600 MHz proton resonance frequency.

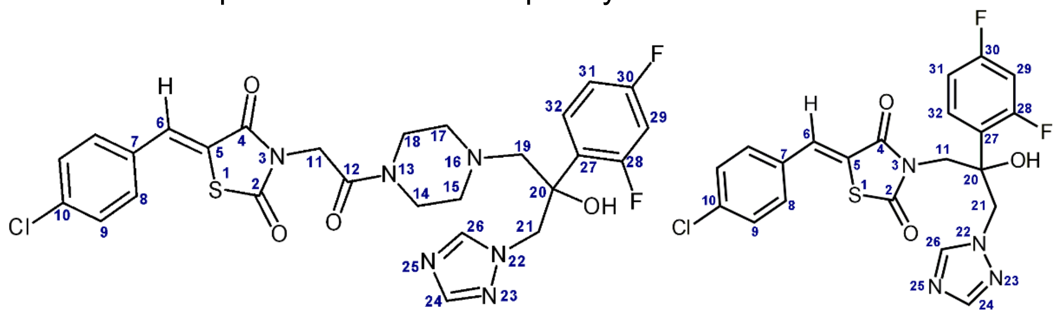

Position	LIB-173		LIB-310		
	δ(^{1}H), ppm	δ(^{13}C), ppm	δ(^{1}H), ppm	δ(^{13}C), ppm	δ(^{15}N), ppm
2	-	166.8	-	167.4	-
3	-	-	-	-	162.6
4	-	165.2	-	166.0	-
5	-	121.7	-	122.0	-
6	7.92	132.1	7.86	131.5	-
7	-	135.4	-	135.3	-
8	7.63	131.8	7.62	131.9	-
9	7.58	129.5	7.58	129.6	-
10	-	131.7	-	132.2	-
11	4.54	42.6	4.11; 4.13	48.1	-
12	-	162.9	-	-	-
13	-	-	-	-	-
14	3.32	41.8	-	-	-
15	2.38; 2.41	53.7	-	-	-
16	-	-	-	-	-
17	2.43; 2.48	54.0	-	-	-
18	3.37	44.1	-	-	-
19	2.71; 2.88	63.4	-	-	-
20	5.74 (OH)	74.7	6.11 (OH)	74.3	-
21	4.58	55.5	4.59; 4.81	55.0	-
22	-	-	-	-	211.6
23	-	-	-	-	298.4
24	7.75	150.5	7.69	150.6	-
25	-	-	-	-	252.3
26	8.29	144.9	8.27	145.0	-
27	-	126.0	-	123.9	-
28	-	158.9	-	159.5	-
29	7.14	103.8	7.17	104.0	-
30	-	161.7	-	162.1	-
31	6.95	110.8	6.87	110.9	-
32	7.41	129.8	7.25	129.7	-

Figure S5. 1D ^{1}H NMR spectrum of **L-310** recorded in DMSO-d_6 at 298K and 600 MHz proton resonance frequency.

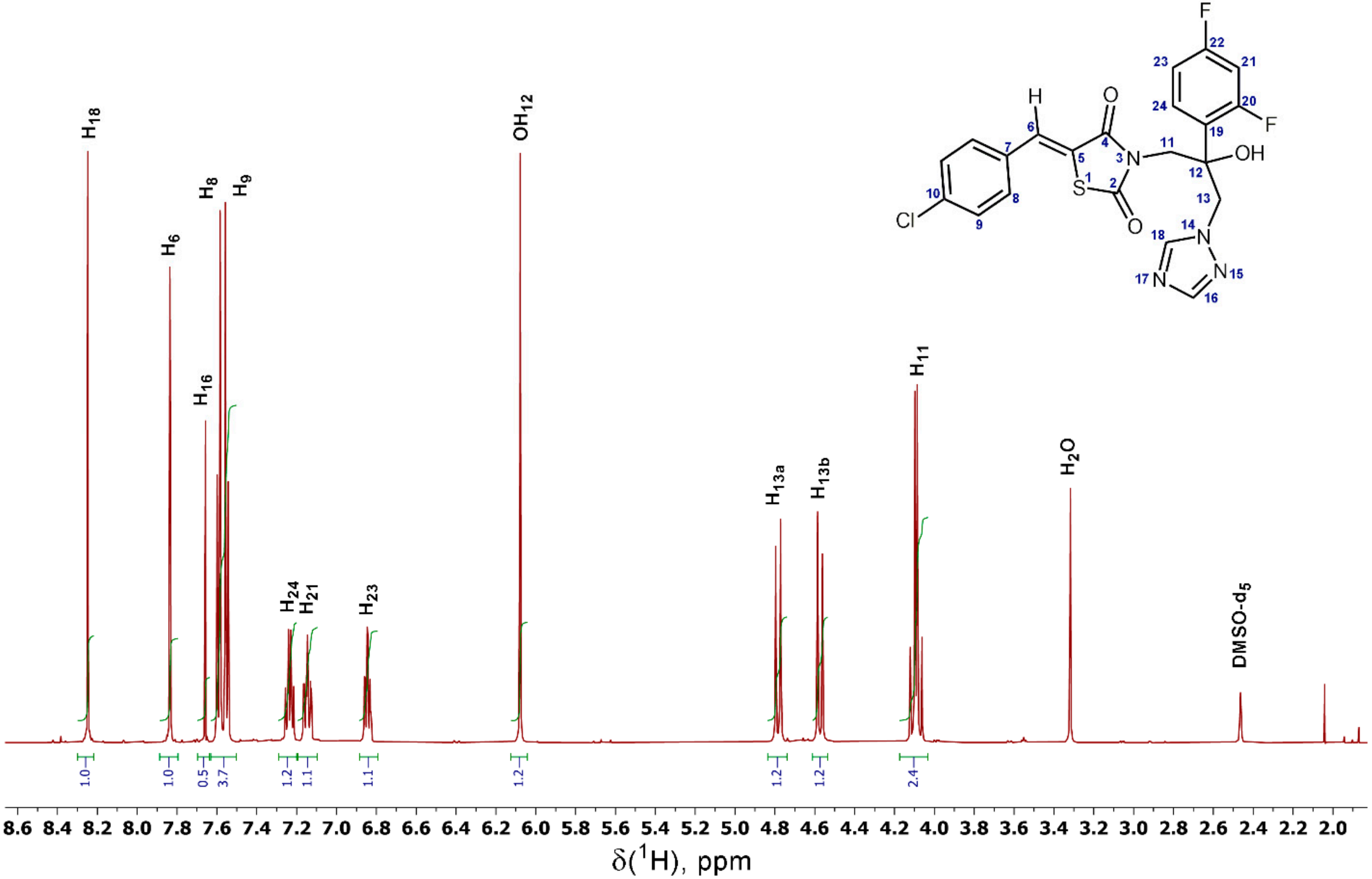

Figure S6. 1D ^{13}C NMR spectrum of **L-310** recorded in DMSO-d_6 at 298K and 150 MHz carbon resonance frequency.

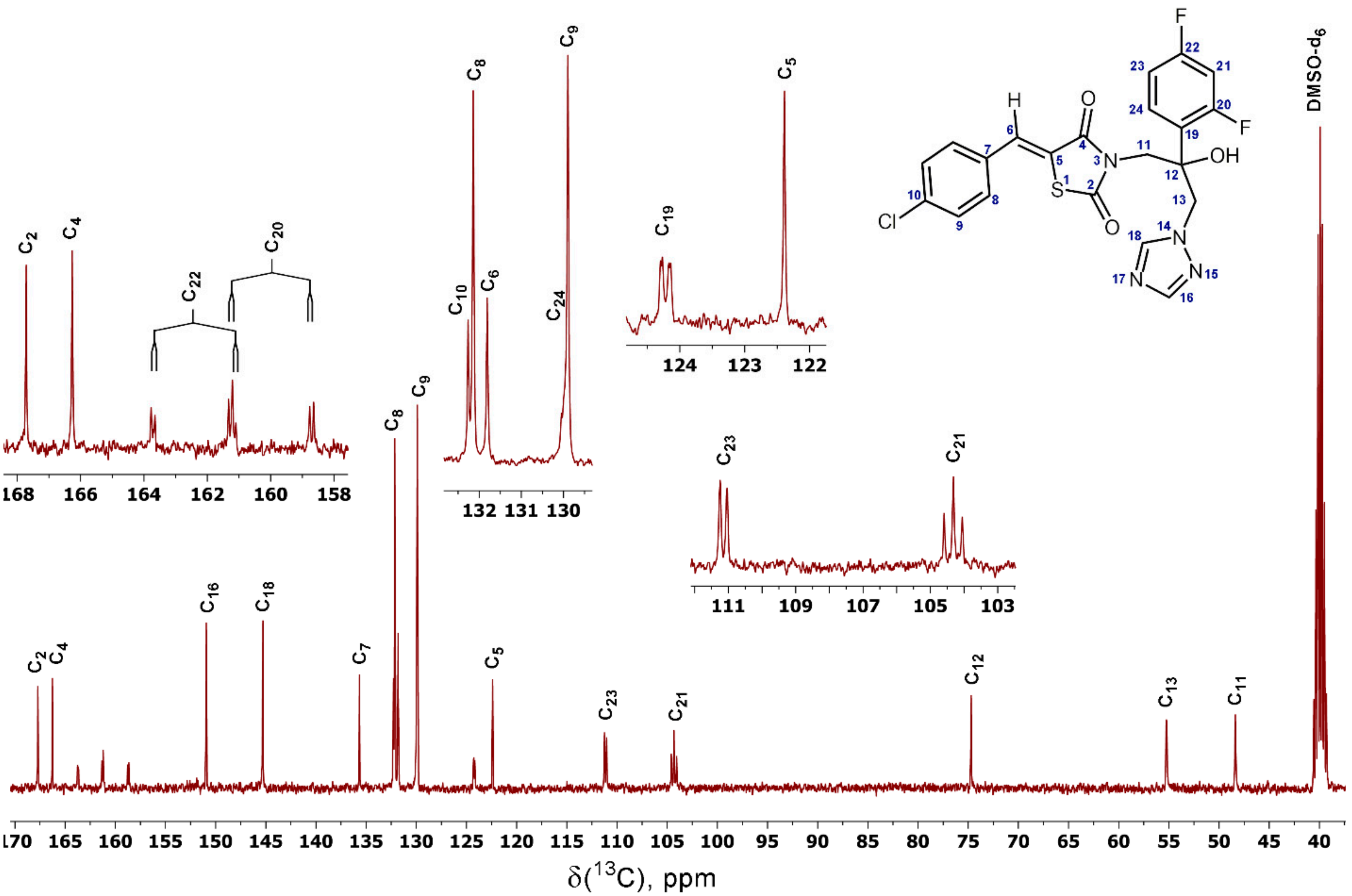

Figure S7. 2D DQF-COSY spectrum of **L-310** recorded in DMSO-d$_6$ at 298K and 600 MHz proton resonance frequency.

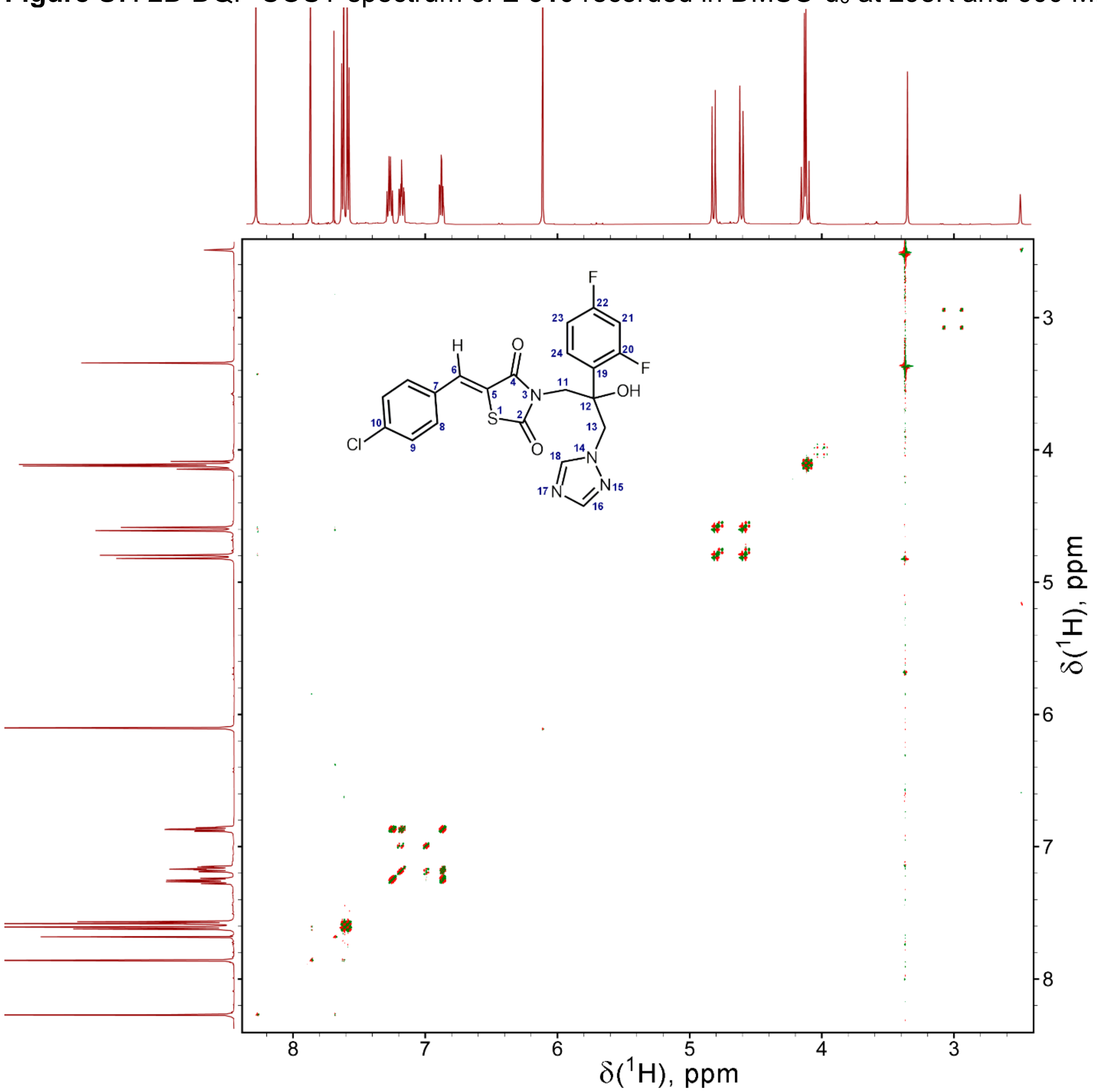

Figure S8. 2D ROESY spectrum of **L-310** recorded in DMSO-d6 at 298K and 600 MHz proton resonance frequency.

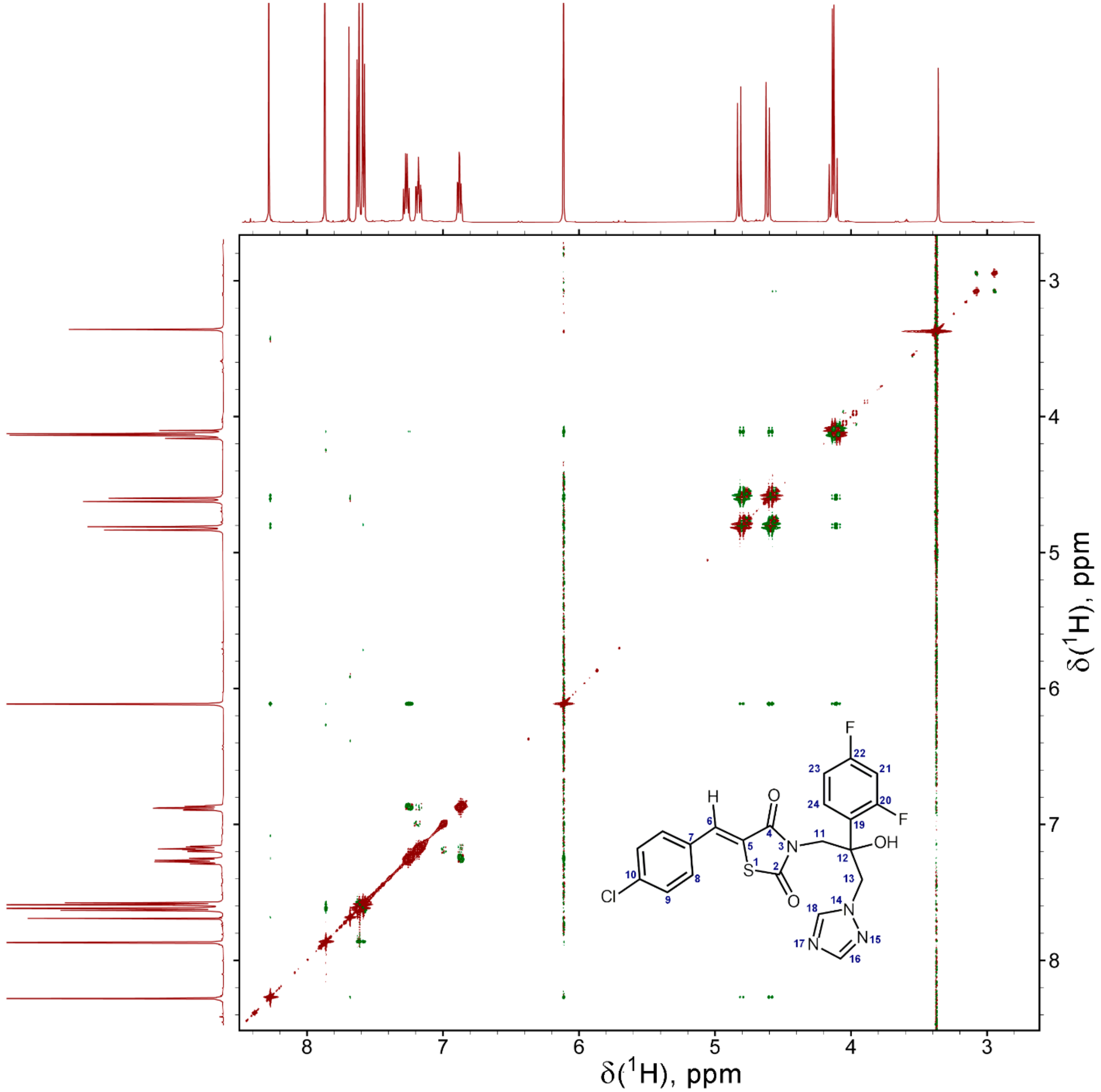

Figure S9. Overlay of 2D spectra ^{13}C-^{1}H HSQC (blue cross-peaks) and ^{13}C-^{1}H HMBC (red cross-peaks) of **L-310** recorded in DMSO-d6 at 298K and 600 MHz proton resonance frequency.

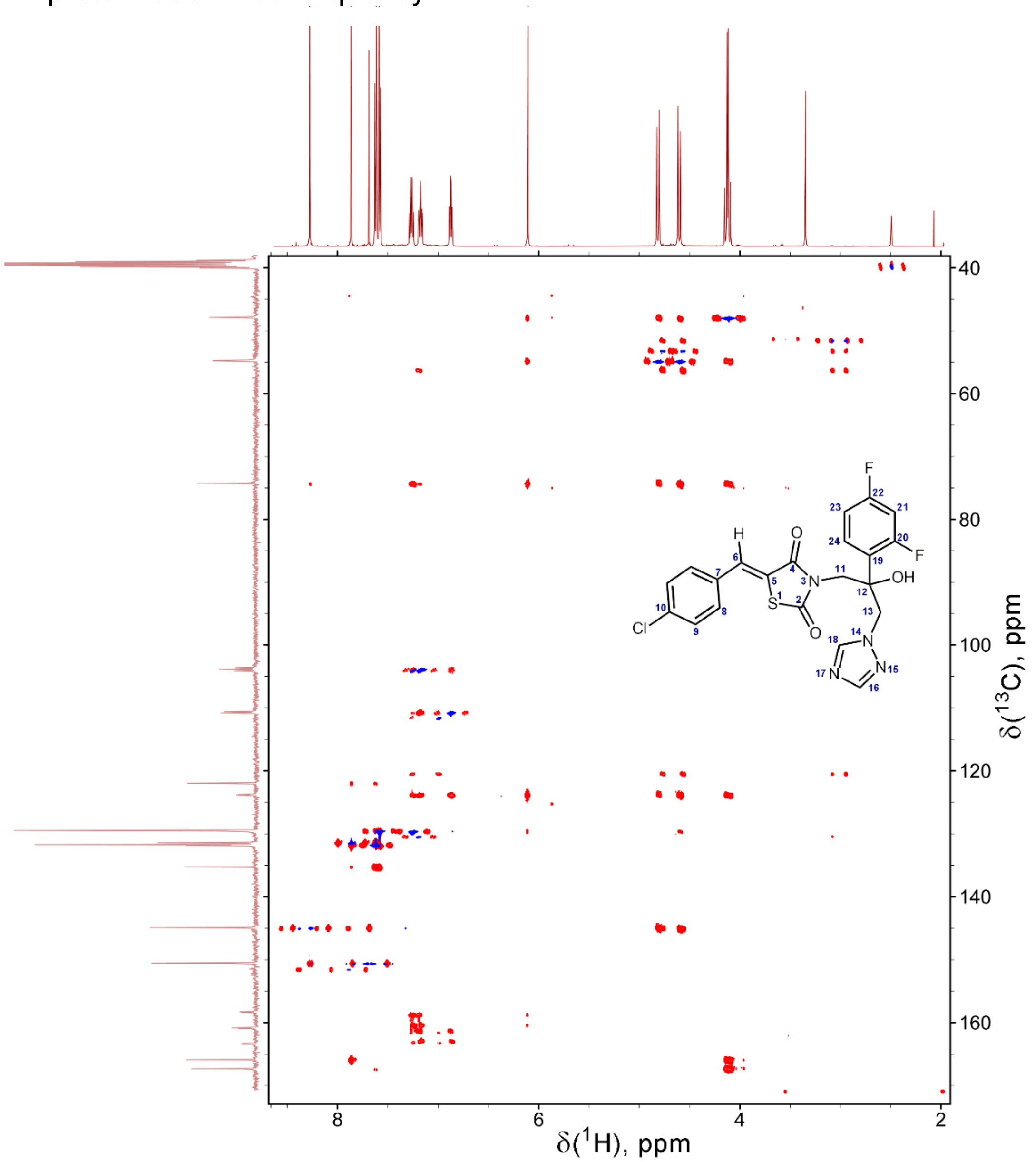

Figure S10. 1D ^{1}H NMR spectrum of **31a** (**L-173**) recorded in DMSO-d$_6$ at 298K and 600 MHz proton resonance frequency.

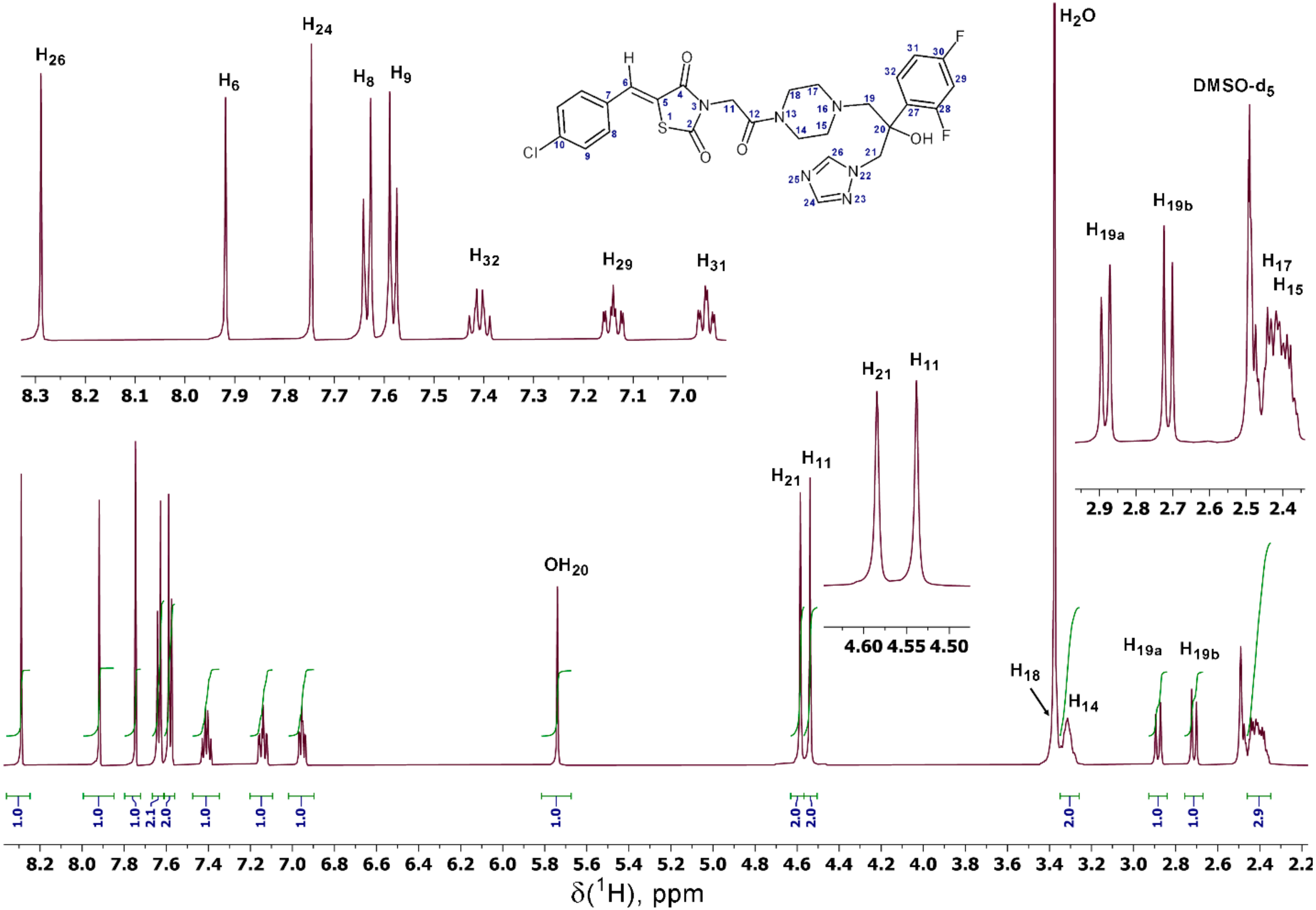

Figure S11. 1D ^{13}C NMR spectrum of **31a** (**L-173**) recorded in DMSO-d_6 at 298K and 150 MHz carbon resonance frequency.

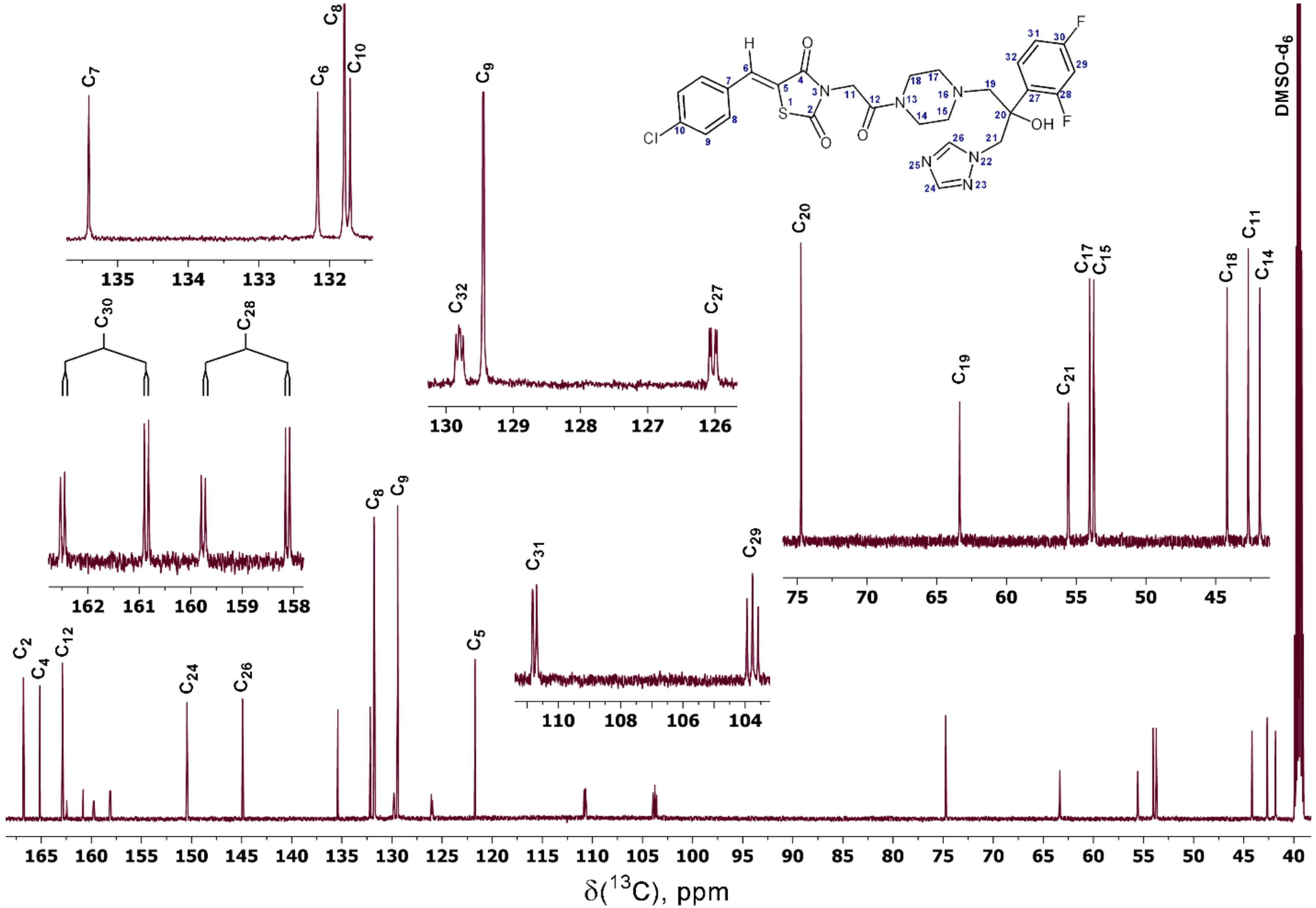

Figure S12. 2D DQF-COSY spectrum of **31a** (**L-173**) recorded in DMSO-d6 at 298K and 600 MHz proton resonance frequency.

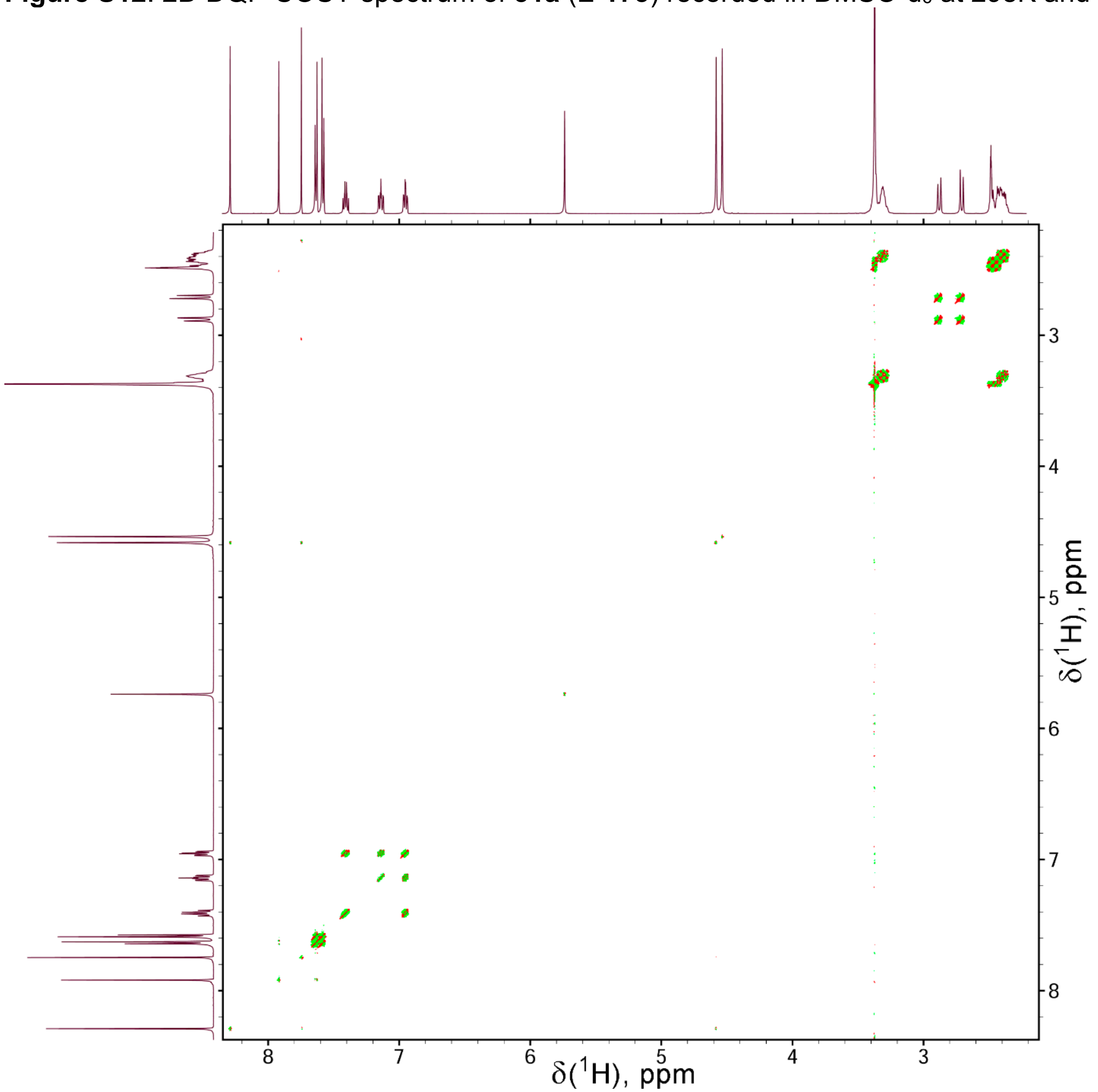

Figure S13. 2D ROESY spectrum of **31a** (**L-173**) recorded in DMSO-d6 at 298K and 600 MHz proton resonance frequency.

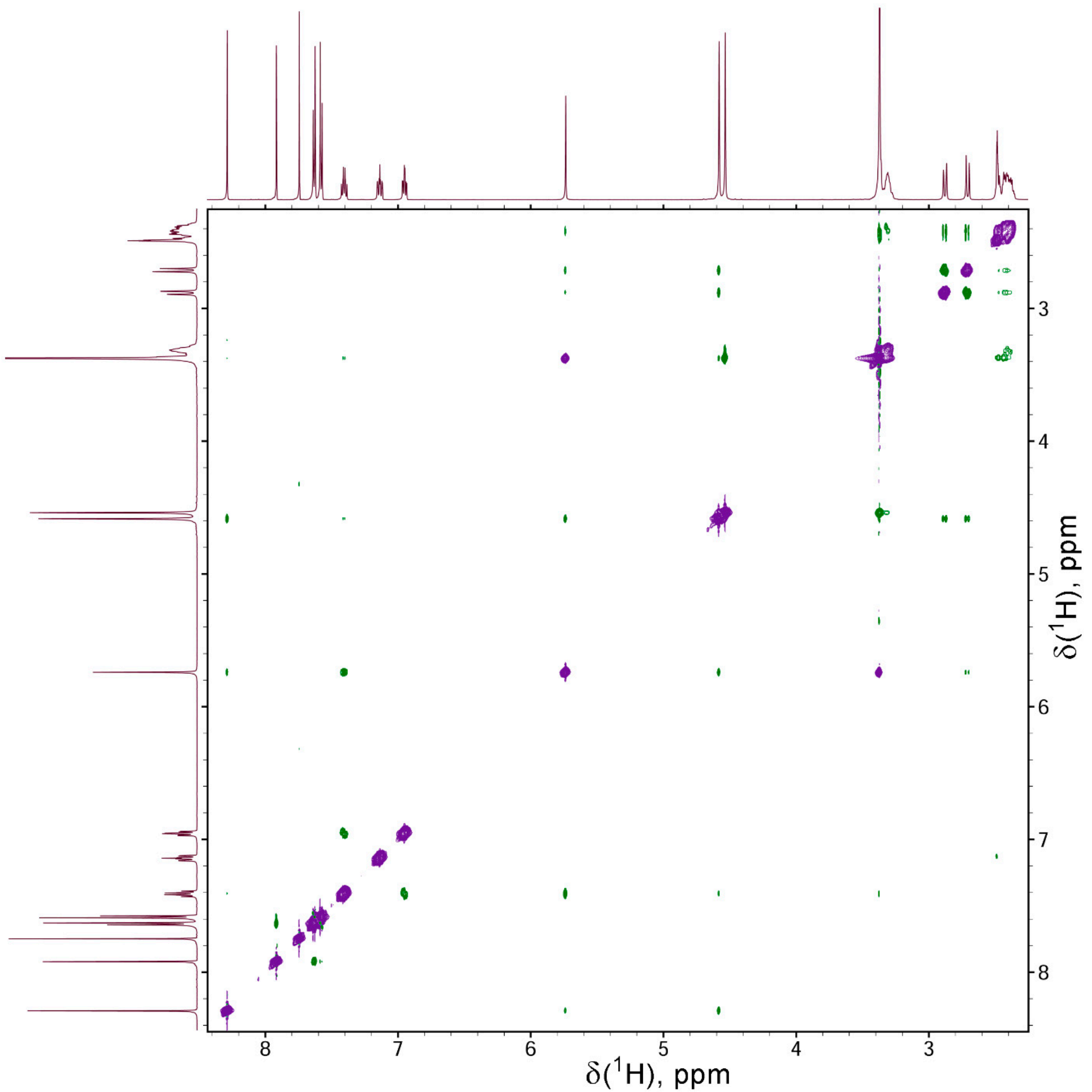

Figure S14. Fragment of the 2D ROESY spectrum of **31a** (**L-173)** recorded in DMSO-d6 at 298K and 600 MHz proton resonance frequency. Green cross-peaks correspond to the dipole-dipole interactions between protons (NOEs), purple cross-peaks correspond to the chemical exchange mechanism of magnetization transfer. It can be seen that rotation around C_{12}-N_{13} amide bond occurs in the time scale of ~100 ms (ROESY mixing time is 350 ms): there is an exchange cross-peak between H_{14} and H_{18} protons. NOE between H_{11} and H_{18} is much stronger than between H_{11} and H_{14}, which confirms assignments of H_{14}-H_{18} resonances.

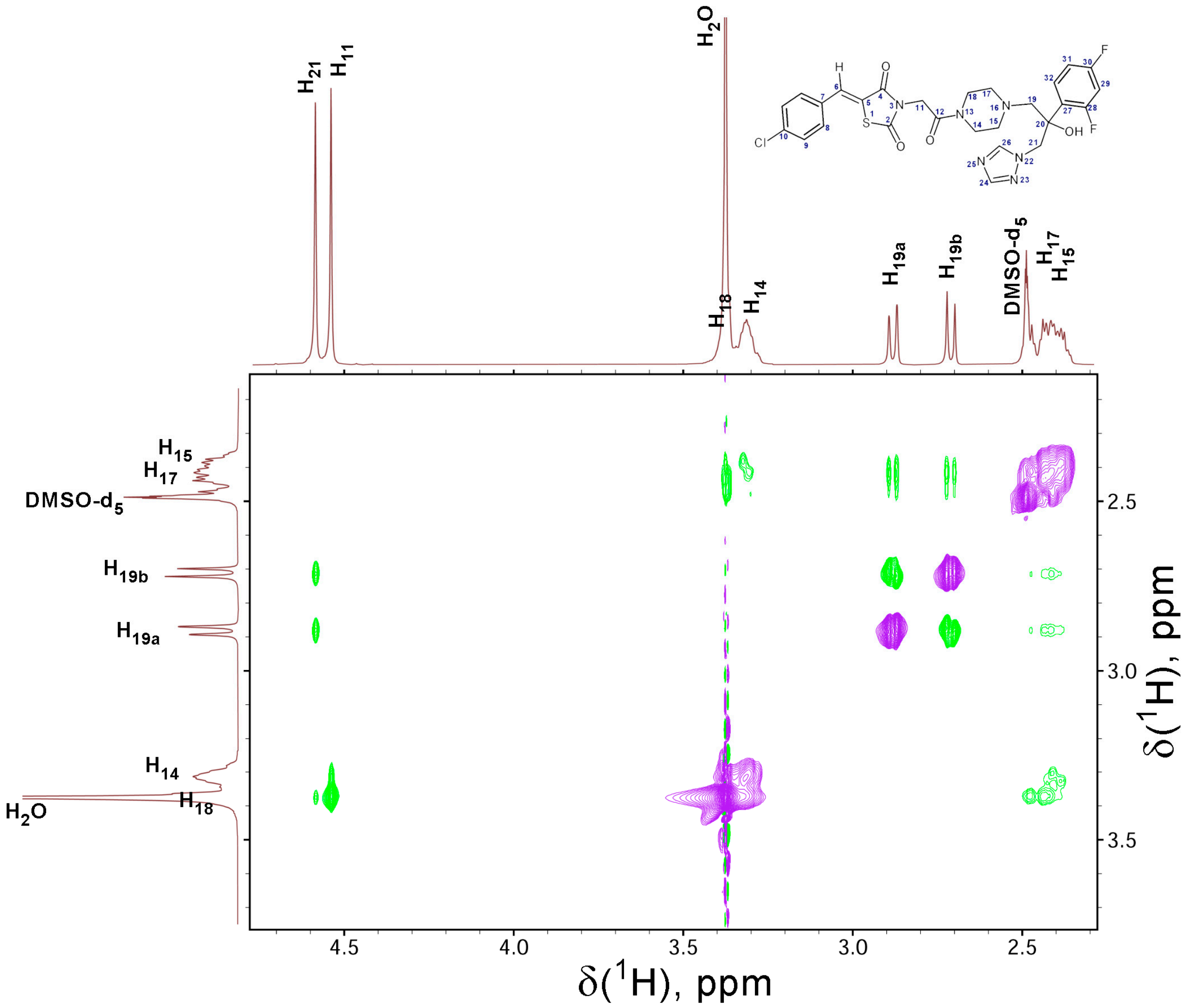

Figure S15. Overlay of 2D spectra ^{13}C-^{1}H HSQC (blue cross-peaks) and ^{13}C-^{1}H HMBC (red cross-peaks) of **31a** (**L-173)** recorded in DMSO-d6 at 298K and 600 MHz proton resonance frequency.

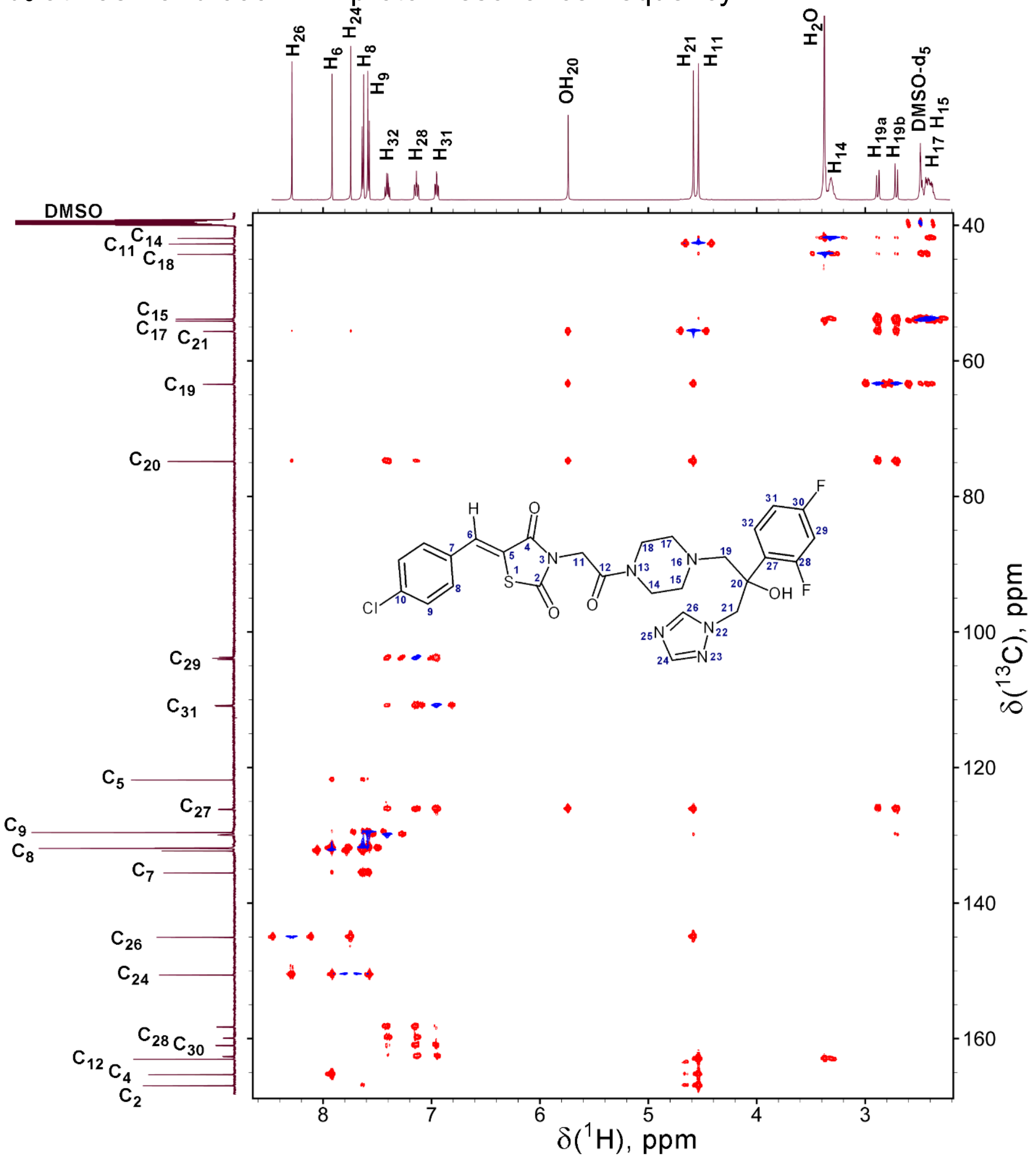

Figure S16. 1D ^{1}H NMR spectrum of **L-163** recorded in DMSO-d6 at 298K and 700 MHz proton resonance frequency.

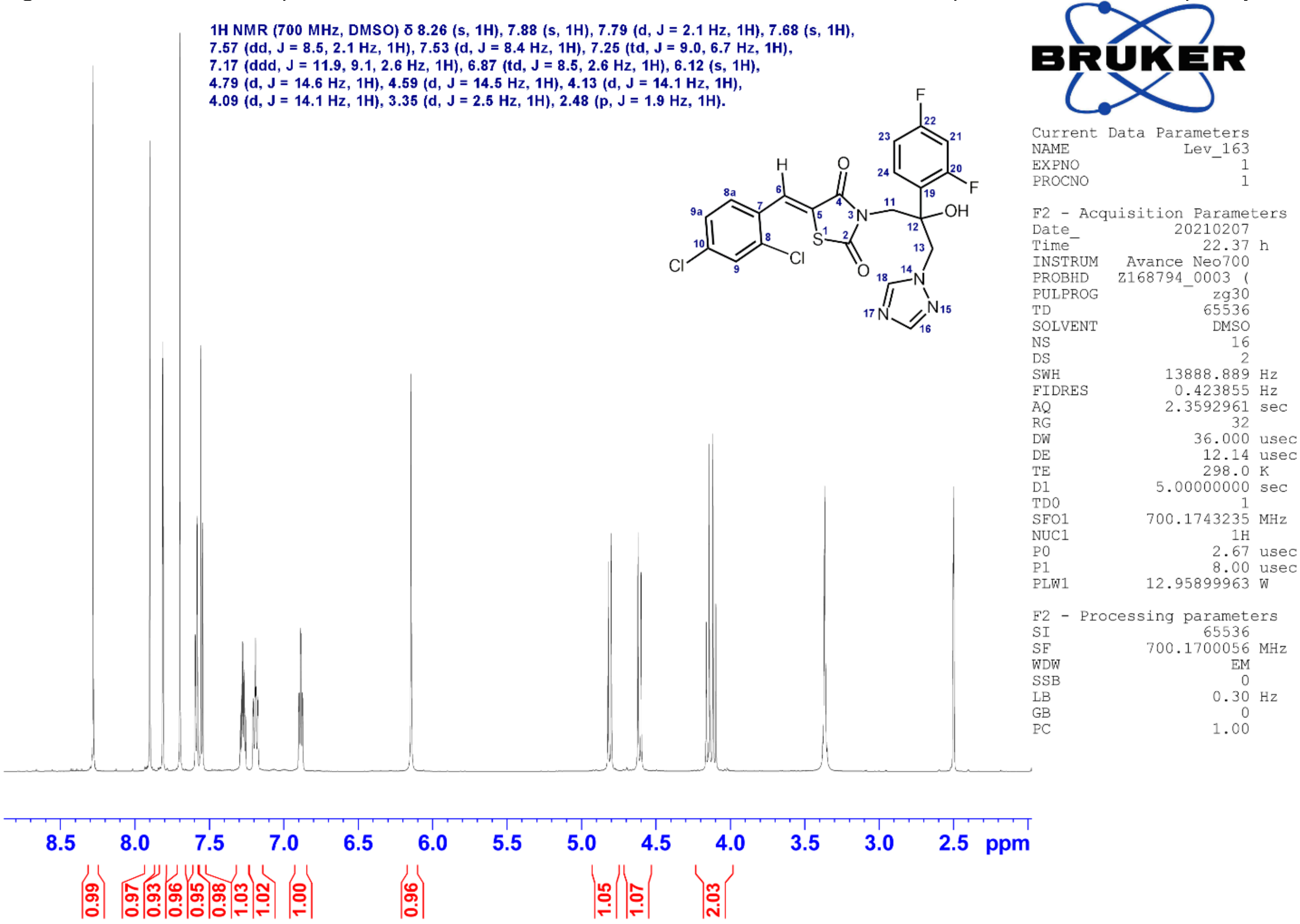

Figure S17. 1D ^{13}C NMR spectrum of **L-163** recorded in DMSO-d_6 at 298K and 176 MHz carbon resonance frequency.

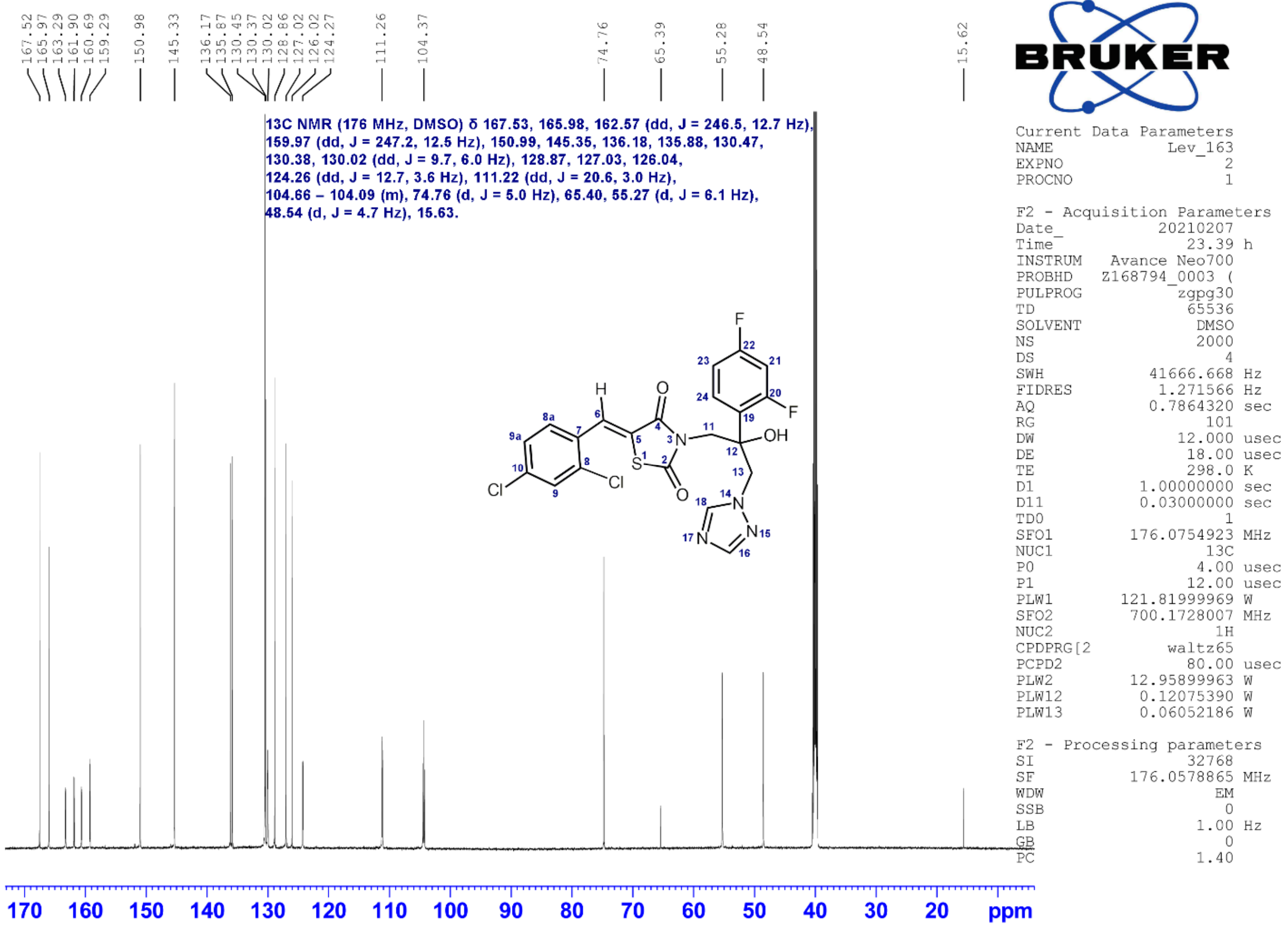

Figure S18. 1D ^{1}H NMR spectrum of **24** recorded in DMSO-d$_6$ at 298K and 400 MHz proton resonance frequency.

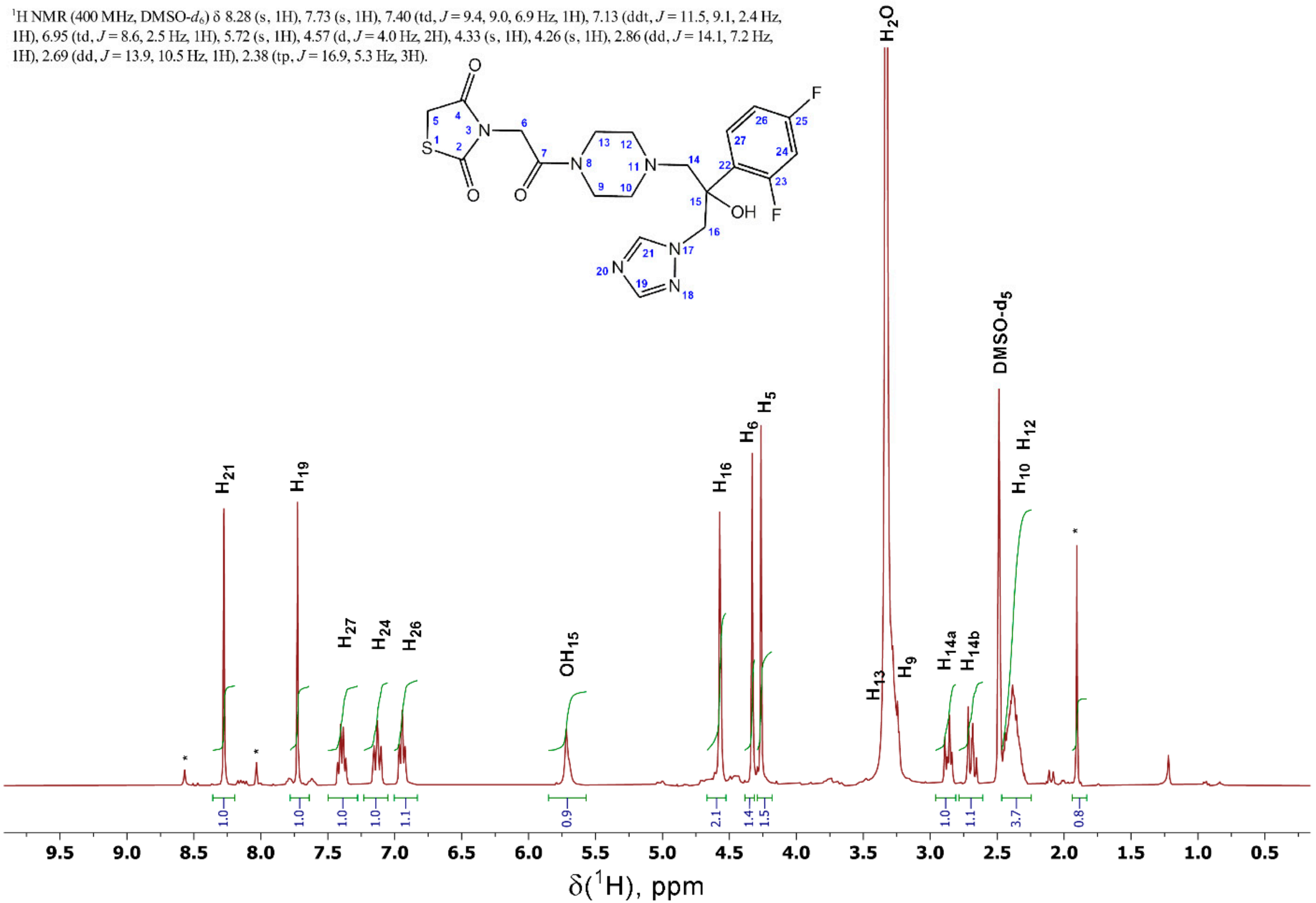

Figure S19. 1D ^{13}C NMR spectrum of **24** recorded in DMSO-d_6 at 298K and 101 MHz carbon resonance frequency.

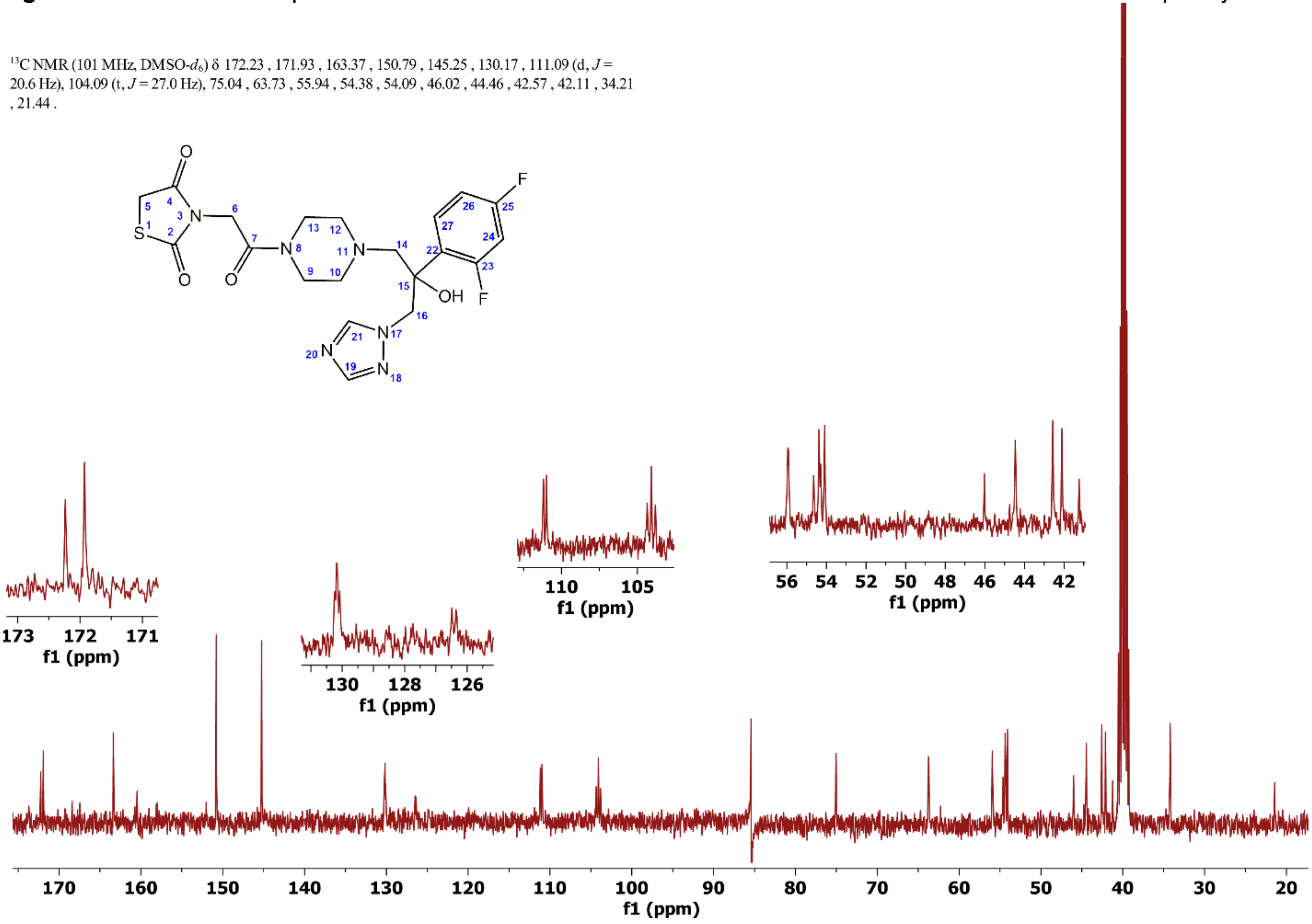

Figure S20. 1D ^{1}H NMR spectrum of **25** recorded in DMSO-d$_6$ at 298K and 400 MHz proton resonance frequency.

^{1}H NMR (400 MHz, DMSO-d_6) δ 8.26 (s, 1H), 7.72 (s, 1H), 7.38 (td, J = 8.9, 6.7 Hz, 1H), 7.12 (ddd, J = 11.9, 9.1, 2.6 Hz, 1H), 6.93 (td, J = 8.5, 2.6 Hz, 1H), 5.69 (s, 1H), 4.94 (qd, J = 7.1, 1.8 Hz, 1H), 4.55 (s, 2H), 4.21 (d, J = 2.7 Hz, 2H), 3.10 (s, 2H), 2.84 (d, J = 13.9 Hz, 1H), 2.67 (dd, J = 13.9, 2.1 Hz, 1H), 2.34 (s, 4H), 1.44 – 1.30 (m, 3H).

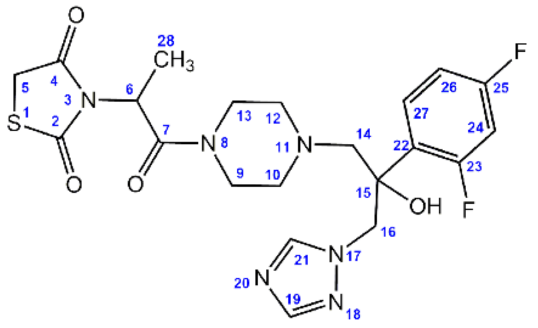

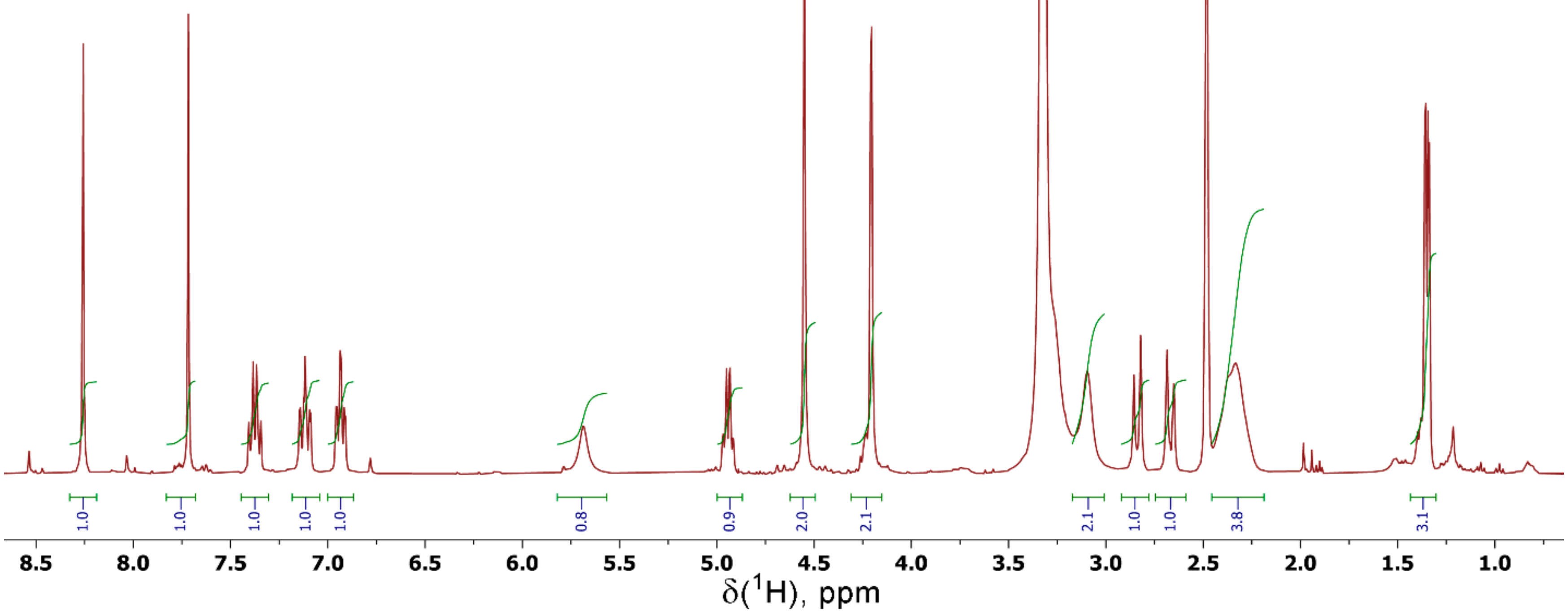

Figure S21. 1D ^{13}C NMR spectrum of **25** recorded in DMSO-d6 at 298K and 101 MHz carbon resonance frequency.

^{13}C NMR (101 MHz, DMSO-d_6) δ 171.95 , 171.78 , 166.34 , 150.80 , 145.24 , 130.15 , 111.09 (d, J = 20.2 Hz), 103.97 (d, J = 27.4 Hz), 75.10 , 63.71 , 55.94 , 54.29 , 49.97 , 45.18 , 42.41 , 33.79 , 14.79 .

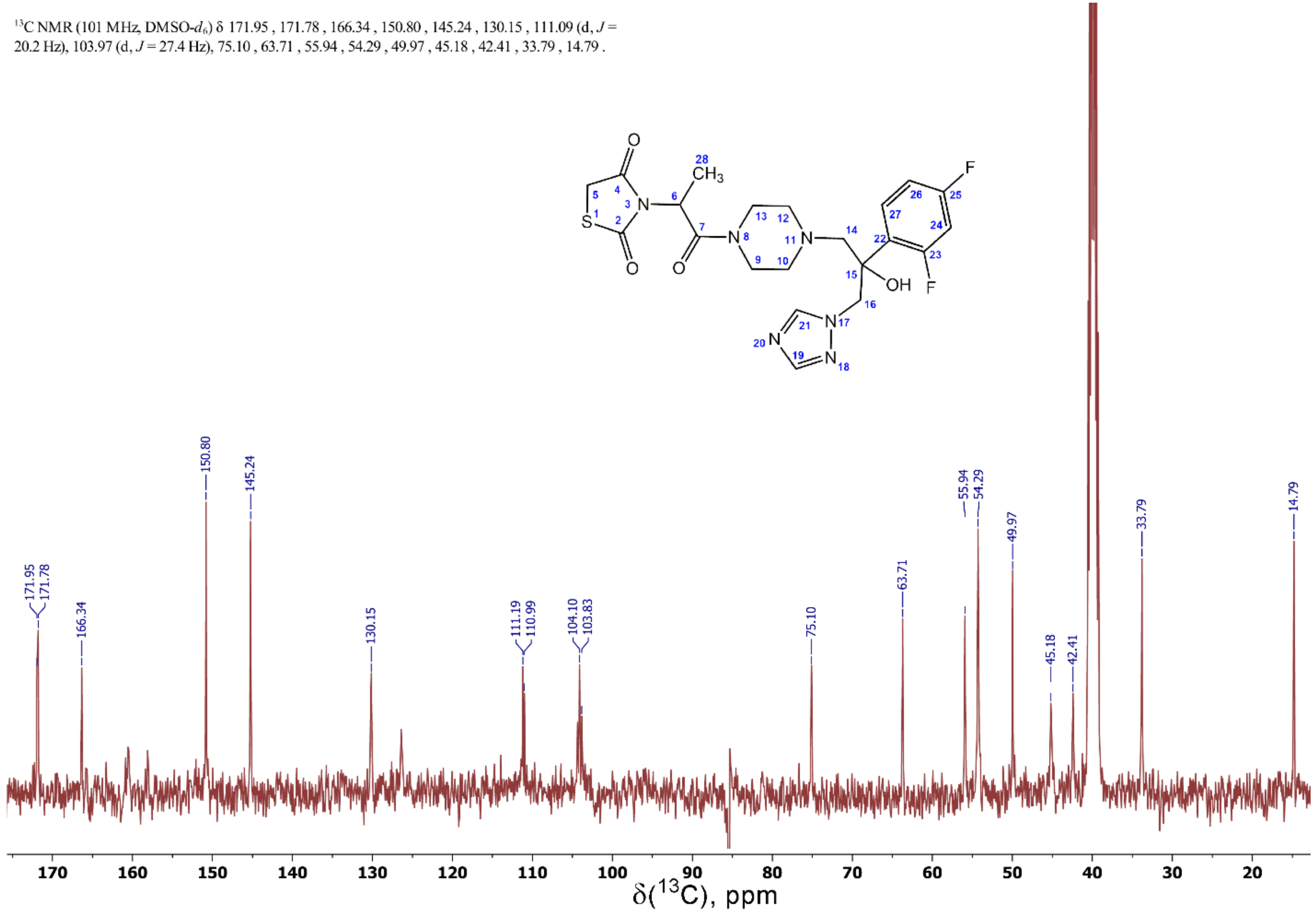

Figure S22. 1D ^{1}H NMR spectrum of **26** recorded in DMSO-d$_6$ at 298K and 400 MHz proton resonance frequency.

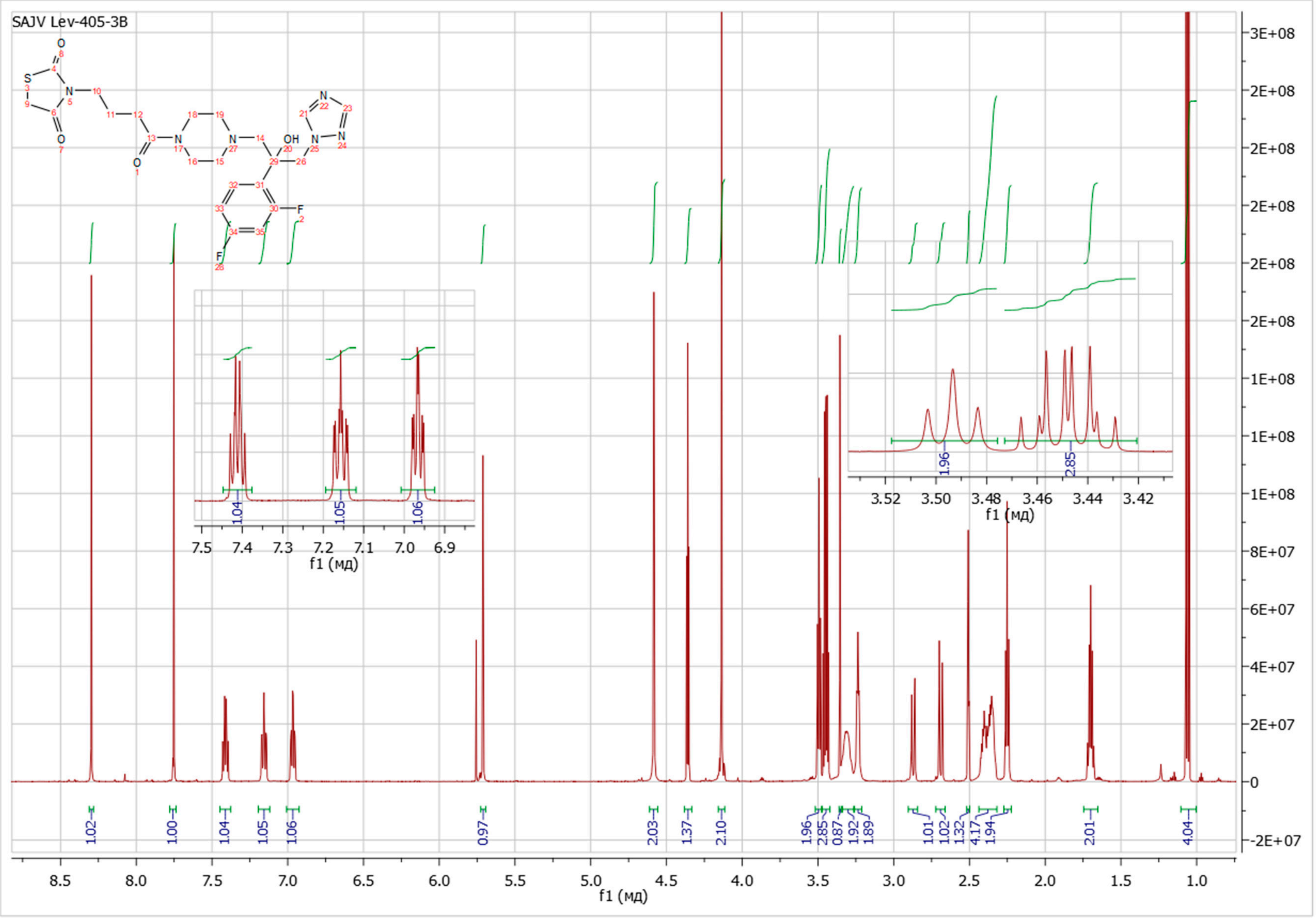

Figure S23. 1D ^{13}C NMR spectrum of **26** recorded in DMSO-d_6 at 298K and 101 MHz carbon resonance frequency.

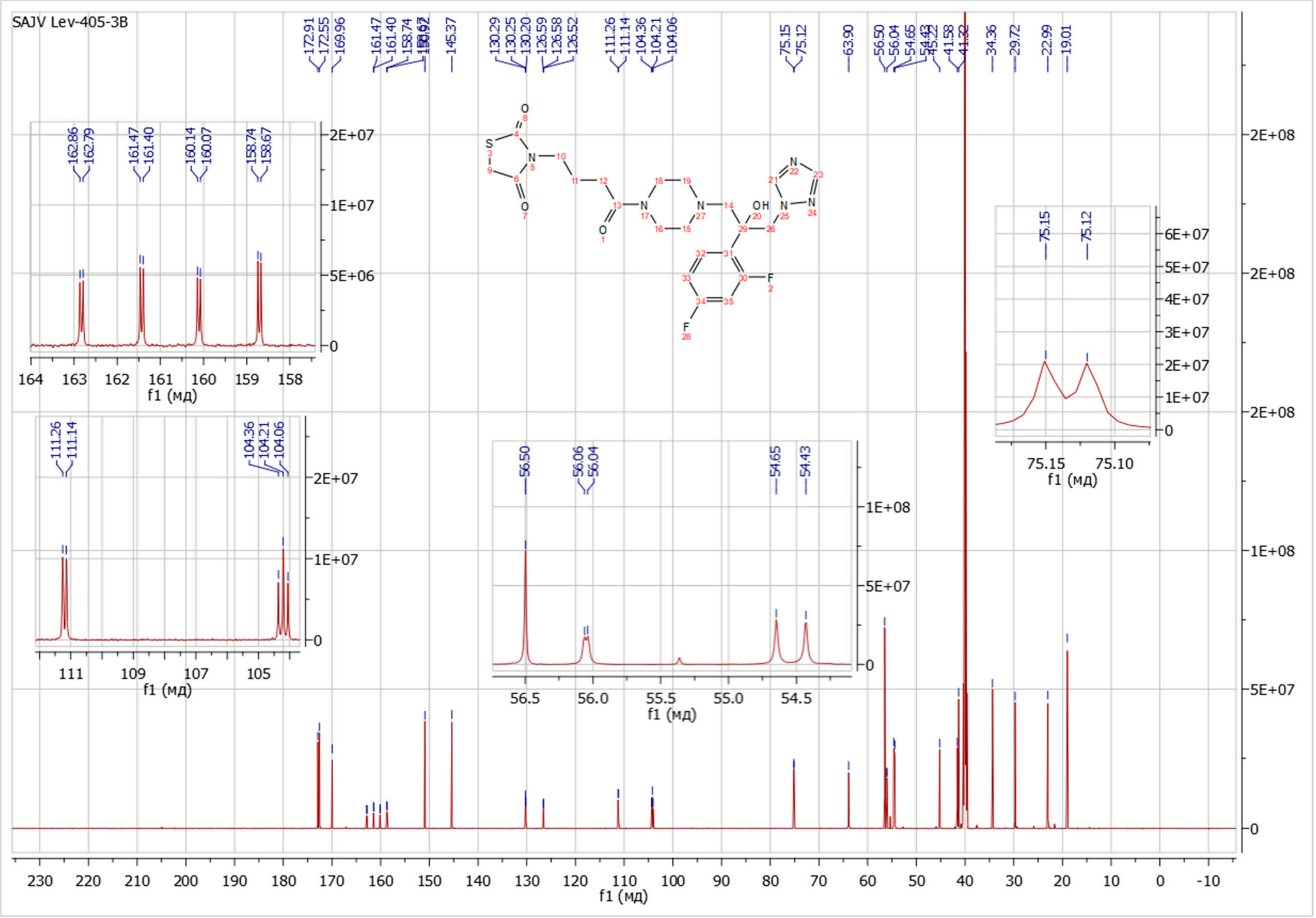

Figure S24. 1D ^{1}H NMR spectrum of **27** recorded in DMSO-d_6 at 298K and 700 MHz proton resonance frequency.

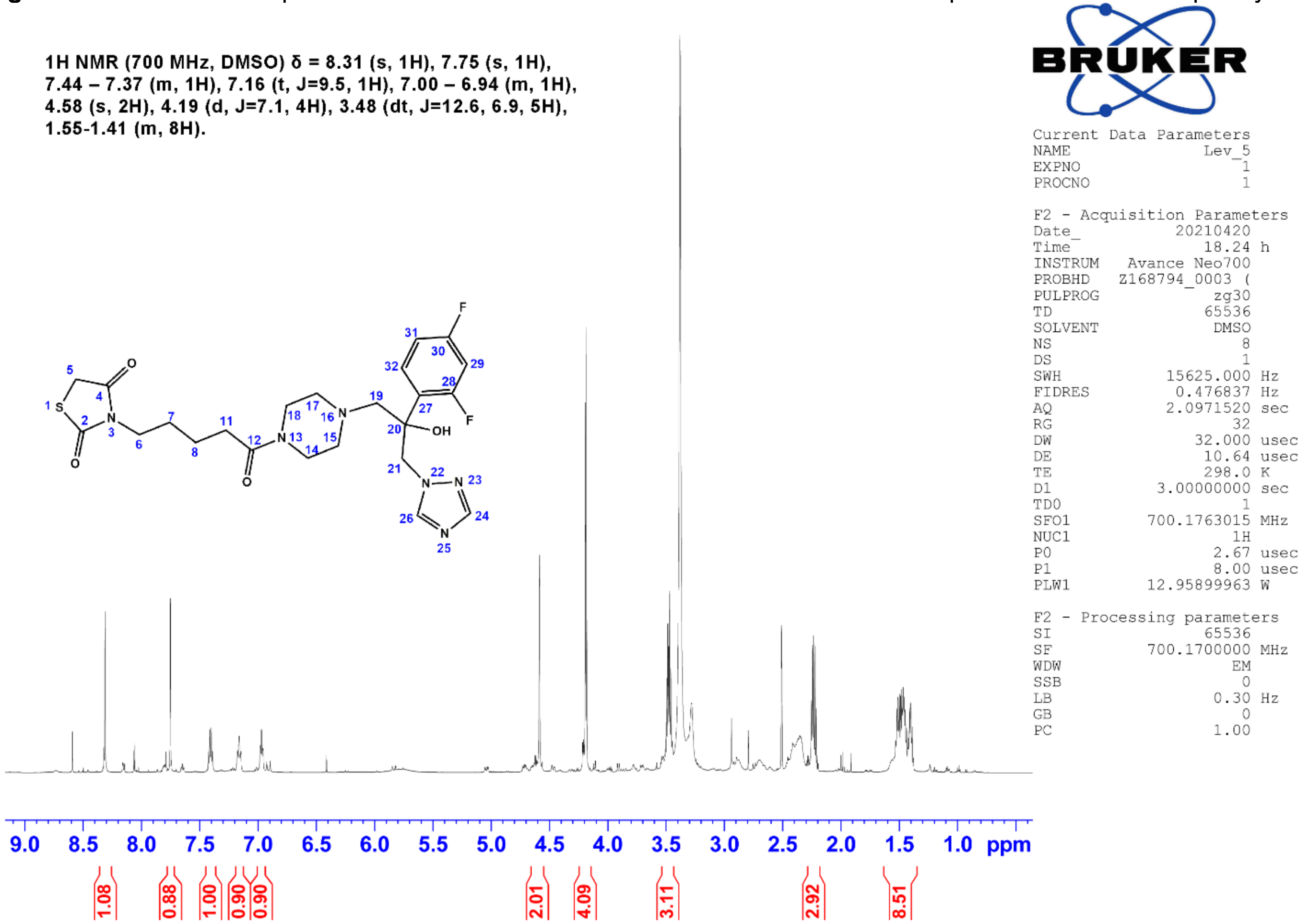

Figure S25. 1D ^{13}C NMR spectrum of **27** recorded in DMSO-d_6 at 298K and 176 MHz carbon resonance frequency.

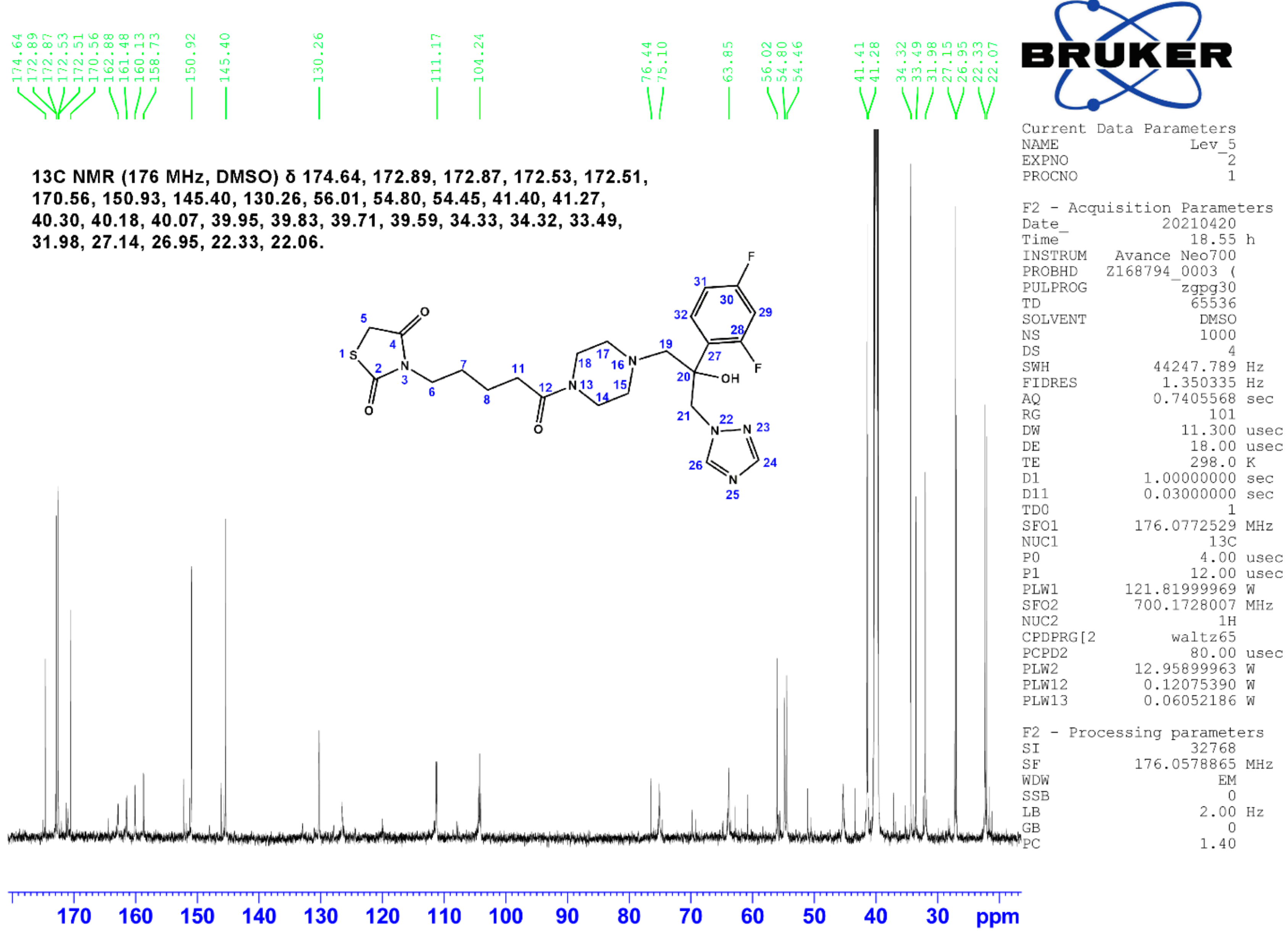

Figure S26. 1D ^{1}H NMR spectrum of **29** recorded in DMSO-d_6 at 298K and 700 MHz proton resonance frequency.

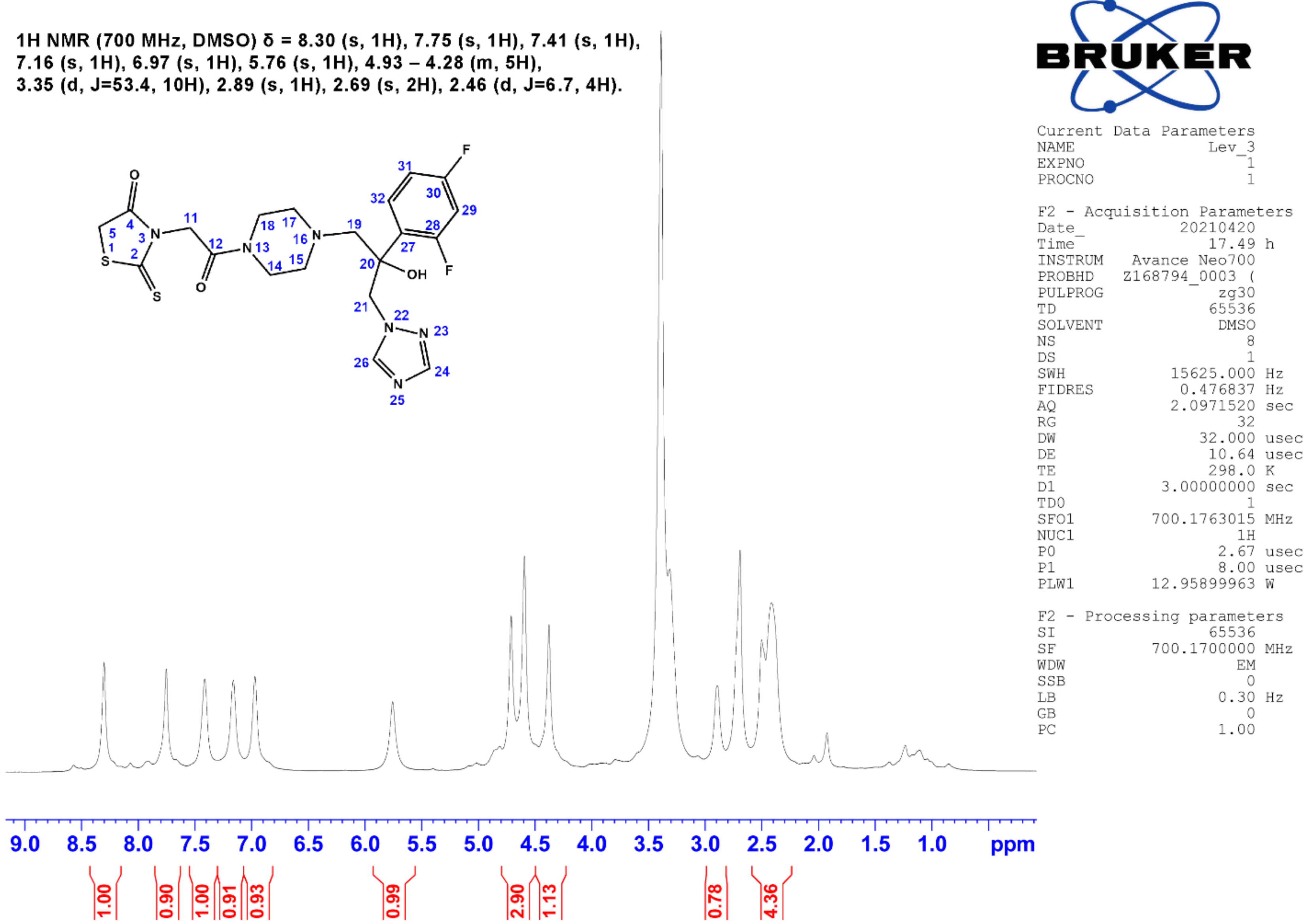

Figure S27. 1D ^{13}C NMR spectrum of **29** recorded in DMSO-d_6 at 298K and 176 MHz carbon resonance frequency.

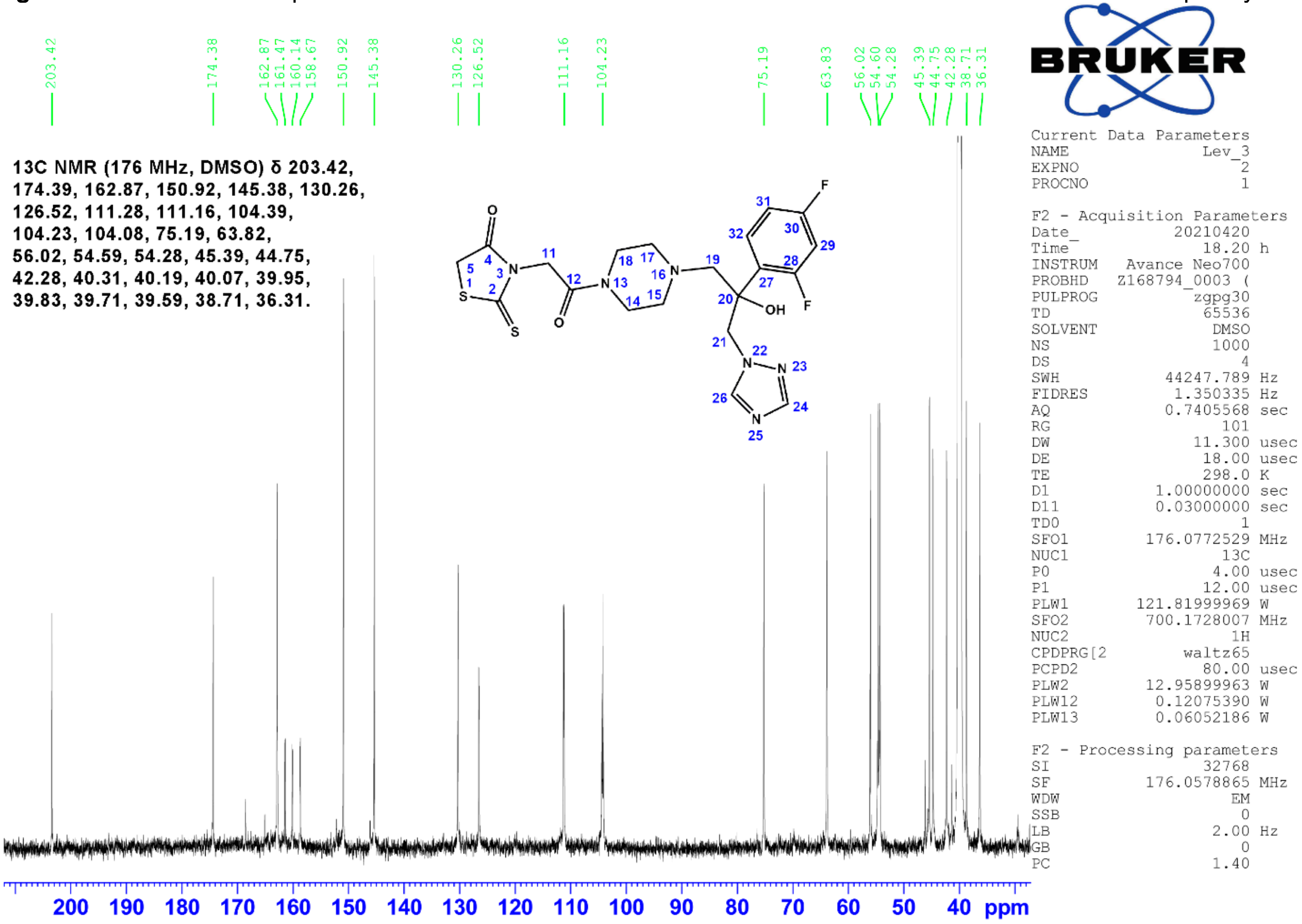

Figure S28. 1D ^{1}H NMR spectrum of **30** recorded in DMSO-d_6 at 298K and 700 MHz proton resonance frequency.

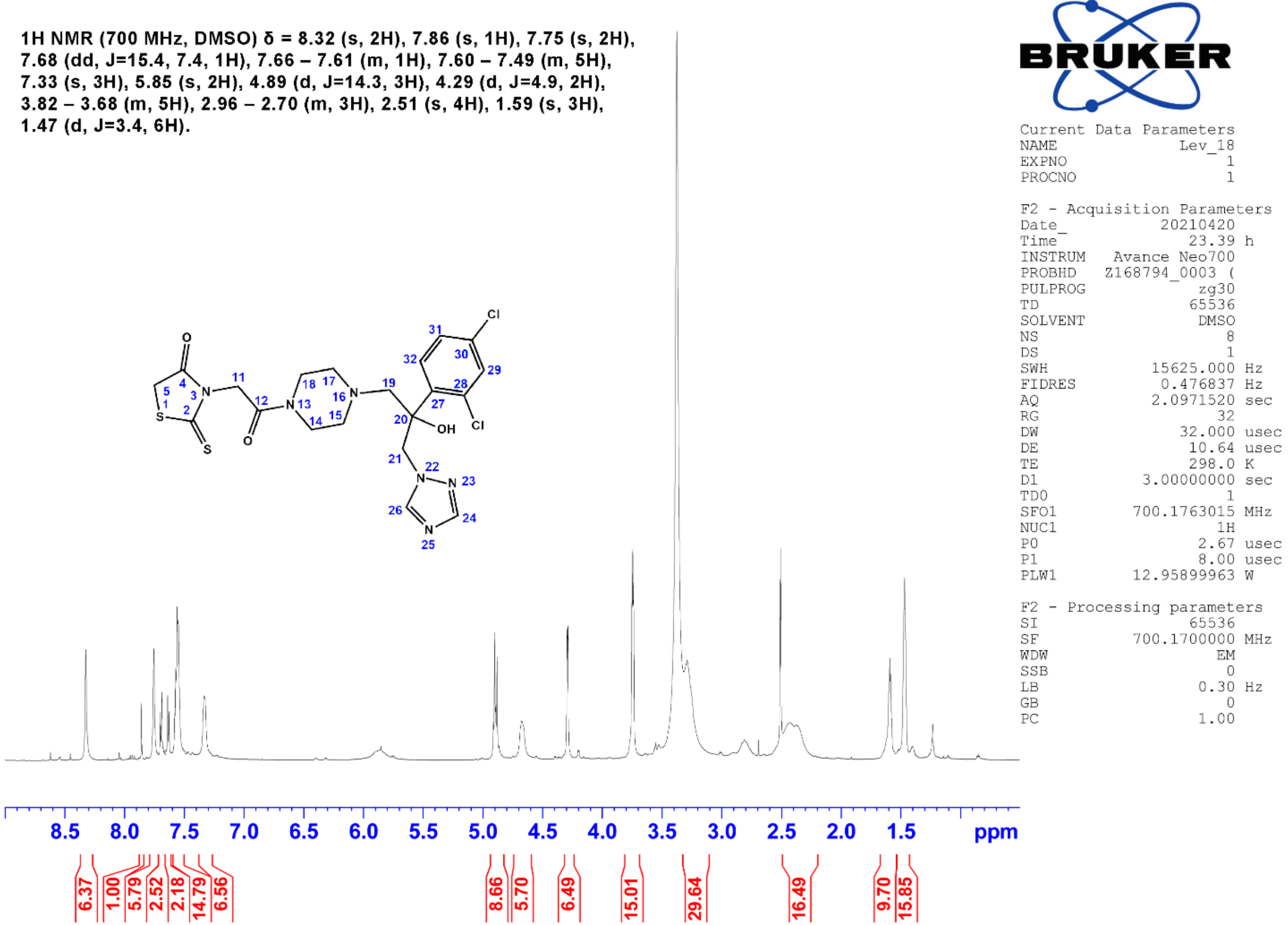

Figure S29. 1D ^{13}C NMR spectrum of **30** recorded in DMSO-d_6 at 298K and 176 MHz carbon resonance frequency.

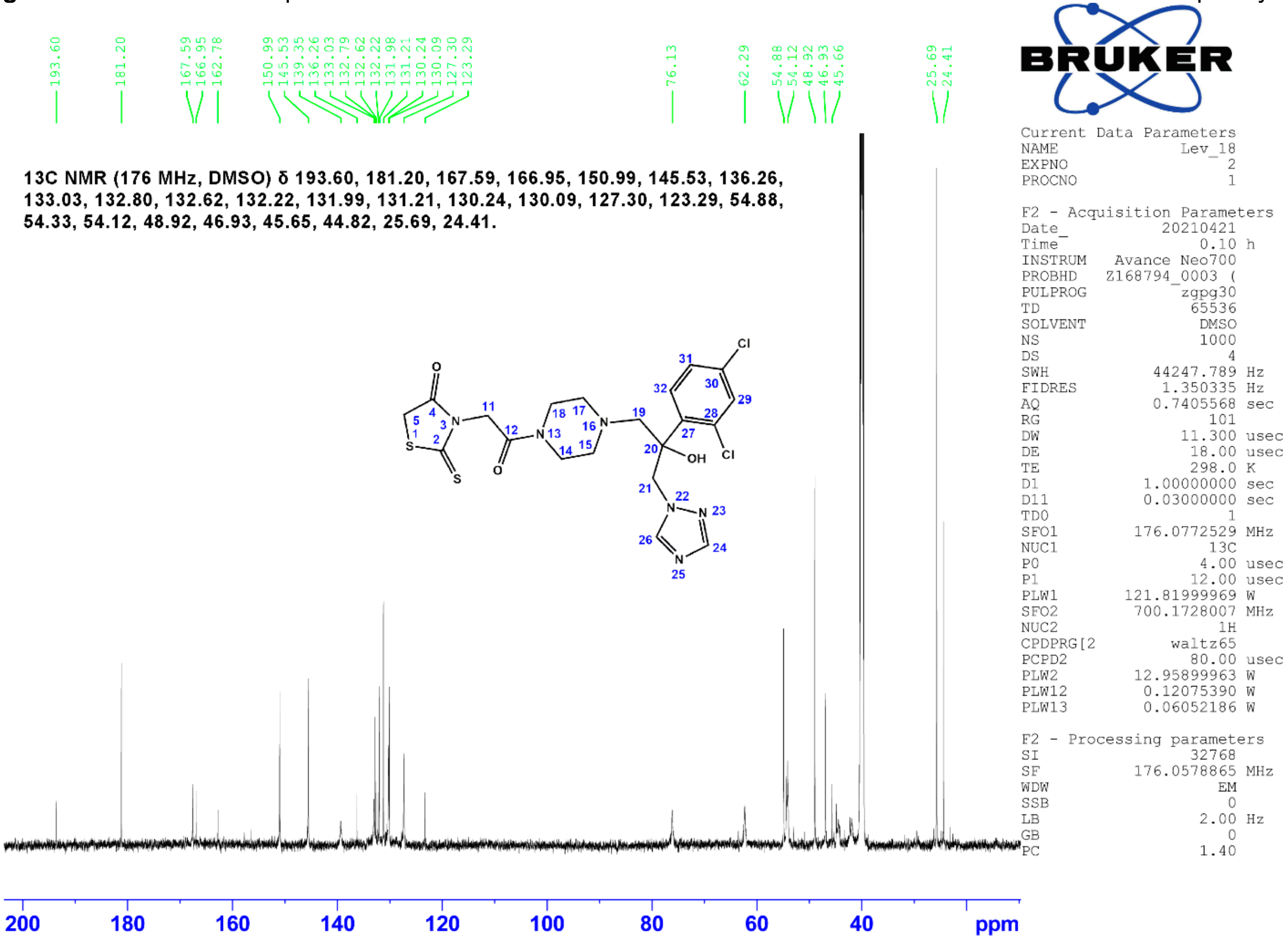

Figure S30. 1D ^{1}H NMR spectrum of **31b** recorded in DMSO-d_6 at 298K and 400 MHz proton resonance frequency. The signals of isopropanol impurity are marked with an asterisk in the spectrum.

^{1}H NMR (400 MHz, DMSO-d_6) δ 8.29 (s, 1H), 7.94 (s, 1H), 7.75 (s, 1H), 7.69 (dd, J = 8.7, 5.4 Hz, 2H), 7.39 (m, J = 20.9, 10.1, 7.8 Hz, 3H), 7.14 (ddd, J = 11.8, 9.1, 2.6 Hz, 1H), 6.95 (td, J = 8.5, 2.6 Hz, 1H), 5.76 (s, 1H), 4.58 (s, 2H), 4.54 (s, 2H), 3.45 – 3.35 (m, 3H), 3.34 – 3.23 (m, 3H), 2.88 (d, J = 13.9 Hz, 1H), 2.70 (d, J = 13.8 Hz, 1H), 2.56 – 2.33 (m, 5H).

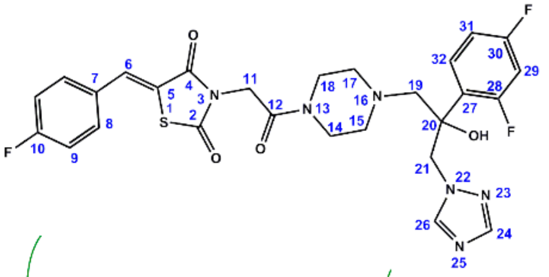

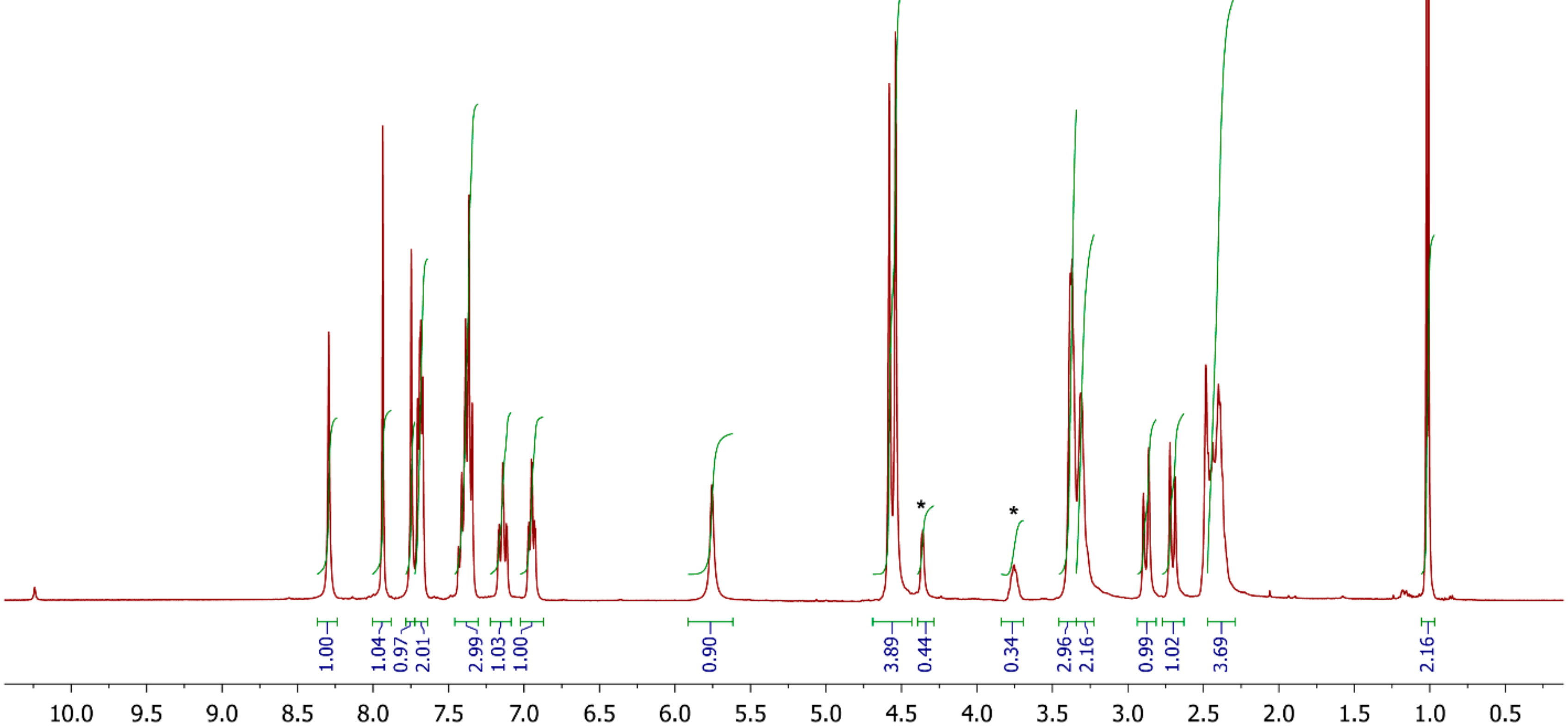

Figure S31. 1D ^{13}C NMR spectrum of **31b** recorded in DMSO-d6 at 298K and 101 MHz carbon resonance frequency. The signals of isopropanol impurity are marked with an asterisk in the spectrum.

^{13}C NMR (101 MHz, DMSO-*d*$_6$) δ 167.37 , 165.67 , 164.75 , 163.34 , 162.25 , 162.11 (dd, *J* = 246.0, 12.7 Hz), 159.37 (dd, *J* = 246.7, 11.9 Hz), 150.92 , 145.37 , 133.17 , 133.09 , 132.85 , 130.23 (t), 129.92 (d, *J* = 3.2 Hz), 126.48 (d, *J* = 13.0 Hz), 121.07 (d, *J* = 2.4 Hz), 117.11 , 116.89 , 111.20 (d, *J* = 20.7 Hz), 104.21 (t, *J* = 26.8 Hz), 75.16 (d, *J* = 5.5 Hz), 62.48 , 56.00 (d), 44.60 , 43.07 , 42.25.tt

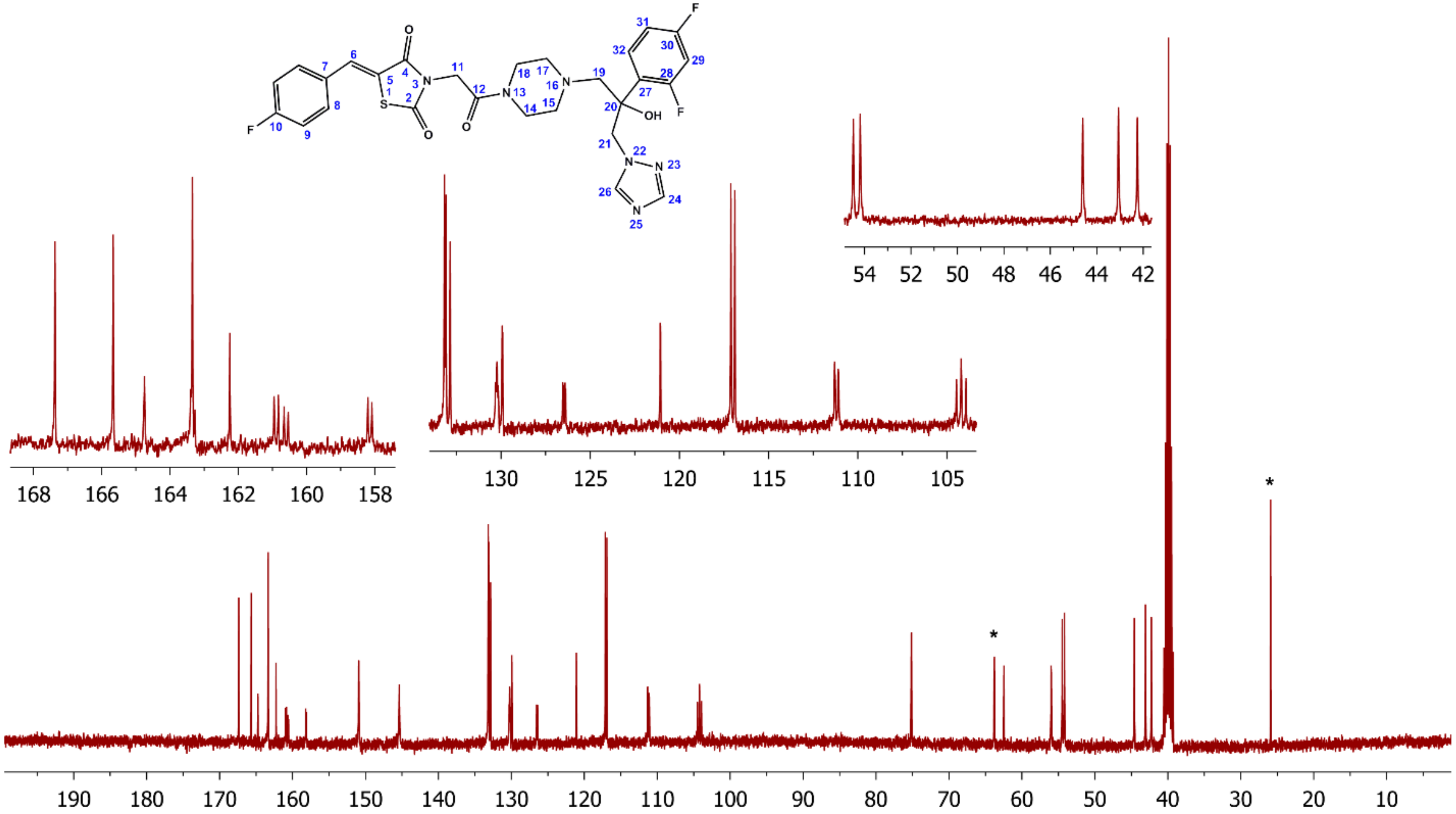

Figure S32. 1D ^{1}H NMR spectrum of **31c** recorded in DMSO-d$_6$ at 298K and 400 MHz proton resonance frequency.

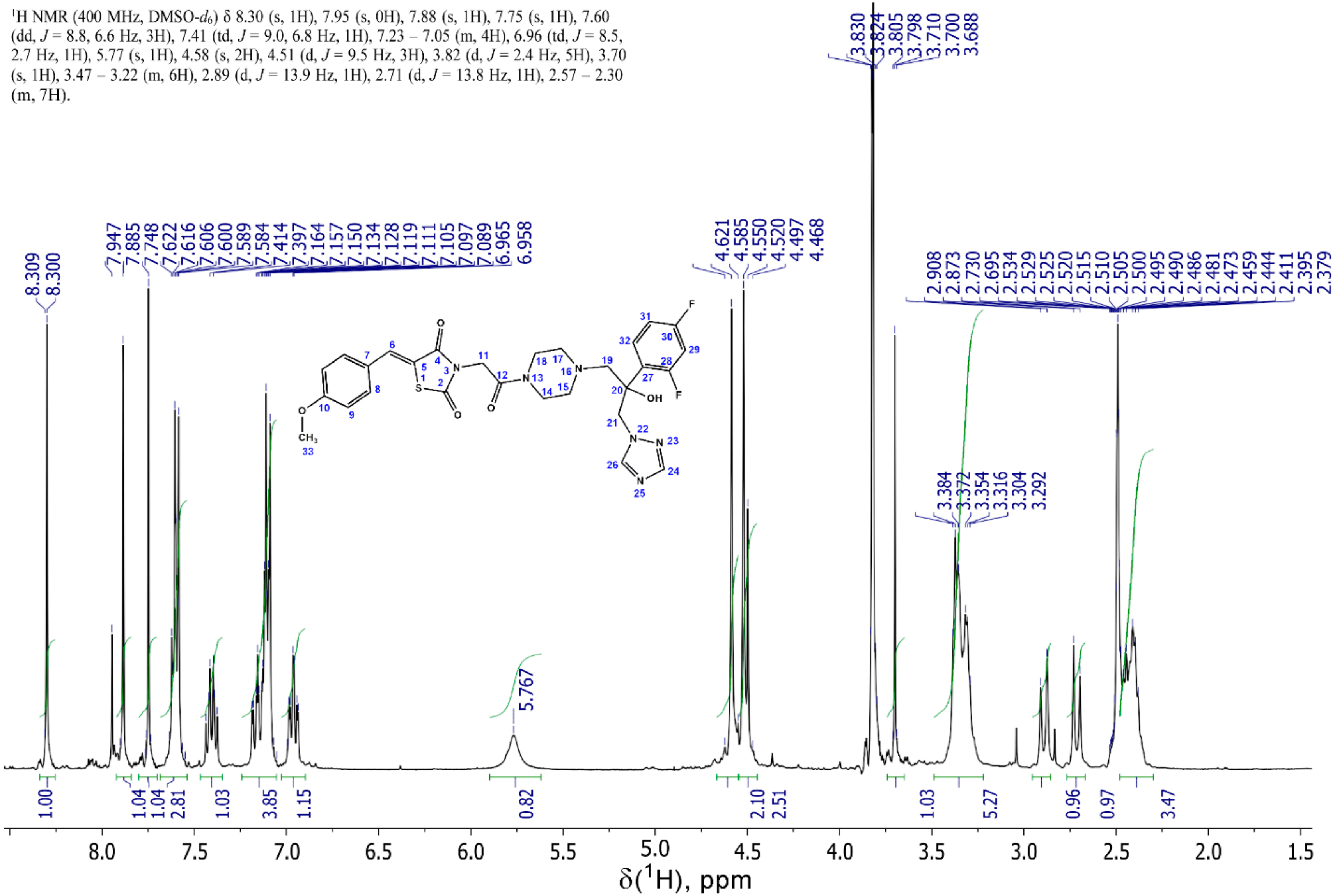

Figure S33. 1D ^{13}C NMR spectrum of **31c** recorded in DMSO-d6 at 298K and 101 MHz carbon resonance frequency.

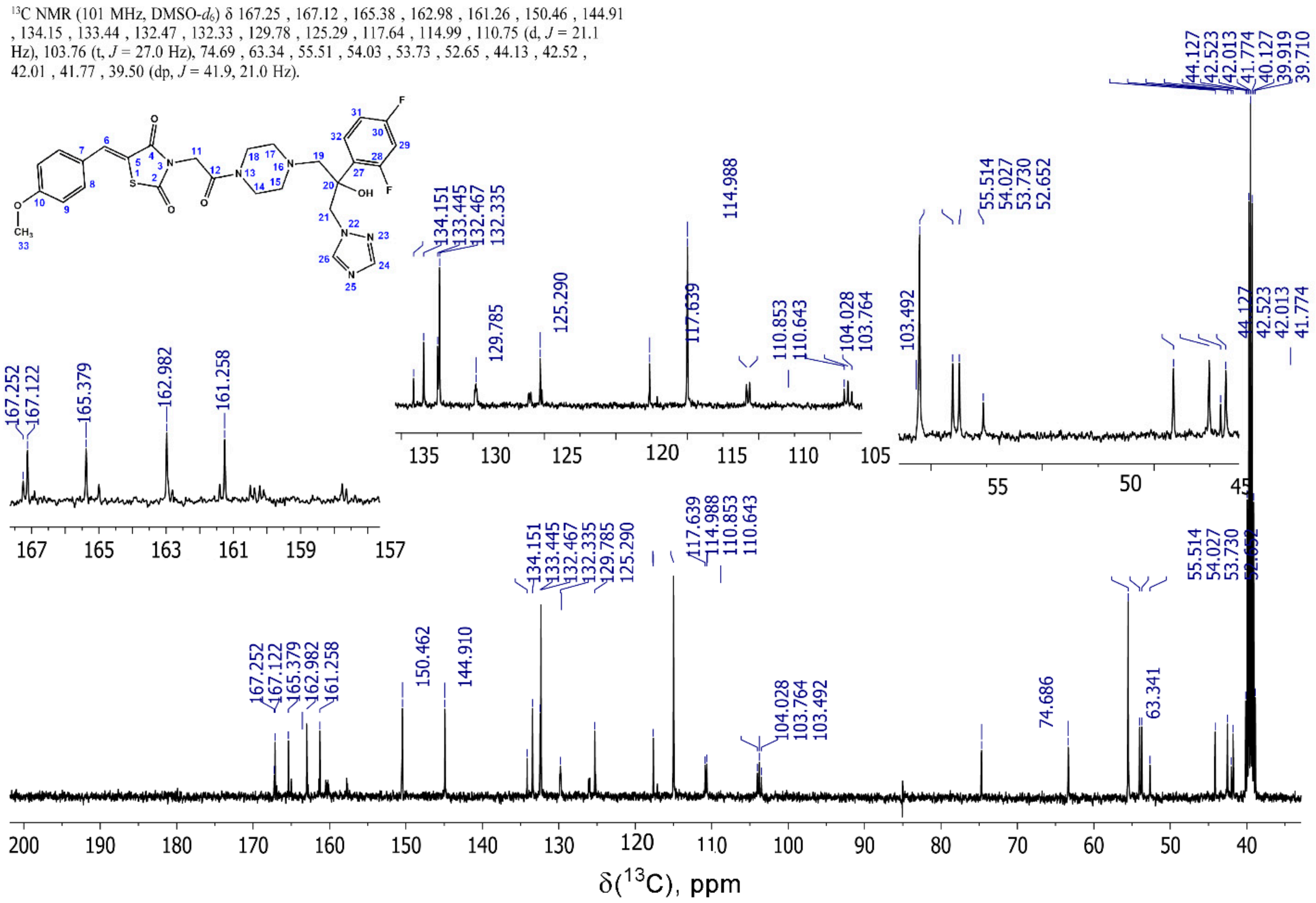

Figure S34. 1D ^{1}H NMR spectrum of **31d** recorded in DMSO-d$_6$ at 298K and 400 MHz proton resonance frequency.

^{1}H NMR (400 MHz, DMSO-*d*$_6$) δ 8.30 (s, 1H), 7.90 (s, 1H), 7.75 (s, 1H), 7.54 (d, *J* = 8.1 Hz, 2H), 7.40 (s, 0H), 7.40 – 7.33 (m, 2H), 7.15 (ddd, *J* = 11.8, 9.1, 2.6 Hz, 1H), 6.96 (td, *J* = 8.5, 2.6 Hz, 1H), 5.76 (s, 1H), 4.58 (s, 2H), 4.53 (s, 2H), 3.36 (d, *J* = 4.9 Hz, 3H), 3.34 – 3.22 (m, 1H), 2.95 – 2.83 (m, 1H), 2.71 (d, *J* = 13.8 Hz, 1H), 2.64 (q, *J* = 7.6 Hz, 2H), 2.42 (qd, *J* = 12.8, 12.3, 6.3 Hz, 2H), 1.17 (t, *J* = 7.6 Hz, 3H).

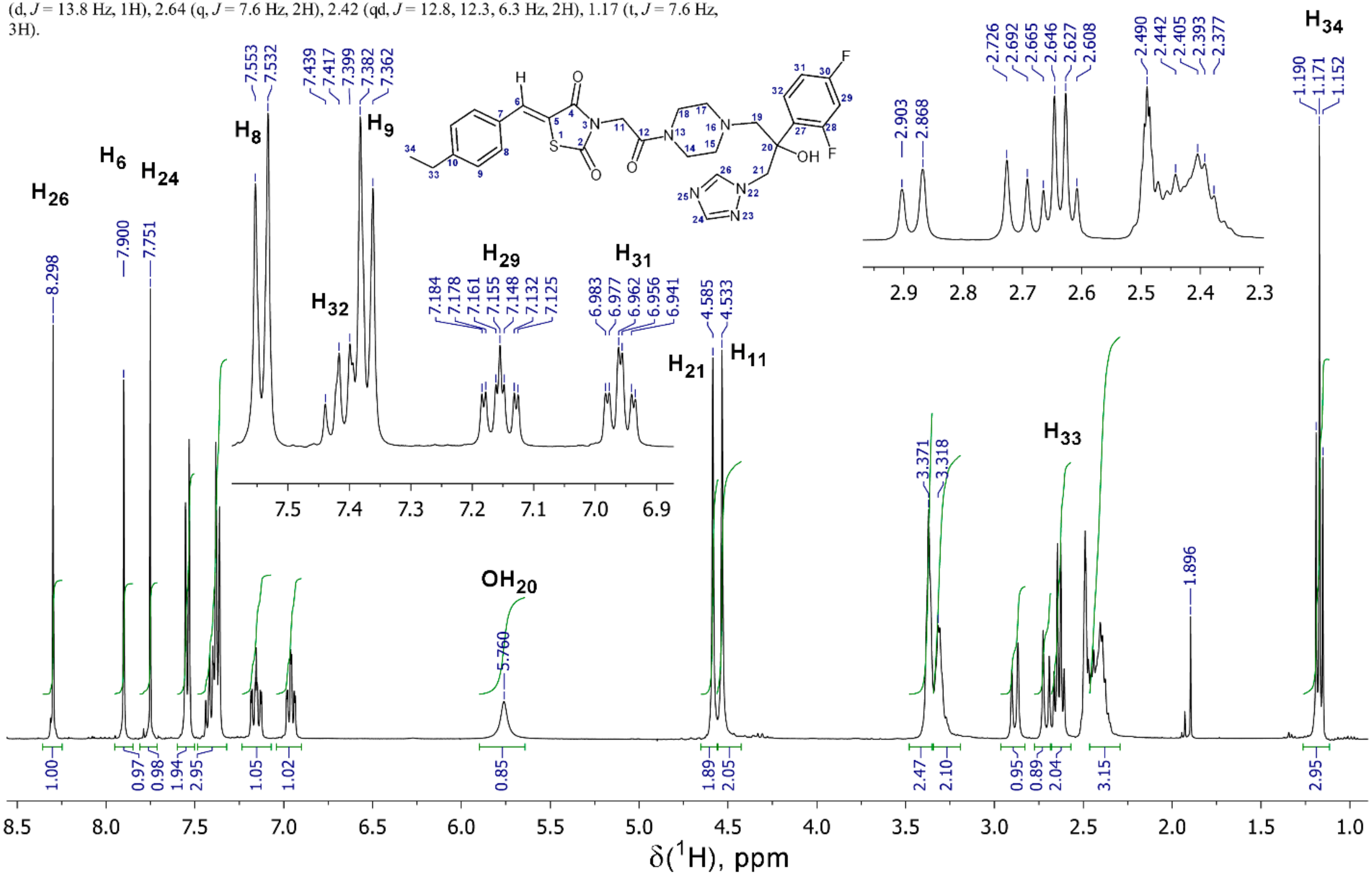

Figure S35. 1D ^{13}C NMR spectrum of **31d** recorded in DMSO-d$_6$ at 298K and 101 MHz carbon resonance frequency.

^{13}C NMR (101 MHz, DMSO-d_6) δ 167.50 , 165.74 , 163.35 , 160.86 (d, J = 12.3 Hz), 160.57 (d, J = 12.2 Hz), 158.12 (d, J = 12.3 Hz), 150.88 , 147.65 , 145.32 , 133.94 , 130.76 (d, J = 4.2 Hz), 130.17 (d, J = 8.6 Hz), 129.26 , 126.45 (d, J = 11.7 Hz), 120.17 , 111.16 (d, J = 20.4 Hz), 104.19 (t, J = 26.8 Hz), 75.14 (d, J = 5.4 Hz), 63.77 , 55.96 , 54.46 , 54.16 , 44.57 , 42.99 , 42.22 , 39.90 (dp, J = 41.8, 20.9 Hz), 28.55 , 15.59 .

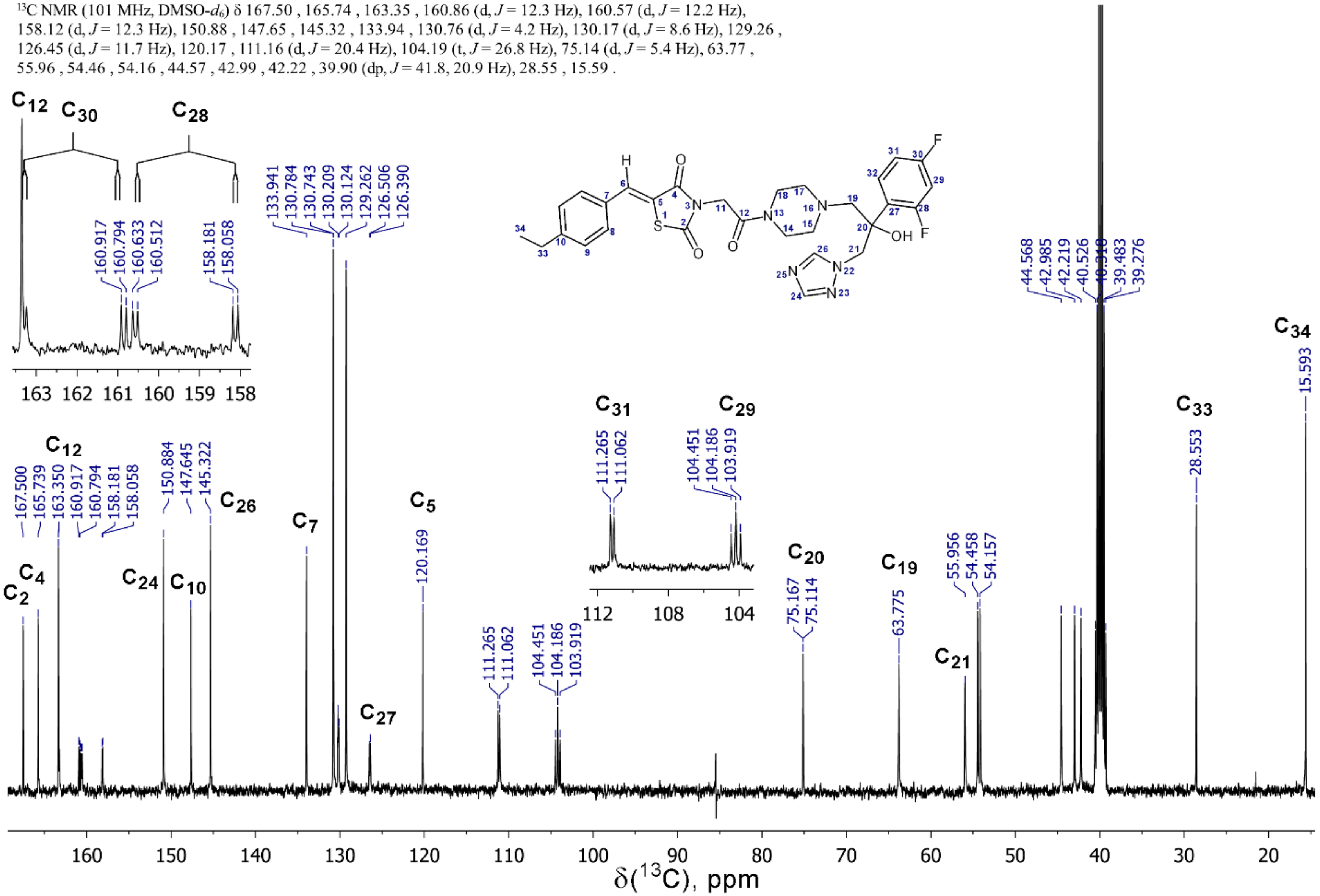

Figure S36. 1D ^{1}H NMR spectrum of **31e** recorded in DMSO-d$_6$ at 298K and 400 MHz proton resonance frequency.

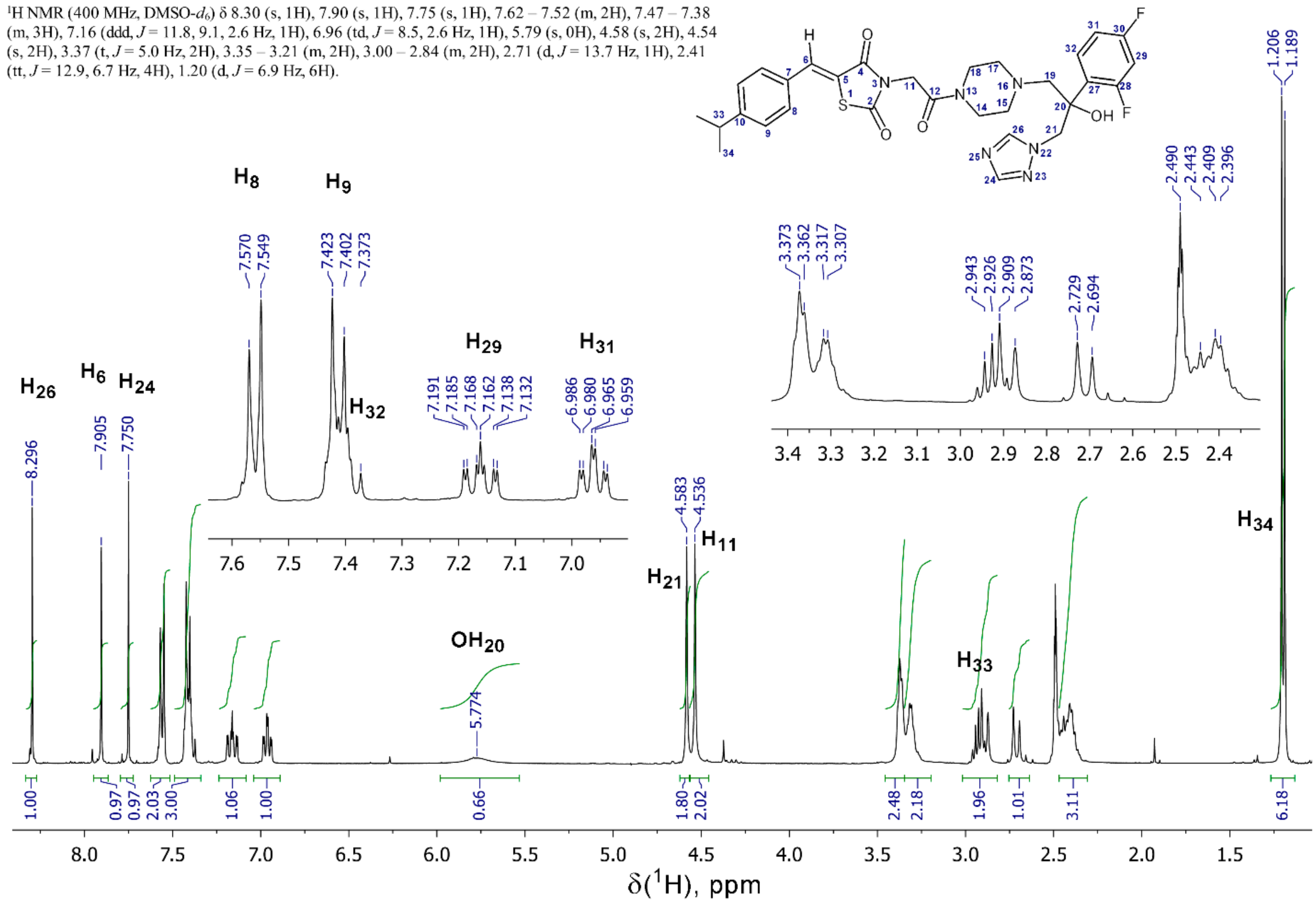

Figure S37. 1D ^{13}C NMR spectrum of **31e** recorded in DMSO-d$_6$ at 298K and 101 MHz carbon resonance frequency.

^{13}C NMR (101 MHz, DMSO-d_6) δ 167.51 , 165.74 , 163.35 , 160.85 (d, J = 12.5 Hz), 160.63 , 158.05 , 152.13 , 150.88 , 145.32 , 133.90 , 130.86 (d, J = 6.7 Hz), 130.16 (d, J = 9.4 Hz), 127.84 , 126.44 (d, J = 13.7 Hz), 120.21 , 111.17 (d, J = 20.1 Hz), 104.60 – 103.83 (m), 85.45 , 75.12 (d, J = 5.5 Hz), 63.76 , 55.95 , 54.45 , 54.15 , 44.54 , 42.99 , 42.19 , 39.90 (dp, J = 42.1, 21.0 Hz), 33.86 , 23.90 .

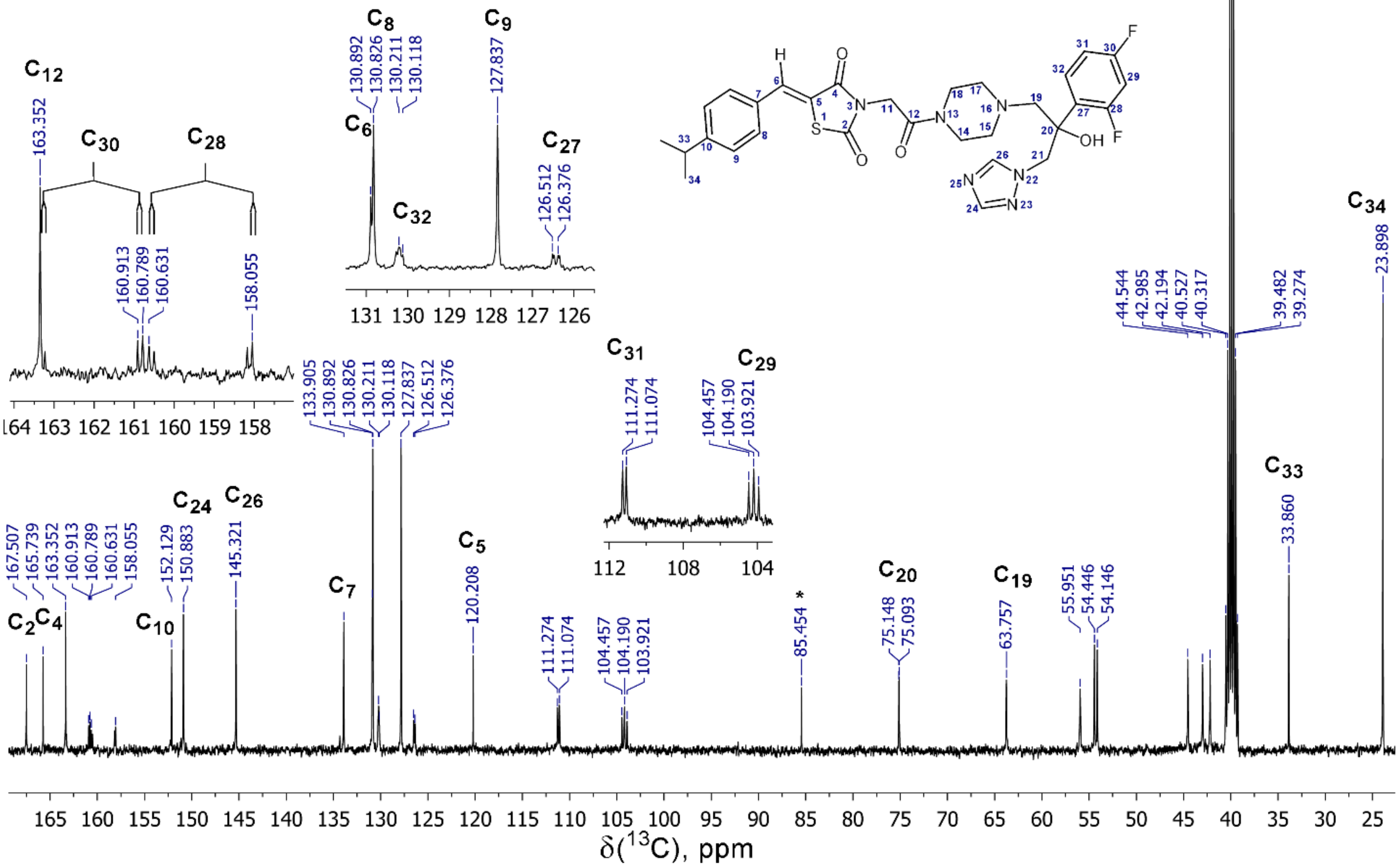

Figure S38. 1D ^{1}H NMR spectrum of **31f** recorded in DMSO-d$_6$ at 298K and 400 MHz proton resonance frequency.

^{1}H NMR (400 MHz, DMSO-d_6) δ 8.30 (s, 1H), 7.91 (s, 1H), 7.75 (s, 1H), 7.57 (d, J = 3.0 Hz, 4H), 7.40 (td, J = 9.0, 6.8 Hz, 1H), 7.16 (ddd, J = 11.8, 9.1, 2.6 Hz, 1H), 6.96 (td, J = 8.5, 2.6 Hz, 1H), 5.78 (s, 1H), 4.58 (s, 2H), 4.54 (s, 2H), 3.37 (d, J = 5.2 Hz, 2H), 3.34 – 3.21 (m, 3H), 2.89 (d, J = 13.9 Hz, 1H), 2.71 (d, J = 13.8 Hz, 1H), 2.42 (h, J = 6.4 Hz, 4H), 1.28 (s, 11H).

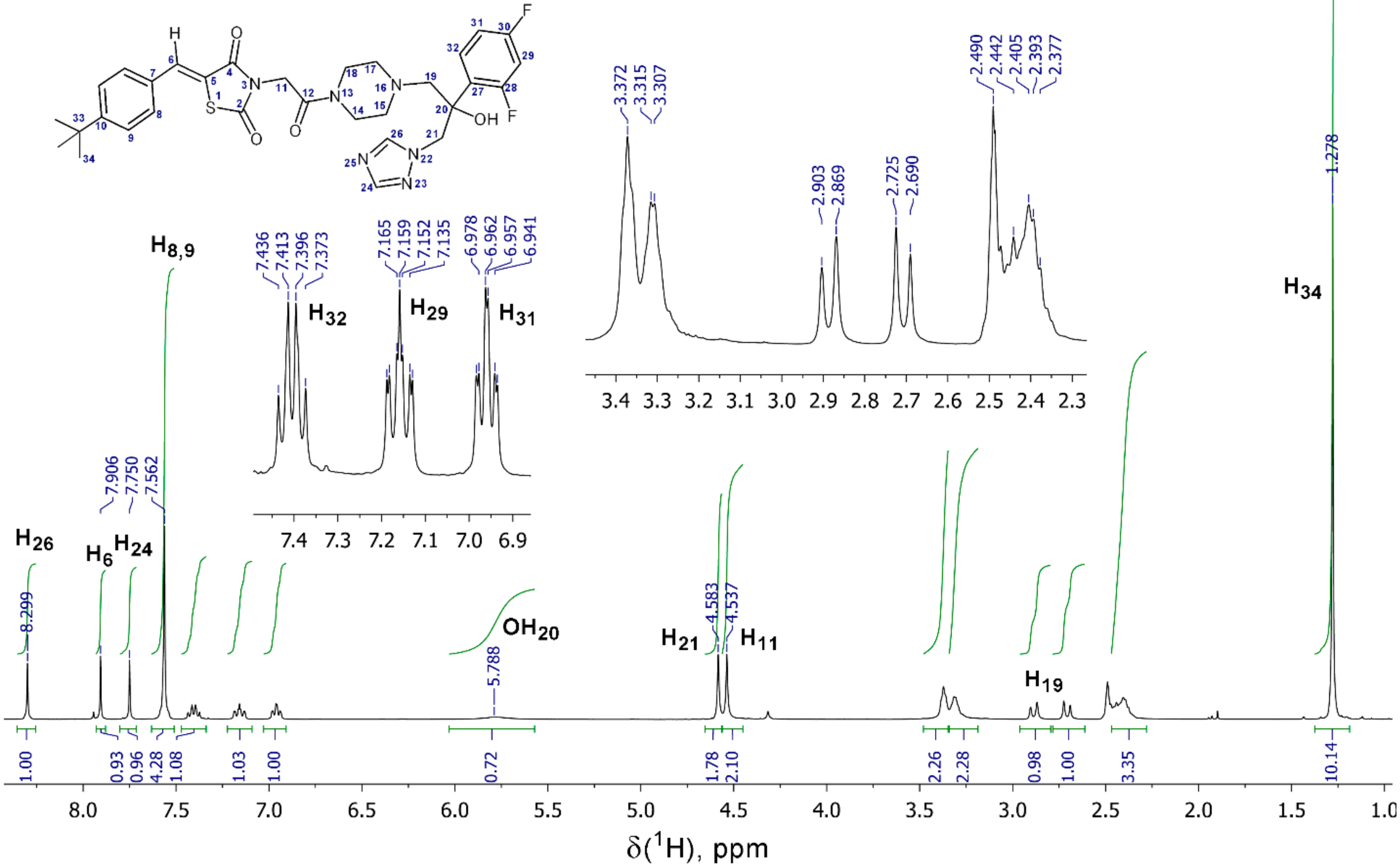

Figure S39. 1D ^{13}C NMR spectrum of **31f** recorded in DMSO-d6 at 298K and 101 MHz carbon resonance frequency.

^{13}C NMR (101 MHz, DMSO-d_6) δ 167.51 , 165.74 , 163.35 , 160.85 (d, J = 12.5 Hz), 160.51 , 158.12 (d, J = 12.0 Hz), 154.30 , 150.88 , 145.33 , 133.78 , 130.57 , 130.22 , 126.71 , 126.59 – 126.28 (m), 120.32 , 111.17 (d, J = 20.6 Hz), 104.19 (t, J = 26.9 Hz), 75.14 (d, J = 5.4 Hz), 63.78 , 55.95 , 54.46 , 54.15 , 44.57 , 43.00 , 42.21 , 39.90 (dp, J = 41.9, 21.0 Hz), 35.20 , 31.19 .

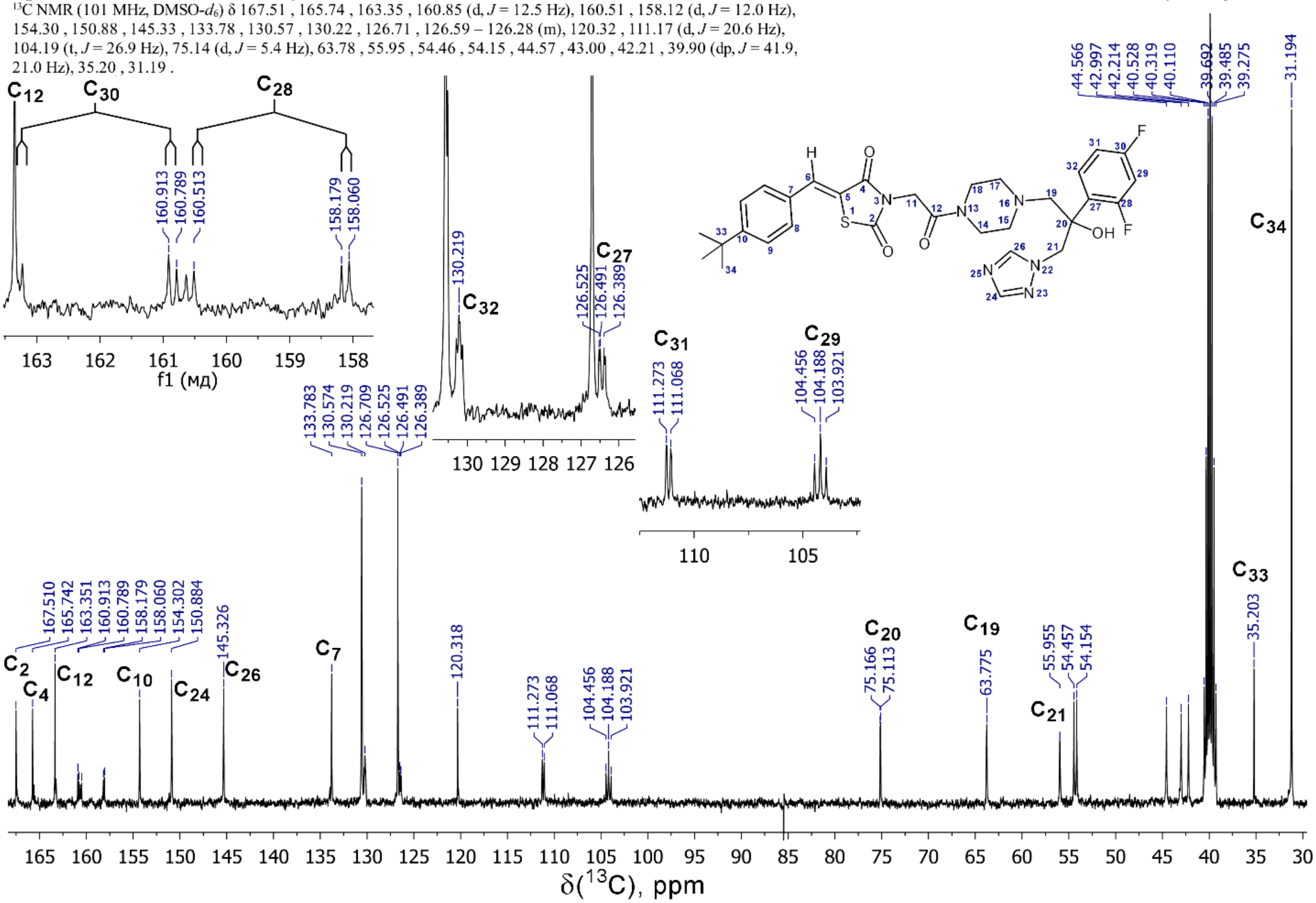

Figure S40. 1D ^{1}H NMR spectrum of **31g** recorded in DMSO-d$_6$ at 298K and 400 MHz proton resonance frequency.

^{1}H NMR (400 MHz, dmso) δ 8.28 (d, J = 4.7 Hz, 1H), 7.94 (d, J = 8.3 Hz, 2H), 7.83 (s, 1H), 7.74 (s, 1H), 7.60 (s, 2H), 7.40 (q, J = 8.6 Hz, 1H), 7.14 (ddd, J = 11.8, 9.0, 2.6 Hz, 1H), 6.95 (td, J = 8.5, 2.6 Hz, 1H), 5.73 (s, 1H), 4.56 (d, J = 10.4 Hz, 5H), 3.37 (d, J = 4.7 Hz, 2H), 2.87 (s, 7H), 2.71 (s, 6H), 2.41 (dd, J = 12.9, 5.9 Hz, 4H).

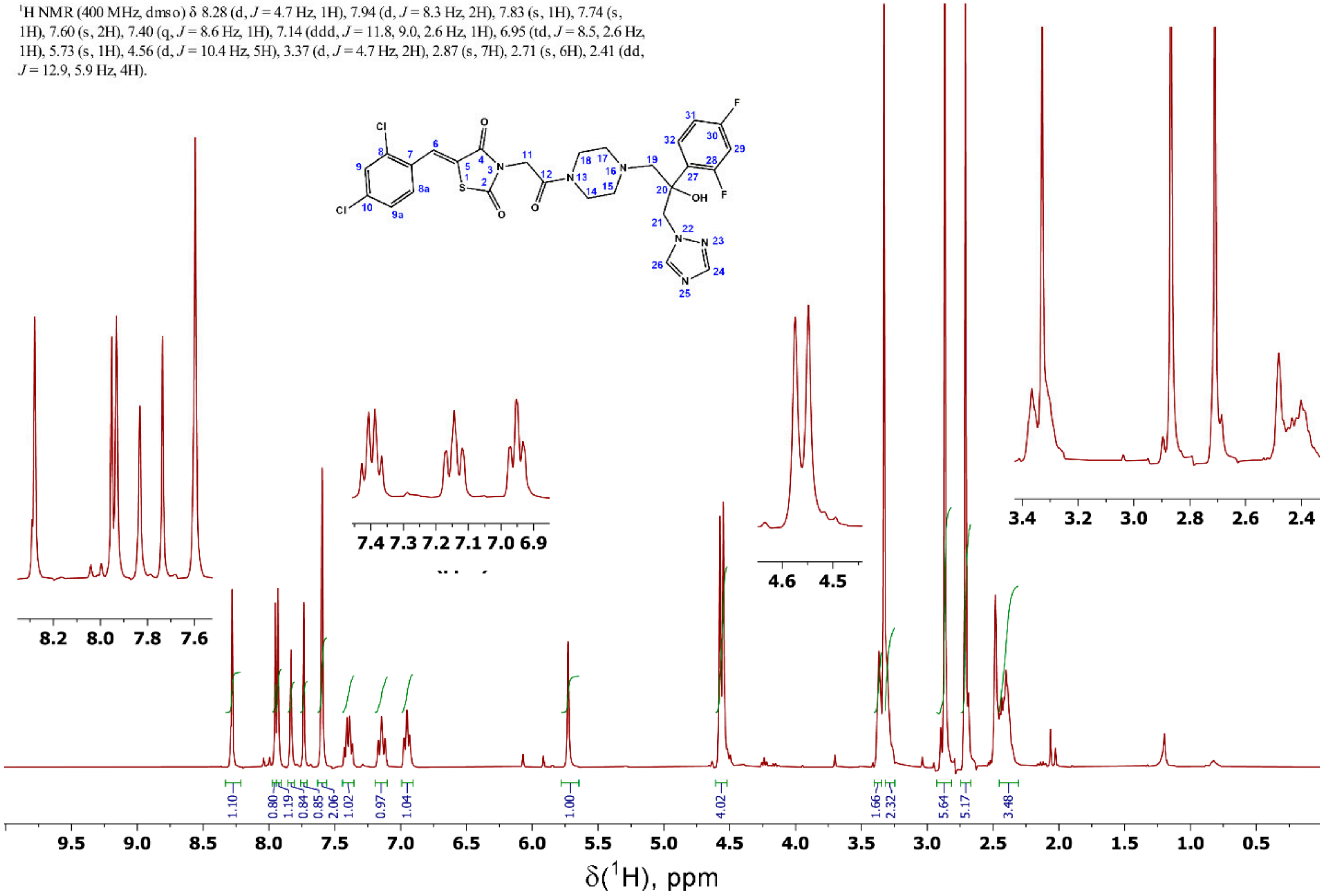

Figure S41. 1D ^{13}C NMR spectrum of **31g** recorded in DMSO-d$_6$ at 298K and 101 MHz carbon resonance frequency.

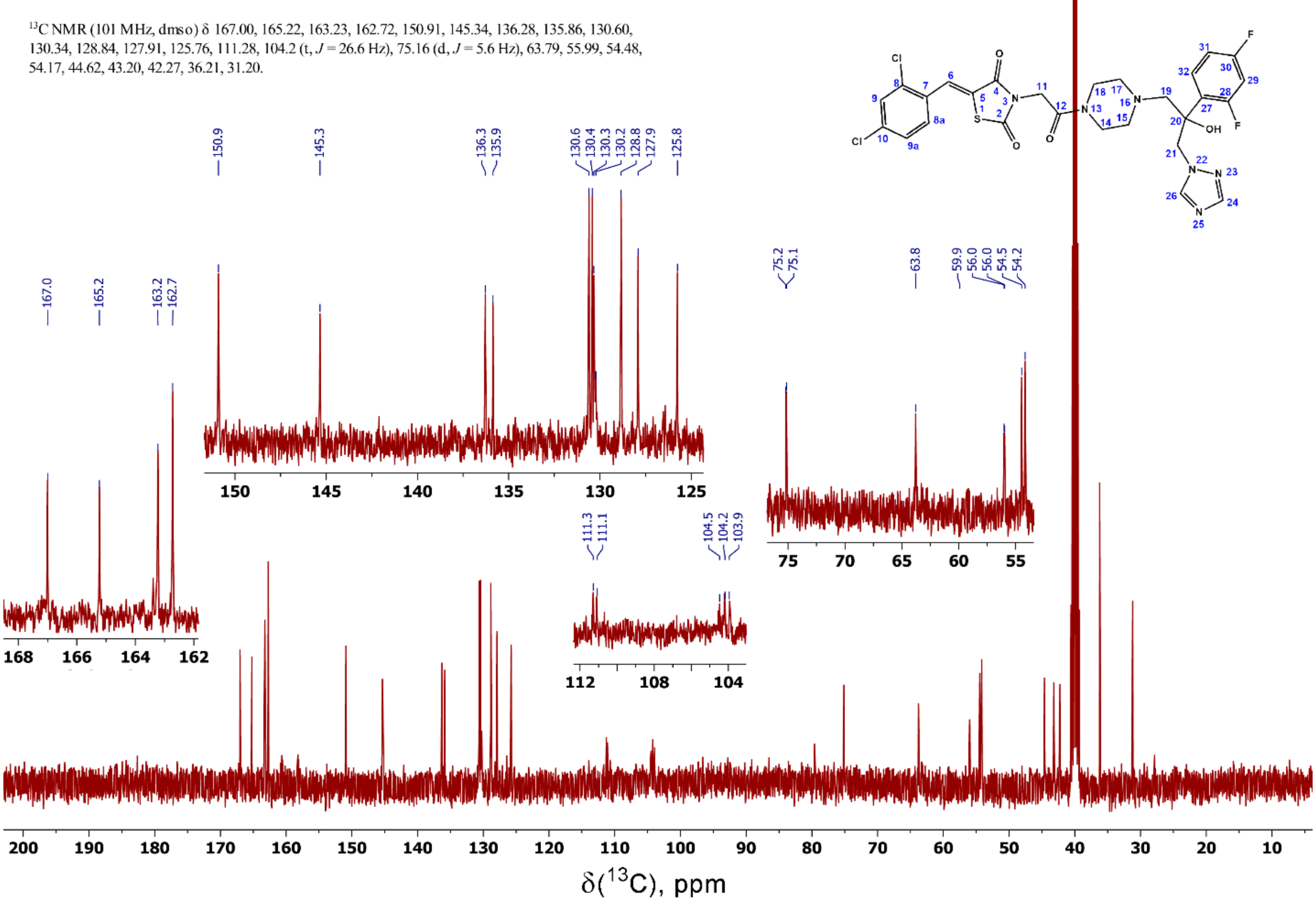

Figure S42. 1D ^{1}H NMR spectrum of **32a** recorded in DMSO-d6 at 298K and 700 MHz proton resonance frequency.

^{1}H NMR (700 MHz, DMSO-*d*$_6$) δ 8.26 (s, 1H), 7.92 – 7.90 (m, 1H), 7.72 (s, 1H), 7.64 (d, *J* = 8.4 Hz, 2H), 7.60 (d, *J* = 8.3 Hz, 2H), 7.37 (q, *J* = 8.3 Hz, 1H), 7.16 – 7.09 (m, 1H), 6.93 (td, *J* = 8.4, 2.6 Hz, 1H), 5.69 (d, *J* = 2.5 Hz, 1H), 5.19 (qd, *J* = 7.2, 2.9 Hz, 1H), 4.55 (s, 2H), 3.33 (s, 1H), 3.30 (s, 3H), 3.20 (s, 2H), 2.83 (dd, *J* = 14.0, 4.9 Hz, 1H), 2.67 (dd, *J* = 14.0, 4.4 Hz, 1H), 2.45 – 2.40 (m, 3H), 2.33 (s, 2H), 2.29 (s, 1H), 1.49 (dd, *J* = 7.2, 4.0 Hz, 3H), 1.02 (d, *J* = 6.0 Hz, 1H).

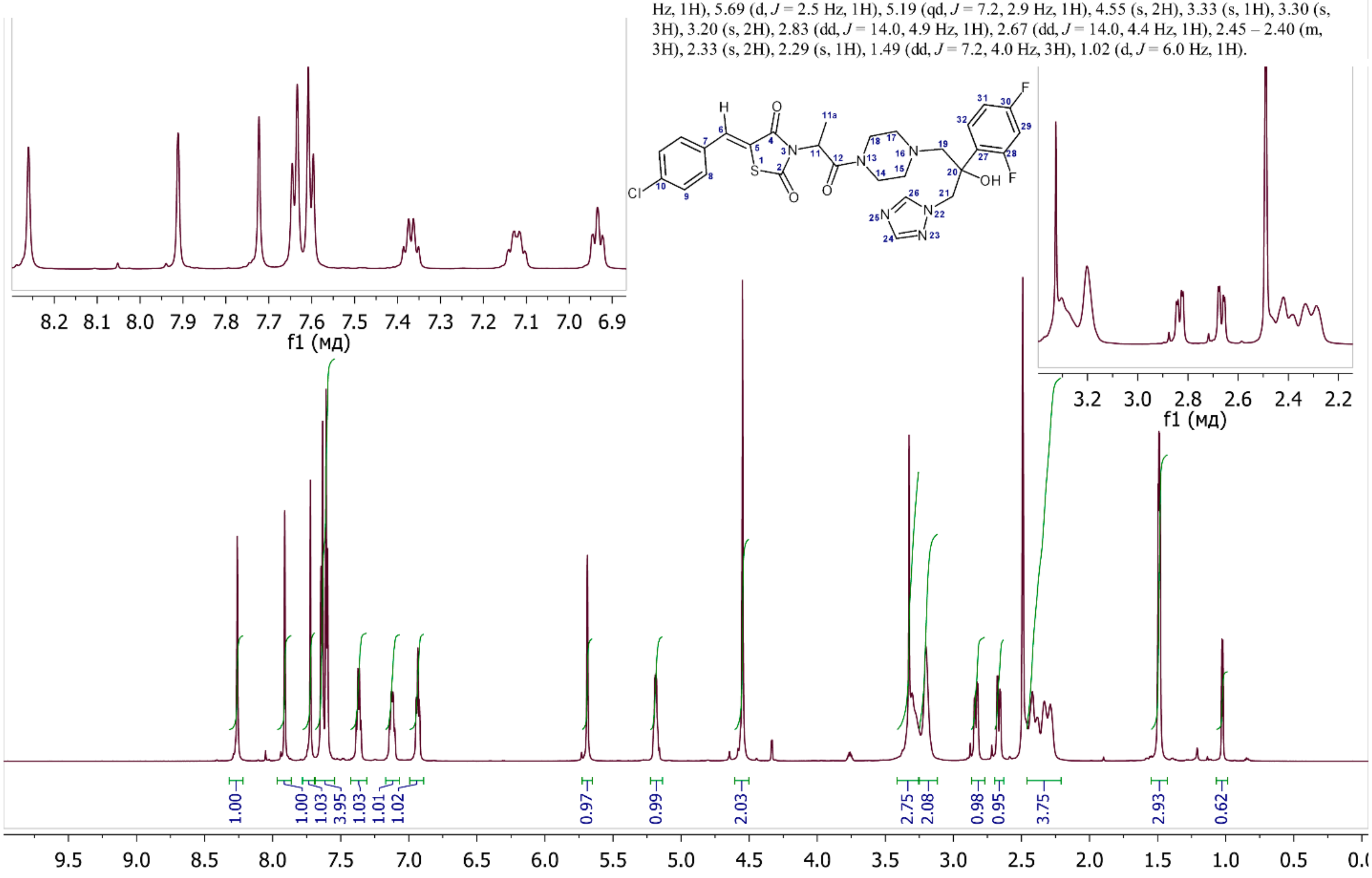

Figure S43. 1D ^{13}C NMR spectrum of **32a** recorded in DMSO-d_6 at 298K and 176 MHz carbon resonance frequency.

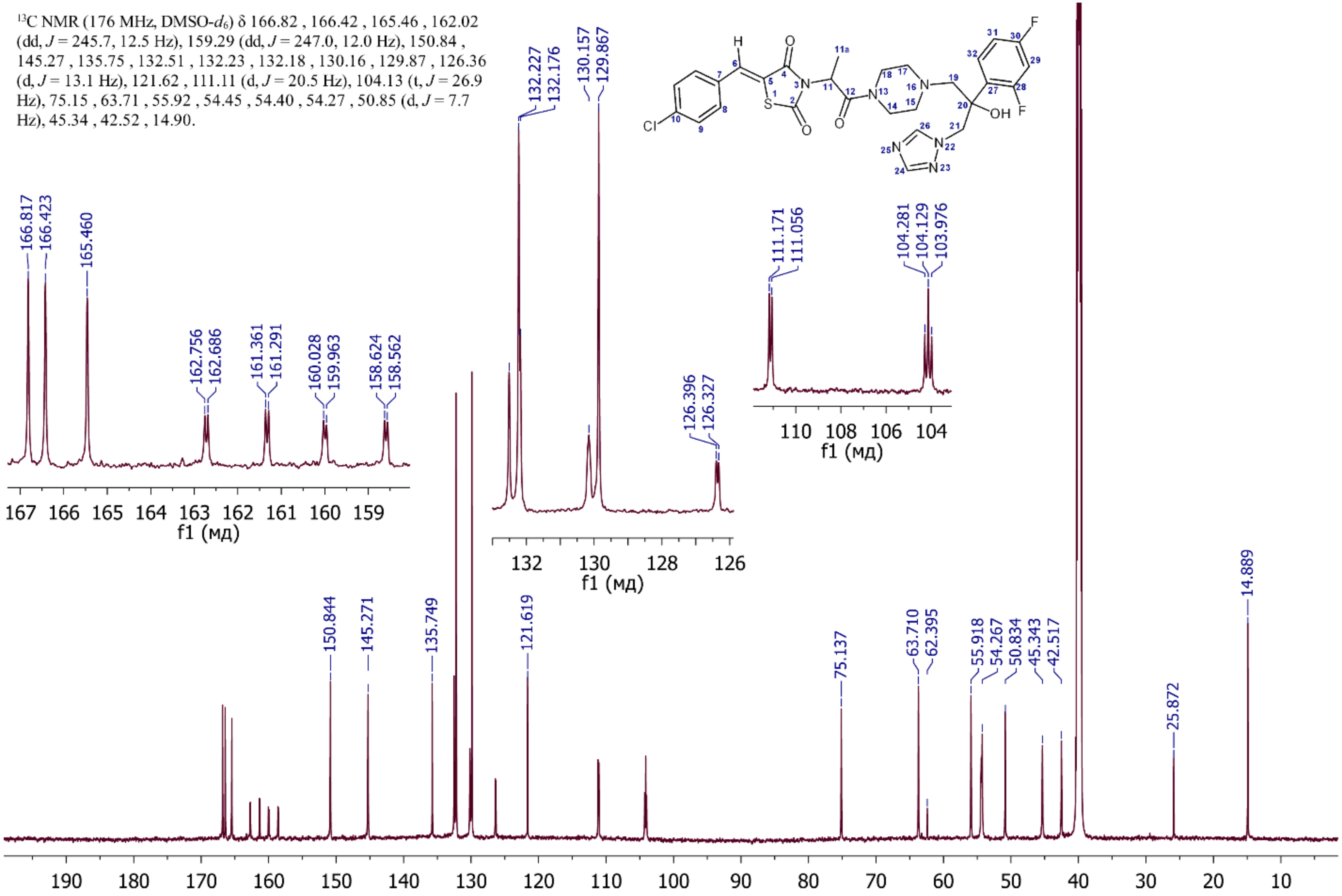

Figure S44. 1D ^{1}H NMR spectrum of **33a** recorded in DMSO-d$_6$ at 298K and 700 MHz proton resonance frequency.

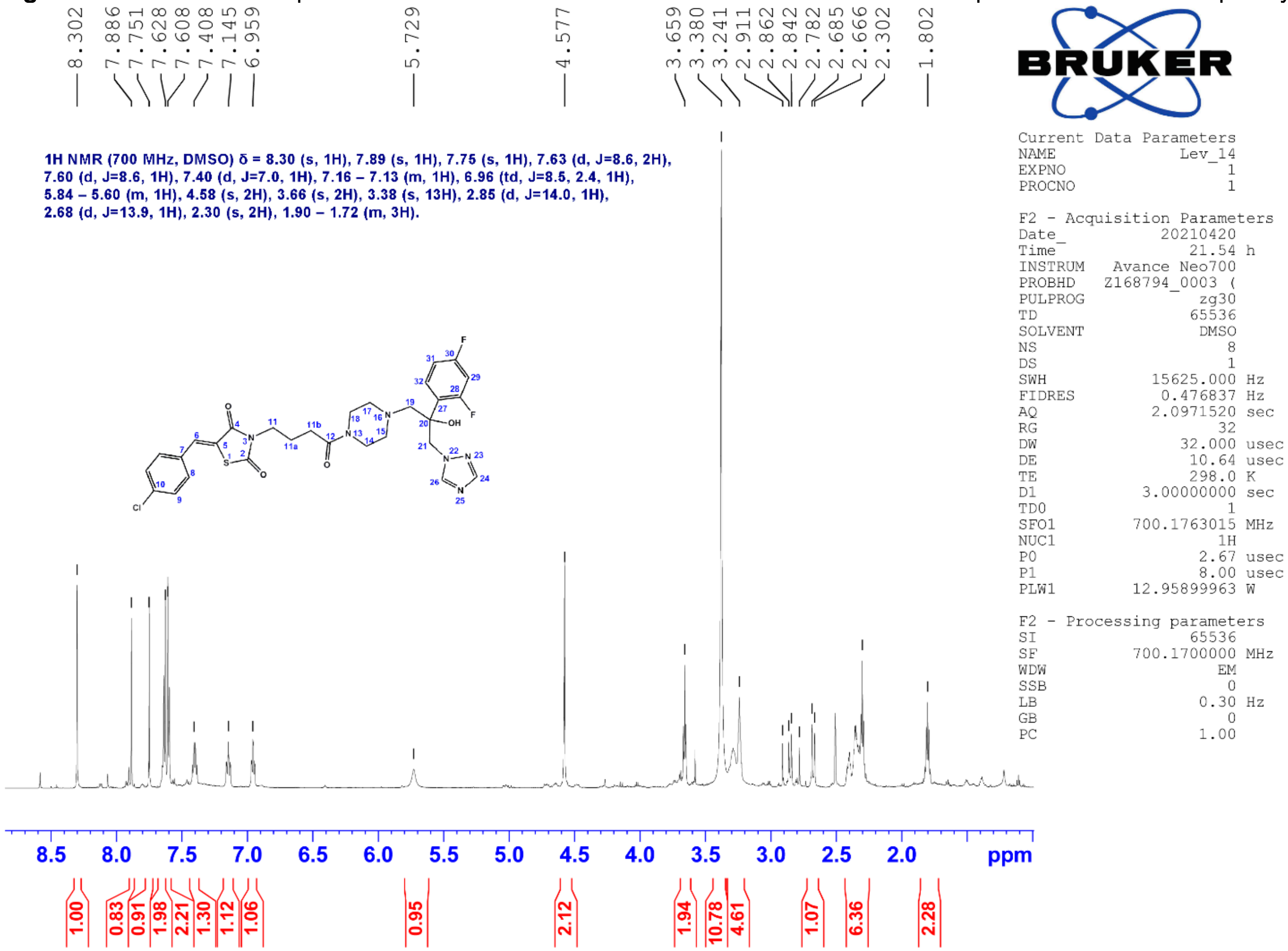

Figure S45. 1D ^{13}C NMR spectrum of **33a** recorded in DMSO-d$_6$ at 298K and 176 MHz carbon resonance frequency.

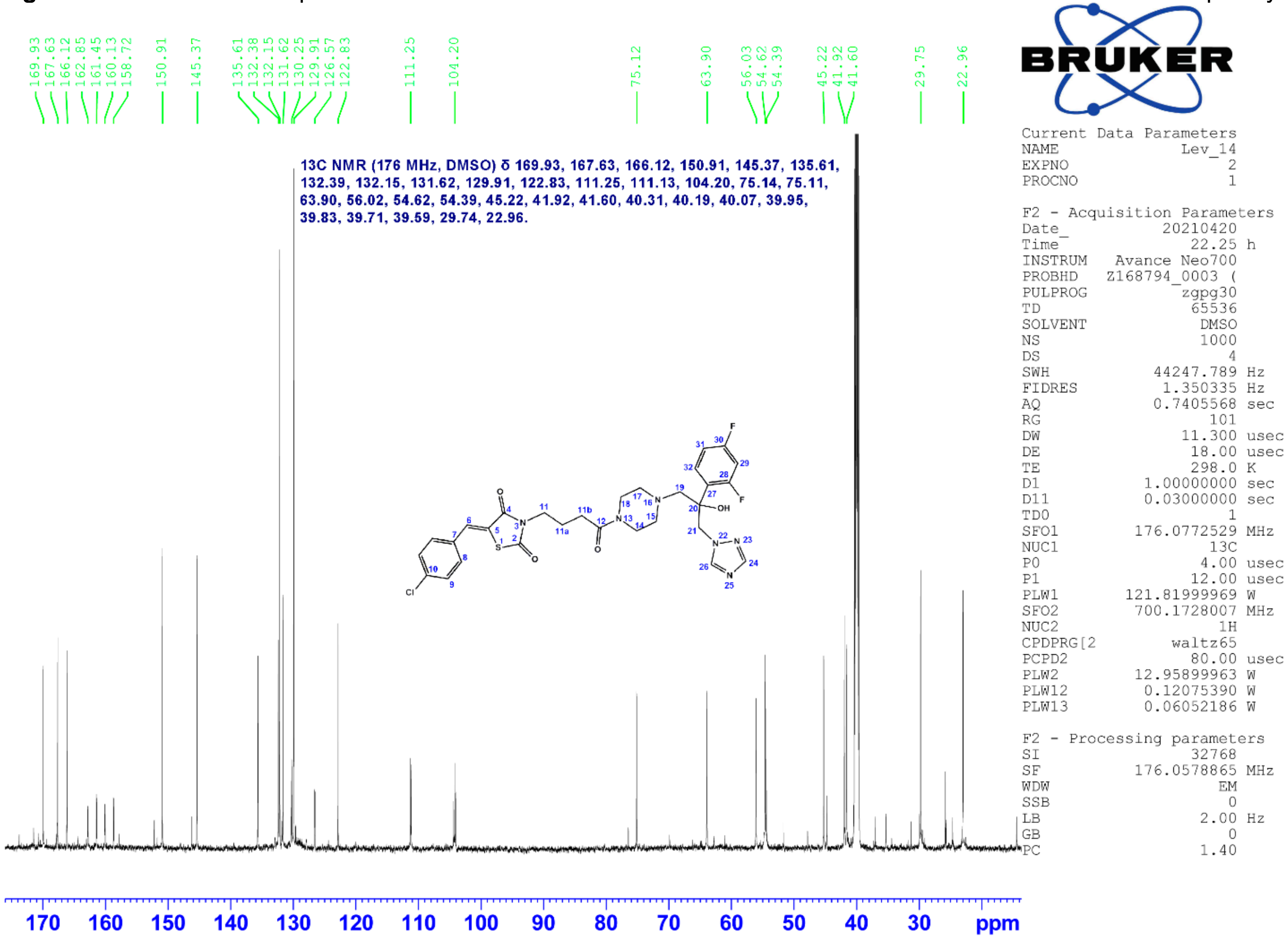

Figure S46. 1D ^{1}H NMR spectrum of **33b** recorded in DMSO-d_6 at 298K and 700 MHz proton resonance frequency.

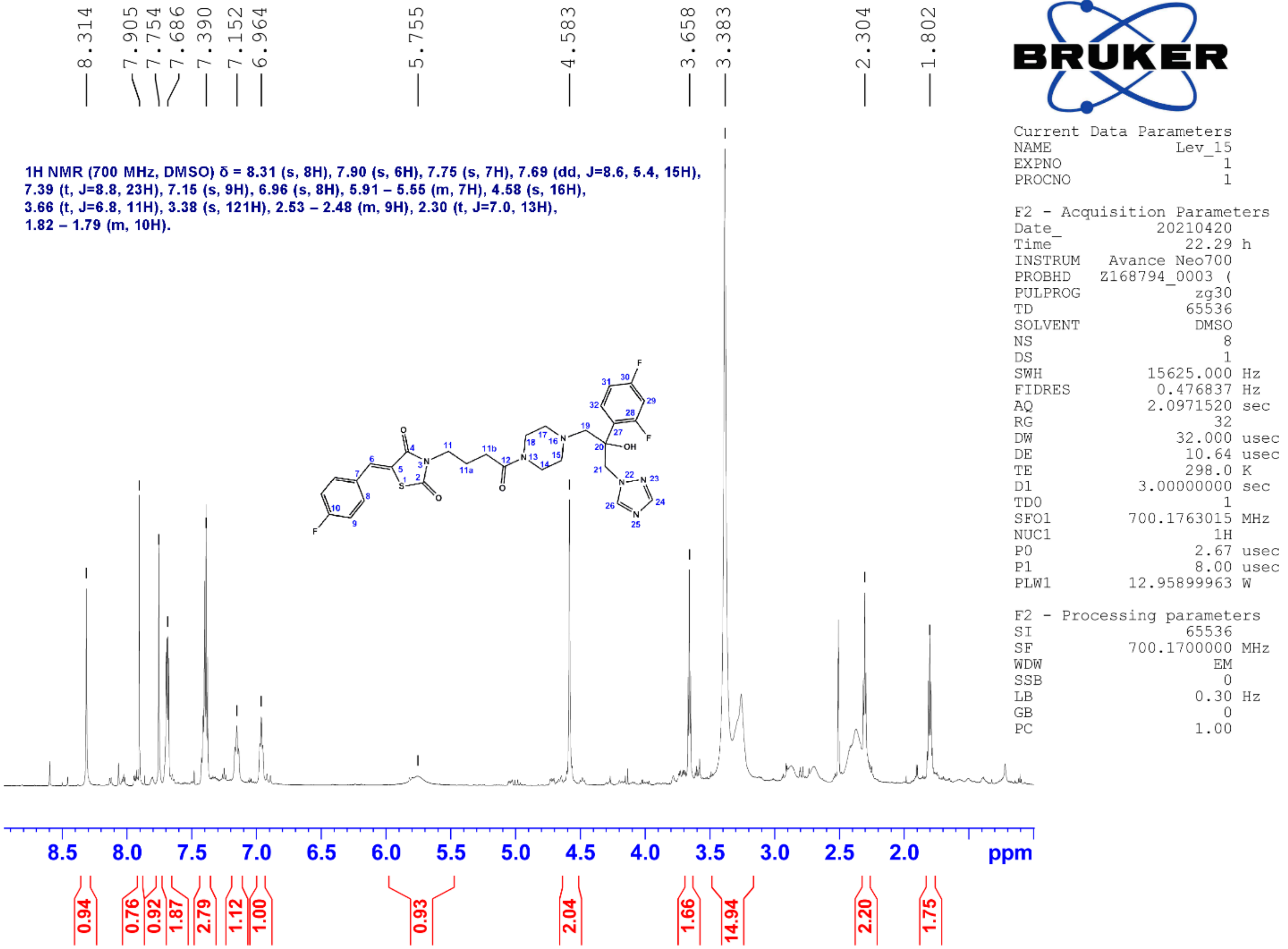

Figure S47. 1D ^{13}C NMR spectrum of **33b** recorded in DMSO-d$_6$ at 298K and 176 MHz carbon resonance frequency.

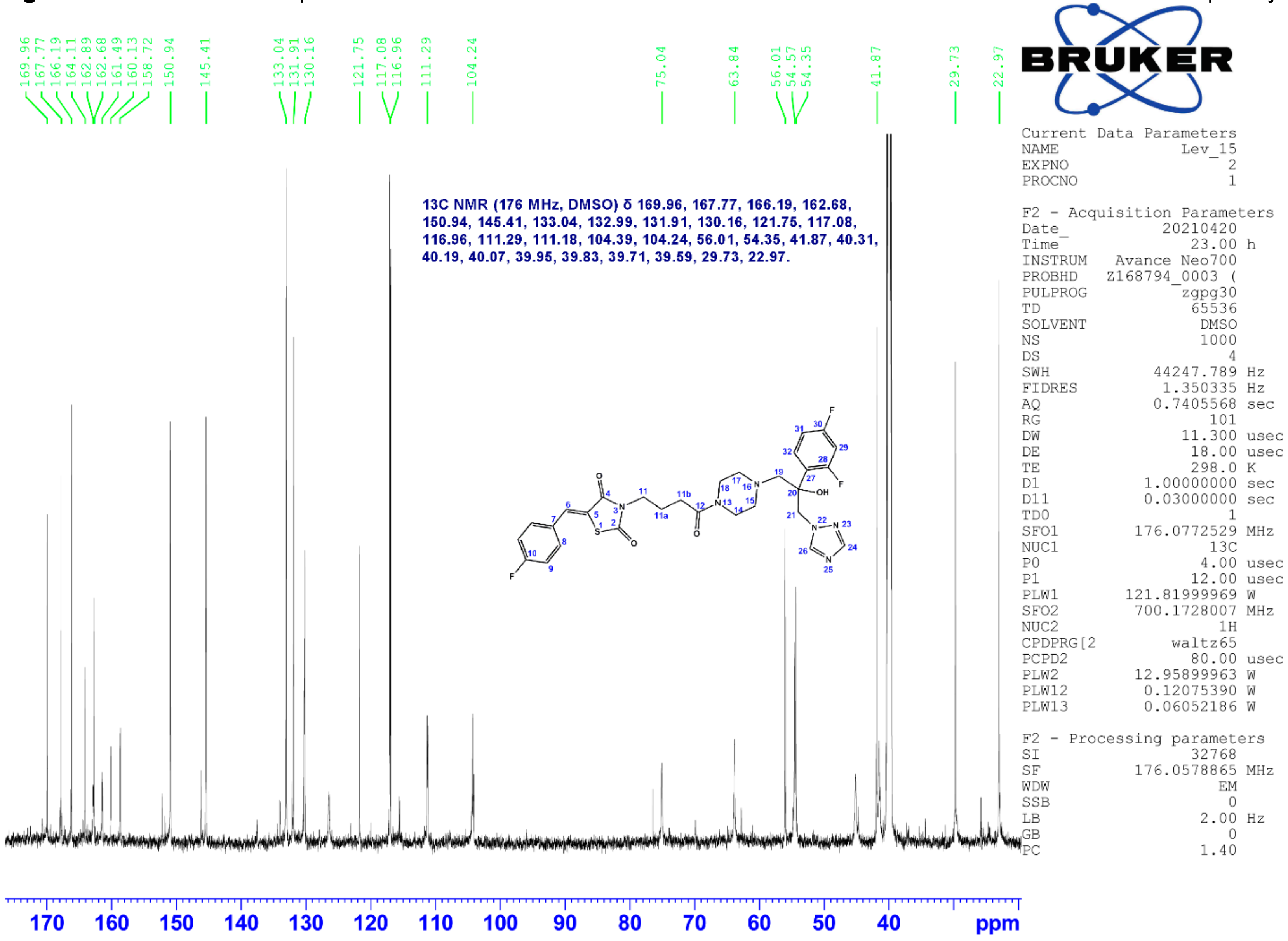

Figure S48. 1D ^{1}H NMR spectrum of **33g** recorded in DMSO-d_6 at 298K and 700 MHz proton resonance frequency.

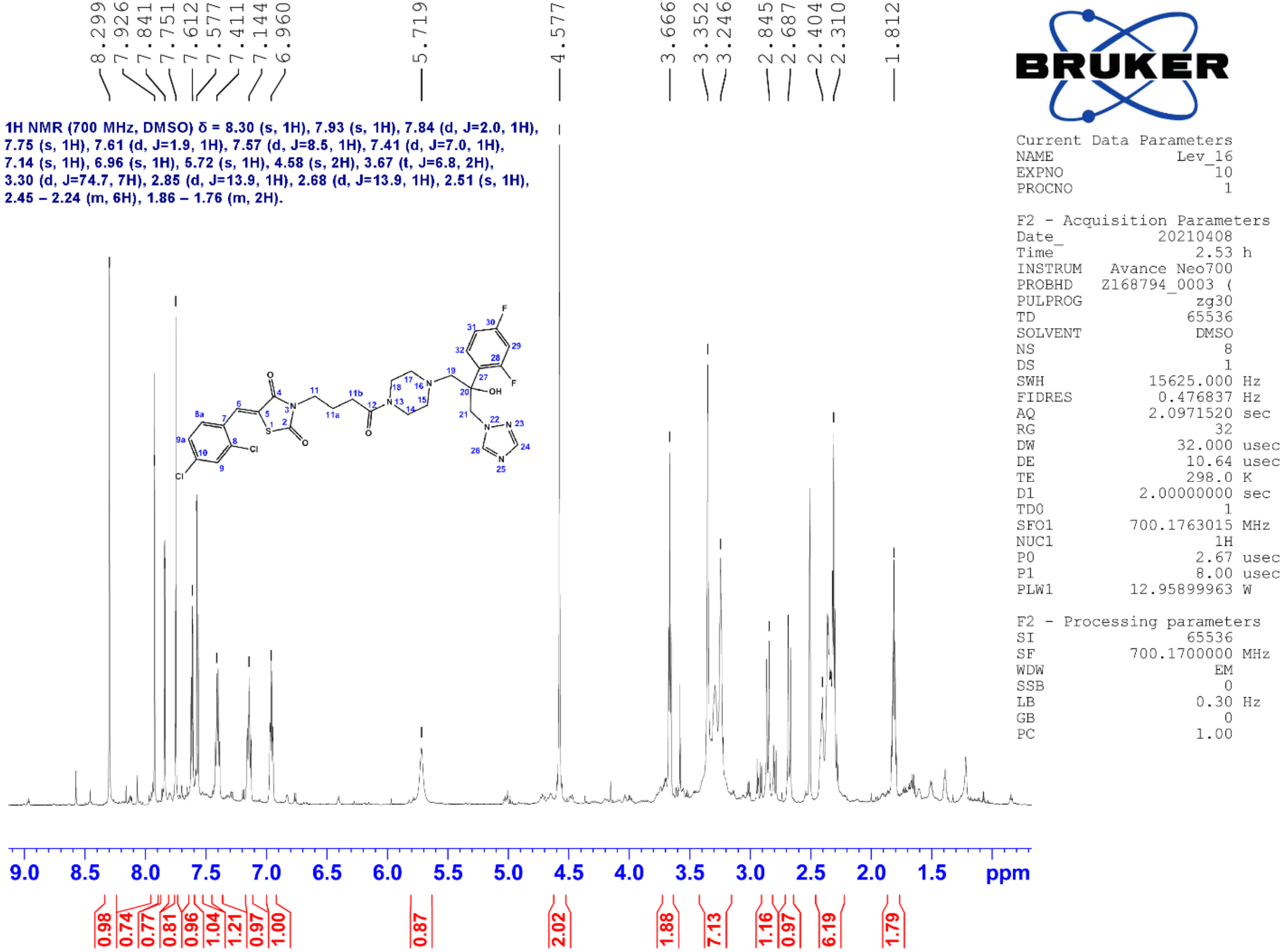

Figure S49. 1D ^{13}C NMR spectrum of **33g** recorded in DMSO-d_6 at 298K and 176 MHz carbon resonance frequency.

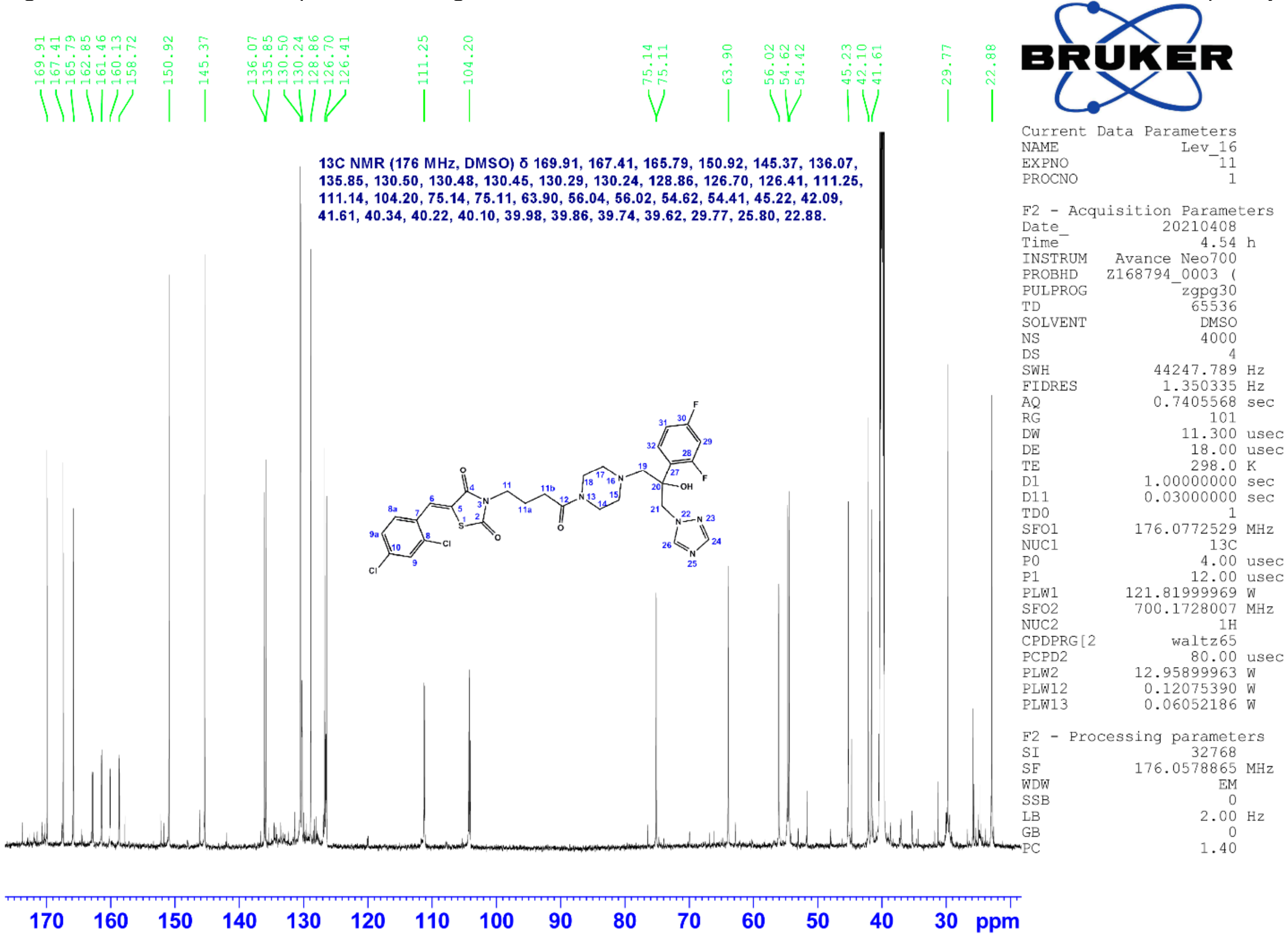

Figure S50. 1D ^{1}H NMR spectrum of **33h** recorded in DMSO-d_6 at 298K and 700 MHz proton resonance frequency.

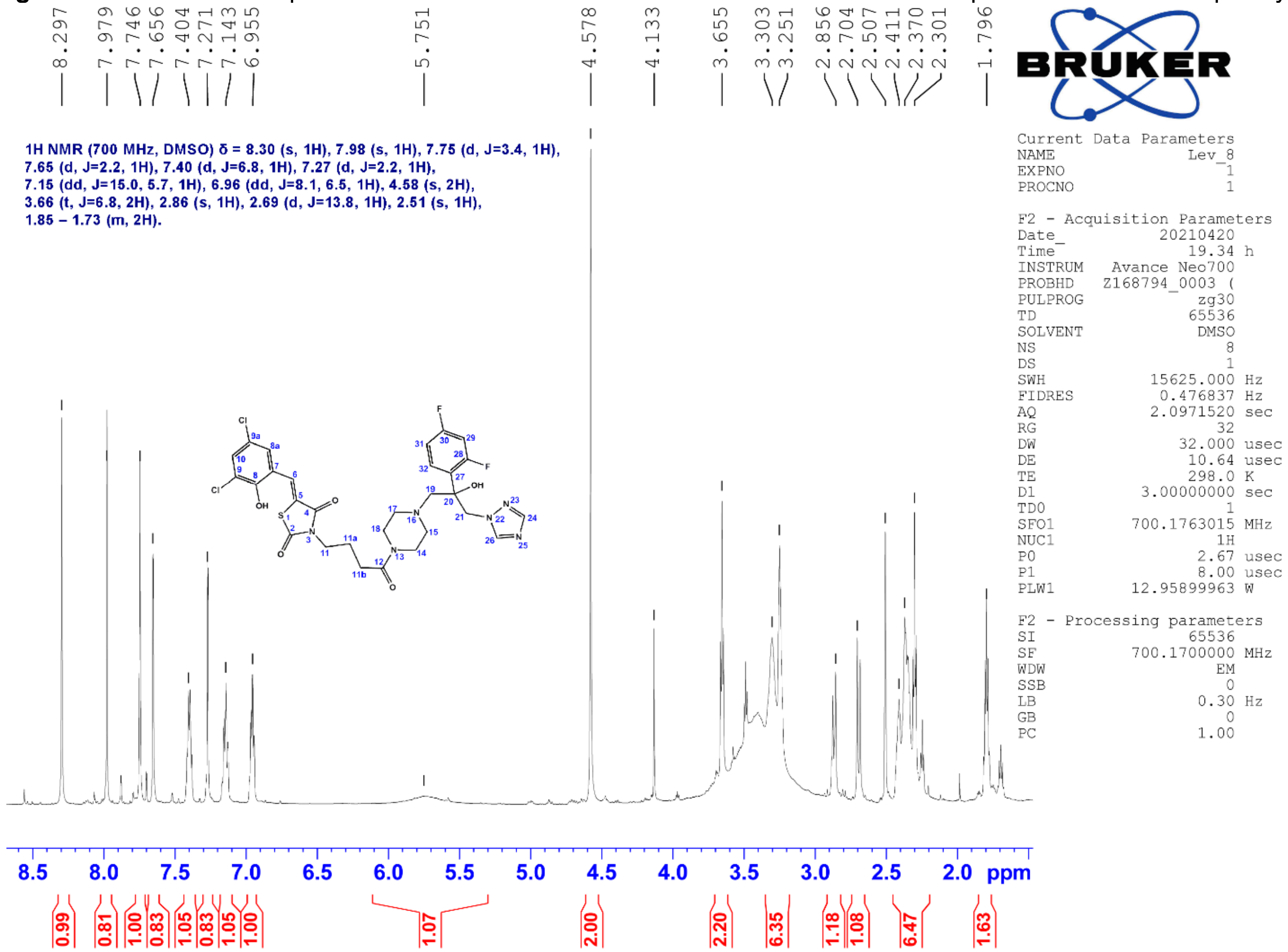

Figure S51. 1D ^{13}C NMR spectrum of **33h** recorded in DMSO-d_6 at 298K and 176 MHz carbon resonance frequency.

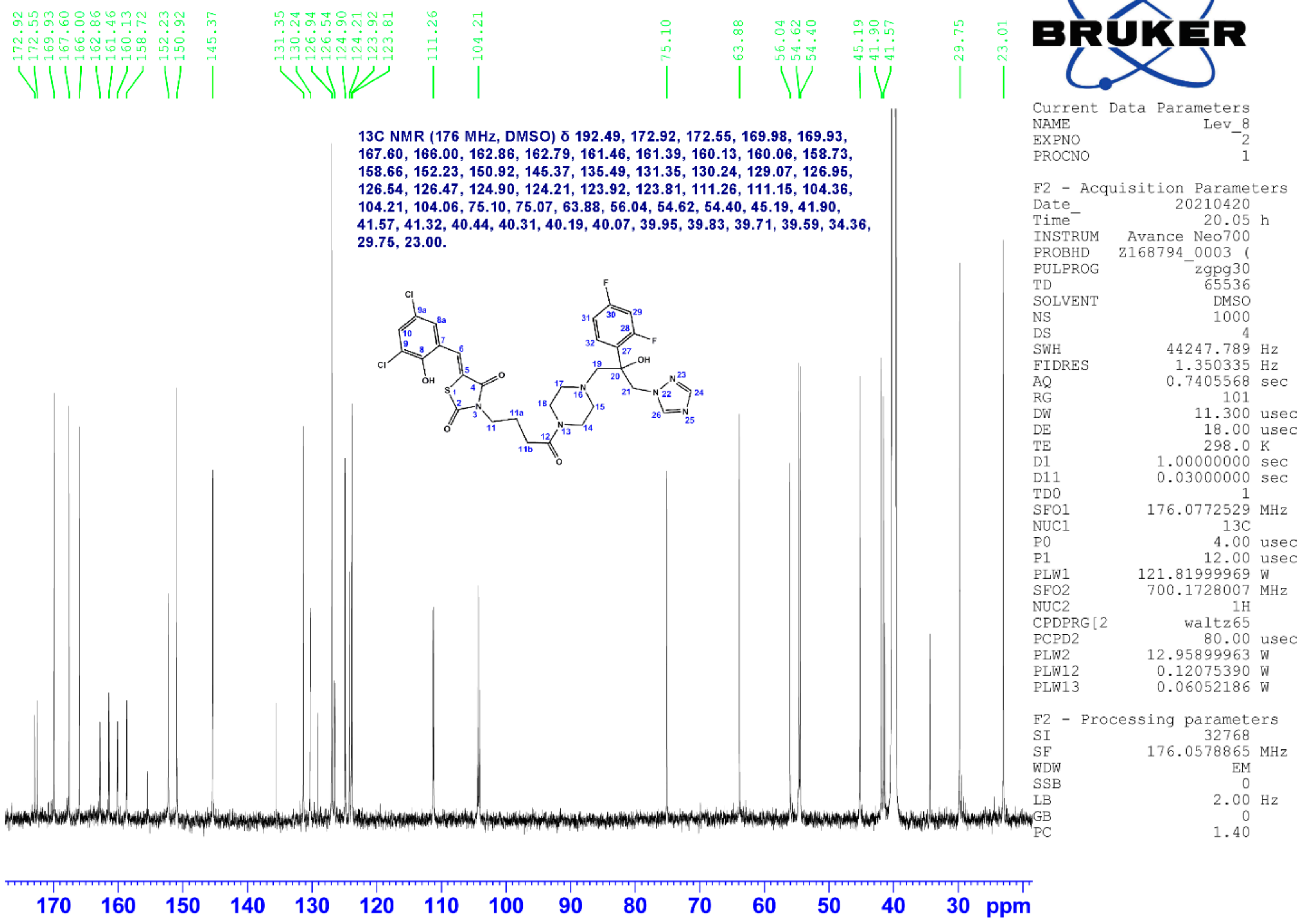

Figure S52. 1D ^{1}H NMR spectrum of **33i** recorded in DMSO-d$_6$ at 298K and 700 MHz proton resonance frequency.

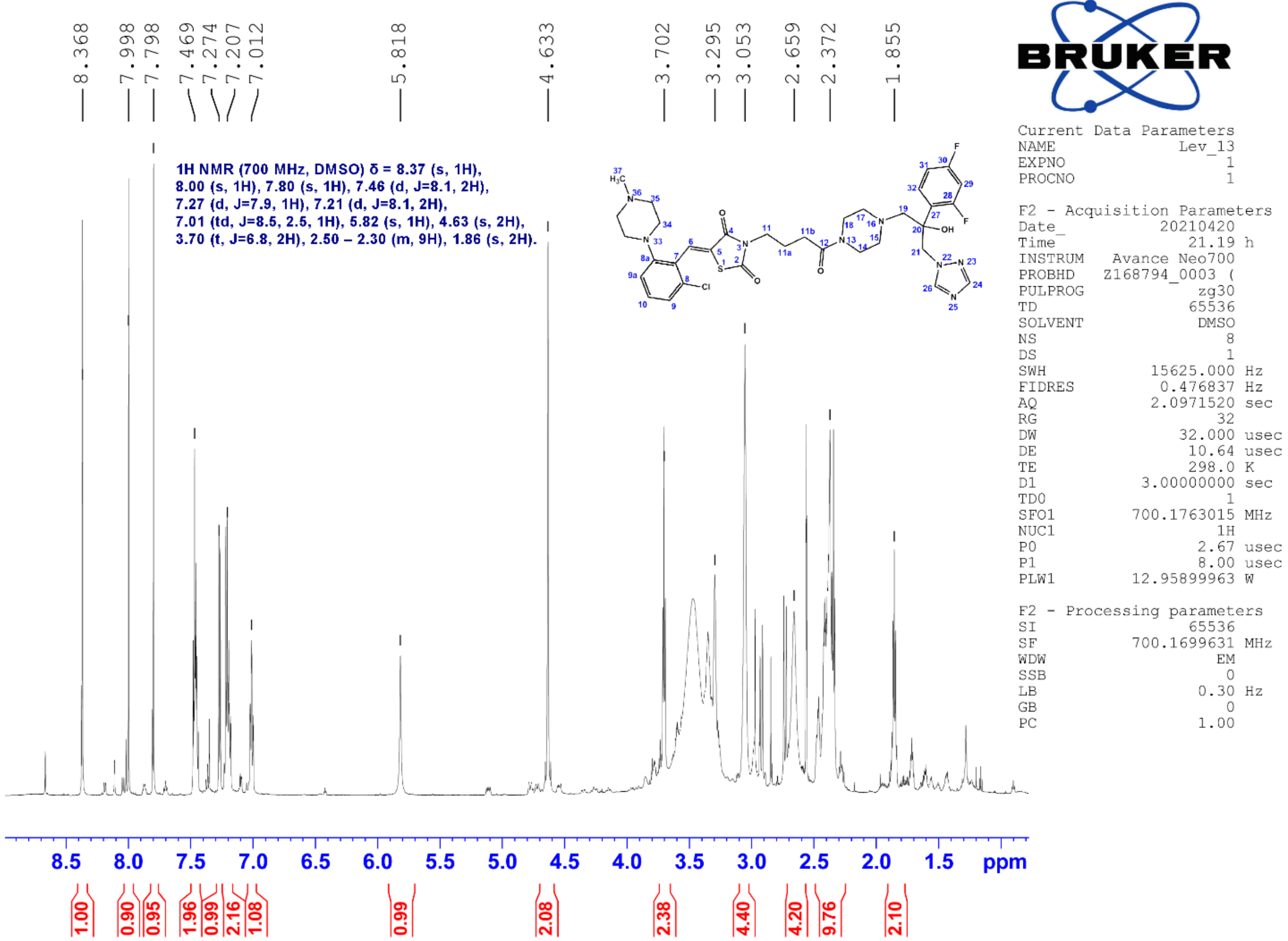

Figure S53. 1D ^{13}C NMR spectrum of **33i** recorded in DMSO-d_6 at 298K and 176 MHz carbon resonance frequency.

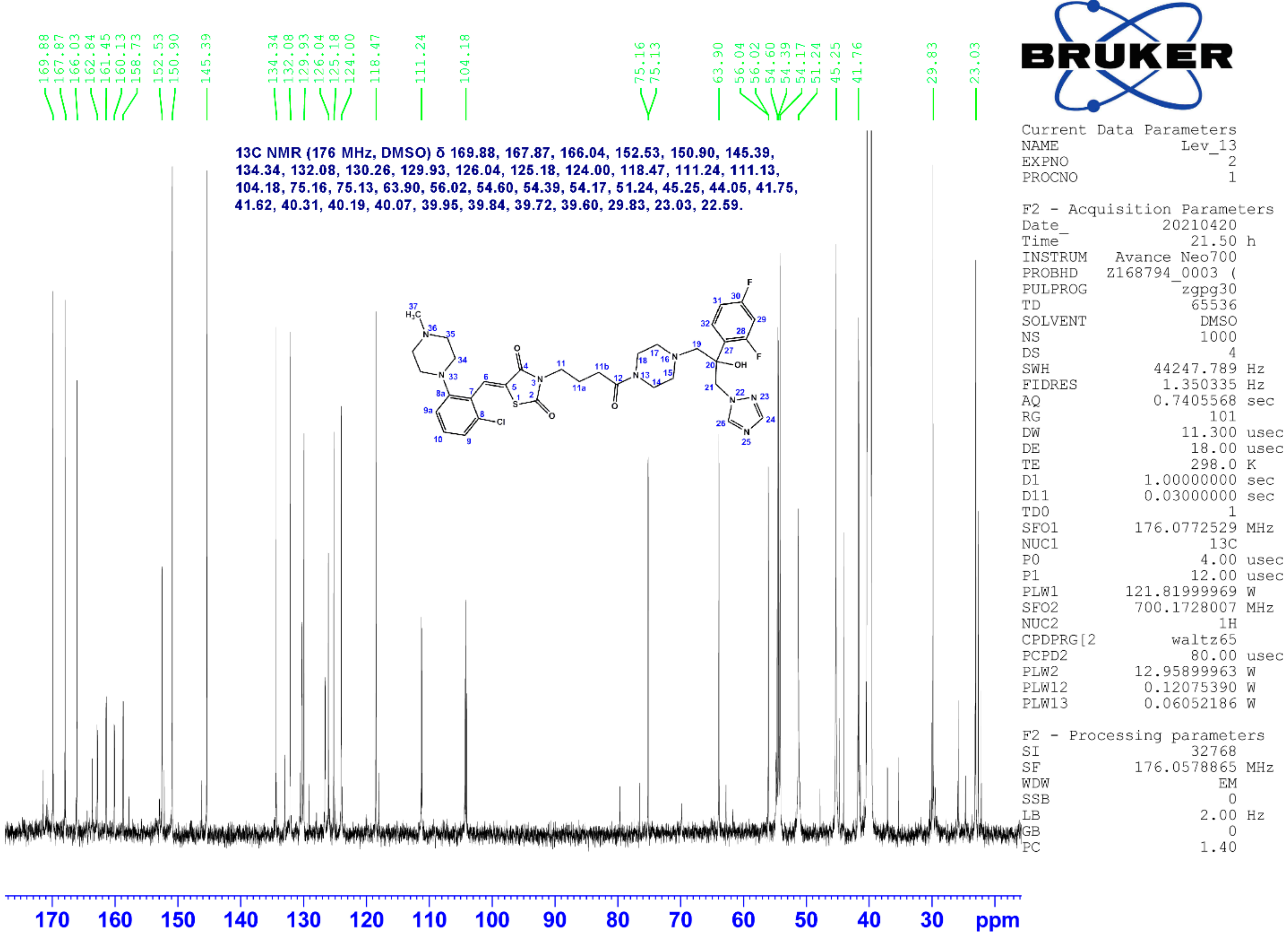

Figure S54. 1D ^{1}H NMR spectrum of **34a** recorded in DMSO-d$_6$ at 298K and 700 MHz proton resonance frequency.

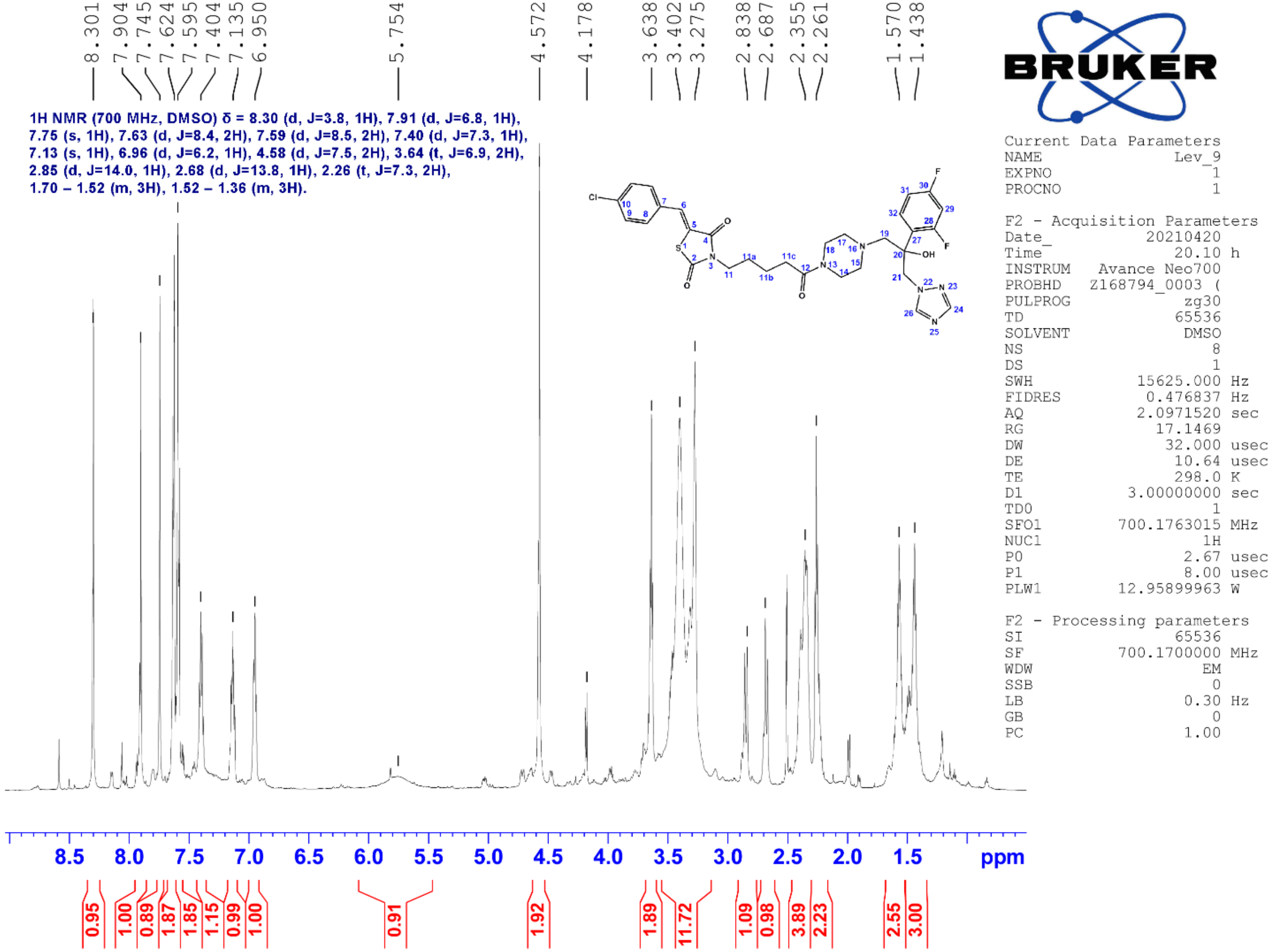

Figure S55. 1D ^{13}C NMR spectrum of **34a** recorded in DMSO-d_6 at 298K and 176 MHz carbon resonance frequency.

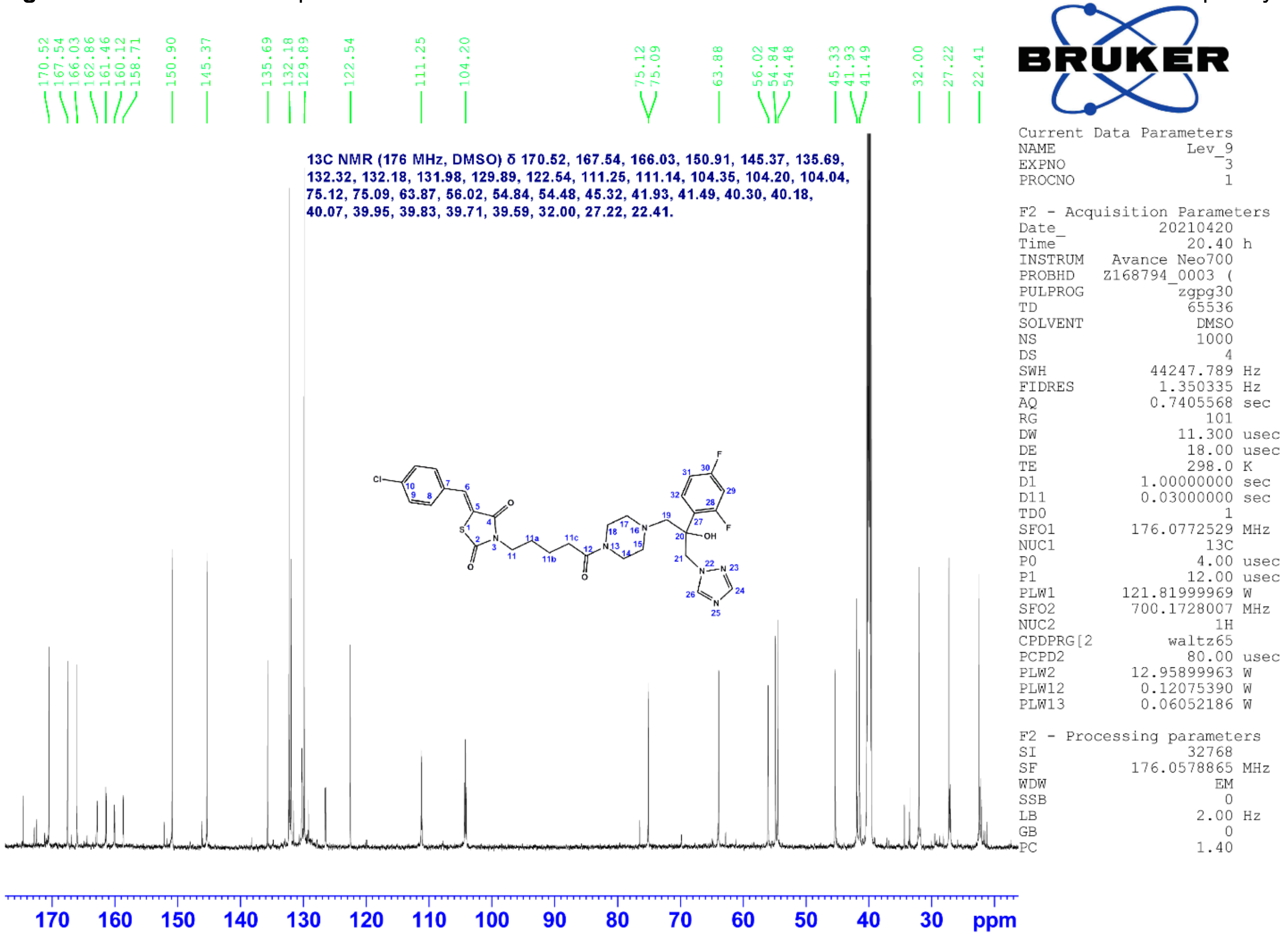

Figure S56. 1D ^{1}H NMR spectrum of **34b** recorded in DMSO-d6 at 298K and 700 MHz proton resonance frequency.

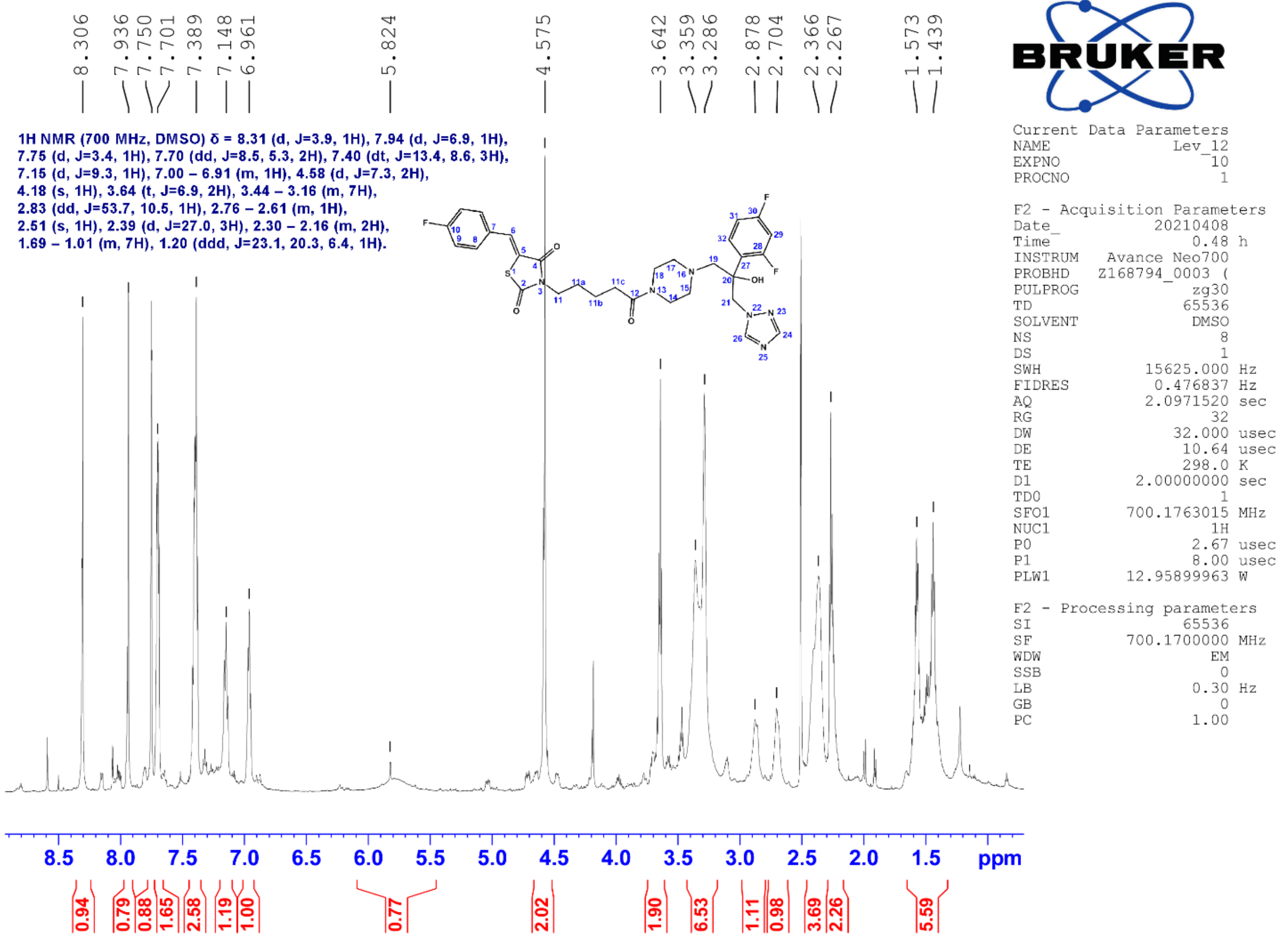

Figure S57. 1D ^{13}C NMR spectrum of **34b** recorded in DMSO-d_6 at 298K and 176 MHz carbon resonance frequency.

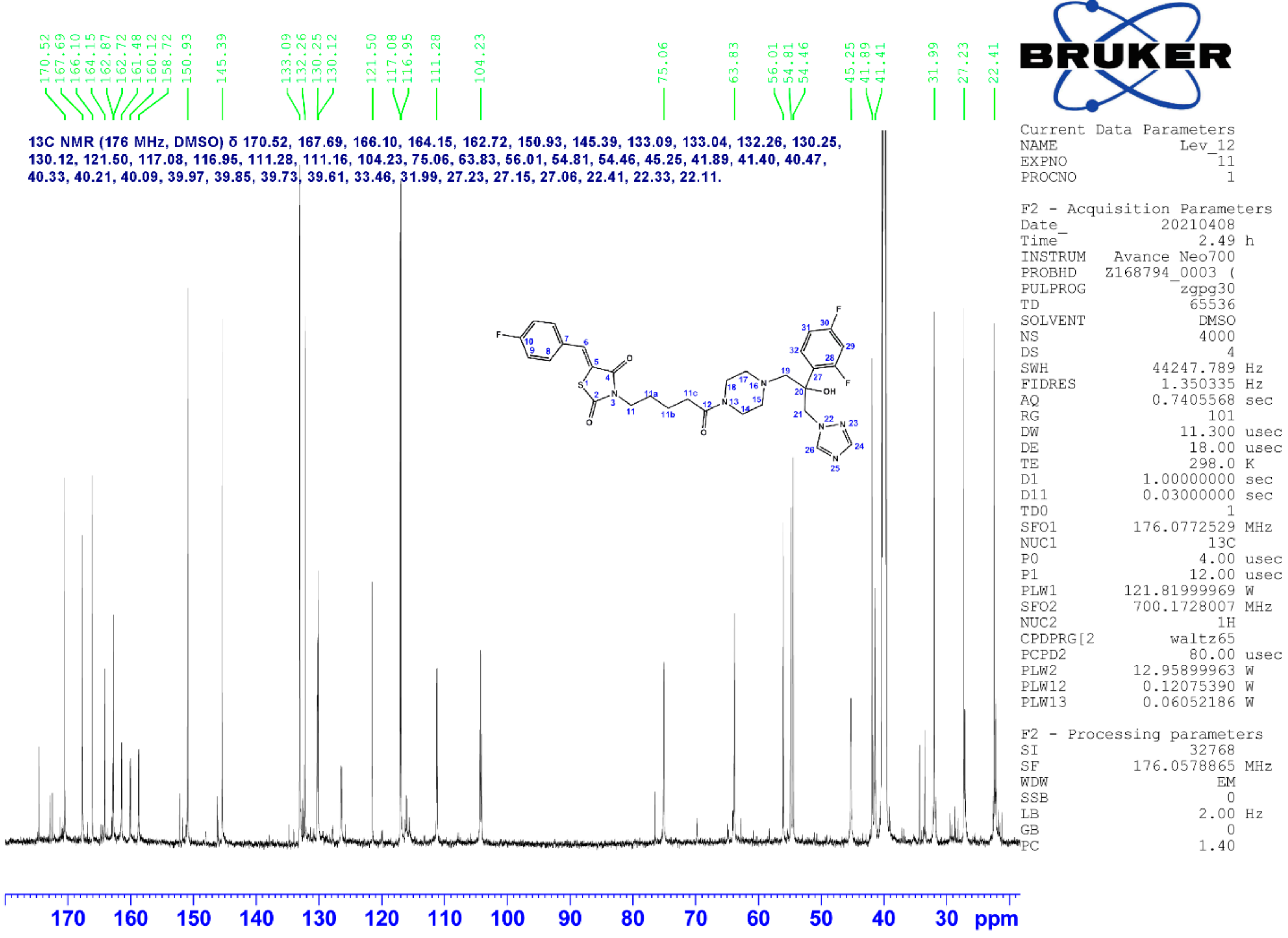

Figure S58. 1D ^{1}H NMR spectrum of **34g** recorded in DMSO-d_6 at 298K and 700 MHz proton resonance frequency.

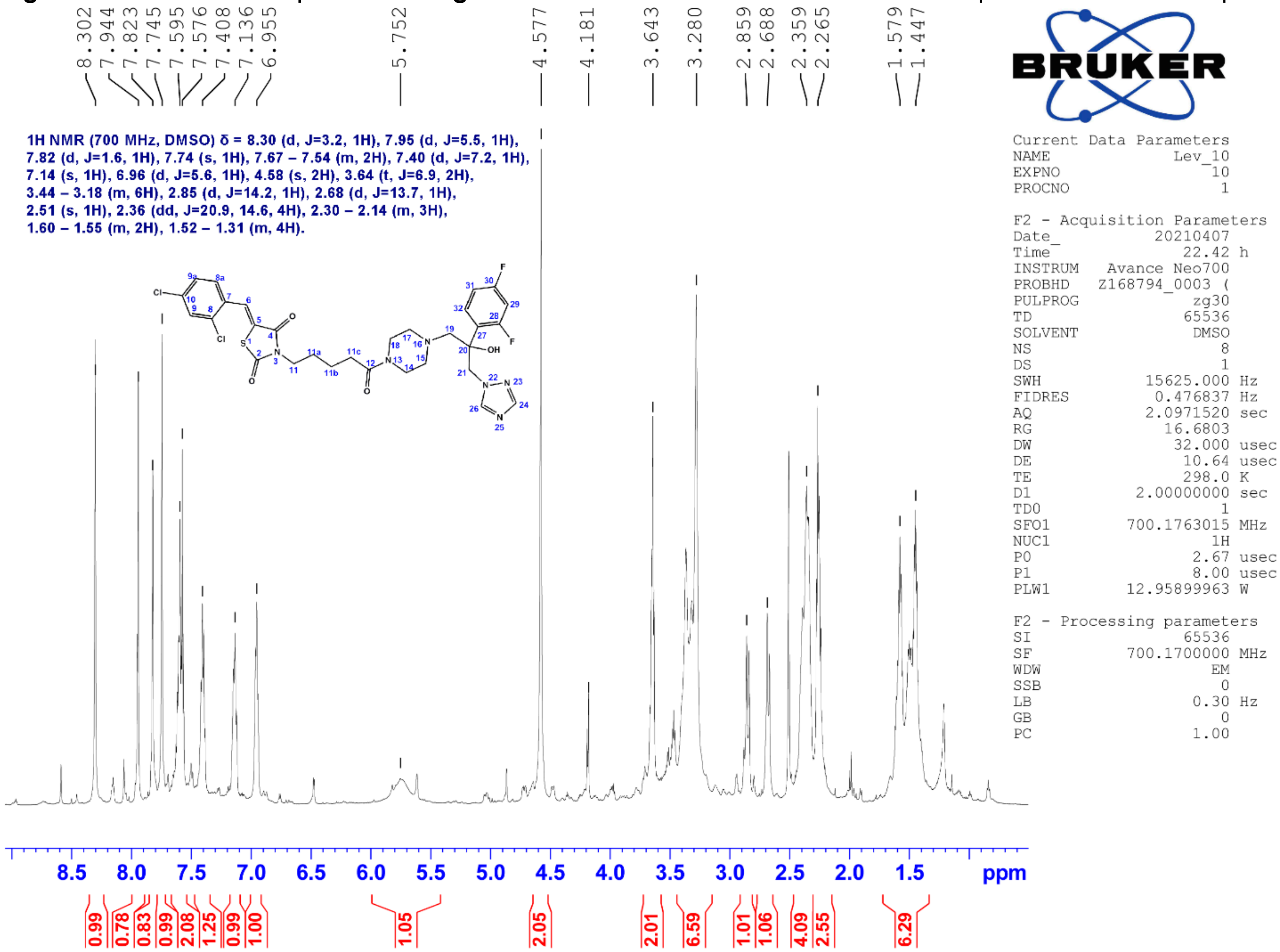

Figure S59. 1D ^{13}C NMR spectrum of **34g** recorded in DMSO-d_6 at 298K and 176 MHz carbon resonance frequency.

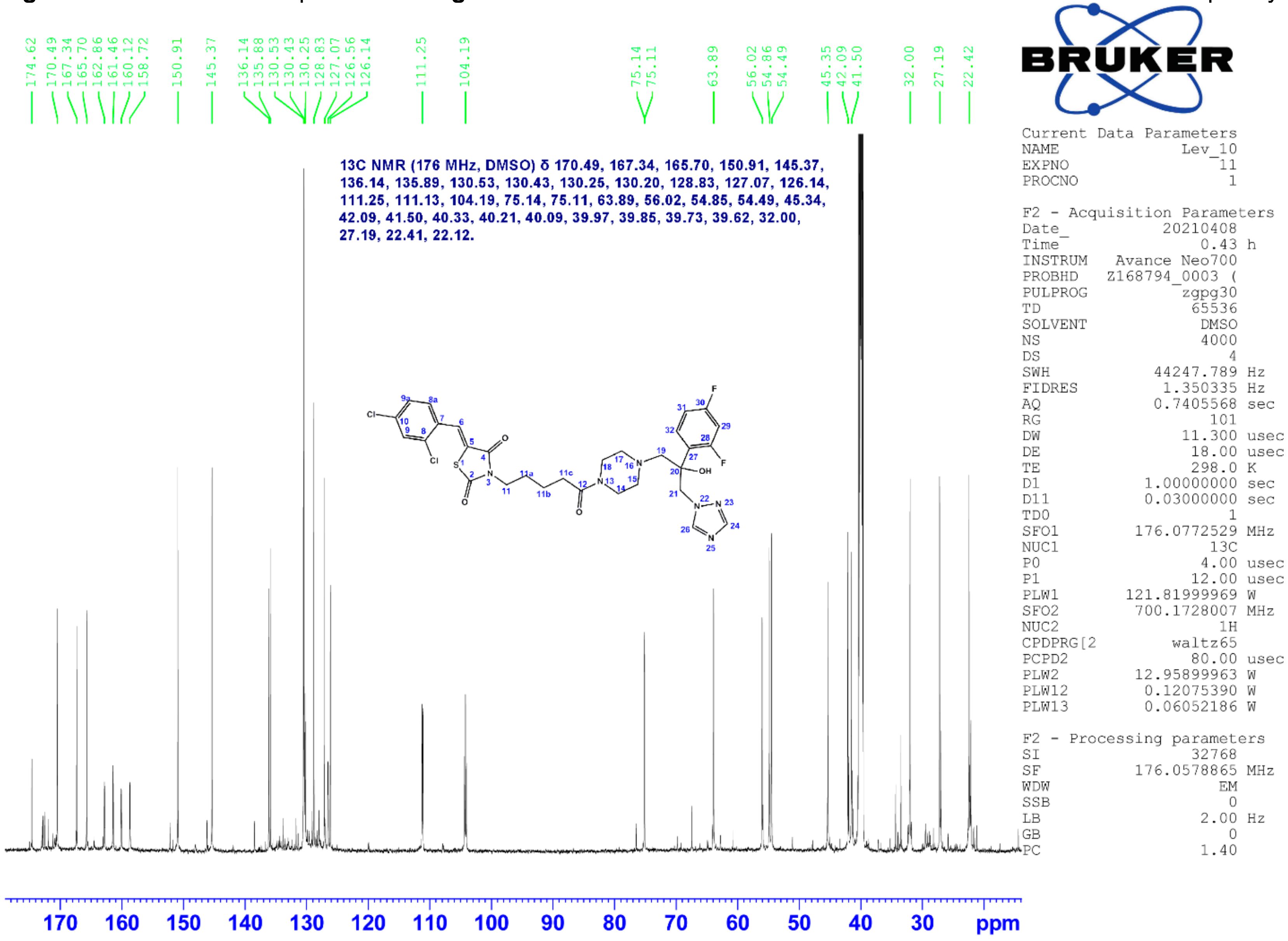

Figure S60. 1D ^{1}H NMR spectrum of **34h** recorded in DMSO-d_6 at 298K and 700 MHz proton resonance frequency.

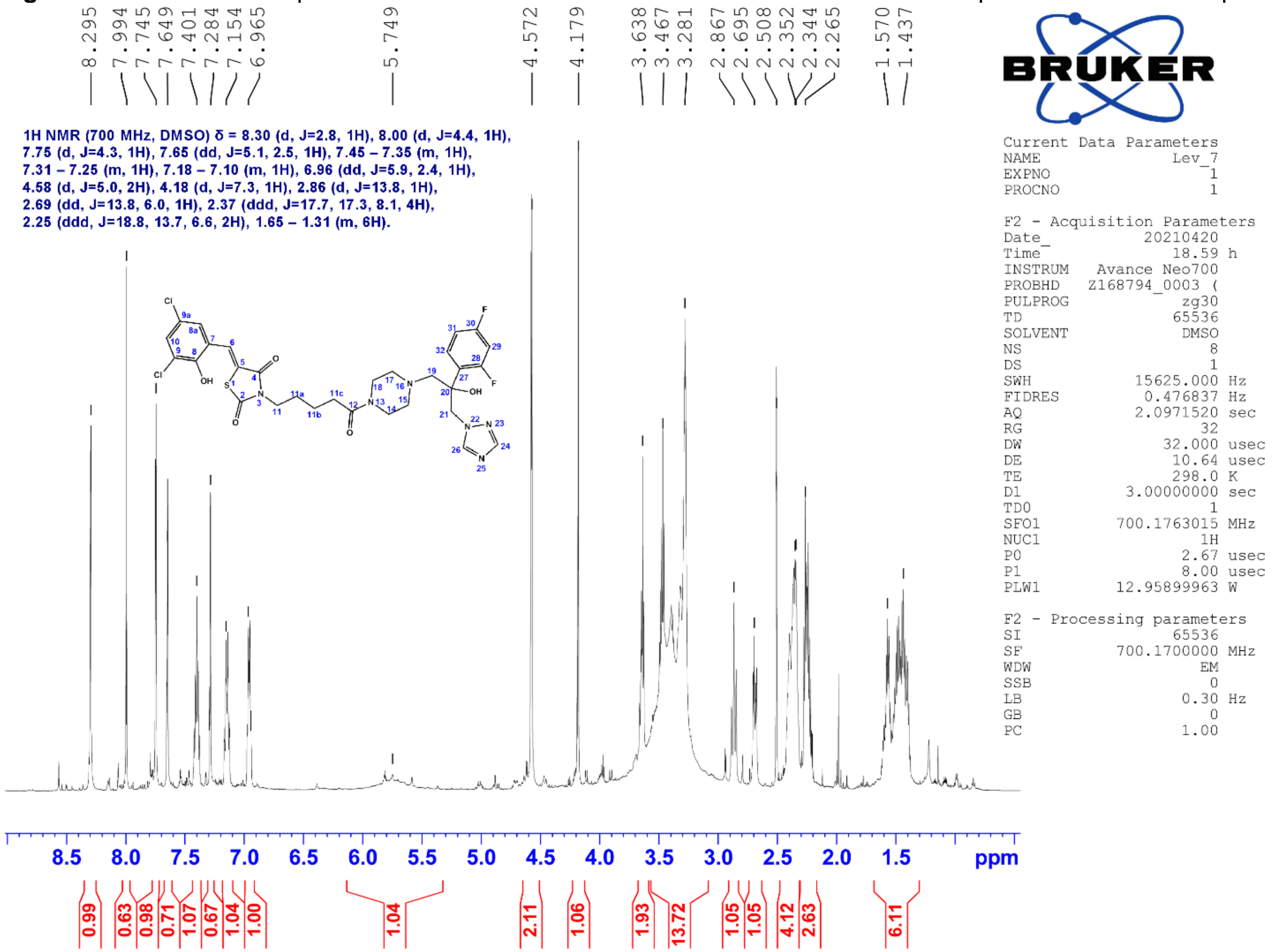

Figure S61. 1D ^{13}C NMR spectrum of **34h** recorded in DMSO-d_6 at 298K and 176 MHz carbon resonance frequency.

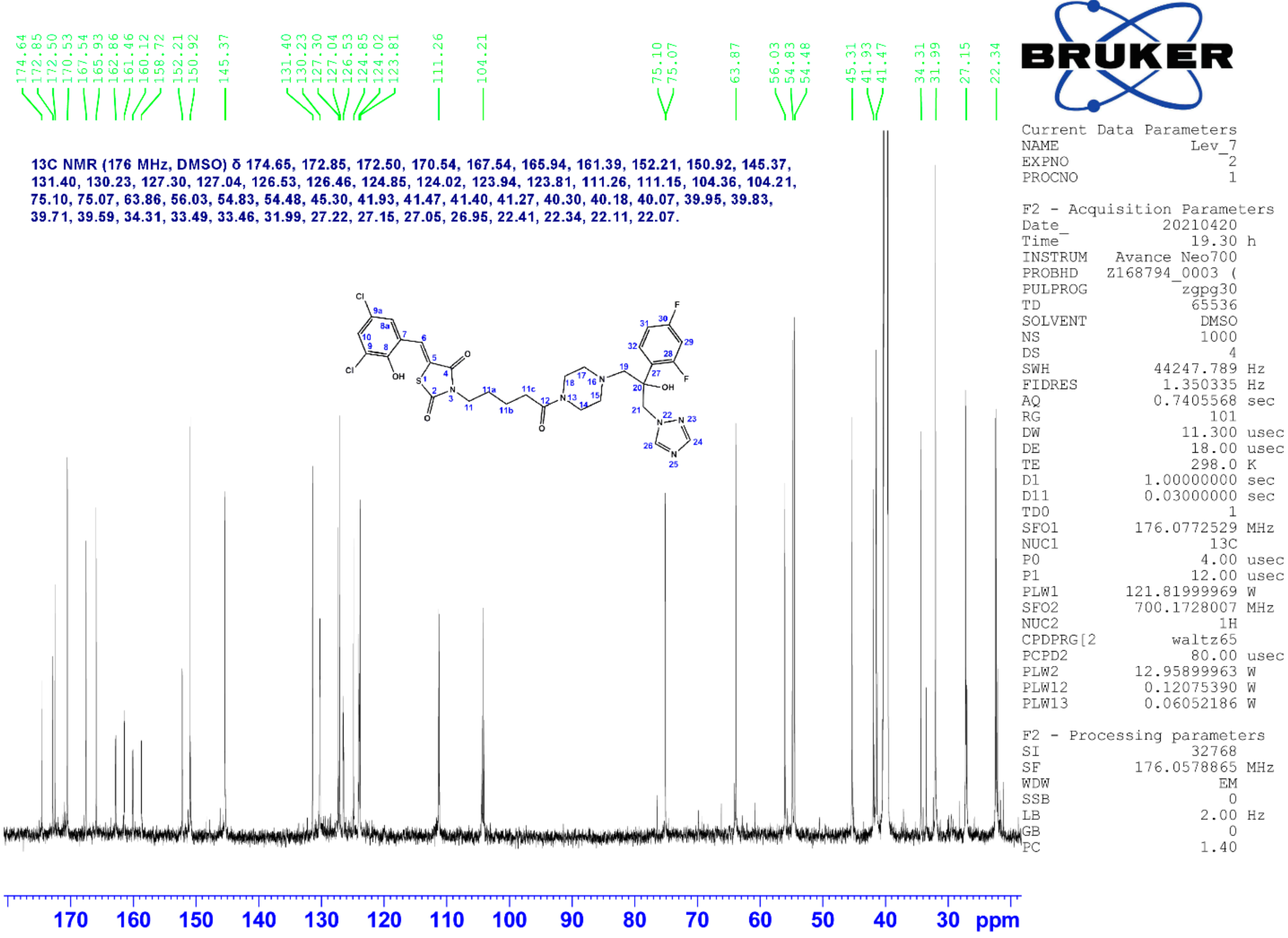

Figure S62. 1D ^{1}H NMR spectrum of **34i** recorded in DMSO-d_6 at 298K and 700 MHz proton resonance frequency.

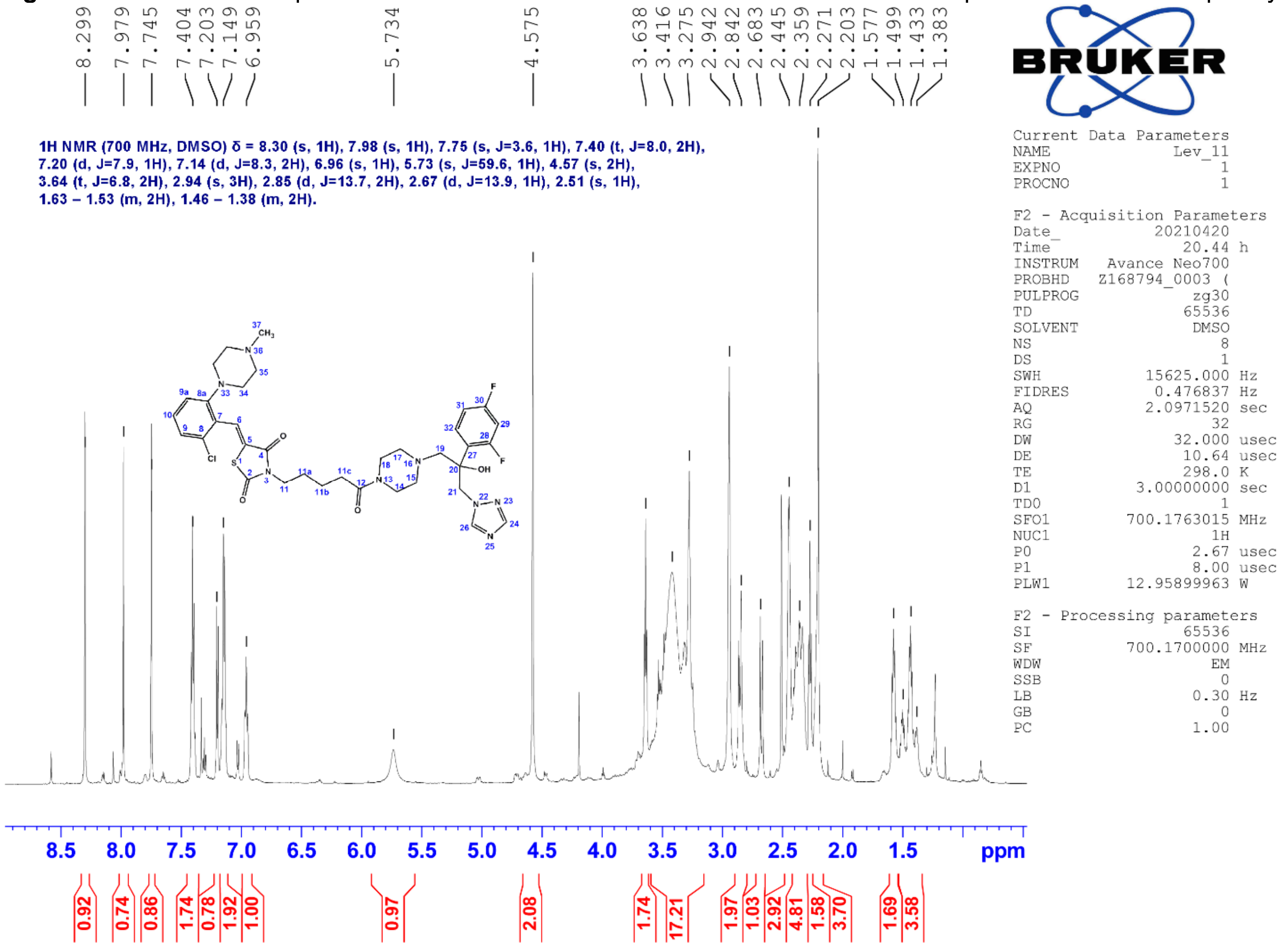

Figure S63. 1D ^{13}C NMR spectrum of **34i** recorded in DMSO-d$_6$ at 298K and 176 MHz carbon resonance frequency.

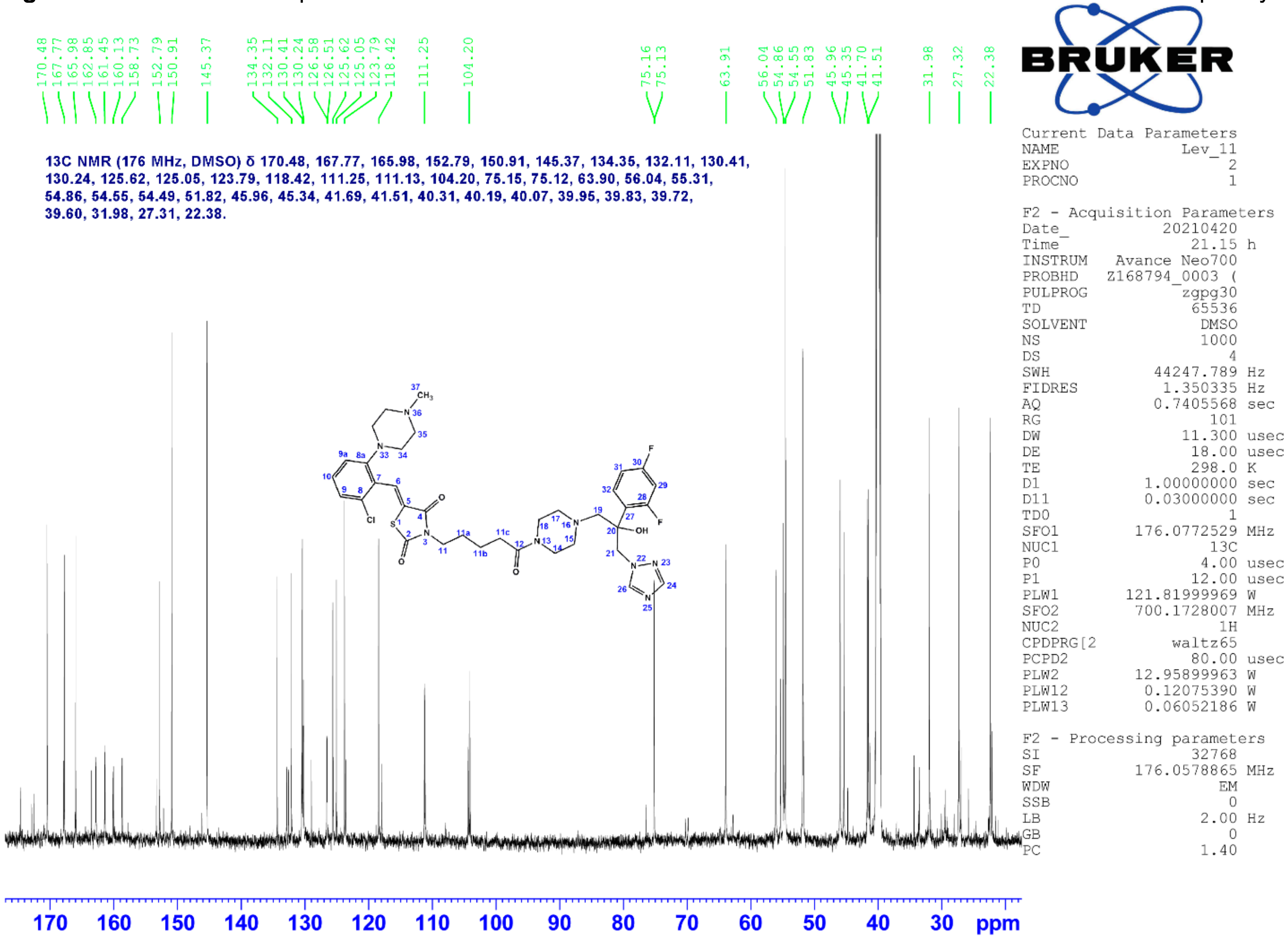

Figure S64. 1D ^{1}H NMR spectrum of **35b** recorded in DMSO-d$_6$ at 298K and 700 MHz proton resonance frequency

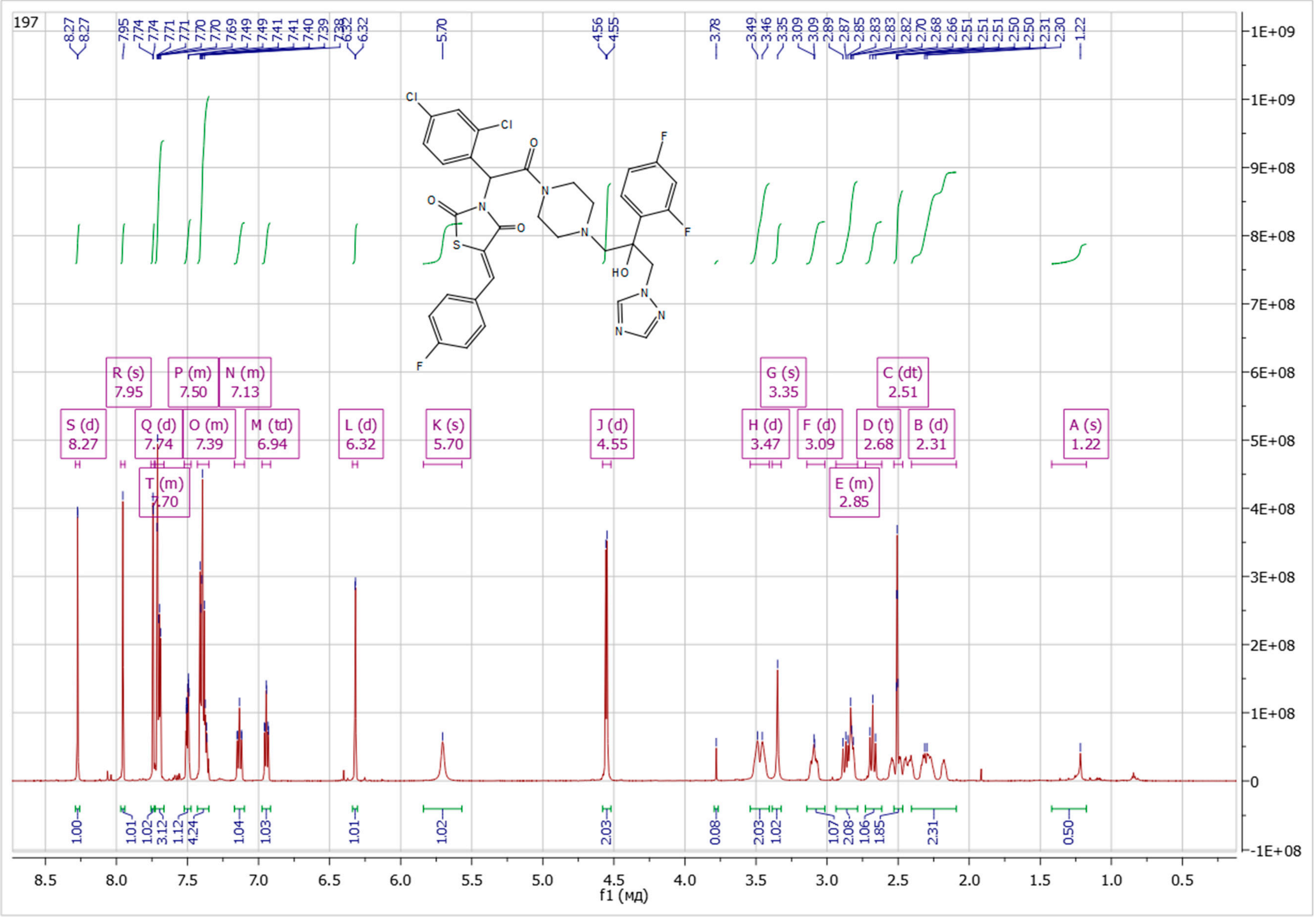

Figure S65. 1D ^{13}C NMR spectrum of **35b** recorded in DMSO-d_6 at 298K and 700 MHz proton resonance frequency

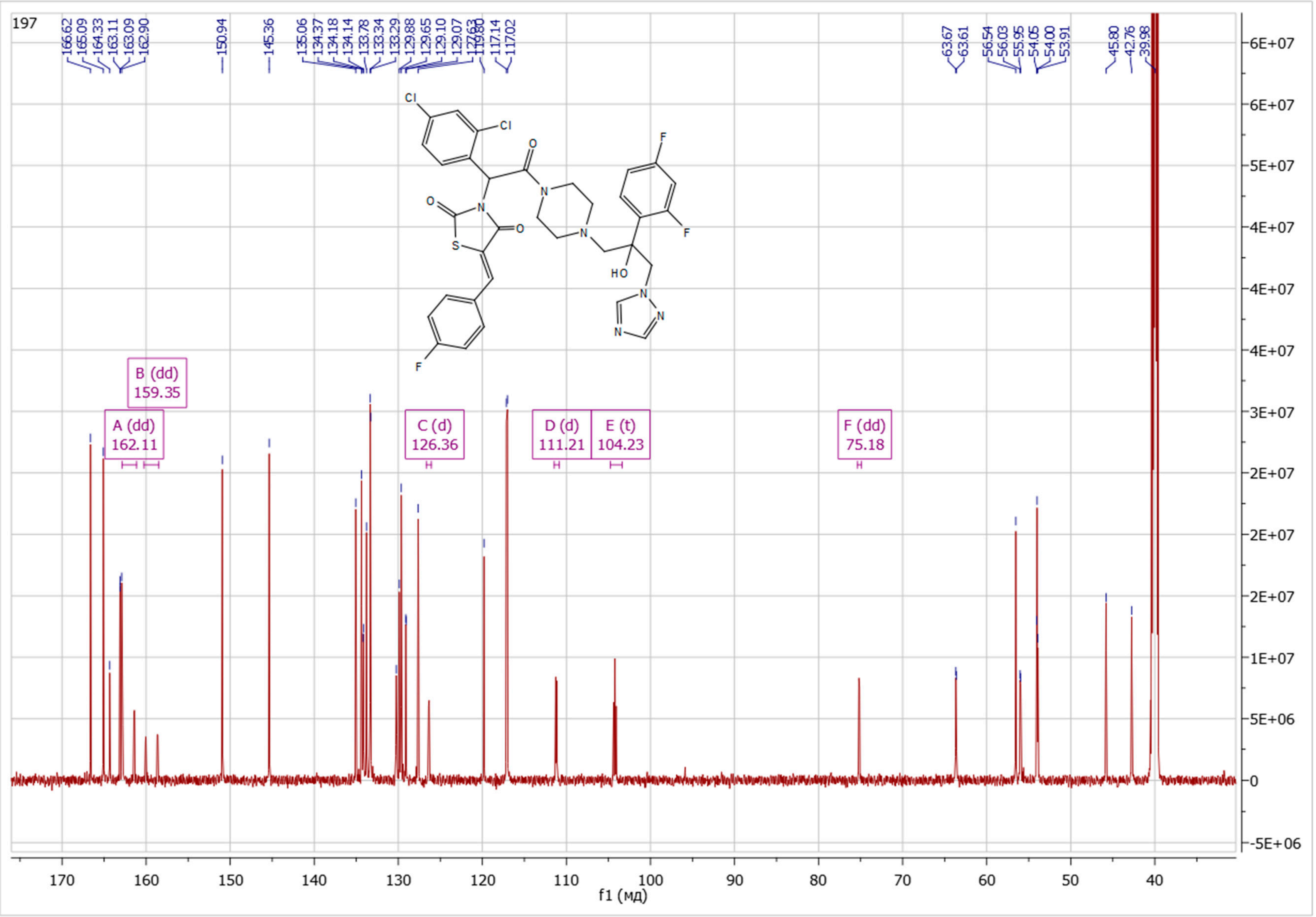

Figure S66. 1D ^{1}H NMR spectrum of **50a** recorded in DMSO-d_6 at 298K and 700 MHz proton resonance frequency.

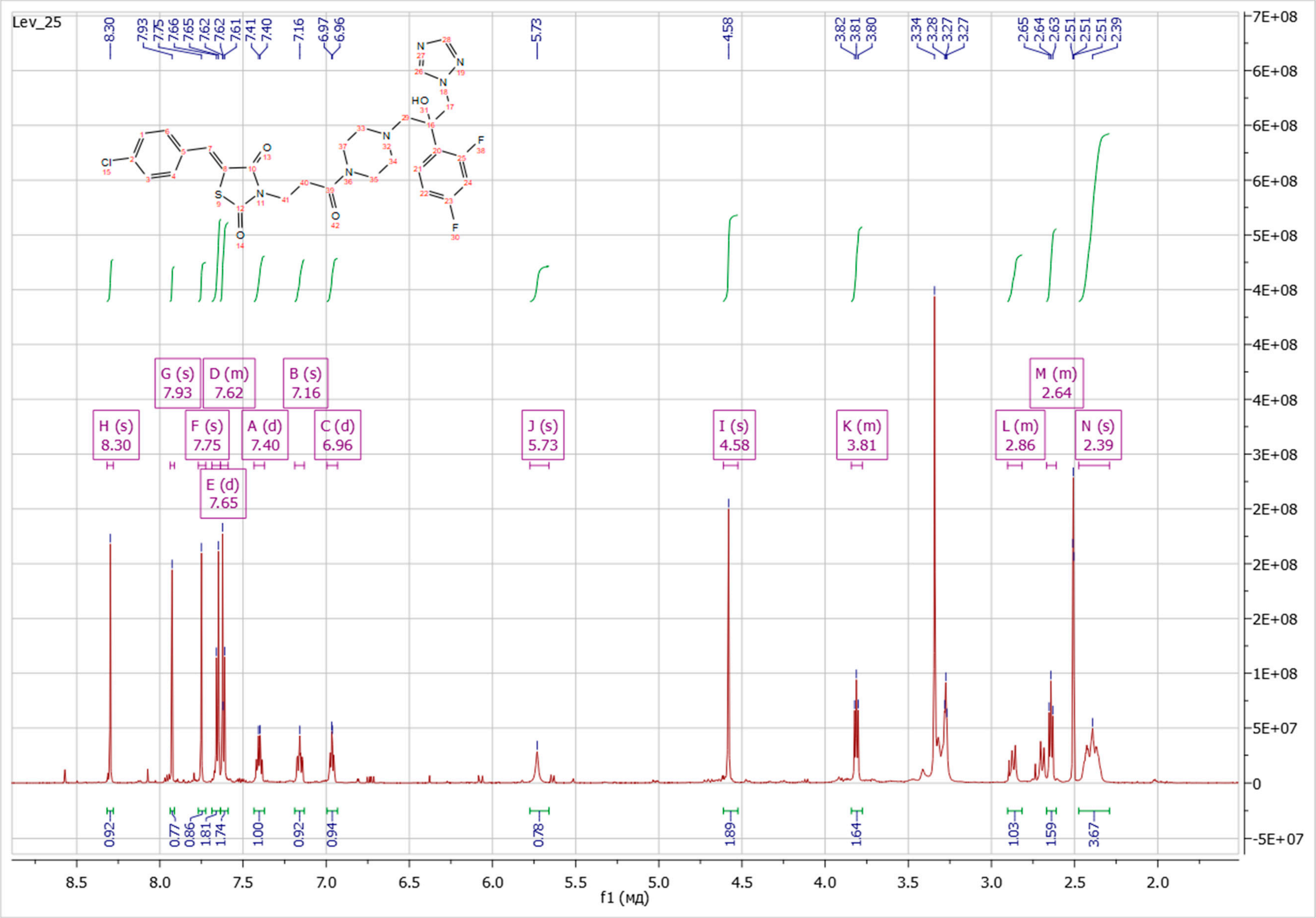

Figure S67. 1D ^{13}C NMR spectrum of **50a** recorded in DMSO-d$_6$ at 298K and 176 MHz carbon resonance frequency.

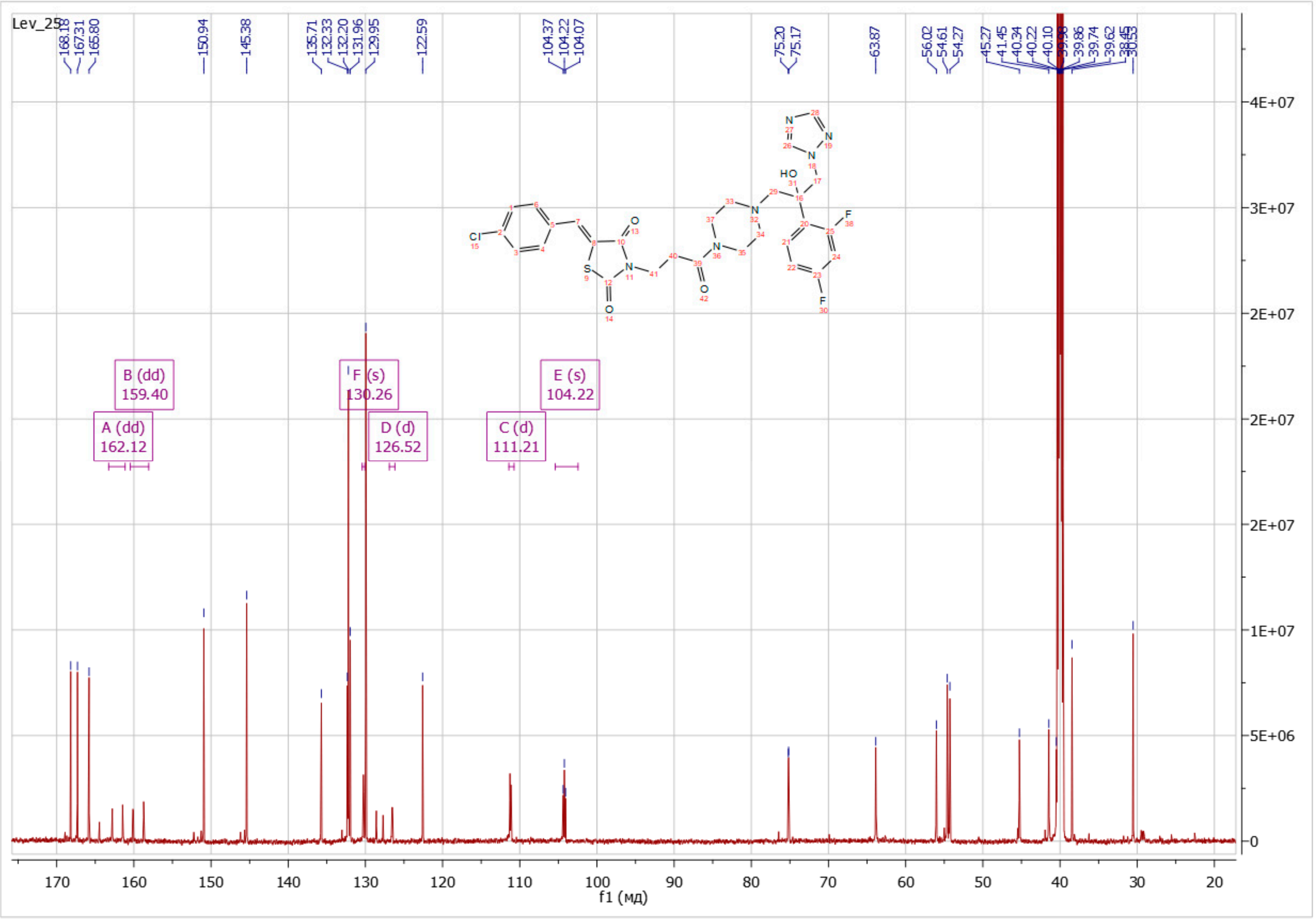

Figure S68. 1D ^{1}H NMR spectrum of **50c** recorded in DMSO-d_6 at 298K and 700 MHz proton resonance frequency.

1H NMR (700 MHz, DMSO) δ = 12.51 (s, 1H), 8.29 (s, 1H), 7.88 (s, 1H), 7.75 (s, 2H), 7.60 (d, J=8.8, 2H), 7.57 (d, J=8.8, 2H), 7.39 (dt, J=16.3, 8.2, 1H), 7.15 (ddd, J=14.6, 8.5, 4.0, 1H), 7.11 (t, J=8.2, 4H), 6.96 (ddd, J=11.7, 8.8, 3.4, 1H), 4.58 (s, 2H), 3.83 (d, J=4.3, 7H), 3.80 (s, 2H), 2.88 (dd, J=18.1, 9.9, 1H), 2.74 – 2.67 (m, 1H), 2.63 (s, 2H), 2.46 – 2.32 (m, 4H), 1.34 – 1.17 (m, 2H).

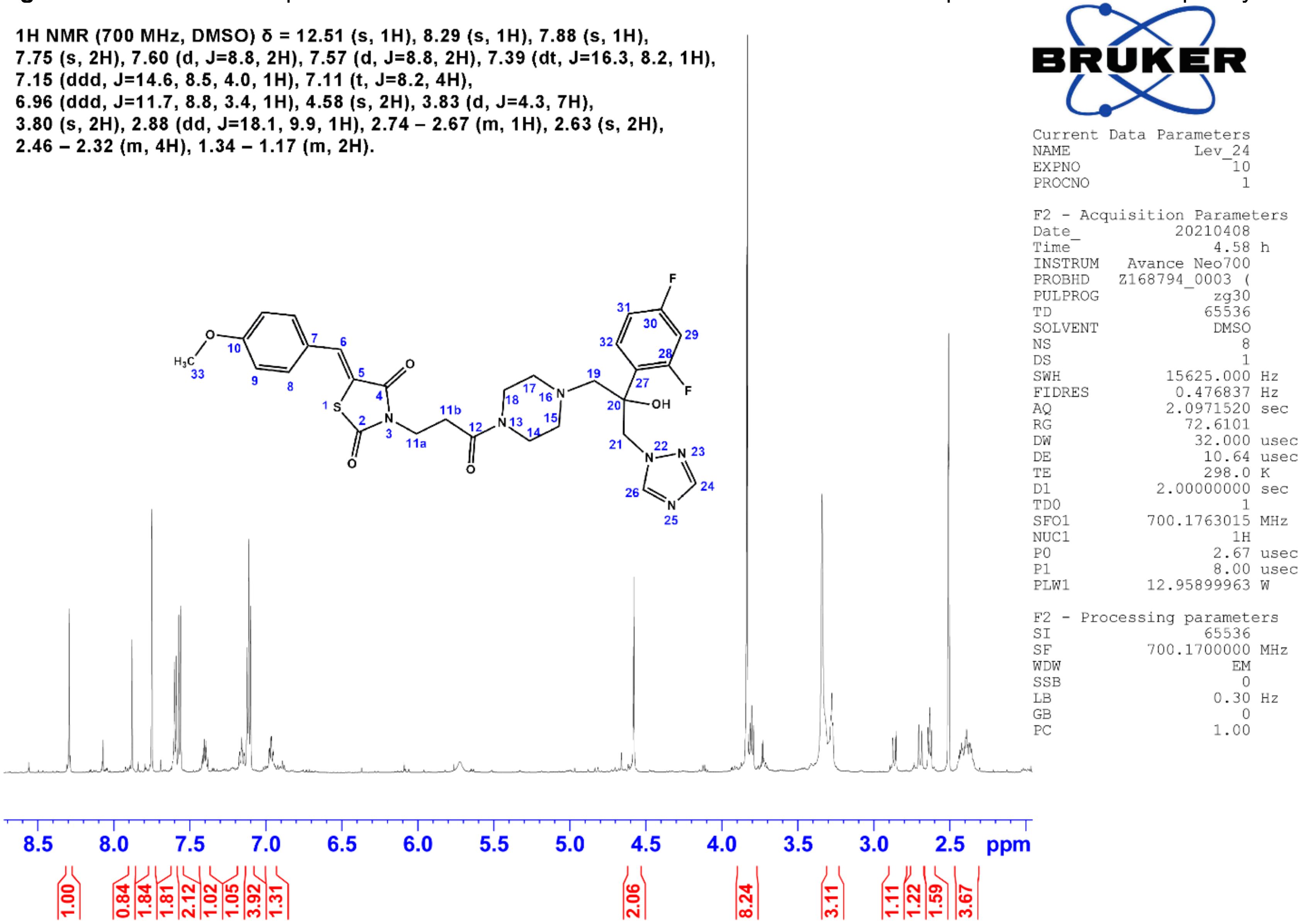

Figure S69. 1D ^{13}C NMR spectrum of **50c** recorded in DMSO-d_6 at 298K and 176 MHz carbon resonance frequency.

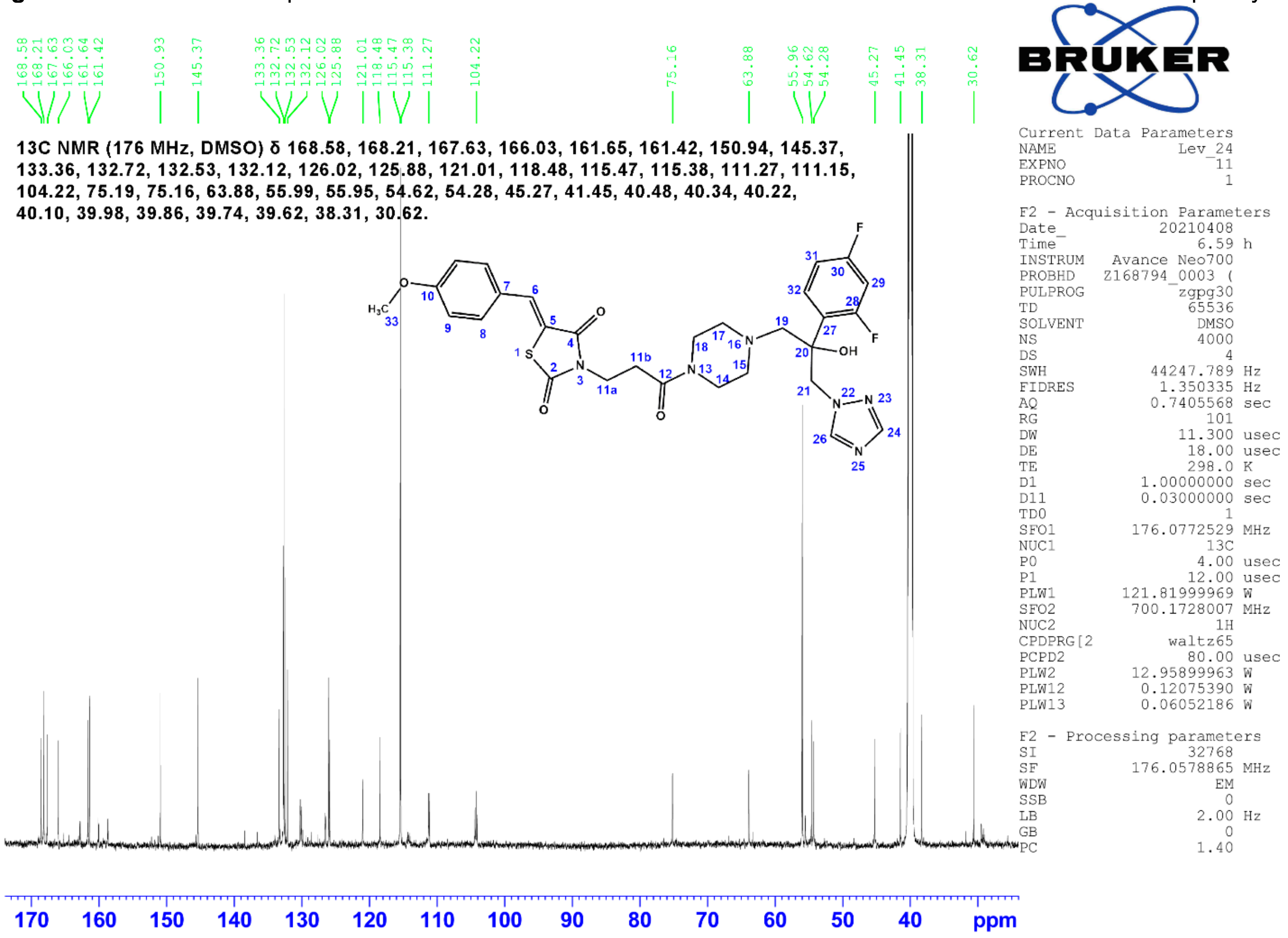

Figure S70. 1D ^{1}H NMR spectrum of **50g** recorded in DMSO-d_6 at 298K and 700 MHz proton resonance frequency.

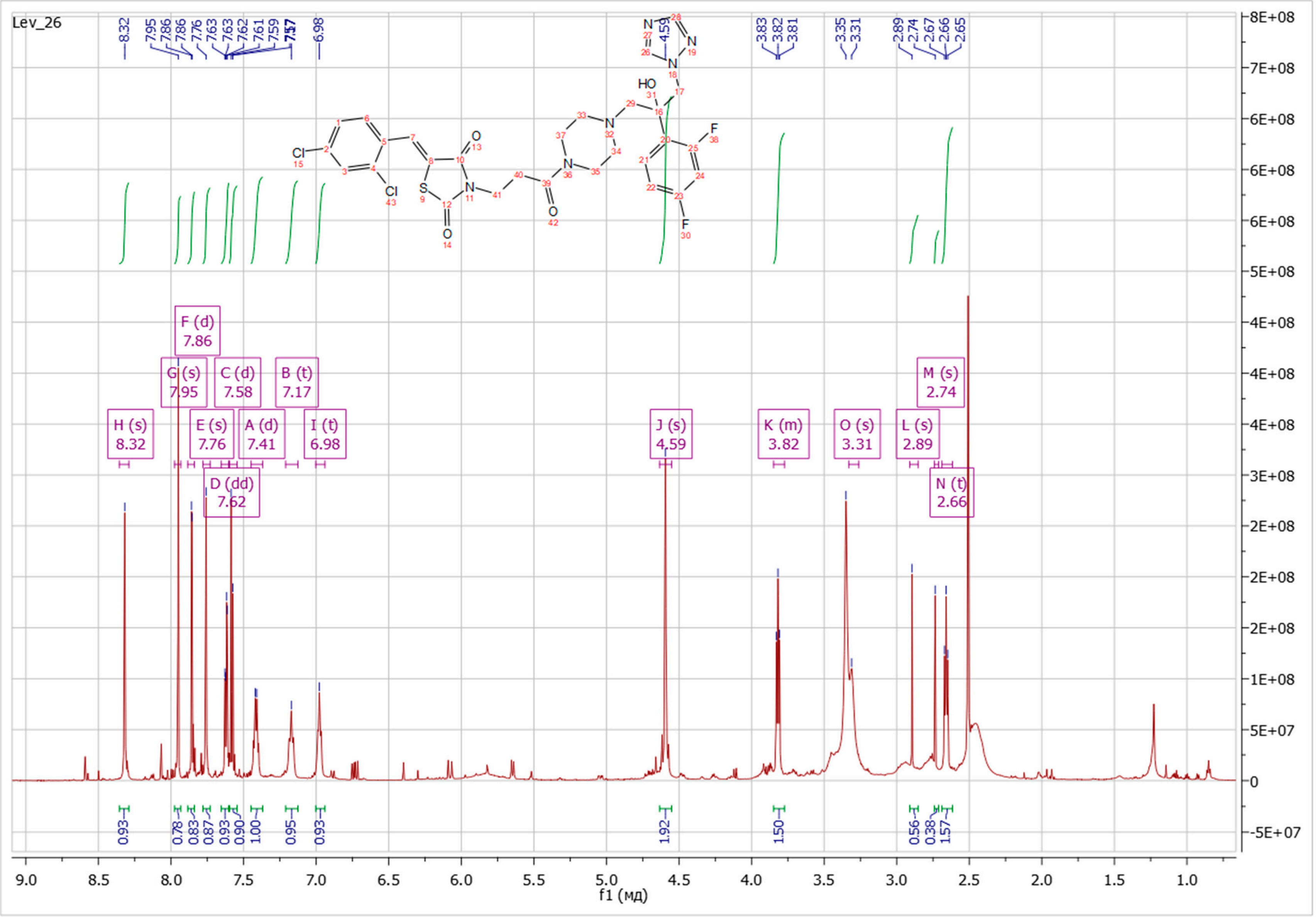

Figure S71. 1D ^{13}C NMR spectrum of **50g** recorded in DMSO-d_6 at 298K and 176 MHz carbon resonance frequency.

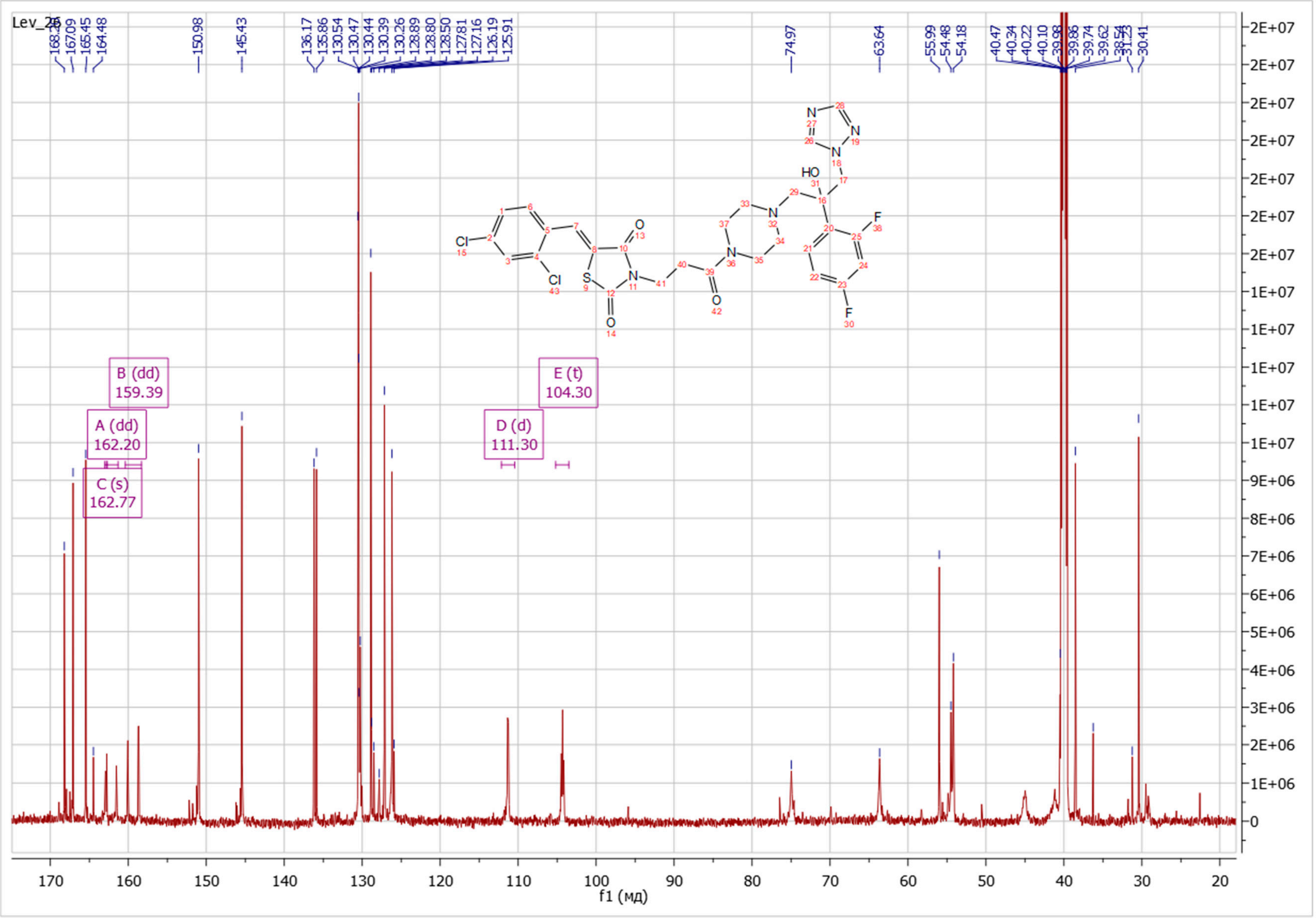

Figure S72. 1D ^{1}H NMR spectrum of **29a** recorded in DMSO-d$_6$ at 298K and 700 MHz proton resonance frequency.

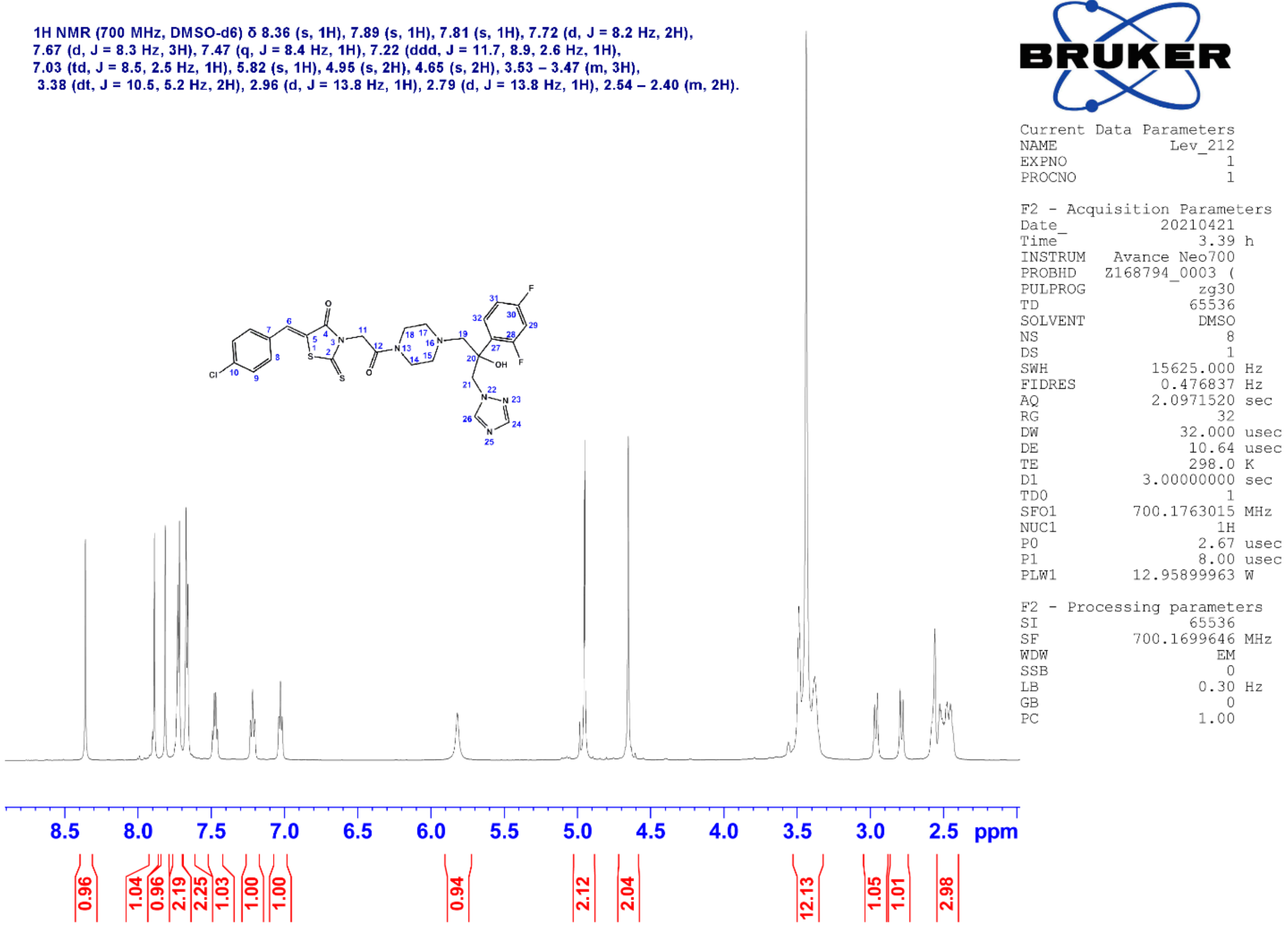

Figure S73. 1D ^{13}C NMR spectrum of **29a** recorded in DMSO-d_6 at 298K and 176 MHz carbon resonance frequency.

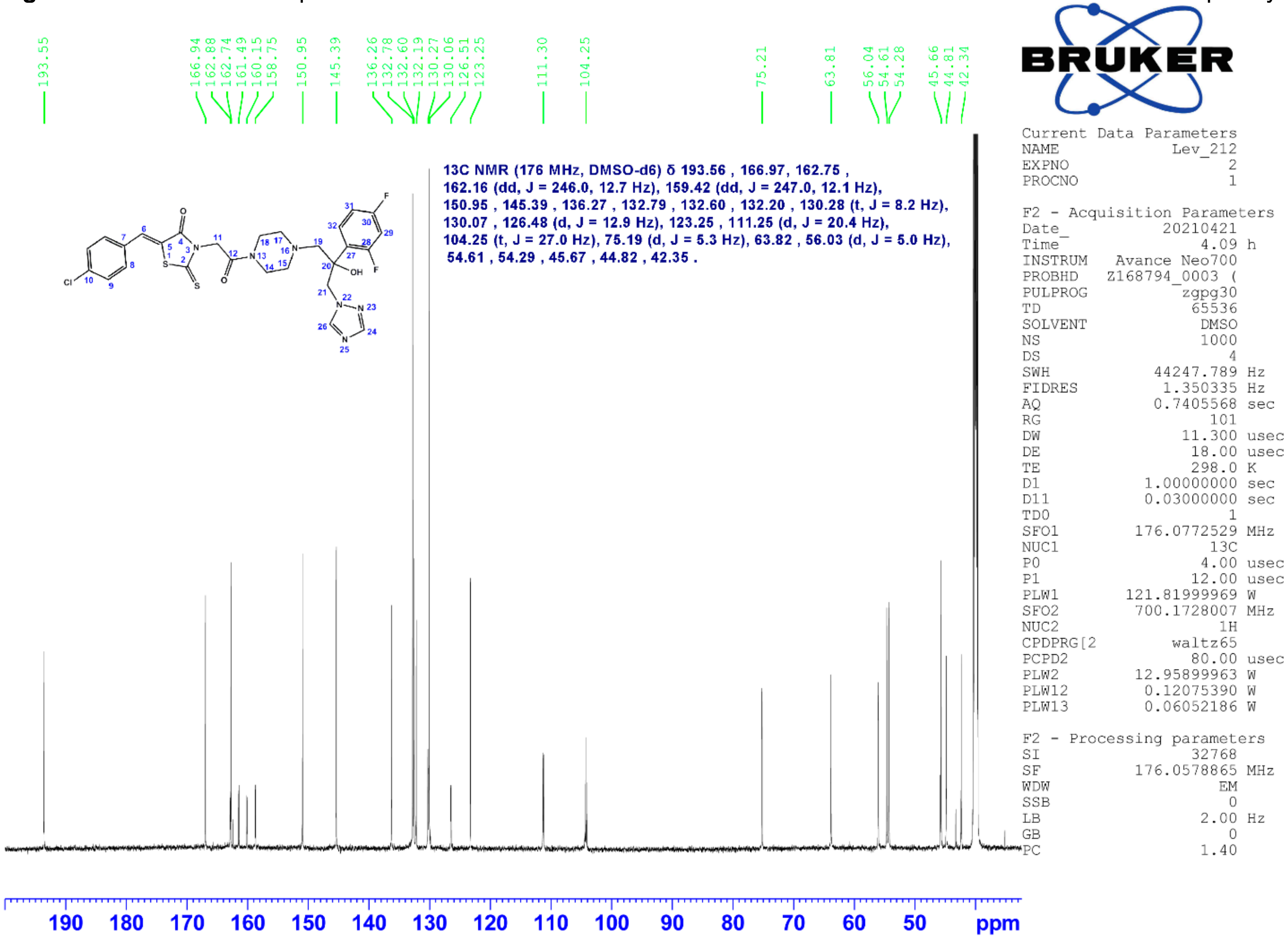

Figure S74. 1D ^{1}H NMR spectrum of **30a** recorded in DMSO-d_6 at 298K and 700 MHz proton resonance frequency.

1H NMR (700 MHz, DMSO) δ = 8.32 (s, 1H), 7.84 (s, 1H), 7.76 (s, 1H), 7.68 (d, J=8.4, 2H), 7.62 (d, J=8.3, 2H), 7.55 (s, 2H), 7.33 (d, J=7.8, 1H), 5.86 (s, 1H), 4.89 (s, 3H), 2.51 (s, 5H).

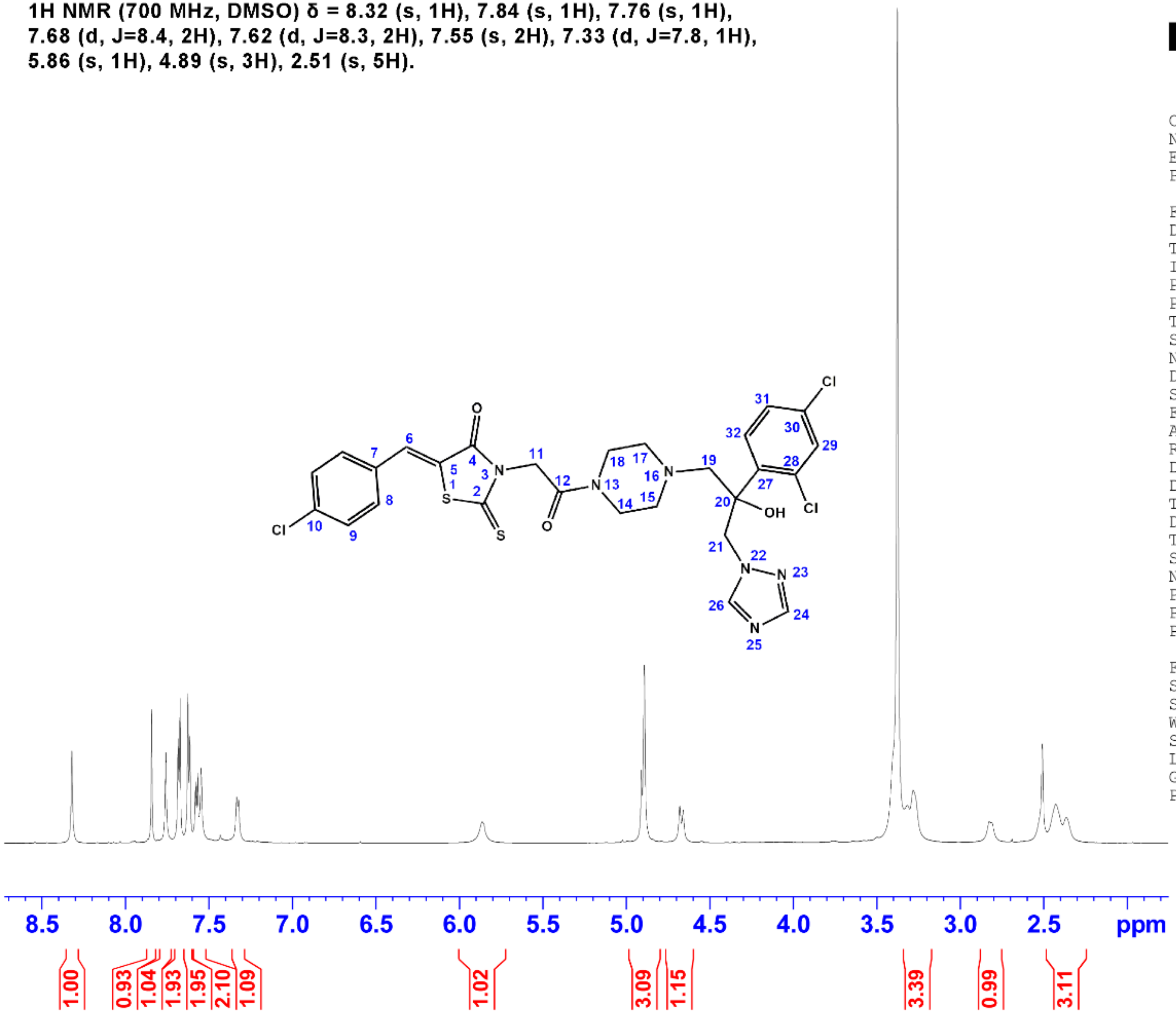


```
Current Data Parameters
NAME              Lev_17
EXPNO                  1
PROCNO                 1

F2 - Acquisition Parameters
Date_           20210420
Time               23.04 h
INSTRUM     Avance Neo700
PROBHD   Z168794_0003 (
PULPROG             zg30
TD                 65536
SOLVENT             DMSO
NS                     8
DS                     1
SWH            15625.000 Hz
FIDRES          0.476837 Hz
AQ             2.0971520 sec
RG                    32
DW                32.000 usec
DE                 10.64 usec
TE                 298.0 K
D1            3.00000000 sec
TD0                    1
SFO1         700.1763015 MHz
NUC1                  1H
P0                  2.67 usec
P1                  8.00 usec
PLW1         12.95899963 W

F2 - Processing parameters
SI                 65536
SF           700.1700000 MHz
WDW                   EM
SSB                    0
LB                  0.30 Hz
GB                     0
PC                  1.00
```

Figure S75. 1D ^{13}C NMR spectrum of **30a** recorded in DMSO-d_6 at 298K and 176 MHz carbon resonance frequency.

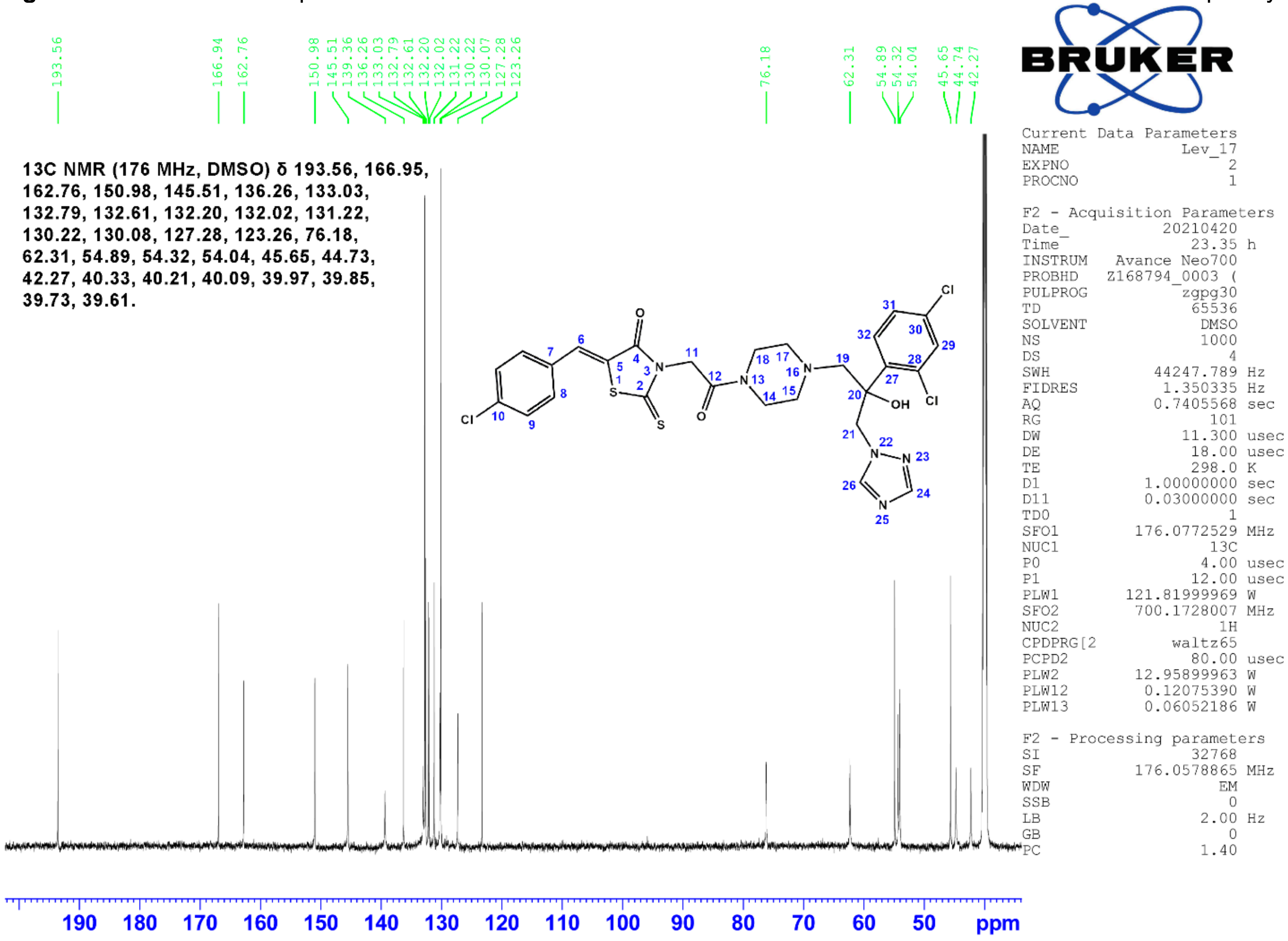

Figure S76. 1D ^{1}H NMR spectrum of **30c** recorded in DMSO-d$_6$ at 298K and 700 MHz proton resonance frequency.

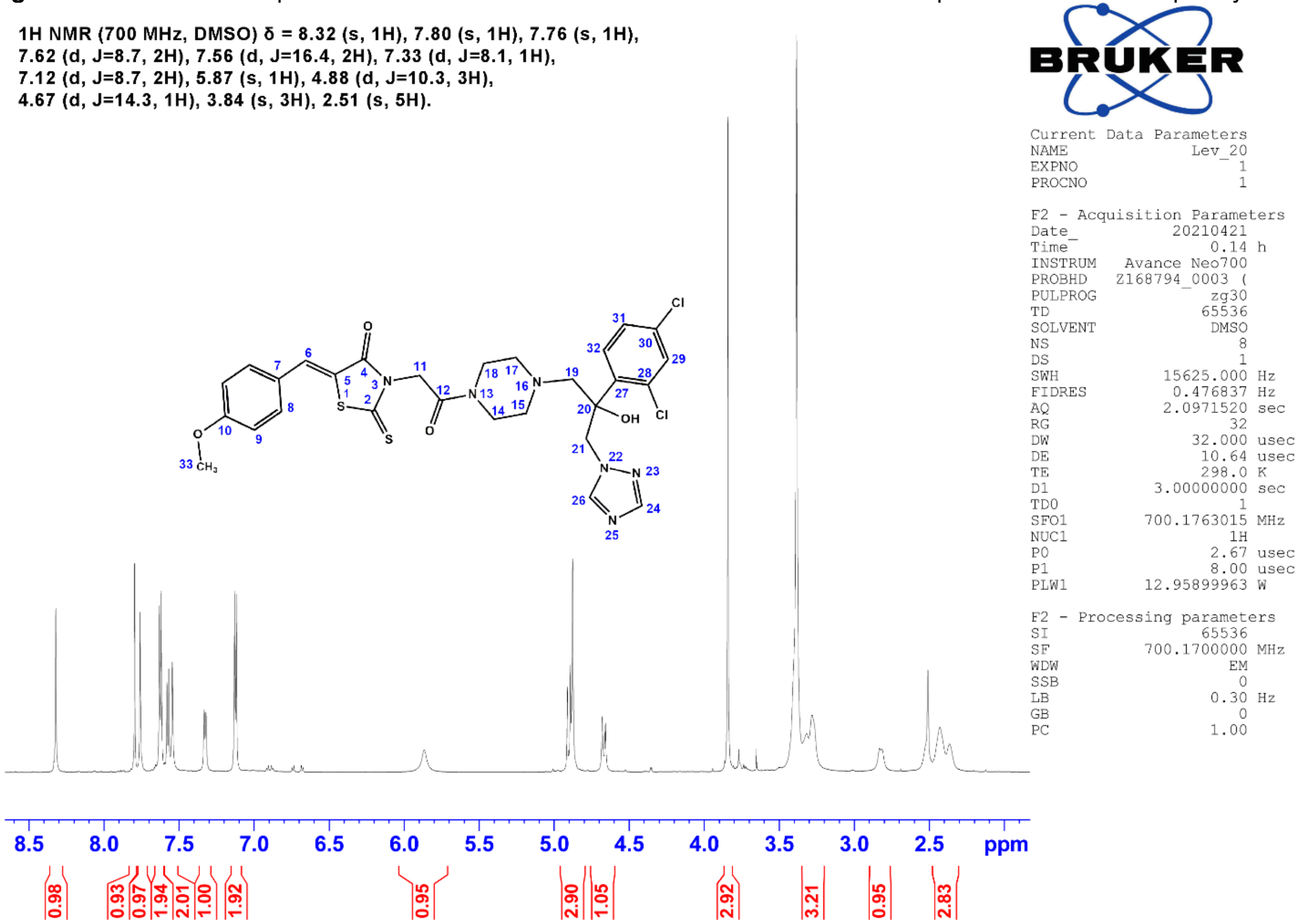

Figure S77. 1D ^{13}C NMR spectrum of **30c** recorded in DMSO-d_6 at 298K and 176 MHz carbon resonance frequency.

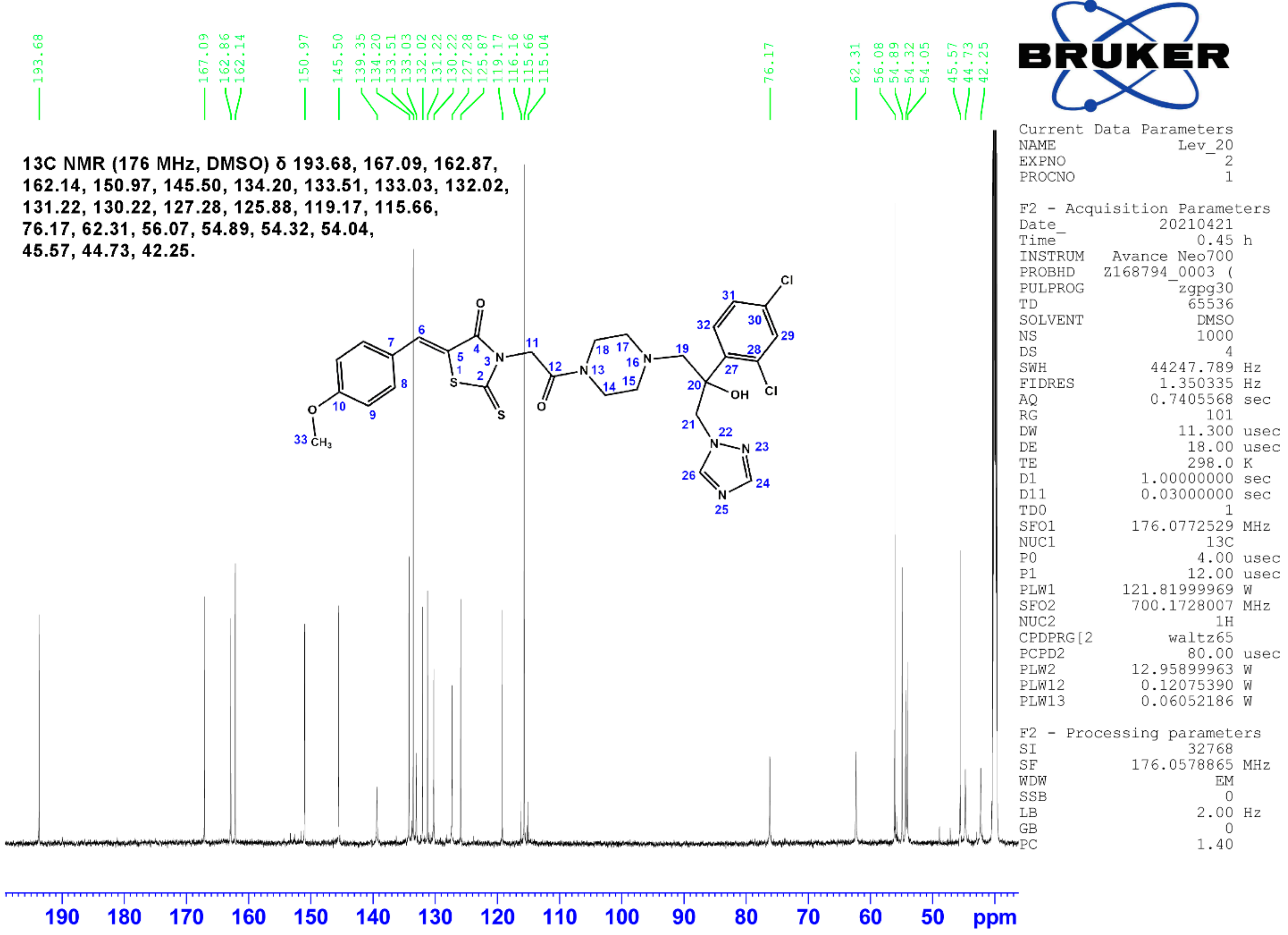

Supplement: Supplementary file 1 [file pharmaceuticals-17-00723-s001.zip › SUPPORTING INFORMATION-v5.pdf]
